# Deep learning for clustering of multivariate clinical patient trajectories with missing values

**DOI:** 10.1093/gigascience/giz134

**Published:** 2019-11-15

**Authors:** Johann de Jong, Mohammad Asif Emon, Ping Wu, Reagon Karki, Meemansa Sood, Patrice Godard, Ashar Ahmad, Henri Vrooman, Martin Hofmann-Apitius, Holger Fröhlich

**Affiliations:** 1 UCB Biosciences GmbH, Alfred-Nobel-Strasse 10, 40789 Monheim, Germany; 2 Fraunhofer Institute for Algorithms and Scientific Computing, Schloss Birlinghoven, Konrad-Adenauer-Strasse, 53754 Sankt Augustin, Germany; 3 Bonn-Aachen International Center for IT, University of Bonn, Konrad-Adenauer-Strasse, 53115 Bonn, Germany; 4 UCB Pharma, Bath Road 216, Slough SL1 3WE, UK; 5 UCB Pharma, Chemin du Foriest 1, 1420 Braine-l’Alleud, Belgium; 6 Erasmus MC, University Medical Center Rotterdam, Department of Radiology, Doctor Molewaterplein 40, PO Box 2040, 3000 CA Rotterdam, Netherlands; 7 Erasmus MC, University Medical Center Rotterdam, Doctor Molewaterplein 40, Department of Medical Informatics, PO Box 2040, 3000 CA Rotterdam, Netherlands

**Keywords:** patient stratification, deep learning, multivariate time series, multivariate longitudinal data, clustering

## Abstract

**Background:**

Precision medicine requires a stratification of patients by disease presentation that is sufficiently informative to allow for selecting treatments on a per-patient basis. For many diseases, such as neurological disorders, this stratification problem translates into a complex problem of clustering multivariate and relatively short time series because (i) these diseases are multifactorial and not well described by single clinical outcome variables and (ii) disease progression needs to be monitored over time. Additionally, clinical data often additionally are hindered by the presence of many missing values, further complicating any clustering attempts.

**Findings:**

The problem of clustering multivariate short time series with many missing values is generally not well addressed in the literature. In this work, we propose a deep learning–based method to address this issue, variational deep embedding with recurrence (VaDER). VaDER relies on a Gaussian mixture variational autoencoder framework, which is further extended to (i) model multivariate time series and (ii) directly deal with missing values. We validated VaDER by accurately recovering clusters from simulated and benchmark data with known ground truth clustering, while varying the degree of missingness. We then used VaDER to successfully stratify patients with Alzheimer disease and patients with Parkinson disease into subgroups characterized by clinically divergent disease progression profiles. Additional analyses demonstrated that these clinical differences reflected known underlying aspects of Alzheimer disease and Parkinson disease.

**Conclusions:**

We believe our results show that VaDER can be of great value for future efforts in patient stratification, and multivariate time-series clustering in general.

## Findings

### Background

In precision medicine, patients are stratified on the basis of their disease subtype, risk, prognosis, or treatment response by means of specialized diagnostic tests. An important question in precision medicine is how to appropriately model disease progression and accordingly decide on the right type and time point of therapy for an individual. However, the progression of many diseases, such as neurological disorders, cardiovascular diseases, diabetes, and obesity [1–5], is highly multifaceted and not well described by 1 clinical outcome measure alone. Classical univariate clustering methods are likely to miss the inherent complexity of diseases that demonstrate a highly multifaceted clinical phenotype. Accordingly, stratification of patients by disease progression translates into the challenging question of how to identify a clustering of a multivariate time series.

Clustering is a fundamental and generally well-investigated problem in machine learning and statistics. Its goal is to segment samples into groups (clusters), such that there is a higher degree of similarity between samples of the same cluster than between samples of different clusters. Following Hastie et al. [6], algorithms to solve clustering problems may be put into 3 main categories, (i) combinatorial algorithms, (ii) mixture modeling, and (iii) mode seeking. Within each of these 3 categories, a wide range of methods is available for a great diversity of clustering problems. Combinatorial algorithms do not assume any underlying probability model but work with the data directly. Examples are K-means clustering, spectral clustering [7], and hierarchical clustering [8]. Mixture models assume that the data can be described by some probabilistic model. An example is Gaussian mixture model clustering. Finally, in mode seeking one tries to directly estimate modes of the underlying multi-modal probability density. An important example here is the mean-shift algorithm [9].

For the clustering of multivariate time-series data, a few techniques have been developed [10–14]. However, these approaches generally rely on time series of far greater length than available in most longitudinal clinical datasets. Moreover, these methods are not suited for the large numbers of missing values often found in clinical data.

Missing values in clinical data can occur for different reasons: (i) patients drop out of a study, e.g., owing to worsening of symptoms; (ii) a certain diagnostic test is not taken at a particular visit (e.g., owing to lack of patient agreement), potentially resulting into missing information for entire variable groups; (iii) unclear further reasons, e.g., time constraints, data quality issues, etc. From a statistical point of view, these reasons manifest into different mechanisms of missing data [15,16]:

1. Missing completely at random (MCAR): The probability of missing information is related neither to the specific value that is supposed to be obtained nor to other observed data. Hence, entire patient records could be skipped without introducing any bias. However, this type of missing data mechanism is probably rare in clinical studies.
2. Missing at random (MAR): The probability of missing information depends on other observed data but is not related to the specific missing value that is expected to be obtained. An example would be patient dropout due to worsening of certain symptoms, which are at the same time recorded during the study.
3. Missing not at random (MNAR): any reason for missing data that is neither MCAR nor MAR. MNAR is problematic because the only way to obtain unbiased estimates is to model missing data.

Multiple-imputation methods have been proposed to deal with missing values in longitudinal patient data [16]. However, any imputation method will result in certain errors, and if imputation and clustering are performed separately, these errors will propagate through to the following clustering procedure.

To address the problem of clustering multivariate and relatively short time-series data with many missing values, in this article we propose an approach that uses techniques from deep learning. Autoencoder networks have been highly successful in learning latent representations of data (e.g., [17–20]). Specifically for clustering, autoencoders can be first used to learn a latent representation of a multivariate distribution, to then independently find clusters [21]. More recently, some authors have suggested simultaneously learning latent representations and cluster assignments. Interesting examples are deep embedded clustering [22] and variational deep embedding (VaDE) [23].

Here, we present a new method for clustering multivariate time series with potentially many missing values, variational deep embedding with recurrence (VaDER). VaDER is in part based on VaDE [23], a clustering algorithm based on variational autoencoder principles, with a latent representation forced towards a multivariate Gaussian mixture distribution. Additionally, VaDER (i) integrates 2 long short-term memory (LSTM) networks [24] into its architecture, to allow for the analysis of multivariate time series; and (ii) adopts an approach of implicit imputation and loss reweighting to account for the typically high degree of missingness in clinical data.

After a validation of VaDER via simulation and benchmark studies, we applied the method to the problem of patient stratification in Alzheimer disease (AD) and Parkinson disease (PD), using data from the Alzheimer’s Disease Neuroimaging Initiative (ADNI) [25] and the Parkinson’s Progression Markers Initiative (PPMI) [26], respectively. AD and PD are multifactorial and highly heterogeneous diseases, in both clinical and biological presentation, as well as in progression [27–30]. For example, PD is characterized by motor symptoms and behavioral changes (e.g., sleeping disorders), as well as cognitive impairment [31]. Cognitive impairment, one of the hallmarks of AD, is not straightforward to assess, because cognition itself is highly multifaceted, and described by, e.g., orientation, speech, and memory. Consequently, in the field of AD, a wide range of tests have been developed to assess different aspects of cognition.

This heterogeneity presents one of the major challenges in understanding these diseases and developing new treatments. As such, better clustering (stratification) of patients by disease presentation could be of great help in improving disease management and designing better clinical trials that specifically focus on treating patients whose disease is rapidly progressing.

Our analyses of the ADNI and PPMI data show that VaDER is highly effective at disentangling multivariate patient trajectories into clinically meaningful patient subgroups.

### Results

#### Variational autoencoders for clustering

Our proposed VaDER method is in part based on VaDE [23], a variational autoencoding clustering algorithm with a multivariate Gaussian mixture prior. In variational autoencoding algorithms, the training objective is to optimize the variational lower bound on the marginal likelihood of a data point **x** [32]:

(1) \begin{equation*}
\mathcal {L}({\bf x}) = \mathbb {E}_{{\it q}({\bf z}|{\bf x})} [\log (p({\bf x}|{\bf z}))] - D_{\mathrm{KL}}({\it q}({\bf z}|{\bf x})||p({\bf z})).
\end{equation*}

This lower bound can be seen as composed of 2 parts. The first term corresponds to the likelihood of seeing **x** given a latent representation **z**. Its negative is often called the "reconstruction loss," and it forces the algorithm to learn good reconstructions of its input data. The negative of the second term is often called the "latent loss." It is the Kullback-Leibler divergence of the prior *p*(**z**) to the variational posterior *q*(**z**|**x**), and it regularizes the latent representation **z** to lie on a manifold specified by the prior *p*(**z**).

In VaDE, this prior is a multivariate Gaussian mixture. Accordingly including a parameter for choosing a cluster *c*, the variational lower bound can then be written as follows:

(2) \begin{equation*}
\mathcal {L}({\bf x}) = \mathbb {E}_{{\it q}({\bf z},{\it c}|{\bf x})} [\log (p({\bf x}|{\bf z}))] - D_{\mathrm{KL}}({\it q}({\bf z},{\it c}|{\bf x})||p({\bf z},{\it c})).
\end{equation*}

By forcing the latent representation **z** towards a multivariate Gaussian mixture distribution, VaDE has the ability to simultaneously learn latent representations and cluster assignments of its input data. For more details on variational autoencoders and VaDE, we refer the reader to Jiang et al. [23], Kingma and Welling [32], and Doersch [33].

#### VaDER

VaDER is an autoencoder-based method for clustering multivariate time series with potentially many missing values. For simultaneously learning latent representations and cluster assignments of its input samples, VaDER uses the VaDE latent loss as described above and in Jiang et al. [23].

**Figure 1:**
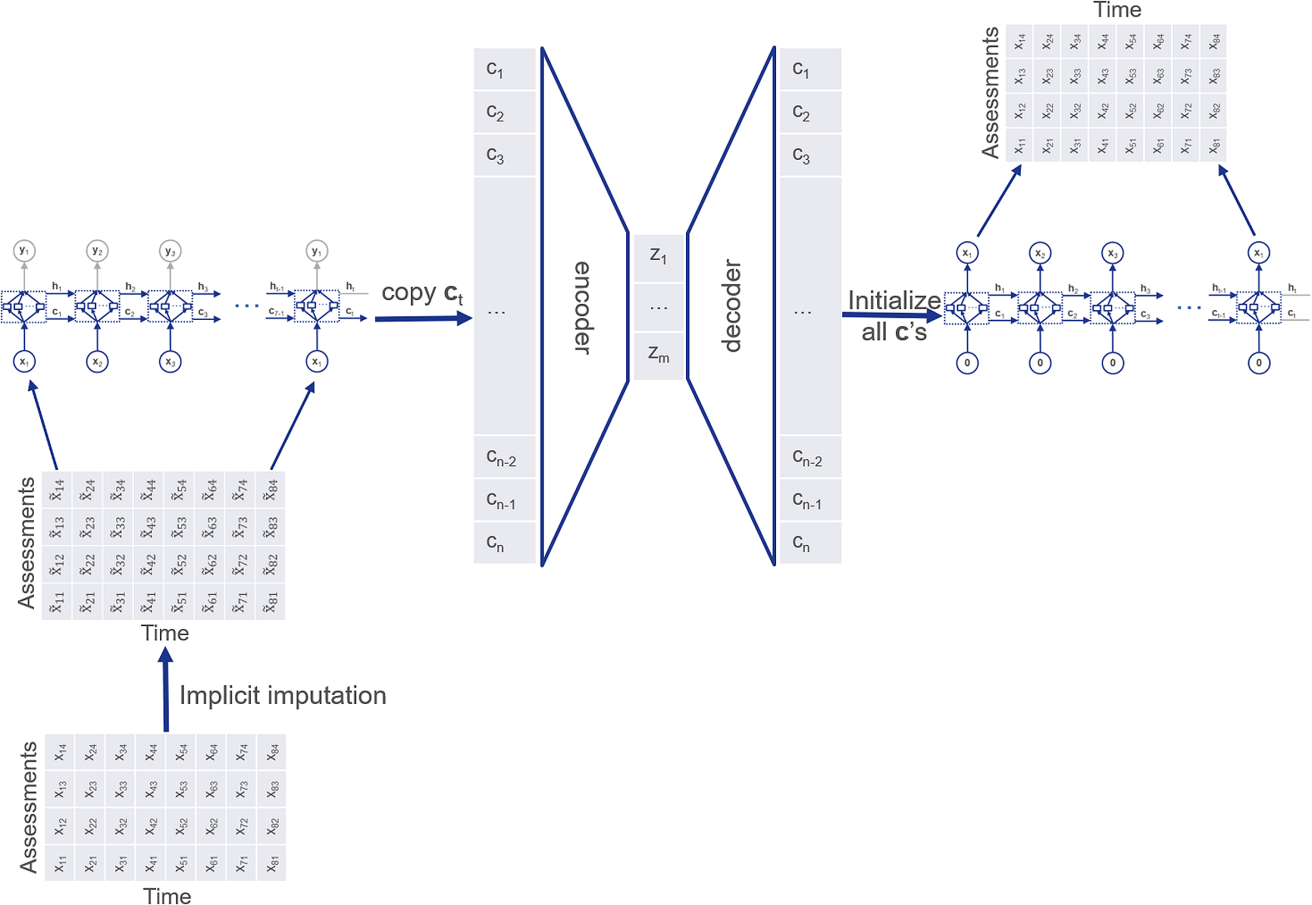
VaDER architecture.

To model the auto- and cross-correlations in multivariate time-series data, we integrate peephole LSTM networks [24,34] into the autoencoder architecture (Fig. 1).

To deal with missing values, we directly integrate imputation into model training. As outlined in the Background, separating imputation from clustering can potentially introduce bias. To avoid this bias, we here propose an implicit imputation scheme, which is performed within VaDER training. Our approach to imputation bears some similarity to other approaches [35,36]. However, in contrast to Lipton et al. [35], VaDER uses missingness indicators for implicit imputation as an integral part of neural network training. Additionally, in contrast to Manning et al. [36], our method of imputation is also suited for MNAR data, which are often encountered in clinical datasets.

We first define a weighted reconstruction loss on feature and sample level: imputed values are weighted to 0, non-imputed values are weighted to 1. To retain the balance with the latent loss, the resulting reconstruction loss is rescaled to match the original dimensions of the data. More formally, for a mean squared reconstruction loss, let *L* be the number of samples in our dataset, **x**^*l*^ a single input sample, and $\hat{{\bf x}}^l$ its corresponding reconstructed output (*l* ∈ 1…*L*). **x**^*l*^ and $\hat{{\bf x}}^l$ are matrices $\in \mathbb {R}^{N\times M}$, where *N* is the number of time points and *M* is the number of clinical outcome measures (e.g., cognitive assessments) for a particular patient. Then the unweighted mean reconstruction loss is

(3) \begin{equation*}
\frac{1}{L} \sum\nolimits _{l=1}^{L} \sum\nolimits _{i=1}^{N} \sum\nolimits _{j=1}^{M} \left(x_{ij}^l - \hat{x}_{ij}^l\right)^2.
\end{equation*}

Now, let $A := \lbrace x_{ij}^l|x_{ij}^l \textrm{is~missing}\rbrace$, **1**_*A*_(.) be the indicator function on set A, and |*A*| be the cardinality of *A*. Then, the weighted mean squared reconstruction loss is:

(4) \begin{equation*}
\frac{N M}{|A|} \sum\nolimits _{l=1}^{L} \sum\nolimits _{i=1}^{N} \sum\nolimits _{j=1}^{M} {\bf 1}_A(x_{ij}^l) \left(x_{ij}^l - \hat{x}_{ij}^l\right)^2.
\end{equation*}

In addition to the weighted reconstruction loss, we adopt an implicit imputation scheme, where imputed values are learned as an integral part of model training. More specifically, Let **x**^*l*^, *N*, *M*, $x_{ij}^l$, *A*, and **1**_*A*_(.) be defined as above. Also assume that all $x_{ij}^l$ for which ${\bf 1}_A(x_{ij}^l) = 1$ are initially imputed with arbitrary finite values. Then we add 1 additional layer before the input LSTM (Fig. 1) as follows:

(5) \begin{equation*}
\tilde{x}_{ij}^l = x_{ij}^l \times [1 - {\bf 1}_A(x_{ij}^l)] + b_{ij} \times {\bf 1}_A(x_{ij}^l).
\end{equation*}

Here, $x_{ij}^l$ is the actual observed (or missing) value of sample *l* at time points *i* and assessment *j*, and $\tilde{x}_{ij}^l$ serves as input to the LSTM. In other words, if $x_{ij}^l$ is missing, then it is replaced by *b_ij_* in $\tilde{{\bf x}}$. Parameters *b_ij_* are trained as an integral part of VaDER using stochastic gradient descent and can be considered (time, assessment)-specific expected values. Note that (i) the initial arbitrary imputation does not influence the eventual clustering and (ii) the implicitly imputed values are weighted to 0 in the reconstruction loss.

#### VaDER achieves high accuracy on simulated data

As a first step in technically validating VaDER, we simulated data with a known ground truth clustering and assessed how well VaDER was able to recover these clusters. A natural framework to this end is the vector autoregressive (VAR) model because (i) it can express serial correlation between time points, (ii) it can express cross-correlation between variables, and (iii) given a fully parameterized VAR process, one can simulate random trajectories from that VAR process.

More specifically, to generate clusters of multivariate time series, we simulated from VAR process mixtures, for different values of a clusterability parameter λ. The clusterability parameter λ influences how easily separable the simulated clusters are (see Section Simulation experiments). Sample data are provided in the Supplementary Figure S1. We used the cluster purity measure [37] to record how well the true clustering could be recovered (for more details, see Methods).

**Figure 2:**
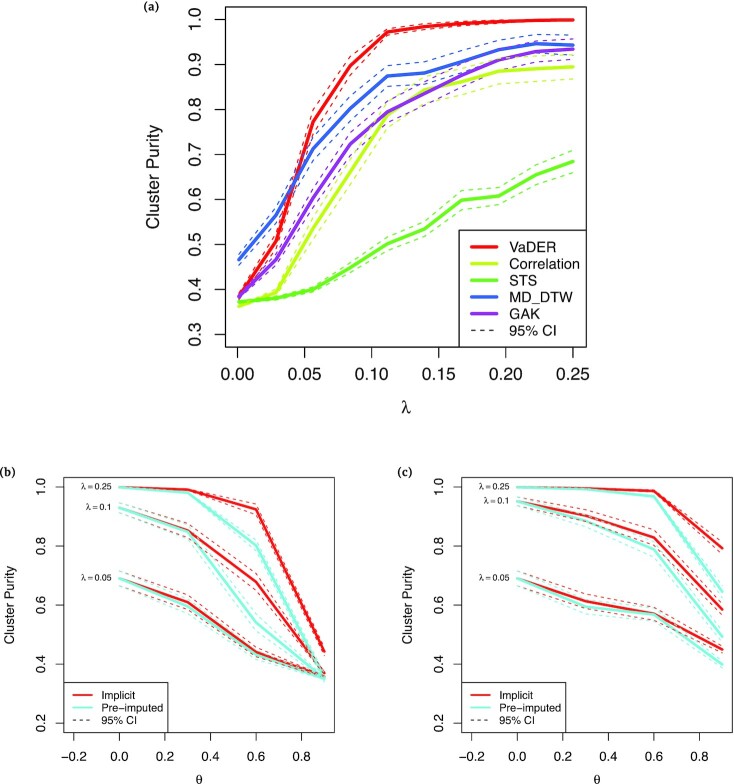
VaDER performance on simulated data, with varying degrees of clusterability and missingness. (a) Cluster purity [37] for clustering of simulated data as a function of the clusterability parameter λ, with higher λ implying a higher degree of similarity between profiles in the same cluster. Results are shown for VaDER as well as hierarchical clustering using 5 different distance measures, (i) Euclidean distance, (ii) Pearson correlation, (iii) the STS distance [40], (IV) multi-dimensional dynamic time warping (MD-DTW), [38] and (5) Global Alignment Kernels (GAK) [39]. (b) Cluster purity as a function of the fraction θ of values missing completely at random (MCAR), for various levels of the clusterability parameter λ, for both VaDER with implicit imputation and VaDER with pre-imputation. Confidence intervals were determined by repeating the clustering 100 times using newly generated random data and missingness patterns. (c) Cluster purity as a function of the fraction θ of values missing not at random (MNAR) (see Methods for details), for various levels of the clusterability parameter λ, for both VaDER with implicit imputation and VaDER with pre-imputation. Confidence intervals were determined by repeating the clustering 100 times using newly generated random data and missingness patterns.

VaDER was able to highly accurately recover the simulated clusters, achieving a cluster purity of >0.9 for λ ≈ 0.08, and converging to 1.0 for larger λ (Fig. 2a). Moreover, even without extensive hyperparameter optimization, VaDER performed substantially better than hierarchical clustering using various distance measures, some of which were specifically designed for multivariate time series (multidimensional dynamic time warping [MD-DTW] [38] and Global Alignment Kernels [GAK] [39]) or short univariate time series (the STS distance [40]). Only for λ < 0.04 was VaDER outperformed by MD-DTW. This may be attributed to the fairly limited number of samples used for the simulation (*n* = 2,000), and omitting extensive optimization of VaDER’s hyperparameters.

We used the same VAR framework to assess how varying degrees of missing values affect the performance of VaDER. Both MCAR and MNAR were simulated as described in the Methods. In the MCAR simulation, missing values were uniformly distributed across time and clinical outcome measures. In the MNAR simulation, the expected degree of missing values sigmoidally depended on time (see Methods). For varying clusterability levels λ, it can be seen that VaDER’s implicit imputation scheme is overall more robust against missing values than using VaDER with pre-imputation of missing values (Figs 2b and c).

#### VaDER achieves high accuracy on benchmark classification datasets

As an additional validation step towards applying VaDER to real-world clinical data, we collected a number of real-world benchmark datasets for multivariate time-series classification (Table 1). The datasets were normalized and processed to equal and/or shorter length as described in the Methods.

**Table 1.** Multivariate time-series classification datasets used in this study

Name	*k*	*n*	*p*	*n_t_*	$n^\prime _t$	Source
ArabicDigits	10	8,800	13	4 - 93	24	UCI [41]
JapaneseVowels	9	640	12	7 - 29	15	UEA/UCR [42]
CharacterTrajectories	20	2,858	3	109 - 205	23	UCI [41]
UWave	8	4,478	3	315	25	UCI [41]

*k*: number of classes; *n*: number of samples; *p*: number of variables; *n_t_*: number of time points; $n^\prime _t$: number of samples after processing to equal and/or shorter length; UCI: University of California Irvine machine learning repository; UEA/UCR: University of East Anglia/University of California, Riverside time-series classification archive.

**Figure 3:**
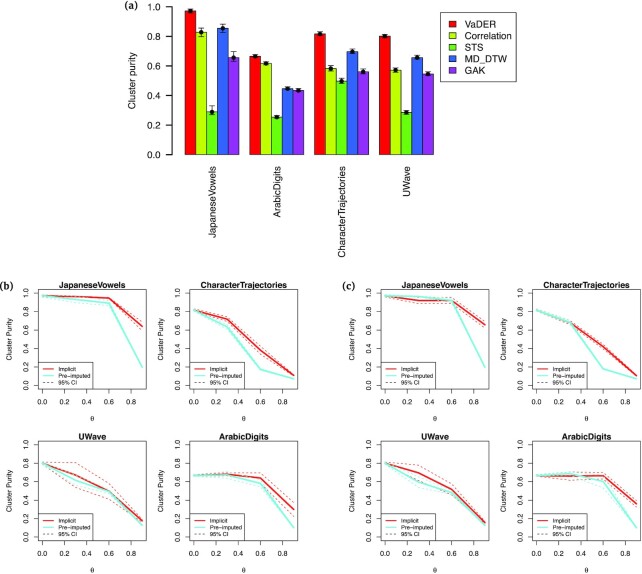
VaDER performance on benchmark data, for varying degrees of missingness. (a) Cluster purity [37] for clustering of benchmark data. Results are shown for VaDER as well as hierarchical clustering using 5 different distance measures, (i) Euclidean distance, (ii) Pearson correlation, (iii) the STS distance [40], (iv) multi-dimensional dynamic time warping (MD-DTW) [38], and (v) Global Alignment Kernels (GAK) [39]. For each dataset, the best performance across methods is marked by a horizontal dotted line. Confidence intervals were determined by bootstrapping the clustering 10^3^ times. (b) Cluster purity as a function of the fraction θ of values missing completely at random (MCAR), for both VaDER with implicit imputation and VaDER with pre-imputation. Confidence intervals were determined by repeating the clustering 5 times using newly generated random missingness patterns. (c) Cluster purity as a function of the fraction θ of values missing not at random (MNAR), for both VaDER with implicit imputation and VaDER with pre-imputation. Confidence intervals were determined by repeating the clustering 5 times using newly generated random missingness patterns.

Comparing the ability of VaDER in recovering these a priori known classes to the other methods mentioned above reveals that VaDER consistently achieves better results (Fig. 3a). Moreover, VADER’s approach of integrating imputation with model training again outperforms pre-imputation of missing values (Figs 3b and c).

#### Application 1: VaDER identifies clinically diverse AD patient subgroups

After the technical validation using simulated and benchmark data, we applied VaDER to clinical data for identifying meaningful patient subgroups. From ADNI [25], we collected data from 689 patients who at some point received a diagnosis of dementia during the course of this study. Four different cognitive assessment scores were available at 8 different visits: ADAS13, CDRSB, MMSE, and FAQ. We pre-processed the data as described in the ADNI data preparation section. Overall, the fraction of missing values was ∼43%. We used VaDER to cluster patients by disease progression as measured using these cognitive assessments.

Hyperparameter optimization was performed by random grid search as described in the Methods. For each number of clusters *k* ∈ {2…15}, the prediction strength [43] of the corresponding optimal model was compared to a null distribution (see Section Hyperparameter optimization and choice of number of clusters), which is shown in Supplementary Figure S2.

For most practical applications, determining an unambiguously correct number of clusters *k* is not possible, and a wide range of rules of thumb exist (see, e.g., [43–47]). For our subsequent analyses, we chose *k* = 3. This demonstrated relatively high prediction strength, significantly different from the null distribution, while still allowing VaDER to demonstrate its ability to uncover potentially interesting statistical interactions between trajectories of different cognitive assessments. A statistical interaction between different cognitive assessments could, e.g., manifest in the ability to distinguish patient subgroups based on 1 cognitive assessment, while this is not possible on another assessment. Another example would be a permuted ordering of clusters with respect to different assessment scores.

**Figure 4:**
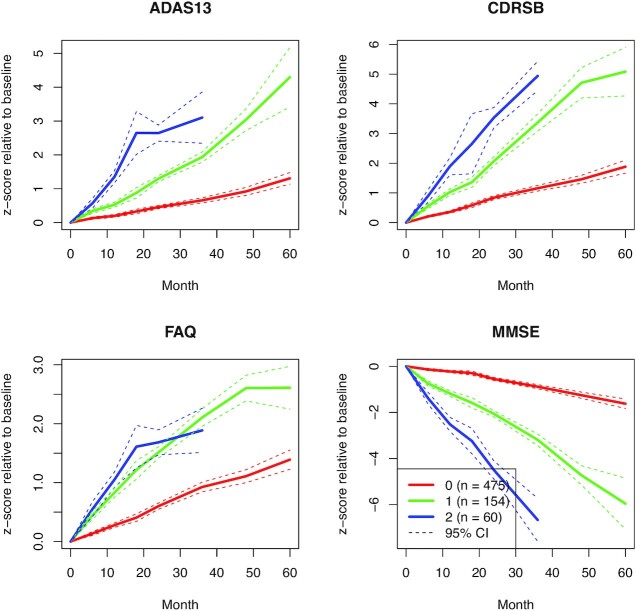
Normalized cluster mean trajectories relative to baseline (*x*-axis in months), as identified by VaDER from the ADNI cognitive assessment data.

For ADNI data the resulting cluster mean trajectories are shown in Fig. 4 and demonstrate that (i) VaDER effectively clusters the data into patient subgroups showing divergent disease progression and (ii) VaDER is able to find interactions between the different cognitive assessments, which would be principally difficult to distill from univariate analyses of the assessments. For example, the patients in Cluster 1 are the patients whose disease is the most severely progressing when assessed using ADAS13, CDRSB, and MMSE. However, the FAQ assessment (instrumental activities of daily living) does not distinguish between these patients with severely progressing disease and the patients with more moderately progressing disease in Cluster 1.

In addition to cognitive assessment measurements, ADNI presents a wealth of data on brain volume and various AD markers that we did not use for clustering. In these data, we identified numerous statistically significant associations with our patient subgroups. For example, the clusters strongly associated with time-to-dementia diagnosis relative to baseline, with Cluster 2 showing generally the shortest time and Cluster 0 the longest. The patients with relatively mildly progressing disease in cluster 0 also demonstrated on average a larger whole-brain volume at baseline, which moreover declined less steeply over time, compared to more patients with severely progressing disease. Especially the middle temporal gyri and fusiform gyri were larger (and shrinking more slowly over time), whereas the ventricles were smaller (and expanding more slowly over time). Indeed, atrophy of the middle temporal gyri and fusiform gyri, as well as ventricular enlargement, have been associated with AD progression [48,49]. As another example, the patients with more severely progressing disease (Cluster 1 and especially Cluster 2) demonstrated lower cerebral glucose uptake and lower cerebrospinal Abeta42 levels, again confirming the literature [50,51] (see Methods and Supplementary Figures S3-8). These observations demonstrate that the clinical differences between our patient subgroups reflect known AD aspects.

#### Application 2: VaDER identifies clinically diverse PD patient subgroups

We additionally applied VaDER to clinical data from the Parkinson’s Progression Markers Initiative (PPMI) [26]. From PPMI, we collected data from 362 *de novo* PD patients who had received a diagnosis within a period of 2 years before study onset and had initially not been treated. Nine variables on several motor and non-motor symptoms (UPDRS total, UPDRS1–3, tremor dominance [TD], postural instability and gait disturbance [PIGD], RBD, ESS, SCOPA-AUT) measured at either 5 or 10 time points were available. The data were pre-processed as described in the PPMI data preparation section. Overall, the fraction of missingness values was ∼17% (or ∼31%, when including time points entirely missing for some assessments). We again used VaDER to cluster patients according to disease progression as measured by these assessments.

**Figure 5:**
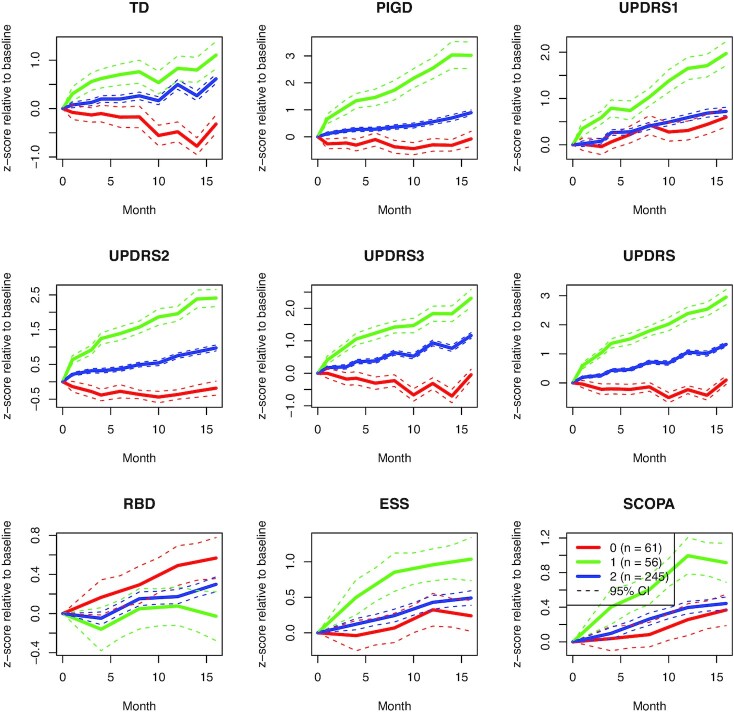
Normalized cluster mean trajectories relative to baseline (*x*-axis in months), as identified by VaDER from the PPMI motor/non-motor score data.

Hyperparameter optimization and selection of the number of clusters was performed in the same way as for ADNI (see Supplementary Figure S9), and we decided on *k* = 3 patient subgroups accordingly. The resulting cluster mean trajectories are shown in Fig. 5. These again illustrate that (i) VaDER effectively clusters the data into clinically divergent patient subgroups, and (ii) VaDER is able to find interactions between the assessments that would principally be difficult to find based on univariate analyses alone. For example, Cluster 0 represents patients with a moderate progression in terms of mental impairment, behavior, and mood (UPDRS1) and autonomic dysfunction (SCOPA). However, these patients remain relatively stable, or even improve, on many other assessments, such as TD, the self-reported ability to perform activities of daily life (UPDRS2), and motor symptoms evaluation (UPDRS3).

Similar to ADNI, PPMI presents a wealth of additional data on brain volume and various PD markers that were not used for clustering. Aligning these data with our PD patient subgroups, we found numerous statistically significant associations that confirmed existing literature, many related to quality of life and physiological changes to the brain. For example, men were over-represented in cluster 1 and showed the most severe disease progression, confirming the literature on sex differences in PD (e.g., [52]). Moreover, these patients with severely progressing disease showed an expected steeply declining ability to perform activities of daily living (modified Schwab and England score [53]), as well as rapidly developing symptoms of depression (geriatric depression scale [54]), common in patients with PD [55]. Additionally, these patients demonstrated physiological differences in the brain when compared to patients with more mildly progressing disease. Examples are the caudate nucleus and putamen brain regions, which were smaller at baseline and during follow-up examinations in the patients with more severely progressing disease in Cluster 1 and, from the literature, are known to be subject to atrophy in PD [56] (see Methods and Supplementary Figures S10-15). These observations demonstrate that the clinical differences between our patient subgroups reflect known aspects of PD disease progression.

### Discussion and conclusions

Identifying subgroups of patients with similar progression patterns can help to better elucidate the heterogeneity of complex diseases. Together with predictive machine learning methods, this might improve decision making on the right time and type of treatment for an individual patient, as well as the design of clinical studies. However, one of the main challenges is the multifaceted nature of progression in many areas of disease.

In this article, we proposed VaDER, a method for clustering multivariate, potentially short, time series with many missing values, a setting that seems generally not well addressed in the literature so far but is nonetheless often encountered in clinical study data.

We validated VaDER by showing the very high accuracy on clustering simulated and real-world benchmark data with a known ground truth. We then applied VaDER to data from (i) ADNI and (ii) PPMI, resulting in subgroups characterized by clinically highly divergent disease progression profiles. A comparison with other data from ADNI and PPMI, such as brain imaging and motor and cognitive assessment data, furthermore supported the observed patient subgroups.

VaDER has 2 main distinctive features. One is that VaDER deals directly with missing values. For clinical research this is crucial because clinical datasets often show a very high degree of missing values [57,58]. The other main distinctive feature is that, as opposed to existing methods [10–14], VaDER is specifically designed to deal with multivariate and relatively short time series that are typical for (observational) clinical studies. However, it is worthwhile to mention that the application of VaDER is not per se limited to longitudinal clinical study data or to time series of short length. Future applications (potentially requiring some adaptations) could, e.g., include data originating from electronic health records, multiple wearable sensors, video recordings, or time-series gene (co-)expression. Moreover, VaDER could be used as a generative model: given a trained model, it is possible to generate “virtual” patient trajectories.

Altogether, we believe that our results show that VaDER has the potential to substantially enhance future patient stratification efforts and multivariate time series clustering in general.

## Methods

### Data preparation

#### ADNI data preparation

Data used in the preparation of this article were obtained from the ADNI database (adni.loni.usc.edu). The ADNI was launched in 2003 as a public-private partnership, led by Principal Investigator Michael W. Weiner, MD. The primary goal of ADNI has been to test whether serial magnetic resonance imaging (MRI), positron emission tomography, other biological markers, and clinical and neuropsychological assessment can be combined to measure the progression of mild cognitive impairment and early AD. For up-to-date information, see www.adni-info.org.

The ADNIMERGE R-package [59] contains mainly 2 categories of data, (i) longitudinal and (ii) non-longitudinal. These data represent 1,737 participants that include healthy controls and patients with a diagnosis of AD. The non-longitudinal features such as demographic characteristics and apolipoprotein E4 status were measured only once, at baseline. The longitudinal features (i.e., neuroimaging features, cerebrospinal fluid biomarkers, cognitive tests, and everyday cognition) were recorded over a span of 5 years.

##### Clinical data

In the present study, we have focused on those participants who received a diagnosis of AD at baseline or during 1 of the follow-up visits. After this filtering step, we had a total of 689 patients. For these 689 patients, 4 cognitive assessments were selected for clustering:

- ADAS-13: Alzheimer's Disease Assessment Scale
- CDRSB: Clinical Dementia Rating Sum of Box Score
- FAQ: Functional Activities Questionnaire
- MMSE: Mini–Mental State Examination

The above assessments were taken at baseline and at 6, 12, 18, 24, 36, 48, and 60 months after baseline. For each of the 4 cognitive assessments, all time points were normalized relative to baseline by (i) subtracting the baseline mean across the 689 patients and (ii) dividing by the baseline standard deviation across the 689 patients.

##### Imaging data

All available MRI scans (T1-weighted scans) from the ADNI database were quantified by an open-source, automated segmentation pipeline at the Erasmus University Medical Center, The Netherlands. The number of slices of the T1-weighted scans varied from 160 to 196 and the in-plane resolution was 256 × 256 on average, yielding an overall voxel size of 1.2 × 1.0 × 1.0 mm. From the 1,715 baseline ADNI scans, the volumes of 34 bilateral cortical brain regions, 68 structures in total, were calculated using a model- and surface-based automated image segmentation procedure, incorporated in the FreeSurfer Package (v.6.0 [60]). Segmentation in Freesurfer was performed by rigid-body registration and nonlinear normalization of images to a probabilistic brain atlas. In the segmentation process, each voxel of the MRI volumes was labeled automatically as a corresponding brain region based on 2 different cortex parcellation guides [61,62], subdividing the brain into 68 and 191 regions, respectively.

#### PPMI data preparation

Patients were selected if their PD diagnosis was <2 years old at baseline and if follow-up data were available for ≥48 months (5–10 time points), resulting in a total of 362 patients. For these 362 patients, a set of 9 motor and non-motor symptoms were selected for clustering:

- TD: tremor dominance
- PIGD: postural instability and gait disturbance
- UPDRS1: Unified Parkinson Disease Rating Scale, part 1: mentation, behavior, and mood
- UPDRS2: Unified Parkinson Disease Rating Scale, part 2: activities of daily living
- UPDRS3: Unified Parkinson Disease Rating Scale, part 3: motor examination
- UPDRS: Unified Parkinson Disease Rating Scale (UPDRS1 + UPDRS2 + UPDRS3)
- RBD: REM sleep behavior disorder
- ESS: Epworth Sleepiness Scale
- SCOPA-AUT: Scales for Outcomes in Parkinson Disease: Assessment of Autonomic Dysfunction

All scores were normalized relative to baseline by (i) subtracting the baseline mean across all patients and (ii) dividing by the baseline standard deviation across all patients.

For some assessments, fewer time points were available. These were treated as missing values.

#### Benchmark datasets for multivariate time-series classification

Because no benchmark datasets exist for multivariate time series clustering, we collected a number of benchmark datasets for multivariate time-series classification [41,42]. Because currently, VaDER still only works with equal-length time series (see also Section Discussion and conclusions), we pre-processed all samples to equal-length time series by linear interpolation between start and end point. Following [63, 64], we chose constant lengths of $\left\lceil {T_{\mathrm{max}}/ \left\lceil {\frac{T_{\mathrm{max}}}{25}}\right\rceil }\right\rceil$, where *T*_max_ is the maximum length of the lengths of the samples in a given dataset.

Moreover, all resulting time series were standardized to zero mean and unit variance.

### VaDER

The VaDER model is extensively described in the Results section. This section describes how VaDER was trained.

#### Pre-training

Similar to Jiang et al. [23], we pre-train VaDER by disregarding the latent loss during the first epochs, essentially fitting a non-variational LSTM autoencoder to the data. We then fit a Gaussian mixture distribution in the latent space of this autoencoder and use its parameters to initialize the final variational training of VaDER.

#### Hyperparameter optimization and choice of number of clusters

We used prediction strength [43] to select suitable values for VaDER’s hyperparameters. These comprise the following:

- number of layers (for both ADNI and PPMI: {1, 2})
- number of nodes per hidden layer (for ADNI: {2^0^, 2^1^, 2^2^, 2^3^, 2^4^, 2^5^, 2^6^}; for PPMI: {2^0^, 2^1^, 2^2^, 2^3^, 2^4^, 2^5^, 2^6^, 2^7^})
- learning rate (for both ADNI and PPMI: {10^−4^, 10^−3^, 10^−2^, 10^−1^})
- mini-batch size (for both ADNI and PPMI: {2^4^, 2^5^, 2^6^, 2^7^})

Hyperparameter optimization was performed via a random grid search (i.e., by randomly sampling a predefined hyperparameter grid) with repeated cross-validation (*n* = 20), using the reconstruction loss as objective. This was done during the pre-training phase of VaDER.

After hyperparameter optimization we trained VaDER models for different numbers of clusters *k* ∈ {2…15}. For each *k*, prediction strength was computed by 2-fold cross-validation [43]: for a given training and test dataset:

1. Train VaDER on the training data (the training data model).
2. Assign clusters to the test data using the training data model.
3. Train VaDER on the test data (the test data model).
4. Assign clusters to the test data using the test data model.
5. Compare the resulting 2 clusterings: for each cluster of the test data model, compute the fraction of pairs of samples in that cluster that are also assigned to the same cluster by the training data model. Prediction strength is defined as the minimum proportion across all clusters of the test data model [43].

Prediction strength was then compared to an empirical null distribution of that measure. The null distribution of the prediction strength was computed by randomly permuting the predicted cluster labels 10^3^ times, then recomputing the prediction strength, and eventually taking the average of the 10^3^ prediction strength values. Doing this for all 20 repeats resulted in 20 values for the eventual null distribution, which were then compared to 20 actual prediction strength values (similarly, 1 for each repeat) by a paired Wilcoxon rank-sum test.

### Simulation experiments

#### Overview of data-generating process

To better understand the performance of VaDER we conducted an extensive simulation study: we simulated multivariate (short) time series via VAR processes [65] because (i) they can model the auto-correlation between time points, (ii) they can model the cross-correlation between variables, and (iii) given a VAR, one can generate random trajectories from that VAR.

We used mixtures of VAR processes to simulate multivariate time-series data of the same dimensions as the ADNI data: 4 variables measured over 8 time points. Given a clusterability factor λ, we generated trajectories as follows:

1. Sample coefficient matrices for 3 VAR(8) processes, by randomly sampling the individual entries of each 4 × 4 matrix from the uniform distribution $\mathcal {U}(-0.1, 0.1)$. Multiply each of the matrix entries by λ.
2. Randomly sample 3 additional 4 × 4 matrices from $\mathcal {U}(-0.1, 0.1)$ and multiply each by its own transpose. Let each of the results correspond to the variance-covariance matrix of 1 of the 3 VAR(8) processes.
3. Repeat 10^3^ times:
  1. Randomly select 1 of the 3 VAR(8) processes (with equal probability).
  2. Generate a random trajectory from the selected VAR(8) process.

The above generates 1 set of random data. Given a value of λ, the entire sampling process was repeated 100 times, and each of the 100 datasets was clustered using both VaDER and hierarchical clustering.

For computational reasons, hyper-parameters for VaDER were fixed and not further optimized during our simulation (10^2^ epochs of both pre-training and training, learning rate: 10^−4^, 2 hidden layers: [36, 4], batch size: 64).

#### Comparison against hierarchical clustering

We compared VaDER against a conventional hierarchical clustering (complete linkage), in which we flatten the *N* × *M* data matrices of each patient into vectors. We considered 3 distance measures for these vectors:

- Pearson correlation
- Euclidean distance
- STS distance [40], a distance measure specifically developed for univariate short time series. The STS distance relies on the difference between adjacent time points. Here we first computed the STS distance for each of the different clinical outcome measures and then summed these up to arrive at an aggregated STS distance across the *M* clinical measures.

Additionally, we compared VaDER against hierarchical clustering using 2 distance measures specifically designed for multivariate time series:

- MD-DTW [38]
- Fast GAK [39]

Given that VaDER is non-deterministic, we ran 100 replicates for each (simulated/benchmark) dataset and determined the consensus clustering by hierarchically clustering a consensus matrix listing, for each pair of samples, how often these 2 samples were clustered together across the 100 replicates.

#### Simulating missing data

To test the ability of VaDER to deal with missing data we performed a separate set of simulations: Let *L* be the number of patients in our dataset and ${\bf x}^l \in \mathbb {R}^{N \times M}$ a single patient trajectory (*l* ∈ 1…*L*), where *N* is the number of time points and *M* is the number of measured features. MCAR were simulated by an individual entry ${\bf x}_{ij}^l$ to missing with probability θ.

MNAR was simulated by letting the probability of a missing value for entry ${\bf x}_{ij}^l$ depend on time. More specifically, each individual entry ${\bf x}_{ij}^l$ was set to missing with probability $1/(1 + e^{i_0 - i / k})$, where $i_0 = (1 + N)/2$ and *k* was varied to result in different overall missingness fractions θ.

To compare VADER’s implicit imputation with pre-imputation, missing values generated using the above approach were additionally imputed using mean substitution: each missing value was substituted with the average conditioned on the relevant time point and variable.

Given that VaDER is non-deterministic, we ran 20 replicates for each (simulated/benchmark) dataset and determined the consensus clustering by hierarchically clustering a consensus matrix listing, for each pair of samples, how often these 2 samples were clustered together across the 20 replicates. Confidence intervals were determined by repeating the aforementioned procedure 100 times (simulation experiments) or 5 times (benchmark experiments) with newly generated missingness patterns (simulation/benchmark experiments) and/or data (simulation experiments).

#### Estimating clustering performance

For the simulation and benchmark datasets, the number of clusters is a priori known. Hence, an intuitive measure of comparing the performance between the different algorithms is cluster purity [37]. Cluster purity can be interpreted as the fraction of correctly clustered samples and is calculated as follows:

1. For each cluster, count the number of samples from the majority class in that cluster.
2. Sum the above counts.
3. Divide by the total number of samples.

For the ADNI and PPMI data, the number of clusters is not a priori known. Hence, performance was recorded using the adjusted Rand index [66, 67] for different values of λ in the interval [0.001, 0.25]. For λ ⪆ 0.25, generating coefficient matrices that lead to stable VARs becomes very difficult.

### Post hoc analysis of patient clusters

We collected a wide range of additional variables from ADNI and PPMI and assessed the association of the identified patient subgroups with a given variable by multinomial logistic regression. For any baseline variable *x*, we first fitted the following full model:

(6) \begin{equation*}
\textrm{subgroup} \sim x + \textrm{confounders} .
\end{equation*}

Each of these full models was then compared to a null model:

(7) \begin{equation*}
\textrm{subgroup} \sim \textrm{confounders}
\end{equation*}

by means of a likelihood ratio test.

For any longitudinal variable *x* measured at time points *t*, we first fitted the following multinomial logistic regression model:

(8) \begin{equation*}
\textrm{subgroup} \sim x + t + x \ast t + \textrm{confounders} .
\end{equation*}

We tested this model against the null model:

(9) \begin{equation*}
\textrm{subgroup} \sim \textrm{confounders}
\end{equation*}

by performing a likelihood ratio test and applying a false discovery rate correction for multiple testing. If the above test was found to be significant (*q* < 0.05), we tested the effects of the individual terms *x* *t, *x*, and *t* against the same null model above.

Confounders considered were age, education, and sex but were only included when univariate results were significantly associated with subgroup. For ADNI, this was only age (*P* = 0.0029, ANOVA *F*-test). For PPMI, this was only sex (*P* = 0.0017, χ^2^test).

In the post hoc analysis, only complete cases were included; i.e., patients with missing values were ignored.

## Availability of Supporting Source Code and Requirements

A complete implementation of VaDER in Python/Tensorflow: https://github.com/johanndejong/VaDER.

An R-package for streamlining the processing of PPMI data: https://github.com/patzaw/PPMI-R-package-generator.

Other code used for generating results presented in this article: https://github.com/johanndejong/VaDER_supporting_code.

Snapshots of all the above code and other supporting data are also available in the GigaScience database, GigaDB [68].

## Additional Files

**Supplementary information**: Supplementary Methods and Results are available via the additional file associated with this article.

Supplementary Figure S1 Multivariate short time series data simulated using vector autoregressive processes, for 4 variables, 8 time points and 3 clusters, and different levels of the similarity parameter λ.

Supplementary Figure S2 ADNI: prediction strength of VaDER for each *k* (blue) and the corresponding permutation-based null distribution.

Supplementary Figure S3 ADNI: associations of the VaDER clustering with a wide range of other baseline data available from ADNI.

Supplementary Figure S4 ADNI: associations of the VaDER clustering with a wide range of other baseline data available from ADNI.

Supplementary Figure S5 ADNI: associations of the VaDER clustering with a wide range of other baseline data available from ADNI.

Supplementary Figure S6 ADNI: associations of the VaDER clustering with a wide range of other baseline data available from ADNI.

Supplementary Figure S7 ADNI: associations of the VaDER clustering with a wide range of other baseline data available from ADNI.

Supplementary Figure S8 ADNI: associations of the VaDER clustering with a wide range of other longitudinal data available from ADNI.

Supplementary Figure S9 PPMI: prediction strength of VaDER for each *k* (blue) and the corresponding permutation-based null distribution.

Supplementary Figure S10 PPMI: associations of the VaDER clustering with a wide range of other baseline data available from PPMI.

Supplementary Figure S11 PPMI: associations of the VaDER clustering with a wide range of other baseline data available from PPMI.

Supplementary Figure S12 PPMI: associations of the VaDER clustering with a wide range of other baseline data available from PPMI.

Supplementary Figure S13 PPMI: associations of the VaDER clustering with a wide range of other longitudinal data available from PPMI.

Supplementary Figure S14 PPMI: associations of the VaDER clustering with a wide range of other longitudinal data available from PPMI.

Supplementary Figure S15 PPMI: associations of the VaDER clustering with a wide range of other longitudinal data available from PPMI.

giz134_GIGA-D-19-00209_Original_Submission

giz134_GIGA-D-19-00209_Revision_1

giz134_Response_to_Reviewer_Comments_Original_Submission

giz134_Reviewer_1_Report_Original_Submission Alexandr Kalinin -- 7/26/2019 Reviewed

giz134_Reviewer_1_Report_Revision_1 Alexandr Kalinin -- 10/8/2019 Reviewed

giz134_Reviewer_2_Report_Original_Submission Karsten Borgwardt -- 8/14/2019 Reviewed

giz134_Supplemental_Files

## Abbreviations

AD: Alzheimer disease; ADAS-13: Alzheimer Disease Assessment Scale; ADNI: Alzheimer’s Disease Neuroimaging Initiative; ANOVA: analysis of variance; CDRSB: Clinical Dementia Rating Sum of Box Score; ESS: Epworth Sleepiness Scale; FAQ: Functional Activities Questionnaire; GAK: Global Alignment Kernels; LSTM: long short-term memory; MAR: missing at random; MCAR: missing completely at random; MD-DTW: multidimensional dynamic time warping; MMSE: Mini–Mental State Examination; MNAR: missing not at random; MRI: magnetic resonance imaging; PD: Parkinson disease; PIGD: postural instability and gait disturbance; PPMI: Parkinson’s Progression Markers Initiative; RBD: REM sleep behavior disorder; SCOPA: Scales for Outcomes in Parkinson's Disease; STS distance: short-time-series distance; TD: tremor dominance; UPDRS: Unified Parkinson's Disease Rating Scale; UPDRS1: Unified Parkinson's Disease Rating Scale, Part 1; UPDRS2: Unified Parkinson’s Disease Rating Scale, Part 2; UPDRS3: Unified Parkinson’s Disease Rating Scale, Part 3; UCI: University of California Irvine Machine Learning Repository; UEA/UCR: University of East Anglia/University of California, Riverside Time-Series Classification Archive; VaDE: variational deep embedding; VaDER: variational deep embedding with recurrence; VAR: vector autoregression; VCF: variant call format.

## Competing Interests

J.d.J. and H.F. received salaries from UCB Biosciences GmbH. UCB Biosciences GmbH had no influence on the content of this work.

## Funding

The research leading to these results has received partial support from the Innovative Medicines Initiative Joint Undertaking under grant agreement No. 115568, resources of which are composed of financial contribution from the European Union’s Seventh Framework Programme (FP7/2007-2013) and EFPIA companies’ in kind contribution.

Data collection and sharing for this project was funded by ADNI (National Institutes of Health Grant U01 AG024904) and DOD ADNI (Department of Defense award No. W81XWH-12-2-0012). ADNI is funded by the National Institute on Aging, the National Institute of Biomedical Imaging and Bioengineering, and through generous contributions from the following: AbbVie; Alzheimer’s Association; Alzheimer’s Drug Discovery Foundation; Araclon Biotech; BioClinica, Inc.; Biogen; Bristol-Myers Squibb Company; CereSpir, Inc.; Cogstate; Eisai, Inc.; Elan Pharmaceuticals, Inc.; Eli Lilly and Company; EuroImmun; F. Hoffmann-La Roche Ltd and its affiliated company Genentech, Inc.; Fujirebio; GE Healthcare; IXICO Ltd.; Janssen Alzheimer Immunotherapy Research & Development, LLC; Johnson & Johnson Pharmaceutical Research & Development, LLC; Lumosity; Lundbeck; Merck & Co., Inc.; Meso Scale Diagnostics, LLC; NeuroRx Research; Neurotrack Technologies; Novartis Pharmaceuticals Corporation; Pfizer, Inc.; Piramal Imaging; Servier; Takeda Pharmaceutical Company; and Transition Therapeutics. The Canadian Institutes of Health Research is providing funds to support ADNI clinical sites in Canada. Private sector contributions are facilitated by the Foundation for the National Institutes of Health (www.fnih.org). The grantee organization is the Northern California Institute for Research and Education, and the study is coordinated by the Alzheimer’s Therapeutic Research Institute at the University of Southern California. ADNI data are disseminated by the Laboratory for Neuro Imaging at the University of Southern California.

## Authors’ Contributions

Method development: J.d.J., H.F.; implementation and testing: J.d.J.; Data preparation: M.A.E., P.W., R.K., M.S., A.A., P.G.; image analysis: H.V.; supervision: H.F., M.H.A.; definition of research project: H.F.
