# Supplementary material for: Deep learning for clustering of multivariate clinical patient trajectories with missing values
Source: Gigascience. 2019 Nov 15;8(11):giz134. doi: 10.1093/gigascience/giz134 (PMC6857688; doi:10.1093/gigascience/giz134)
Supplement: giz134_GIGA-D-19-00209_Original_Submission [file giz134_giga-d-19-00209_original_submission.pdf]

GigaScience

Deep learning for clustering of multivariate clinical patient trajectories with missing values

--Manuscript Draft--

**Manuscript Number:**	GIGA-D-19-00209
**Full Title:**	Deep learning for clustering of multivariate clinical patient trajectories with missing values
**Article Type:**	Technical Note
**Funding Information:**	
**Abstract:**	Background.<br>Precision medicine requires a stratification of patients by disease presentation that is sufficiently informative to allow for selecting treatments on a per-patient basis. For many diseases, such as neurological disorders, this stratification problem translates into a complex problem of clustering multivariate short time series, because (1) these diseases are multifactorial and not well described by single clinical outcome variables, and (2) disease progression needs to be monitored over time. Clinical datasets often additionally suffer from the presence of many missing values, further complicating any clustering attempts.<br><br>Findings.<br>No standard methods exist for clustering multivariate short time series with many missing values. In this work, we propose a deep learning-based method to address this issue, variational deep embedding with recurrence (VaDER). VaDER extends the variational deep embedding clustering algorithm by (1) incorporating long short term memory networks into a VaDE architecture and (2) further extending VaDE's architecture and loss function to directly deal with missing values via implicit imputation and loss re-weighting. We validated VaDER by accurately recovering clusters from simulated data with known ground truth clustering, while varying the degree of missingness. We then used VaDER to successfully stratify Alzheimer's disease (AD) patients and Parkinson's disease (PD) patients into subgroups characterized by clinically divergent disease progression profiles. Additional analyses demonstrated that these clinical differences reflected known underlying aspects of AD and PD.<br><br>Conclusions.<br>We believe our results show that VaDER can be of great value for future efforts in patient stratification, and multivariate short time series clustering in general.
**Corresponding Author:**	Johann de Jong, Ph.D.<br>UCB Biosciences GmbH<br>LEVERKUSEN, Nordrhein-Westfalen GERMANY
**Corresponding Author Secondary Information:**	
**Corresponding Author's Institution:**	UCB Biosciences GmbH
**Corresponding Author's Secondary Institution:**	
**First Author:**	Johann de Jong, Ph.D.
**First Author Secondary Information:**	
**Order of Authors:**	Johann de Jong, Ph.D.
	Mohammed Asif Emon
	Ping Wu
	Reagon Karki
	Meemansa Sood
	Patrice Godard
	Ashar Ahmad

Powered by Editorial Manager® and ProduXion Manager® from Aries Systems Corporation

	Henri Vrooman
	Martin Hofmann-Apitius
	Holger Froehlich
**Order of Authors Secondary Information:**	
**Additional Information:**	
**Question**	**Response**
Are you submitting this manuscript to a special series or article collection?	No
**Experimental design and statistics**<br><br>Full details of the experimental design and statistical methods used should be given in the Methods section, as detailed in our Minimum Standards Reporting Checklist. Information essential to interpreting the data presented should be made available in the figure legends.<br><br>Have you included all the information requested in your manuscript?	Yes
**Resources**<br><br>A description of all resources used, including antibodies, cell lines, animals and software tools, with enough information to allow them to be uniquely identified, should be included in the Methods section. Authors are strongly encouraged to cite Research Resource Identifiers (RRIDs) for antibodies, model organisms and tools, where possible.<br><br>Have you included the information requested as detailed in our Minimum Standards Reporting Checklist?	Yes
**Availability of data and materials**<br><br>All datasets and code on which the conclusions of the paper rely must be either included in your submission or deposited in publicly available repositories (where available and ethically	Yes

Powered by Editorial Manager® and ProduXion Manager® from Aries Systems Corporation

appropriate), referencing such data using a unique identifier in the references and in the "Availability of Data and Materials" section of your manuscript.<br><br>Have you have met the above requirement as detailed in our Minimum Standards Reporting Checklist?	

Manuscript Click here to download Manuscript manuscript.pdf

OXFORD (GIGA)^n SCIENCE

GigaScience, 2017, 1–12

doi: xx.xxxx/xxxx
Manuscript in Preparation
Paper

PAPER

Deep learning for clustering of multivariate clinical patient trajectories with missing values

Johann de Jong[1,*], Mohammad Asif Emon[2,3], Ping Wu[4], Reagon Karki[2,3], Meemansa Sood [2,3], Patrice Godard[6], Ashar Ahmad[3], Henri Vrooman[5], Martin Hofmann-Apitius[2,3] and Holger Fröhlich[1,3,*]

[1]UCB Biosciences GmbH, 40789 Monheim, Germany and [2]Fraunhofer Institute for Algorithms and Scientific Computing, 53754 Sankt Augustin, Germany and [3]Bonn-Aachen International Center for IT, University of Bonn, 53115 Bonn, Germany and [4]UCB Pharma, Slough SL1 3WE, United Kingdom and [5]Erasmus MC, University Medical Center Rotterdam, Departments of Radiology and Medical Informatics, PO Box 2040 3000 CA Rotterdam, Netherlands and [6]UCB Pharma, 1420 Braine-l'Alleud, Belgium

[*]johann.dejong@ucb.com; holger.froehlich@ucb.com; frohlich@bit.uni-bonn.de

Abstract

Background. Precision medicine requires a stratification of patients by disease presentation that is sufficiently informative to allow for selecting treatments on a per-patient basis. For many diseases, such as neurological disorders, this stratification problem translates into a complex problem of clustering multivariate and relatively short time series, because (1) these diseases are multifactorial and not well described by single clinical outcome variables, and (2) disease progression needs to be monitored over time. Clinical datasets often additionally suffer from the presence of many missing values, further complicating any clustering attempts.
Findings. No standard methods exist for clustering multivariate short time series with many missing values. In this work, we propose a deep learning-based method to address this issue, variational deep embedding with recurrence (VaDER). VaDER relies on a Gaussian mixture of variational autoencoder framework, which is further extended by (1) incorporating long short term memory units and (2) defining an appropriate approach to directly deal with missing values via implicit imputation and loss re-weighting. We validated VaDER by accurately recovering clusters from simulated data with known ground truth clustering, while varying the degree of missingness. We then used VaDER to successfully stratify Alzheimer's disease (AD) patients and Parkinson's disease (PD) patients into subgroups characterized by clinically divergent disease progression profiles. Additional analyses demonstrated that these clinical differences reflected known underlying aspects of AD and PD.
Conclusions. We believe our results show that VaDER can be of great value for future efforts in patient stratification, and multivariate short time series clustering in general.

Key words: Patient stratification; deep learning; multivariate short time series; multivariate longitudinal data; clustering

Findings

Background

In precision medicine, patients are stratified based on their disease subtype, risk, prognosis, or treatment response using specialized diagnostic tests. An important question in precision

Compiled on: June 6, 2019.
Draft manuscript prepared by the author.

medicine, is how to appropriately model disease progression and accordingly decide for the right type and time point of therapy for an individual. However, the progression of many diseases, such as neurological disorders, cardiovascular diseases, diabetes, obesity [1, 2, 3, 4, 5], is highly multifaceted and not well described by one clinical outcome measure alone. Classical univariate clustering methods are likely to miss the inherent complexity of diseases that demonstrate a highly multifaceted clinical phenotype. Accordingly, stratification of patients by disease progression translates into the challenging question of how to identify a clustering of a multivariate time series.

Clustering is a fundamental and generally well investigated problem in machine learning and statistics. Its goal is to segment samples into groups (clusters), such that there is a higher degree of similarity between samples of the same cluster than between samples of different clusters. Following [6], algorithms to solve clustering problems may be put into three main categories, (1) combinatorial algorithms, (2) mixture modeling and (3) mode seeking. Within each of these three categories, a wide range of methods is available for a great diversity of clustering problems. Combinatorial algorithms do not assume any underlying probability model, but work with the data directly. Examples are K-means clustering, spectral clustering [7] and hierarchical clustering [8]. Mixture models assume that the data can be described by some probabilistic model. An example is Gaussian mixture model clustering. Finally, in mode seeking one tries to directly estimate modes of the underlying multi-modal probability density. An important example here is the mean-shift algorithm [9].

For the clustering of multivariate time series data, a few techniques have been developed [10, 11, 12, 13, 14]. However, these approaches generally rely on time series of far greater length than available in most longitudinal clinical datasets. Moreover, these methods are not suited for the large numbers of missing values often found in clinical data.

Missing values in clinical data can occur due to different reasons: (1) patients drop out of a study, e.g. due to worsening of symptoms; (2) a certain diagnostic test is not taken at a particular visit (e.g. due to lack of patient agreement), potentially resulting into missing information for entire variable groups; (3) unclear further reasons, e.g. time constraints, data quality issues, etc. From a statistical point of view, these reasons manifest into different mechanisms of missing data [15, 16]:

i. Missing completely at random (MCAR): The probability of missing information is neither related to either the specific value which is supposed to be obtained, nor to other observed data. Hence, entire patient records could be skipped without introducing any bias. However, this type of missing data mechanism is probably rare in clinical studies.
ii. Missing at random (MAR): The probability of missing information depends on other observed data, but is not related to the specific missing value that is expected to be obtained. An example would be patient drop out due the to worsening of certain symptoms, which are at the same time recorded during the study.
iii. Missing not at random (MNAR): any reason for missing data, which is neither MCAR nor MAR. MNAR is problematic, because the only way to obtain unbiased estimates is to model missing data.

Multiple imputation methods have been proposed to deal with missing data in longitudinal patient data [16]. However, any imputation method will result in certain errors, and if imputation and clustering are done separately, these errors will propagate through to the following clustering procedure.

To address the problem of clustering multivariate and relatively short time series data with many missing values, in this paper we propose an approach that uses techniques from deep learning. Autoencoder networks have been highly successful in learning latent representations of data, e.g. [17, 18, 19, 20]. Specifically for clustering, autoencoders can be used to first learn a latent representation of a multivariate distribution, and then independently find clusters [21]. More recently, some authors have suggested to simultaneously learn latent representations and cluster assignments. Interesting examples are deep embedded clustering (DEC) [22] and variational deep embedding (VaDE) [23].

Here, we present a new method for clustering multivariate short time series with potentially many missing values, VaDER (variational deep embedding with recurrence). VaDER is based on VaDE [23], a clustering algorithm based on variational autoencoder principles, with a latent representation forced towards a multivariate Gaussian mixture distribution. VaDER extends VaDE by (1) integrating two LSTM networks [24] into a VaDE architecture, to allow for the analysis of multivariate short time series, and (2) adopting an approach of implicit imputation and loss re-weighting to account for the typically high degree of missingness in clinical data.

After a validation of VaDER via a simulation study, we applied the method to the problem of patient stratification in Alzheimer's disease (AD) and Parkinson's disease (PD), using data from the Alzheimer's Disease Neuroimaging Initiative (ADNI) [25] and the Parkinson's Progression Markers Initiative (PPMI) [26] respectively. Alzheimer's and Parkinson's disease are multifactorial and highly heterogeneous diseases, both in clinical and biological presentation, as well as in progression [27, 28, 29, 30]. For example, PD is characterized by motor symptoms, behavioral changes (e.g. sleeping disorders) as well as cognitive impairment [1]. Cognitive impairment, one of the hallmarks of AD, is not straightforward to assess, since cognition itself is highly multifaceted, and described by e.g. orientation, speech and memory. Consequently, in the field of AD, a wide range of tests have been developed to assess different aspects of cognition.

This heterogeneity presents one of the major challenges in understanding these diseases and developing new treatments. As such, better clustering (stratification) of patients by disease presentation could be of great help in improving disease management and designing better clinical trials that specifically focus on treating patients that are rapidly progressing.

Our analyses of the ADNI and PPMI data show that VaDER is highly effective at disentangling multivariate patient trajectories into clinically meaningful patient subgroups.

Results

Variational deep embedding (VaDE)

The basis for our proposed variational deep embedding with recurrence (VaDER) method is variational deep embedding (VaDE) [23], a variational autoencoding clustering algorithm with a multivariate Gaussian mixture prior. In variational autoencoding algorithms, the training objective is to optimize the variational lower bound on the marginal likelihood of a data point $\mathbf{x}$ [31]:

$$\mathcal{L}(\mathbf{x}) = \mathbb{E}_{q(\mathbf{z}|\mathbf{x})}[\log(p(\mathbf{x}|\mathbf{z}))] - D_{KL}(q(\mathbf{z}|\mathbf{x})||p(\mathbf{z})) \quad (1)$$

This lower bound can be seen as composed of two parts. The first term corresponds to the likelihood of seeing $\mathbf{x}$ given a la-

1 https://www.ninds.nih.gov/Disorders/All-Disorders/Parkinsons-Disease-Information-Page

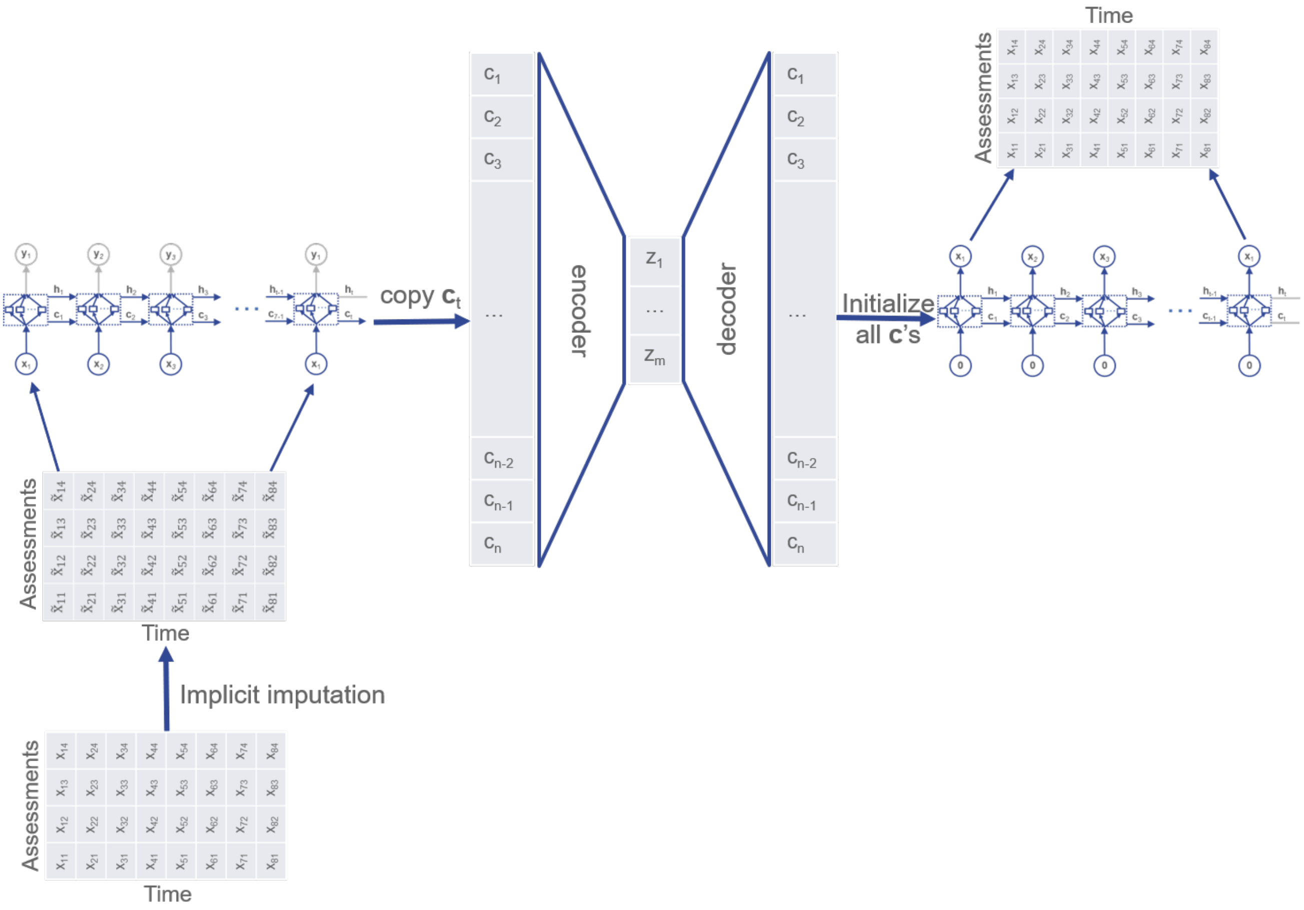

Figure 1. VaDER architecture

tent representation $\mathbf{z}$. Its negative is often called the *reconstruction loss*, and it forces the algorithm to learn good reconstructions of its input data. The negative of the second term is often called the *latent loss*. It is the Kullback-Leibler divergence of the prior $p(\mathbf{z})$ to the variational posterior $q(\mathbf{z}|\mathbf{x})$, and it regularizes the latent representation $\mathbf{z}$ to lie on a manifold specified by the prior $p(\mathbf{z})$.

In VaDE, this prior is a multivariate Gaussian mixture. Accordingly including a parameter for choosing a cluster c, the variational lower bound can then be written as follows:

$$\mathcal{L}(\mathbf{x}) = \mathbb{E}_{q(\mathbf{z},c|\mathbf{x})}[\log(p(\mathbf{x}|\mathbf{z}))] - D_{KL}(q(\mathbf{z},c|\mathbf{x})||p(\mathbf{z},c)) \quad (2)$$

By forcing the latent representation $\mathbf{z}$ towards a multivariate Gaussian mixture distribution, VaDE has the ability to simultaneously learn latent representations and cluster assignments of its input data. For more details on variational autoencoders and VaDE, we refer the reader to [32, 31, 23].

Variational deep embedding with recurrence (VaDER)

VaDER builds upon and extends VaDE in a number of ways, mainly related to (1) modeling multivariate short time series, and (2) dealing with missing values.

To model the auto- and cross-correlations in multivariate short time series data, we integrate peephole LSTM networks [24, 33] into the VaDE architecture. The resulting architecture is visualized in Figure 1.

To deal with missing values, we directly integrate imputation into model training. As outlined in Section Background, separating imputation from clustering can potentially introduce bias. To avoid this bias, we here propose an implicit imputation scheme, which is performed within VaDER training. Our approach bears some similarity to other approaches [34, 35]. However, in contrast to [34], VaDER uses missingness indicators for implicit imputation as an integral part of neural network training. Additionally, in contrast to [35], our method is also suited for MNAR data, which are often encountered in clinical datasets.

We first define a weighted reconstruction loss on feature and sample level: Imputed values are weighted to 0, non-imputed values are weighted to 1. To retain the balance with the latent loss, the resulting reconstruction loss is re-scaled to match the original dimensions of the data. More formally, for a mean squared reconstruction loss, let L be the number of samples in our dataset, $\mathbf{x}^l$ a single input sample, and $\hat{\mathbf{x}}^l$ its corresponding reconstructed output ($l \in 1 \ldots L$). $\mathbf{x}^l$ and $\hat{\mathbf{x}}^l$ are matrices $\in \mathbb{R}^{N \times M}$, where N is the number of time points and M is the number of clinical outcome measures (e.g. cognitive assessments) for a particular patient. Then the unweighted mean reconstruction loss is:

$$\frac{1}{L}\sum_{l=1}^{L}\sum_{i=1}^{N}\sum_{j=1}^{M}(x_{ij}^l - \hat{x}_{ij}^l)^2 \quad (3)$$

Now, let $A := \{x_{ij}^l | x_{ij}^l \text{is missing}\}$, $\mathbf{1}_A(.)$ be the indicator function on set A, and $|A|$ be the cardinality of A. Then, the weighted mean squared reconstruction loss is:

$$\frac{NM}{|A|}\sum_{l=1}^{L}\sum_{i=1}^{N}\sum_{j=1}^{M}\mathbf{1}_A(x_{ij}^l)(x_{ij}^l - \hat{x}_{ij}^l)^2 \quad (4)$$

In addition to the weighted reconstruction loss, we adopt an implicit imputation scheme, where imputed values are learned as an integral part of model training. More specifically, Let $\mathbf{x}^l$, N, M, x^l_{ij}, A and $\mathbf{1}_A(.)$ be defined as above. Also assume that all x^l_{ij} for which $\mathbf{1}_A(x^l_{ij}) = 1$, are initially imputed with arbitrary finite values. Then we add one additional layer before the input LSTM (Figure 1) as follows:

$$\tilde{x}^l_{ij} = x^l_{ij} \times (1 - \mathbf{1}_A(x^l_{ij})) + b_{ij} \times \mathbf{1}_A(x^l_{ij}) \quad (5)$$

Here, x^l_{ij} is the actual observed (or missing) value of sample l at time points i and assessment j, and $\tilde{x}^l_{ij}$ serves as input to the LSTM. In other words, if x^l_{ij} is missing, then it is replaced by b_{ij} in $\tilde{\mathbf{x}}$. Parameters b_{ij} are trained as an integral part of VaDER using stochastic gradient descent, and can be considered (time, assessment)-specific expected values. Note that (1) the initial arbitrary imputation does not influence the eventual clustering, and (2) the implicitly imputed values are weighted to 0 in the reconstruction loss.

VaDER achieves high accuracy on simulated data

To our knowledge, no benchmark datasets exist for multivariate short time series clustering. Hence, we simulated data for validating VaDER instead. A natural framework to this end is the vector autoregressive (VAR) model, because (1) it can express serial correlation between time points, (2) it can express cross-correlation between variables, and (3) given a fully parameterized VAR process, one can simulate random trajectories from that VAR process.

More specifically, to generate clusters of multivariate short time series, we simulated from VAR process mixtures, for different values of a clusterability parameter λ. The clusterability parameter λ influences how easily separable the simulated clusters are (see Section Simulation experiments). Sample data is provided in the Supplemental Material.

As shown in Figure 2a, VaDER was able to highly accurately recover the simulated clusters, achieving an adjusted rand index of ~0.9 for $\lambda \approx 0.05$, and converging to 1.0 for larger λ. In contrast, hierarchical clustering performed substantially worse and even close to random for lower λ. This highlights that data generated from VAR process mixtures is not trivially clustered.

We used the same VAR framework to assess how varying degrees of missing values affect the performance of VaDER. Both missing values completely at random (MCAR) and missing values not at random (MNAR) were simulated as described in Section Methods. In the MCAR simulation, missing values were uniformly distributed across time and clinical outcome measures. In the MNAR simulation, the expected degree of missing values depended on time (see Section Methods). For varying clusterability levels λ, it can be seen that VaDER seems remarkably robust against high degrees of both MCAR and MNAR (Figures 2b and 2c).

Application 1: VaDER identifies clinically diverse AD patient subgroups

We applied VaDER to clinical data for identifying meaningful patient subgroups. From the Alzheimer's Disease Neuroimaging Initiative (ADNI) [25], we collected data from 689 patients that were at some point diagnosed with dementia during the course of this study. Four different cognitive assessment scores were available at 8 different visits: ADAS13, CDRSB, MMSE and FAQ. We pre-processed the data as described in Section ADNI data preparation. Overall, the fraction of missing values was ~ 43%. We used VaDER to cluster patients by disease progression as measured using these cognitive assessments.

Hyperparameter optimization was performed by random grid search as described in Section Methods. For each number of clusters $k \in \{2 \ldots 15\}$, the prediction strength [37] of the corresponding optimal model was compared to a null distribution (see Section Hyperparameter optimization and choice of number of clusters), which is shown in the Supplemental Materials. For our subsequent analyses, we chose k = 3, which demonstrated relatively high prediction strength, significantly different from the null distribution, while still allowing VaDER to uncover potentially interesting interactions between the time series.

The resulting cluster mean trajectories are shown in Figure 3, and demonstrate that (1) VaDER effectively clusters the data into patient subgroups showing divergent disease progression, and (2) VaDER is able to find interactions between the different cognitive assessments, which would be principally difficult to distill from univariate analyses of the assessments. For example, the patients in cluster 1 are the most severely progressing patients when assessed using ADAS13, CDRSB and MMSE. However, the FAQ assessment (instrumental activities of daily living) does not distinguish between these severely progressing patients and the more moderately progressing patients in cluster 1.

In addition to cognitive assessment measurements, ADNI presents a wealth of data on brain volume and various AD markers that we did not use for clustering. In this data, we identified numerous statistically significant associations with our patient subgroups. For example, the clusters strongly associated with time-to-dementia diagnosis relative to baseline, with cluster 2 showing generally the shortest time, and cluster 0 the longest. The relatively mildly progressing patients in cluster 0 also demonstrated on average a larger whole brain volume at baseline, which moreover declined less steeply over time, compared to more severely progressing patients. Especially the middle temporal gyri and fusiform gyri were larger (and shrinking more slowly over time), whereas the ventricles were smaller (and expanding more slowly over time). Indeed, atrophy of the middle temporal gyri and fusiform gyri, as well as ventricular enlargment, have been associated with Alzheimer's disease progression [38, 39]. As another example, the more severely progressing patients (clusters 1 and especially 2), demonstrated lower cerebral glucose uptake and lower cerebrospinal Abeta42 levels, again confirming the literature [40, 41] (see Section Methods and Supplemental Material). These observations demonstrate that the clinical differences between our patient subgroups reflect known Alzheimer's disease aspects.

Application 2: VaDER identifies clinically diverse PD patient subgroups

We additionally applied VaDER to data from the Parkinson's Progression Markers Initiative (PPMI) [26]. From PPMI, we collected data from 362 de novo PD patients that had been diagnosed within a time period of two years before study onset and were initially not been treated. 9 variables on several motor and non-motor symptoms (UPDRS total, UPDRS1-3, TD, PIGD, RBD, ESS, SCOPA-AUT) measured at either 5 or 10 time points were available. The data was pre-processed as described in Section PPMI data preparation. Overall, the fraction of missingness values was ~ 17% (or ~ 31%, when including time points entirely missing for some assessments). We again used VaDER to cluster patients according to disease progression as measured by these assessments.

Hyperparameter optimization and selection of the number of clusters was performed in the same way as for ADNI, and we decided on $k = 3$ patient subgroups accordingly. The resulting cluster mean trajectories are shown in Figure 4. These again illustrate that (1) VaDER effectively clusters the data into clini-

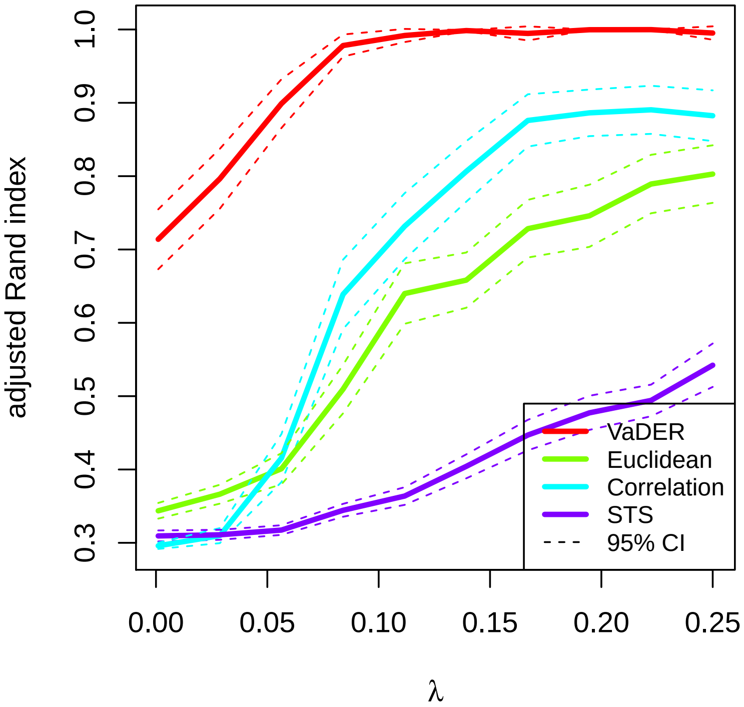

(a) Adjusted rand index for clustering of simulated data as a function of the clusterability parameter λ, with higher λ implying a higher degree of similarity between profiles in the same cluster. Results are shown for VaDER as well as hierarchical clustering using three different distance measures, (1) Euclidean distance, (2) Pearson correlation and (3) Short time series (STS) distance [36]

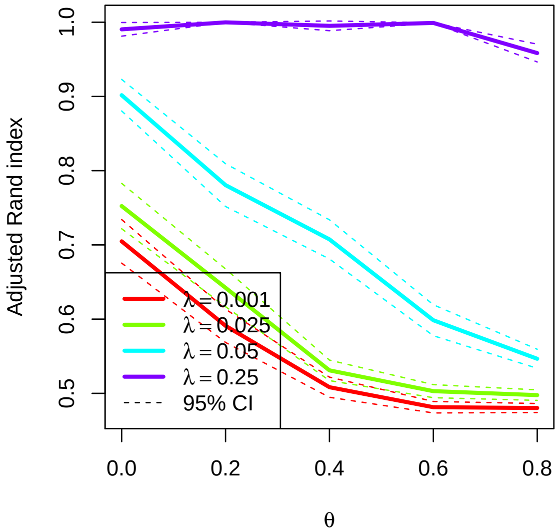

(b) Adjusted rand index as a function of the fraction θ of values missing completely at random (MCAR), for various levels of the clusterability parameter λ.

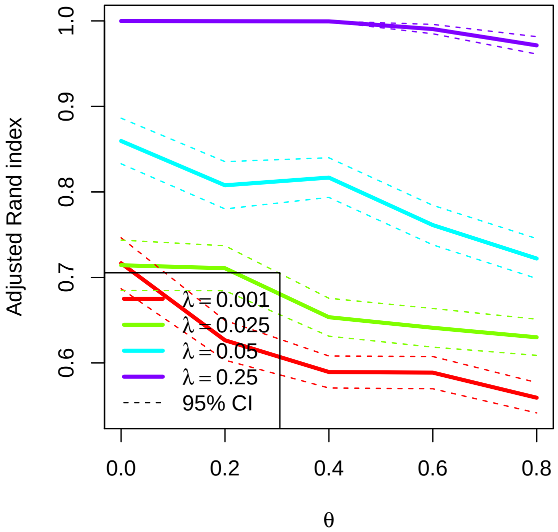

(c) Adjusted rand index as a function of the fraction θ of values missing not at random (MNAR) (see Section Methods for details), for various levels of the clusterability parameter λ.

Figure 2. VaDER performance on simulated data, with varying degrees of clusterability and missingness.

cally divergent patient subgroups, and (2) VaDER is able to find interactions between the assessments that would principally be difficult to find based on univariate analyses alone. For example, cluster 0 represents patients with a moderate progression in terms of mental impairment, behavior, and mood (UPDRS1) and autonomic dysfunction (SCOPA). However, these patients remain relatively stable, or even improve, on many other assessments, such as tremor dominance (TD), the self-reported ability to perform activities of daily life (UPDRS2) and motor symptoms evaluation (UPDRS3).

Similar to ADNI, PPMI presents a wealth of additional data on brain volume and various PD markers that were not used for clustering. Aligning these data with our PD patient subgroups, we found numerous statistically significant associations that

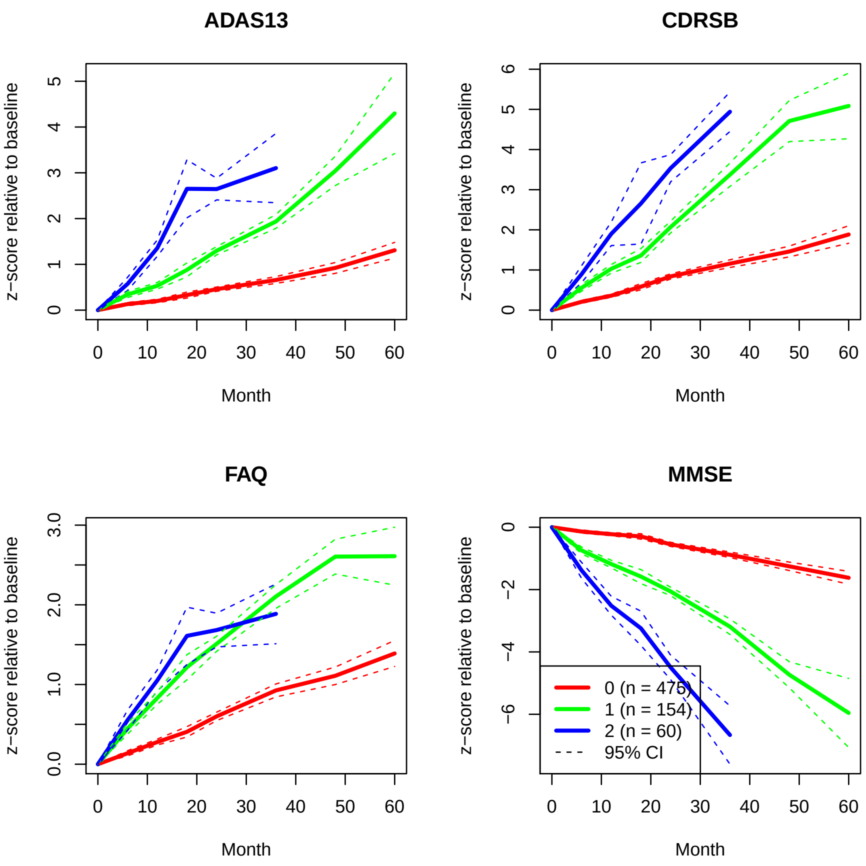

Figure 3. Normalized cluster mean trajectories relative to baseline (*x*-axis in months), as identified by VaDER from the ADNI cognitive assessment data.

confirmed existing literature, many related to quality of life and physiological changes to the brain. For example, men were over-represented in cluster 1, and showed the most severe disease progression, confirming the literature on gender differences in PD (e.g. [42]). Moreover, these severely progressing patients showed an expected steeply declining ability to perform activities of daily living (modified Schwab and England score [43]), as well as rapidly developing symptoms of depression (geriatric depression scale [44]), common in PD patients [45]. Additionally, these patients demonstrated physiological differences in the brain when compared to more mildly progressing patients. Examples are the caudate nucleus and putamen brain regions, which were smaller at baseline and during follow-ups in the more severely progressing patients in cluster 1, and from the literature are known to be subject to atrophy in PD [46] (see Section Methods and Supplemental Material). These observations demonstrate that the clinical differences between our patient subgroups reflect known aspects of PD disease progression.

Discussion and conclusions

Identifying subgroups of patients with similar progression patterns can help to better understand the heterogeneity of complex diseases. Together with predictive machine learning methods, this might help to better decide on the right time and type of treatment for an individual patient, as well as to improve the design of clinical studies. However, one of the main challenges is the multifaceted nature of progression in many areas of disease.

In this paper, we proposed VaDER, an extension of VaDE [23] for clustering multivariate short time series with potentially many missing values, a setting that seems generally not well addressed in the literature so far, but nonetheless often encountered in clinical study data. The main extensions compared to VaDE are (1) the use of LSTMs and (2) an architecture and loss function that directly deal with missing values.

We validated VaDER by showing the very high accuracy on clustering simulated data with a known ground truth. We then applied VaDER to data from (1) ADNI and (2) PPMI, resulting in subgroups characterized by clinically highly divergent disease progression profiles. A comparison with other data from ADNI and PPMI, such as brain imaging, motor- and cognitive assessment data, furthermore supported the observed patient subgroups.

VaDER has two main distinctive features. One is that VaDER deals directly with missing values. For clinical research this is crucial, since clinical data often show a very high degree of missing values [47, 48]. The other main distinctive feature is that, as opposed to existing methods [10, 11, 12, 13, 14], VaDER is specifically designed to deal with multivariate and relatively short time series that are typical for (observational) clinical studies. However, it is worthwhile to mention that VaDER is not per se limited to longitudinal clinical study data. Future applications (potentially requiring some adaptations) could e.g. include data originating from electronic health records, multiple wearable sensors, video recordings, or time series gene (co-)expression. Moreover, VaDER could be used as a generative model: given a trained model, it is possible to generate "virtual" patient trajectories.

Altogether, we believe that our results show that VaDER has the potential to enhance future patient stratification efforts, and multivariate short time series clustering in general.

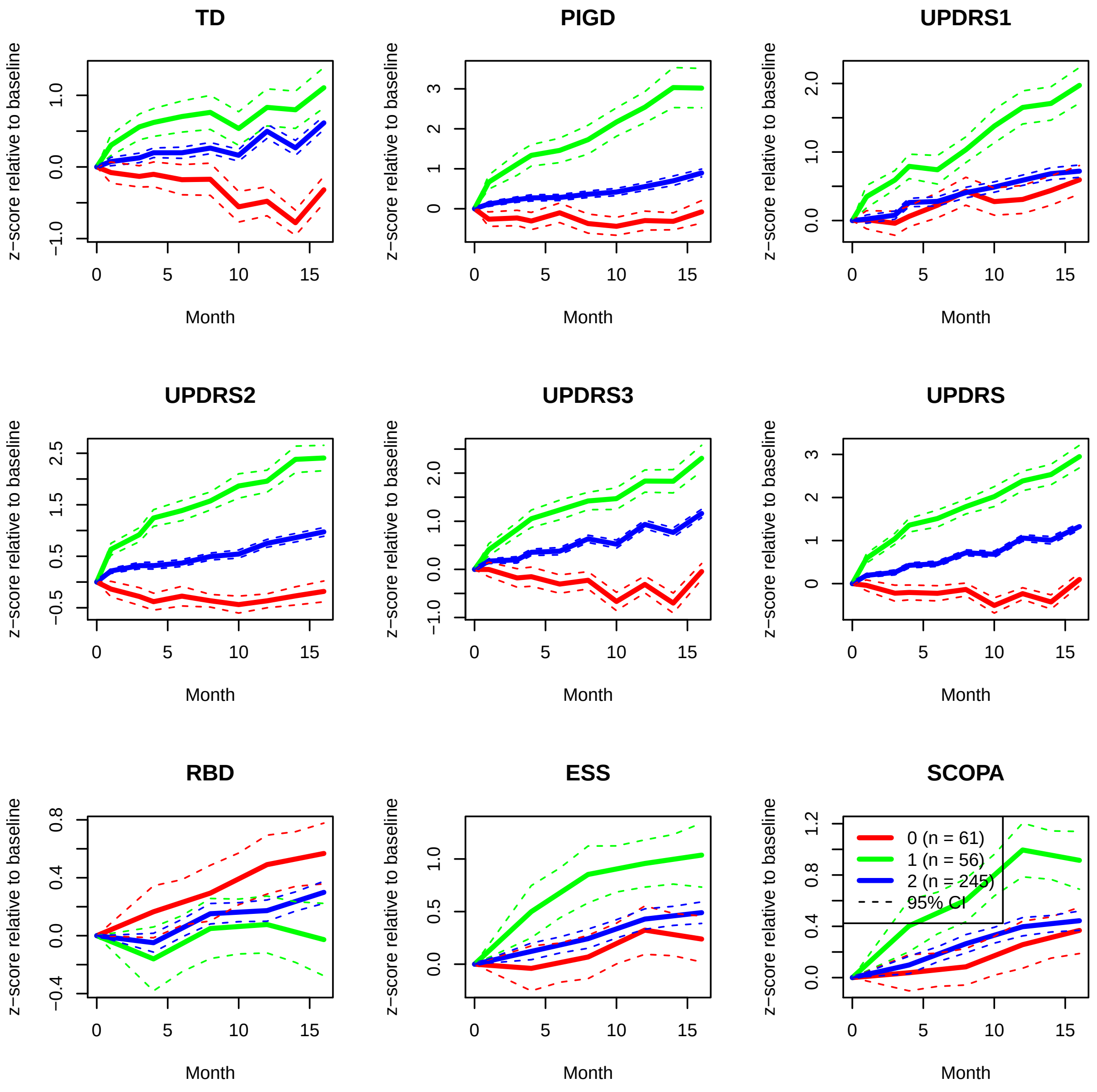

Figure 4. Normalized cluster mean trajectories relative to baseline (*x*-axis in months), as identified by VaDER from the PPMI motor/non-motor score data.

Methods

Data preparation

ADNI data preparation

The ADNIMERGE R-package [49] contains mainly two categories of data, (1) longitudinal and (2) non-longitudinal. These data represent 1737 participants that include healthy controls and patients diagnosed with Alzheimer's Disease (AD). The non-longitudinal features such as demographics and APOE e4 status were measured only once, at baseline. The longitudinal features (i.e. neuroimaging features, cerebrospinal fluid (CSF) biomarkers, cognitive tests and everyday cognition) were recorded over a span of 5 years.

Clinical data. In the current study, we have focused on those participants who were diagnosed with AD at baseline or during one of the follow-up visits. After this filtering step, we had a total of 689 patients. For these 689 patients, four cognitive assessments were selected for clustering:

- ADAS-13: The Alzheimer's disease assessment scale
- CDRSB: The clinical dementia rating sum of box score.
- FAQ: The functional activities questionnaire.
- MMSE: mini-mental state examination

The above assessments were taken at baseline and at 6, 12, 18, 24, 36 48 and 60 months after baseline. For each of the four cognitive assessments, all time points were normalized relative to baseline by (1) subtracting the baseline mean across the 689 patients, and (2) dividing by the baseline standard deviation across the 689 patients.

Imaging data. All available MR scans (T1-weighted scans) from the ADNI database were quantified by an open-source, automated segmentation pipeline at the Erasmus University Medical Center, The Netherlands. The number of slices of the T1w scans varied from 160 to 196 and the in-plane resolution was 256 x 256 on average, yielding an overall voxel-size of 1.2 x 1.0 x 1.0 mm. From the 1715 baseline ADNI scans, the volumes of 34 bilateral cortical brain regions, 68 structures in total, were calculated using a model- and surface-based automated image segmentation procedure, incorporated in the FreeSurfer Package (v.6.0, http://surfer.nmr.mgh.harvard.edu/). Segmentation in Freesurfer was performed by rigid-body registration and nonlinear normalization of images to a probabilistic brain

atlas. In the segmentation process, each voxel of the MRI volumes was labeled automatically as a corresponding brain region based on two different cortex parcellation guides [50, 51], subdividing the brain into 68 and 191 regions respectively.

PPMI data preparation

Patients were selected if their PD diagnosis was less than 2 years old at baseline time, and if follow up data was available for at least 48 months (5 - 10 time points), resulting in a total of 362 patients. For these 362 patients, a set of 9 motor and non-motor symptoms were selected for clustering:

- TD: tremor-dominant
- PIGD: postural instability and gait disturbance.
- UPDRS1: Unified Parkinson's disease rating scale, part 1: mentation, behavior, and mood.
- UPDRS2: Unified Parkinson's disease rating scale, part 2: activities of daily living.
- UPDRS3: Unified Parkinson's disease rating scale, part 3: motor examination.
- UPDRS: Unified Parkinson's disease rating scale (UPDRS1 + UPDRS2 + UPDRS3).
- RBD: REM sleep behavior disorder.
- ESS: Epworth sleepiness scale.
- SCOPA-AUT: Scales for outcomes in Parkinson's disease: assessment of autonomic dysfunction.

All scores were normalized relative to baseline by (1) subtracting the baseline mean across all patients, and (2) dividing by the baseline standard deviation across all patients.

For some assessments, fewer time points were available. These were treated as missing values.

Variational deep embedding with recurrence (VaDER)

The VaDER model is extensively described in Section Results. This section describes how VaDER was trained.

Pre-training

Similar to [23], we pre-train VaDER by disregarding the latent loss during the first epochs, essentially fitting an non-variational LSTM autoencoder to the data. We then fit a Gaussian mixture distribution in the latent space of this autoencoder, and use its parameters to initialize the final variational training of VaDER.

Hyperparameter optimization and choice of number of clusters

We used prediction strength [37] to select suitable values for VaDER's hyperparameters. These comprise:

- number of layers (for both ADNI and PPMI: $\{1, 2\}$)
- number of nodes per hidden layer (for ADNI: $\{2^0, 2^1, 2^2, 2^3, 2^4, 2^5, 2^6\}$; for PPMI: $\{2^0, 2^1, 2^2, 2^3, 2^4, 2^5, 2^6, 2^7\}$)
- learning rate (for both ADNI and PPMI: $\{10^{-4}, 10^{-3}, 10^{-2}, 10^{-1}\}$)
- mini-batch size (for both ADNI and PPMI: $\{2^4, 2^5, 2^6, 2^7\}$)

Hyperparameter optimization was performed via a random grid search (i.e. by randomly sampling a predefined hyperparameter grid) with repeated cross-validation ($n = 20$), using the reconstruction loss as objective. This was done during the pre-training phase of VaDER.

After hyperparameter optimization we trained VaDER models for different numbers of clusters $k \in \{2 \ldots 15\}$. For each k, prediction strength was computed by 2-fold cross-validation [37]: For a given training and test dataset:

i. Train VaDER on the training data. (the training data model)
ii. Assign clusters to the test data using the training data model.
iii. Train VaDER on the test data. (the test data model)
iv. Assign clusters to the test data using the test data model.
v. Compare the resulting two clusterings: For each cluster of the test data model, compute the fraction of pairs of samples in that cluster that are also assigned to the same cluster by the training data model. Prediction strength is defined as the minimum proportion across all clusters of the test data model. [37].

Prediction strength was then compared to an empirical null distribution of that measure. The null distribution of the prediction strength was computed by randomly permuting the predicted cluster labels 10^3 times, then recomputing the prediction strength, and eventually taking the average of the 10^3 prediction strength values. Doing this for all 20 repeats, resulted in 20 values for the eventual null distribution, which were then compared to 20 actual prediction strength values (similarly, one for each repeat).

Simulation experiments

Overview about data generating process

To better understand the performance of VaDER we conducted an extensive simulation study: We simulated multivariate short time series via vector autoregressive (VAR) processes [52], because (1) they can model the auto-correlation between time points, (2) they can model the cross-correlation between variables and (3) given a VAR, one can generate random trajectories from that VAR.

We used mixtures of VAR processes to simulated multivariate time series data of the same dimensions as the ADNI data: 4 variables measured over 8 time points. Given a clusterability factor λ, we generated trajectories as follows:

i. Sample coefficient matrices for 3 VAR(8) processes, by randomly sampling the individual entries of each 4×4 matrix from the uniform distribution $\mathcal{U}(-.1, .1)$. Multiply each of the matrix entries by λ.
ii. Randomly sample 3 additional 4×4 matrices from $\mathcal{U}(-.1, .1)$, and multiply each with its own transpose. Let each of results correspond to the variance-covariance matrix of one of the 3 VAR(8) processes.
iii. Repeat 10^3 times:
   i. Randomly select one of the 3 VAR(8) processes (with equal probability).
   ii. Generate a random trajectory from the selected VAR(8) process.

The above generates one set of random data. Given a value of λ, the entire sampling process was repeated 100 times, and each of the 100 datasets was clustered using both VaDER and hierarchical clustering (with three distance measures). For computational reasons, hyper-parameters for VaDER were fixed and not further optimized during our simulation (10^3 epochs of training, learning rate: 10^{-3}, two hidden layers: [36, 4], $\alpha : 0.2$, batch size: 64).

Comparison against hierarchical clustering

We compared VaDER against a conventional hierarchical clustering (complete linkage), in which we flatten the $N \times M$ data matrices of each patient into vectors. We considered three distance measures for these vectors:

- Pearson correlation
- Euclidean distance
- Short time series (STS) distance [36], a distance measure especially developed for univariate short time series. The STS distance relies on the difference between adjacent time points. Here we first computed the STS distance for each of the different clinical outcome measures, and then summed these up to arrive at an aggregated STS distance across the M clinical measures.

Simulating missing data

To test the ability of VaDER to deal with missing data we performed a separate set of simulations: Let L be the number of patients in our dataset, and $\mathbf{x}^l \in \mathbb{R}^{N \times M}$ a single patient trajectory ($l \in 1 \ldots L$), where N is the number of time points and M is the number of measured features. Missing values completely at random (MCAR) were simulated by an individual entry $\mathbf{x}^l_{ij}$ to missing with probability θ.

Missing not at random (MNAR) was simulated by letting the probability of a missing value for entry $\mathbf{x}^l_{ij}$ depend on time. More specifically, each individual entry $\mathbf{x}^l_{ij}$ was set to missing with probability $\frac{\theta}{1+e^{i_0 - i}}$, where $i_0 = \frac{1+N}{2}$. The above results in a missingness that sigmoidally increases as a function of i, and an expected missingness fraction of θ when aggregated over all time points.

Estimating clustering performance

Performance was recorded using the adjusted rand index [53, 54] for different values of λ in the interval $[0.001, 0.25]$. For $\lambda \gtrapprox 0.25$, generating coefficient matrices that lead to stable VARs becomes very difficult.

Post-hoc analysis of patient clusters

We collected a wide range of additional variables from ADNI and PPMI, and assessed the association of the identified patient subgroups with a given variable by multinomial logistic regression. For any baseline variable x, we first fitted the following full model:

$$\text{subgroup} \sim x + \text{confounders} \tag{6}$$

Each of these full models were then compared to a null model:

$$\text{subgroup} \sim \text{confounders} \tag{7}$$

by means of a likelihood ratio test.

For any longitudinal variable x measured at timepoints t, we first fitted the following multinomial logistic regression model:

$$\text{subgroup} \sim x + t + x * t + \text{confounders} \tag{8}$$

We tested this model against the null model:

$$\text{subgroup} \sim \text{confounders} \tag{9}$$

by performing a likelihood ratio test, and applying an FDR correction for multiple testing. If the above test was found to be significant ($q < 0.05$), we tested the effects of the individual terms $x * t$, x and t against the same null model above.

Confounders considered were age, education and gender, but were only included when univariate significantly associated with subgroup. For ADNI, this was only age ($p = 0.0029$, ANOVA F-test). For PPMI, this was only gender ($p = 0.0017$, χ^2-test).

In the post-hoc analysis, only complete cases were included, i.e. patients with missing values were ignored.

Availability of supporting source code and requirements

A complete implementation of VaDER in Python/Tensorflow: https://github.com/johanndejong/VaDER.

An R-package for streamlining the processing of PPMI data: https://github.com/patzaw/PPMI-R-package-generator

Other code used for generating results presented in this paper: https://github.com/johanndejong/VaDER_supporting_code

List of abbreviations

- AD: Alzheimer's disease
- ADAS-13: The Alzheimer's disease assessment scale
- ADNI: Alzheimer's Disease Neuroimaging Initiative
- CDRSB: The clinical dementia rating sum of box score.
- CSF: cerebrospinal fluid
- ESS: Epworth sleepiness scale.
- FAQ: The functional activities questionnaire.
- LSTM: long short term memory
- MAR: missing at random
- MCAR: missing completely at random
- MMSE: mini-mental state examination
- MNAR: missing not at random
- PD: Parkinson's disease
- PIGD: postural instability and gait disturbance.
- PPMI: Parkinson's Progression Markers Initiative
- RBD: REM sleep behavior disorder.
- SCOPA: scales for outcomes in Parkinson's disease.
- TD: tremor-dominant
- UPDRS: Unified Parkinson's disease rating scale.
- UPDRS1: Unified Parkinson's disease rating scale, part 1.
- UPDRS2: Unified Parkinson's disease rating scale, part 2.
- UPDRS3: Unified Parkinson's disease rating scale, part 3.
- VaDE: variational deep embedding
- VaDER: variational deep embedding with recurrence
- VAR: vector auto regression
- VCF: variant call format

Competing interests

JdJ and HF received salaries from UCB Biosciences GmbH. UCB Biosciences GmbH had no influence on the content of this work.

Funding

The research leading to these results has received partial support from the Innovative Medicines Initiative Joint Undertaking under grant agreement #115568, resources of which are composed of financial contribution from the European Union's Seventh Framework Programme (FP7/2007-2013) and EFPIA companies' in kind contribution.

Authors' contributions

Method development: JdJ, HF; implementation and testing: JdJ; Data preparation: MAE, PW, RK, MS, AA; image analysis: HV; supervision: HF, MHA; definition of research project: HF

Acknowledgements

Data collection and sharing for this project was funded by the Alzheimer's Disease Neuroimaging Initiative (ADNI) (National Institutes of Health Grant U01 AG024904) and DOD ADNI (Department of Defense award number W81XWH-12-2-0012). ADNI is funded by the National Institute on Aging, the National Institute of Biomedical Imaging and Bioengineering, and through generous contributions from the following: AbbVie, Alzheimer's Association; Alzheimer's Drug Discovery Foundation; Araclon Biotech; BioClinica, Inc.; Biogen; Bristol-Myers Squibb Company; CereSpir, Inc.; Cogstate; Eisai Inc.; Elan Pharmaceuticals, Inc.; Eli Lilly and Company; EuroImmun; F. Hoffmann-La Roche Ltd and its affiliated company Genentech, Inc.; Fujirebio; GE Healthcare; IXICO Ltd.; Janssen Alzheimer Immunotherapy Research & Development, LLC.; Johnson & Johnson Pharmaceutical Research & Development LLC.; Lumosity; Lundbeck; Merck & Co., Inc.; Meso Scale Diagnostics, LLC.; NeuroRx Research; Neurotrack Technologies; Novartis Pharmaceuticals Corporation; Pfizer Inc.; Piramal Imaging; Servier; Takeda Pharmaceutical Company; and Transition Therapeutics. The Canadian Institutes of Health Research is providing funds to support ADNI clinical sites in Canada. Private sector contributions are facilitated by the Foundation for the National Institutes of Health (www.fnih.org). The grantee organization is the Northern California Institute for Research and Education, and the study is coordinated by the Alzheimer's Therapeutic Research Institute at the University of Southern California. ADNI data are disseminated by the Laboratory for Neuro Imaging at the University of Southern California.

Data used in the preparation of this article were obtained from the Parkinson's Progression Markers Initiative (PPMI) database (www.ppmi-info.org/data). For up-todate information on the study, visit www.ppmi-info.org. PPMI – a public-private partnership – is funded by the Michael J. Fox Foundation for Parkinson's Research and funding partners. A list of names of all of the PPMI funding partners can be found at www.ppmi-info.org/about-ppmi/who-we-are/study-sponsors/.

References

1. Hruby A, Hu FB. The Epidemiology of Obesity: A Big Picture. Pharmacoeconomics 2015 Jul;33(7):673–689. https://www.ncbi.nlm.nih.gov/pubmed/25471927, 25471927[pmid].
2. van Tilburg J, van Haeften TW, Pearson P, Wijmenga C. Defining the genetic contribution of type 2 diabetes mellitus. Journal of Medical Genetics 2001;38(9):569–578. https://jmg.bmj.com/content/38/9/569.
3. Cordell HJ, Todd JA. Multifactorial inheritance in type 1 diabetes. Trends in Genetics 1995;11(12):499 – 504. http://www.sciencedirect.com/science/article/pii/S016895250089160X.
4. Ruppert V, Maisch B. Genetics of HumanHypertension. Herz 2003 Dec;28(8):655–662. https://doi.org/10.1007/s00059-003-2516-6.
5. Poulter N. Coronary heart disease is a multifactorial disease. American Journal of Hypertension 1999;12(10, Supplement 1):92S – 95S. http://www.sciencedirect.com/science/article/pii/S0895706199001636.
6. Hastie T, Tibshirani R, Friedman JH. The elements of statistical learning: data mining, inference, and prediction, 2nd Edition. Springer series in statistics, Springer; 2009. http://www.worldcat.org/oclc/300478243.
7. Kannan R, Vempala S. On Clusterings - Good, Bad and Spectral. In: Proc. Symp. Found. Comp. Sci.; 2000. p. 367–377.
8. Jain A, Dubes R. Algorithms for Clustering Data. Englewood Cliffs, NJ: Prentice-Hall; 1988.
9. Fukunaga K, Hostetler LD. The estimation of the gradient of a density function, with applications in pattern recognition. IEEE Transactions on Information Theory 1975;21:32–39.
10. Aghabozorgi S, Seyed Shirkhorshidi A, Ying Wah T. Time-series Clustering - A Decade Review. Inf Syst 2015 Oct;53(C):16–38. http://dx.doi.org/10.1016/j.is.2015.04.007.
11. Rani S, Sikka G. Article: Recent Techniques of Clustering of Time Series Data: A Survey. International Journal of Computer Applications 2012 August;52(15):1–9. Full text available.
12. Warren Liao T. Clustering of time series data—a survey. Pattern Recognition 2005;38(11):1857–1874.
13. Ghassempour S, Girosi F, Maeder A. Clustering Multivariate Time Series Using Hidden Markov Models. International Journal of Environmental Research and Public Health 2014;11(3):2741–2763. http://www.mdpi.com/1660-4601/11/3/2741.
14. Sun J. Clustering multivariate time series based on Riemannian manifold. Electronics Letters 2016 September;52:1607–1609(2). https://digital-library.theiet.org/content/journals/10.1049/el.2016.0701.
15. Rubin DB. Inference and Missing Data. Biometrika 1976;63(3):581–592. http://www.jstor.org/stable/2335739.
16. Kang H. The prevention and handling of the missing data. Korean Journal of Anesthesiology 2013 May;64(5):402–406. https://www.ncbi.nlm.nih.gov/pmc/articles/PMC3668100/.
17. Mikolov T, Sutskever I, Chen K, Corrado G, Dean J. Distributed Representations of Words and Phrases and Their Compositionality. In: Proceedings of the 26th International Conference on Neural Information Processing Systems - Volume 2 NIPS'13, USA: Curran Associates Inc.; 2013. p. 3111–3119. http://dl.acm.org/citation.cfm?id=2999792.2999959.
18. Hinton GE, Salakhutdinov RR. Reducing the Dimensionality of Data with Neural Networks. Science 2006;313(5786):504–507. http://science.sciencemag.org/content/313/5786/504.
19. Frome A, Corrado GS, Shlens J, Bengio S, Dean J, Ranzato MA, et al. DeViSE: A Deep Visual-Semantic Embedding Model. In: Burges CJC, Bottou L, Welling M, Ghahramani Z, Weinberger KQ, editors. Advances in Neural Information Processing Systems 26 Curran Associates, Inc.; 2013.p. 2121–2129. http://papers.nips.cc/paper/5204-devise-a-deep-visual-semantic-embedding-model.pdf.
20. Asgari E, Mofrad MRK. Continuous Distributed Representation of Biological Sequences for Deep Proteomics and Genomics. PLOS ONE 2015 11;10(11):1–15. https://doi.org/10.1371/journal.pone.0141287.
21. Trigeorgis G, Bousmalis K, Zafeiriou S, Schuller B. A Deep Semi-NMF Model for Learning Hidden Representations. In: Xing EP, Jebara T, editors. Proceedings of the 31st International Conference on Machine Learning, vol. 32 of

Proceedings of Machine Learning Research Bejing, China: PMLR; 2014. p. 1692–1700. http://proceedings.mlr.press/v32/trigeorgis14.html.
22. Xie J, Girshick R, Farhadi A. Unsupervised Deep Embedding for Clustering Analysis. In: Proceedings of the 33rd International Conference on International Conference on Machine Learning - Volume 48 ICML'16, JMLR.org; 2016. p. 478–487. http://dl.acm.org/citation.cfm?id=3045390.3045442.
23. Jiang Z, Zheng Y, Tan H, Tang B, Zhou H. Variational Deep Embedding: An Unsupervised and Generative Approach to Clustering. In: IJCAI ijcai.org; 2017. p. 1965–1972.
24. Hochreiter S, Schmidhuber J. Long Short-Term Memory. Neural Comput 1997 Nov;9(8):1735–1780. http://dx.doi.org/10.1162/neco.1997.9.8.1735.
25. Petersen RC, Aisen PS, Beckett LA, Donohue MC, Gamst AC, Harvey DJ, et al. Alzheimer's Disease Neuroimaging Initiative (ADNI). Neurology 2010;74(3):201–209. http://n.neurology.org/content/74/3/201.
26. Marek K, Jennings D, Lasch S, Siderowf A, Tanner C, Simuni T, et al. The Parkinson Progression Marker Initiative (PPMI). Progress in Neurobiology 2011 12;95(4):629–635.
27. Komarova NL, Thalhauser CJ. High Degree of Heterogeneity in Alzheimer's Disease Progression Patterns. PLoS Computational Biology 2011;7(11). https://doi.org/10.1371/journal.pcbi.1002251.
28. Lam B, Masellis M, Freedman M, Stuss DT, Black SE. Clinical, imaging, and pathological heterogeneity of the Alzheimer's disease syndrome. Alzheimer's Research & Therapy 2013 Jan;5(1):1. https://doi.org/10.1186/alzrt155.
29. Lewis SJG, Foltynie T, Blackwell AD, Robbins TW, Owen AM, Barker RA. Heterogeneity of Parkinson's disease in the early clinical stages using a data driven approach. Journal of Neurology, Neurosurgery & Psychiatry 2005;76(3):343–348. https://jnnp.bmj.com/content/76/3/343.
30. von Coelln R, Shulman LM. Clinical subtypes and genetic heterogeneity: of lumping and splitting in Parkinson disease. Current Opinion in Neurology 2016;29(6). https://journals.lww.com/co-neurology/Fulltext/2016/12000/Clinical_subtypes_and_genetic_heterogeneity___of.10.aspx.
31. Kingma DP, Welling M, Auto-Encoding Variational Bayes; 2013. http://arxiv.org/abs/1312.6114, cite arxiv:1312.6114.
32. Doersch C, Tutorial on Variational Autoencoders; 2016. http://arxiv.org/abs/1606.05908, cite arxiv:1606.05908.
33. Gers FA, Schraudolph NN, Schmidhuber J. Learning Precise Timing with Lstm Recurrent Networks. J Mach Learn Res 2003 Mar;3:115–143. https://doi.org/10.1162/153244303768966139.
34. Lipton ZC, Kale DC, Wetzel RC. Directly Modeling Missing Data in Sequences with RNNs: Improved Classification of Clinical Time Series. In: MLHC, vol. 56 of JMLR Workshop and Conference Proceedings JMLR.org; 2016. p. 253–270.
35. Nazábal A, Olmos PM, Ghahramani Z, Valera I. Handling Incomplete Heterogeneous Data using VAEs. CoRR 2018;abs/1807.03653. http://arxiv.org/abs/1807.03653.
36. Möller-Levet CS, Klawonn F, Cho K, Wolkenhauer O. Fuzzy Clustering of Short Time-Series and Unevenly Distributed Sampling Points. In: Berthold MR, Lenz H, Bradley E, Kruse R, Borgelt C, editors. Advances in Intelligent Data Analysis V, 5th International Symposium on Intelligent Data Analysis, IDA 2003, Berlin, Germany, August 28-30, 2003, Proceedings, vol. 2810 of Lecture Notes in Computer Science Springer; 2003. p. 330–340. https://doi.org/10.1007/978-3-540-45231-7_31.
37. Tibshirani R, Walther G. Cluster validation by prediction strength. Journal of Computational and Graphical Statistics 2005;14(3):511–528.
38. Convit A, de Asis J, de Leon MJ, Tarshish CY, Santi SD, Rusinek H. Atrophy of the medial occipitotemporal, inferior, and middle temporal gyri in non-demented elderly predict decline to Alzheimer's disease. Neurobiology of Aging 2000;21(1):19 – 26. http://www.sciencedirect.com/science/article/pii/S0197458099001074.
39. Nestor SM, Rupsingh R, Borrie M, Smith M, Accomazzi V, Wells JL, et al. Ventricular enlargement as a possible measure of Alzheimer's disease progression validated using the Alzheimer's disease neuroimaging initiative database. Brain 2008 07;131(9):2443–2454. https://doi.org/10.1093/brain/awn146.
40. Butterfield DA, Halliwell B. Oxidative stress, dysfunctional glucose metabolism and Alzheimer disease. Nature Reviews Neuroscience 2019;20(3):148–160. https://doi.org/10.1038/s41583-019-0132-6.
41. Tapiola T, Alafuzoff I, Herukka SK, Parkkinen L, Hartikainen P, Soininen H, et al. Cerebrospinal Fluid Beta-Amyloid 42 and Tau Proteins as Biomarkers of Alzheimer-Type Pathologic Changes in the Brain. JAMA Neurology 2009 03;66(3):382–389. https://doi.org/10.1001/archneurol.2008.596.
42. Moisan F, Kab S, Mohamed F, Canonico M, Le Guern M, Quintin C, et al. Parkinson disease male-to-female ratios increase with age: French nationwide study and meta-analysis. Journal of Neurology, Neurosurgery & Psychiatry 2016;87(9):952–957. https://jnnp.bmj.com/content/87/9/952.
43. Schrag A, Jahanshahi M, Quinn N. What contributes to quality of life in patients with Parkinson's disease? Journal of Neurology, Neurosurgery & Psychiatry 2000;69(3):308–312. https://jnnp.bmj.com/content/69/3/308.
44. Sheikh JI, Yesavage JA. Geriatric Depression Scale (GDS): Recent evidence and development of a shorter version. Clinical Gerontologist: The Journal of Aging and Mental Health 1986;5(1-2):165–173.
45. Marsh L. Depression and Parkinson's disease: current knowledge. Curr Neurol Neurosci Rep 2013 Dec;13(12):409–409. https://www.ncbi.nlm.nih.gov/pubmed/2419078, 24190780[pmid].
46. Pitcher TL, Melzer TR, MacAskill MR, Graham CF, Livingston L, Keenan RJ, et al. Reduced striatal volumes in Parkinson's disease: a magnetic resonance imaging study. Translational Neurodegeneration 2012 Aug;1(1):17. https://doi.org/10.1186/2047-9158-1-17.
47. Pedersen AB, Mikkelsen EM, Cronin-Fenton D, Kristensen NR, Pham TM, Pedersen L, et al. Missing data and multiple imputation in clinical epidemiological research. Clin Epidemiol 2017 Mar;9:157–166. https://www.ncbi.nlm.nih.gov/pubmed/28352203, 28352203[pmid].
48. Marston L, Carpenter JR, Walters KR, Morris RW, Nazareth I, Petersen I. Issues in multiple imputation of missing data for large general practice clinical databases. Pharmacoepidemiology and Drug Safety 2010;19(6):618–626. https://onlinelibrary.wiley.com/doi/abs/10.1002/pds.1934.
49. the ADNI team. ADNIMERGE: Alzheimer's Disease Neuroimaging Initiative; 2018, r package version 0.0.1.
50. Desikan R, Ségonne F, Fischl B, Quinn BT, Dickerson BC, Blacker D, et al. An automated labeling system for subdividing the human cerebral cortex on MRI scans into gyral based regions of interest. NeuroImage 2006;31(3):968–980. https://doi.org/10.1016/j.neuroimage.2006.01.021.
51. Destrieux C, Fischl B, Dale AM, Halgren E. Automatic parcellation of human cortical gyri and sulci using standard anatomical nomenclature. NeuroImage 2010;53(1):1–15. http://dblp.uni-trier.de/db/journals/

neuroimage/neuroimage53.html#DestrieuxFDH10.

52. Sims C. Macroeconomics and Reality. Econometrica 1980;48(1):1–48. https://EconPapers.repec.org/RePEc:ecm:emetrp:v:48:y:1980:i:1:p:1-48.
53. Rand WM. Objective criteria for the evaluation of clustering methods. Journal of the American Statistical Association 1971;66(336):846–850.
54. Hubert L, Arabie P. Comparing partitions. Journal of classification 1985;2(1):193–218. http://scholar.google.de/scholar.bib?q=info:IkrWWF2JxwoJ:scholar.google.com/&output=citation&hl=de&ct=citation&cd=0.

Click here to access/download
Supplementary Material
supplemental_material.pdf

Personal Cover Click here to download Personal Cover letter_to_the_editor.pdf

Prof. Dr. Holger Fröhlich
University of Bonn
Bonn-Aachen International Center for IT
Endenicher Allee 19a
53115 Bonn, Germany

To the Editor in Chief
GigaScience

Dear Editor,

Submission of our manuscript "Deep learning for clustering of multivariate clinical patient trajectories with missing values"

I would like to draw your attention towards our manuscript entitled "Deep learning for clustering of multivariate clinical patient trajectories with missing values" for review by GigaScience. In this work, we propose a new method for clustering multivariate short time series with potentially many missing values, and we demonstrate its potential in Alzheimer's and Parkinson's disease patient stratification, within a broader context of precision medicine.

In precision medicine, the goal is to select treatments on a per-patient basis. One facet of this aim is to identify patients with differences in their disease progression. However, for many multifactorial diseases, such as neurological disorders, there is no single outcome measure that describes clinical disease progression. Hence, grouping patients by disease progression translates into a highly complex problem of clustering multivariate times series. An additionally complicating factor is that in real clinical studies typically large numbers of missing values are observed, e.g. due to patient drop out. No standard methods currently exist for this setting.

For this purpose, we propose a new deep learning-based method, variational deep embedding with recurrence (VaDER). We first technically validate VaDER on simulated data, and then use VaDER to stratify Alzheimer's disease patients and Parkinson's disease patients into clinically divergent subgroups. Finally, we demonstrate that the observed differences in disease progression reflect known underlying aspects of Alzheimer's and Parkinson's disease.

We believe our results show that VaDER has the potential to significantly enhance future patient stratification efforts using clinical time series data, and multivariate time series clustering in general.

We hope that you will find our manuscript a valuable contribution to your journal.

Sincerely yours,

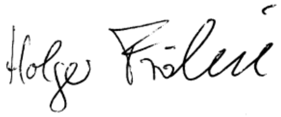

Holger Fröhlich
