# Supplementary material for: Deep learning for clustering of multivariate clinical patient trajectories with missing values
Source: Gigascience. 2019 Nov 15;8(11):giz134. doi: 10.1093/gigascience/giz134 (PMC6857688; doi:10.1093/gigascience/giz134)
Supplement: giz134_GIGA-D-19-00209_Revision_1 [file giz134_giga-d-19-00209_revision_1.pdf]

GigaScience

Deep learning for clustering of multivariate clinical patient trajectories with missing values

--Manuscript Draft--

Manuscript Number:	GIGA-D-19-00209R1
Full Title:	Deep learning for clustering of multivariate clinical patient trajectories with missing values
Article Type:	Technical Note
Funding Information:	Innovative Medicines Initiative () (115568) — Dr. Martin Hofmann-Apitius
Abstract:	Background. Precision medicine requires a stratification of patients by disease presentation that is sufficiently informative to allow for selecting treatments on a per-patient basis. For many diseases, such as neurological disorders, this stratification problem translates into a complex problem of clustering multivariate and relatively short time series, because (1) these diseases are multifactorial and not well described by single clinical outcome variables, and (2) disease progression needs to be monitored over time. Clinical datasets often additionally suffer from the presence of many missing values, further complicating any clustering attempts.<br>Findings. The problem of clustering multivariate short time series with many missing values has generally not been well addressed in the literature so far. In this work, we propose a deep learning-based method to address this issue, variational deep embedding with recurrence (VaDER). VaDER relies on a Gaussian mixture variational autoencoder framework, which is further extended by (1) incorporating long short term memory units and (2) defining an appropriate approach to directly deal with missing values via implicit imputation and loss re-weighting. We validated VaDER by accurately recovering clusters from simulated and benchmark data with known ground truth clustering, while varying the degree of missingness. We then used VaDER to successfully stratify Alzheimer's disease (AD) patients and Parkinson's disease (PD) patients into subgroups characterized by clinically divergent disease progression profiles. Additional analyses demonstrated that these clinical differences reflected known underlying aspects of AD and PD.<br>Conclusions. We believe our results show that VaDER can be of great value for future efforts in patient stratification, and multivariate short time series clustering in general
Corresponding Author:	Johann de Jong, Ph.D.<br>UCB Biosciences GmbH<br>LEVERKUSEN, Nordrhein-Westfalen GERMANY
Corresponding Author Secondary Information:	
Corresponding Author's Institution:	UCB Biosciences GmbH
Corresponding Author's Secondary Institution:	
First Author:	Johann de Jong, Ph.D.
First Author Secondary Information:	
Order of Authors:	Johann de Jong, Ph.D.<br>Mohammed Asif Emon<br>Ping Wu<br>Reagon Karki<br>Meemansa Sood<br>Patrice Godard<br>Ashar Ahmad

Powered by Editorial Manager® and ProduXion Manager® from Aries Systems Corporation

	Henri Vrooman
	Martin Hofmann-Apitius
	Holger Froehlich
**Order of Authors Secondary Information:**	
**Response to Reviewers:**	RESPONSE TO REVIEWER 1<br>Following the reviewer's suggestion, we extended the technical validation by including four real-world benchmark datasets for multivariate time series classification, to assess how well VaDER and other methods can recover the a priori known classes.<br>Morover, we extended the missingness analyses by adding experiments comparing VaDER's implicit imputation directly with pre-imputed inputs, for various degrees and modes of missingness.<br>Regarding comparing VaDER with other methods for multivariate (short) time series clustering, we appreciate the suggestions and references given by the reviewer, but after careful examination must conclude that VaDER can unfortunately not be directly compared with the methods in these references:<br>•Paparrizos et al.: k-shape does not support multivariate time series.<br>•Mikalsen et al.: While it would be very interesting to compare TCK to VaDER, unfortunately no free software implementation of TCK is available. Although the paper states that an R implementation is available, after a correspondence with the authors it appears this is not the case.<br>•Nazabal et al.: Although there is some resemblance in imputation techniques, HI-VAE was not designed for time series, and can therefore not be compared to VaDER.<br>To nonetheless address the reviewer's request for a more extensive comparison with other methods, we extended our comparison to include hierarchical clustering using two other distance/similarity measures specifically designed for multivariate time series: (1) multi-dimensional dynamic time warping (Tormene et al, 2008) and (2) global alignment kernels (Cuturi et al., 2011).<br>We agree with the reviewer that it is not clear whether VaDER performs better than the other methods on clustering the ADNI/PPMI data. We would however argue that it is not possible to unambiguously determine which method is better, because no ground-truth clustering is available for these two datasets.<br>Likewise, determining an unambiguously correct number of clusters is difficult. This strongly depends on the method, the data and the question of interest, the general treatment of which we consider outside the scope of our current work. We agree with the reviewer that if we were to use the gap between the null and the model, we would choose k = 2 for the ADNI data. However, we instead chose a number of clusters that we could demonstrate performs significantly better than expected by random chance, while still allowing VaDER enough flexibility to demonstrate its ability to uncover interactions between the time series. The ability to uncover interactions between variables is one of the main advantages of taking a multivariate approach to time series clustering, and hence one that we wish to highlight in this manuscript. By interactions, we mean e.g. distinguishing patients on one cognitive assessment that are indistinguishable on another cognitive assessment. We agree with the reviewer that this should have been better clarified in the manuscript, and we have therefore adjusted the text accordingly.<br><br>RESPONSE TO REVIEWER 2<br>While we cannot directly compare VaDER to VaDE, because VaDE does neither model time series nor handle missing values, we appreciate the reviewer's request for quantitatively assessing how implicit imputation affects the results. We therefore extended the missingness analyses by adding experiments comparing VaDER with implicit imputation directly to VaDER with pre-imputed inputs, for various degrees and modes of missingness.<br>We furthermore extended our method comparison to include hierarchical clustering using two other distance measures specifically designed for multivariate time series: (1) multi-dimensional dynamic time warping (Tormene et al, 2008) and (2) global alignment kernels (Cuturi et al., 2011).
**Additional Information:**	
**Question**	**Response**
Are you submitting this manuscript to a	No

Powered by Editorial Manager® and ProduXion Manager® from Aries Systems Corporation

special series or article collection?	
**Experimental design and statistics**<br><br>Full details of the experimental design and statistical methods used should be given in the Methods section, as detailed in our Minimum Standards Reporting Checklist. Information essential to interpreting the data presented should be made available in the figure legends.<br><br>Have you included all the information requested in your manuscript?	Yes
**Resources**<br><br>A description of all resources used, including antibodies, cell lines, animals and software tools, with enough information to allow them to be uniquely identified, should be included in the Methods section. Authors are strongly encouraged to cite Research Resource Identifiers (RRIDs) for antibodies, model organisms and tools, where possible.<br><br>Have you included the information requested as detailed in our Minimum Standards Reporting Checklist?	Yes
**Availability of data and materials**<br><br>All datasets and code on which the conclusions of the paper rely must be either included in your submission or deposited in publicly available repositories (where available and ethically appropriate), referencing such data using a unique identifier in the references and in the "Availability of Data and Materials" section of your manuscript.<br><br>Have you have met the above requirement as detailed in our Minimum Standards Reporting Checklist?	Yes

Click here to access/download;LaTeX - Main Document (TeX file);manuscript.tex

OXFORD (GIGA)^n SCIENCE

GigaScience, 2017, 1–14

doi: xx.xxxx/xxxx
Manuscript in Preparation
Paper

PAPER

Deep learning for clustering of multivariate clinical patient trajectories with missing values

Johann de Jong[1,*], Mohammad Asif Emon[2,3], Ping Wu[4], Reagon Karki[2,3], Meemansa Sood [2,3], Patrice Godard[6], Ashar Ahmad[3], Henri Vrooman[5], Martin Hofmann-Apitius[2,3] and Holger Fröhlich[1,3,*]

[1]UCB Biosciences GmbH, 40789 Monheim, Germany and [2]Fraunhofer Institute for Algorithms and Scientific Computing, 53754 Sankt Augustin, Germany and [3]Bonn-Aachen International Center for IT, University of Bonn, 53115 Bonn, Germany and [4]UCB Pharma, Slough SL1 3WE, United Kingdom and [5]Erasmus MC, University Medical Center Rotterdam, Departments of Radiology and Medical Informatics, PO Box 2040 3000 CA Rotterdam, Netherlands and [6]UCB Pharma, 1420 Braine-l'Alleud, Belgium

[*]johann.dejong@ucb.com; holger.froehlich@ucb.com; frohlich@bit.uni-bonn.de

Abstract

Background. Precision medicine requires a stratification of patients by disease presentation that is sufficiently informative to allow for selecting treatments on a per-patient basis. For many diseases, such as neurological disorders, this stratification problem translates into a complex problem of clustering multivariate and relatively short time series, because (1) these diseases are multifactorial and not well described by single clinical outcome variables, and (2) disease progression needs to be monitored over time. Additionally, clinical often additionally suffer from the presence of many missing values, further complicating any clustering attempts.
Findings. The problem of clustering multivariate short time series with many missing values is generally not well addressed in the literature so far. In this work, we propose a deep learning-based method to address this issue, variational deep embedding with recurrence (VaDER). VaDER relies on a Gaussian mixture variational autoencoder framework, which is further extended to (1) model multivariate time series and (2) directly deal with missing values. We validated VaDER by accurately recovering clusters from simulated and benchmark data with known ground truth clustering, while varying the degree of missingness. We then used VaDER to successfully stratify Alzheimer's disease (AD) patients and Parkinson's disease (PD) patients into subgroups characterized by clinically divergent disease progression profiles. Additional analyses demonstrated that these clinical differences reflected known underlying aspects of AD and PD.
Conclusions. We believe our results show that VaDER can be of great value for future efforts in patient stratification, and multivariate short time series clustering in general.

Key words: Patient stratification; deep learning; multivariate short time series; multivariate longitudinal data; clustering

Findings

Background

In precision medicine, patients are stratified based on their disease subtype, risk, prognosis, or treatment response using specialized diagnostic tests. An important question in precision medicine, is how to appropriately model disease progression and accordingly decide for the right type and time point of therapy for an individual. However, the progression of many diseases, such as neurological disorders, cardiovascular diseases, diabetes, obesity [1, 2, 3, 4, 5], is highly multifaceted and not

Compiled on: October 19, 2019.
Draft manuscript prepared by the author.

well described by one clinical outcome measure alone. Classical univariate clustering methods are likely to miss the inherent complexity of diseases that demonstrate a highly multifaceted clinical phenotype. Accordingly, stratification of patients by disease progression translates into the challenging question of how to identify a clustering of a multivariate time series.

Clustering is a fundamental and generally well investigated problem in machine learning and statistics. Its goal is to segment samples into groups (clusters), such that there is a higher degree of similarity between samples of the same cluster than between samples of different clusters. Following [6], algorithms to solve clustering problems may be put into three main categories, (1) combinatorial algorithms, (2) mixture modeling and (3) mode seeking. Within each of these three categories, a wide range of methods is available for a great diversity of clustering problems. Combinatorial algorithms do not assume any underlying probability model, but work with the data directly. Examples are K-means clustering, spectral clustering [7] and hierarchical clustering [8]. Mixture models assume that the data can be described by some probabilistic model. An example is Gaussian mixture model clustering. Finally, in mode seeking one tries to directly estimate modes of the underlying multi-modal probability density. An important example here is the mean-shift algorithm [9].

For the clustering of multivariate time series data, a few techniques have been developed [10, 11, 12, 13, 14]. However, these approaches generally rely on time series of far greater length than available in most longitudinal clinical datasets. Moreover, these methods are not suited for the large numbers of missing values often found in clinical data.

Missing values in clinical data can occur due to different reasons: (1) patients drop out of a study, e.g. due to worsening of symptoms; (2) a certain diagnostic test is not taken at a particular visit (e.g. due to lack of patient agreement), potentially resulting into missing information for entire variable groups; (3) unclear further reasons, e.g. time constraints, data quality issues, etc. From a statistical point of view, these reasons manifest into different mechanisms of missing data [15, 16]:

i. Missing completely at random (MCAR): The probability of missing information is neither related to the specific value which is supposed to be obtained, nor to other observed data. Hence, entire patient records could be skipped without introducing any bias. However, this type of missing data mechanism is probably rare in clinical studies.
ii. Missing at random (MAR): The probability of missing information depends on other observed data, but is not related to the specific missing value that is expected to be obtained. An example would be patient drop out due the to worsening of certain symptoms, which are at the same time recorded during the study.
iii. Missing not at random (MNAR): any reason for missing data, which is neither MCAR nor MAR. MNAR is problematic, because the only way to obtain unbiased estimates is to model missing data.

Multiple imputation methods have been proposed to deal with missing values in longitudinal patient data [16]. However, any imputation method will result in certain errors, and if imputation and clustering are done separately, these errors will propagate through to the following clustering procedure.

To address the problem of clustering multivariate and relatively short time series data with many missing values, in this paper we propose an approach that uses techniques from deep learning. Autoencoder networks have been highly successful in learning latent representations of data, e.g. [17, 18, 19, 20]. Specifically for clustering, autoencoders can be first used to learn a latent representation of a multivariate distribution, to then independently find clusters [21]. More recently, some authors have suggested to simultaneously learn latent representations and cluster assignments. Interesting examples are deep embedded clustering (DEC) [22] and variational deep embedding (VaDE) [23].

Here, we present a new method for clustering multivariate short time series with potentially many missing values, VaDER (variational deep embedding with recurrence). VaDER is in part based on VaDE [23], a clustering algorithm based on variational autoencoder principles, with a latent representation forced towards a multivariate Gaussian mixture distribution. Additionally, VaDER (1) integrates two LSTM networks [24] into its architecture, to allow for the analysis of multivariate short time series, and (2) adopts an approach of implicit imputation and loss re-weighting to account for the typically high degree of missingness in clinical data.

After a validation of VaDER via simulation and benchmark studies, we applied the method to the problem of patient stratification in Alzheimer's disease (AD) and Parkinson's disease (PD), using data from the Alzheimer's Disease Neuroimaging Initiative (ADNI) [25] and the Parkinson's Progression Markers Initiative (PPMI) [26] respectively. Alzheimer's and Parkinson's disease are multifactorial and highly heterogeneous diseases, both in clinical and biological presentation, as well as in progression [27, 28, 29, 30]. For example, PD is characterized by motor symptoms, behavioral changes (e.g. sleeping disorders) as well as cognitive impairment [1]. Cognitive impairment, one of the hallmarks of AD, is not straightforward to assess, since cognition itself is highly multifaceted, and described by e.g. orientation, speech and memory. Consequently, in the field of AD, a wide range of tests have been developed to assess different aspects of cognition.

This heterogeneity presents one of the major challenges in understanding these diseases and developing new treatments. As such, better clustering (stratification) of patients by disease presentation could be of great help in improving disease management and designing better clinical trials that specifically focus on treating patients that are rapidly progressing.

Our analyses of the ADNI and PPMI data show that VaDER is highly effective at disentangling multivariate patient trajectories into clinically meaningful patient subgroups.

Results

Variational autoencoders for clustering

Our proposed variational deep embedding with recurrence (VaDER) method is in part based on variational deep embedding (VaDE) [23], a variational autoencoding clustering algorithm with a multivariate Gaussian mixture prior. In variational autoencoding algorithms, the training objective is to optimize the variational lower bound on the marginal likelihood of a data point $\mathbf{x}$ [31]:

$$\mathcal{L}(\mathbf{x}) = \mathbb{E}_{q(\mathbf{z}|\mathbf{x})}[\log(p(\mathbf{x}|\mathbf{z}))] - D_{KL}(q(\mathbf{z}|\mathbf{x})||p(\mathbf{z})) \qquad (1)$$

This lower bound can be seen as composed of two parts. The first term corresponds to the likelihood of seeing $\mathbf{x}$ given a latent representation $\mathbf{z}$. Its negative is often called the *reconstruction loss*, and it forces the algorithm to learn good reconstructions of its input data. The negative of the second term is often called the *latent loss*. It is the Kullback-Leibler divergence of the prior $p(\mathbf{z})$ to the variational posterior $q(\mathbf{z}|\mathbf{x})$, and

1 https://www.ninds.nih.gov/Disorders/All-Disorders/Parkinsons-Disease-Information-Page

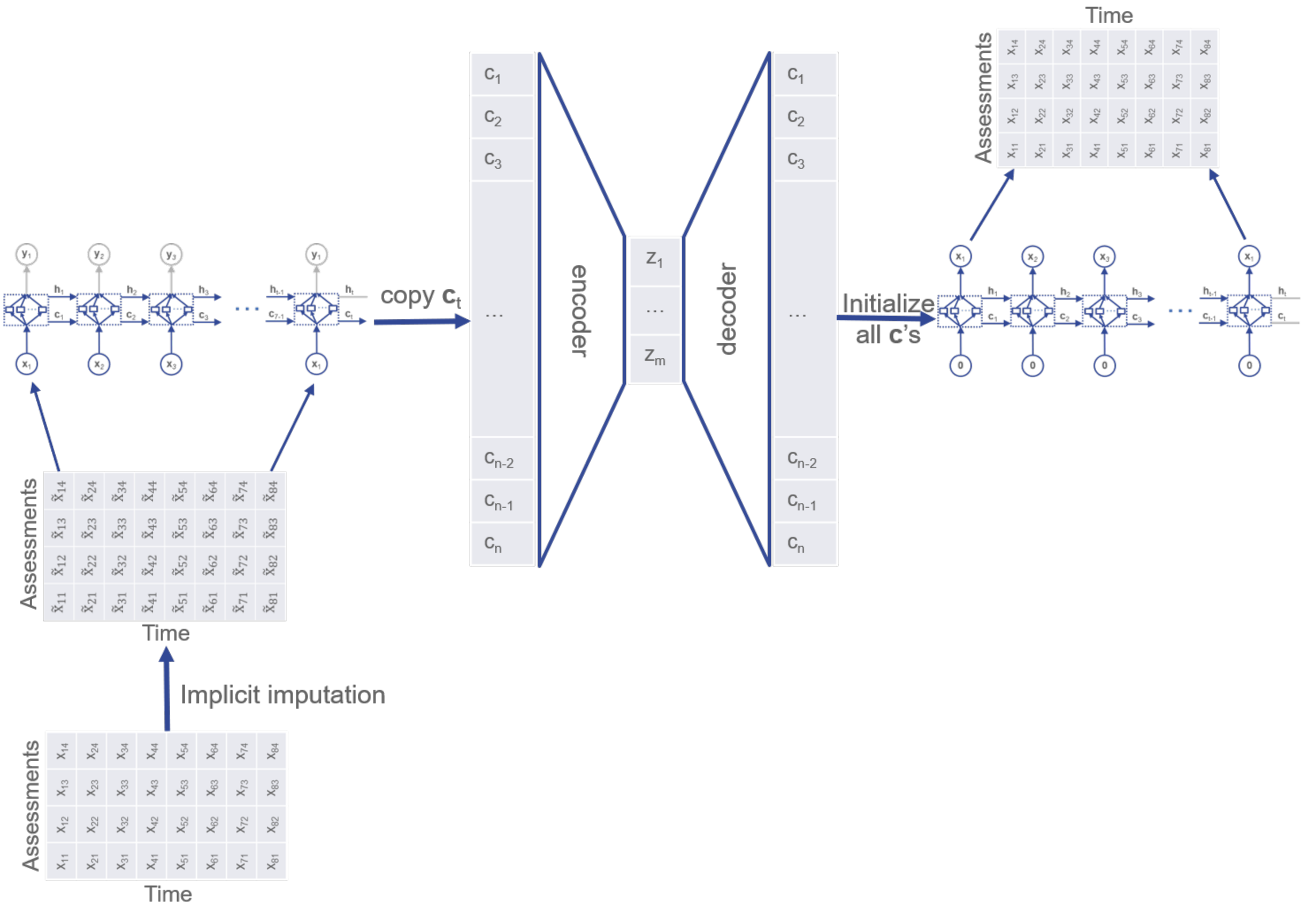

Figure 1. VaDER architecture

it regularizes the latent representation $\mathbf{z}$ to lie on a manifold specified by the prior $p(\mathbf{z})$.

In VaDE, this prior is a multivariate Gaussian mixture. Accordingly including a parameter for choosing a cluster c, the variational lower bound can then be written as follows:

$$\mathcal{L}(\mathbf{x}) = \mathbb{E}_{q(\mathbf{z},c|\mathbf{x})}[\log(p(\mathbf{x}|\mathbf{z}))] - D_{KL}(q(\mathbf{z},c|\mathbf{x})||p(\mathbf{z},c)) \quad (2)$$

By forcing the latent representation $\mathbf{z}$ towards a multivariate Gaussian mixture distribution, VaDE has the ability to simultaneously learn latent representations and cluster assignments of its input data. For more details on variational autoencoders and VaDE, we refer the reader to [32, 31, 23].

Variational deep embedding with recurrence (VaDER)

VaDER is an autoencoder-based method for clustering multivariate short time series with potentially many missing values. For simultaneously learning latent representations and cluster assignments of its input samples, VaDER uses the VaDE latent loss as described above and in [23].

To model the auto- and cross-correlations in multivariate short time series data, we integrate peephole LSTM networks [24, 33] into the autoencoder architecture (Figure 1).

To deal with missing values, we directly integrate imputation into model training. As outlined in Section Background, separating imputation from clustering can potentially introduce bias. To avoid this bias, we here propose an implicit imputation scheme, which is performed within VaDER training. Our approach to imputation bears some similarity to other approaches [34, 35]. However, in contrast to [34], VaDER uses missingness indicators for implicit imputation as an integral part of neural network training. Additionally, in contrast to [35], our method of imputation is also suited for MNAR data, which are often encountered in clinical datasets.

We first define a weighted reconstruction loss on feature and sample level: Imputed values are weighted to 0, non-imputed values are weighted to 1. To retain the balance with the latent loss, the resulting reconstruction loss is re-scaled to match the original dimensions of the data. More formally, for a mean squared reconstruction loss, let L be the number of samples in our dataset, $\mathbf{x}^l$ a single input sample, and $\hat{\mathbf{x}}^l$ its corresponding reconstructed output ($l \in 1 \ldots L$). $\mathbf{x}^l$ and $\hat{\mathbf{x}}^l$ are matrices $\in \mathbb{R}^{N \times M}$, where N is the number of time points and M is the number of clinical outcome measures (e.g. cognitive assessments) for a particular patient. Then the unweighted mean reconstruction loss is:

$$\frac{1}{L}\sum_{l=1}^{L}\sum_{i=1}^{N}\sum_{j=1}^{M}(x_{ij}^l - \hat{x}_{ij}^l)^2 \quad (3)$$

Now, let $A := \{x_{ij}^l | x_{ij}^l \text{is missing}\}$, $\mathbf{1}_A(.)$ be the indicator function on set A, and $|A|$ be the cardinality of A. Then, the weighted mean squared reconstruction loss is:

$$\frac{NM}{|A|}\sum_{l=1}^{L}\sum_{i=1}^{N}\sum_{j=1}^{M}\mathbf{1}_A(x_{ij}^l)(x_{ij}^l - \hat{x}_{ij}^l)^2 \quad (4)$$

In addition to the weighted reconstruction loss, we adopt an implicit imputation scheme, where imputed values are learned as an integral part of model training. More specifically, Let $\mathbf{x}^l$,

N, M, x^l_{ij}, A and $\mathbf{1}_A(.)$ be defined as above. Also assume that all x^l_{ij} for which $\mathbf{1}_A(x^l_{ij}) = 1$, are initially imputed with arbitrary finite values. Then we add one additional layer before the input LSTM (Figure 1) as follows:

$$\tilde{x}^l_{ij} = x^l_{ij} \times (1 - \mathbf{1}_A(x^l_{ij})) + b_{ij} \times \mathbf{1}_A(x^l_{ij}) \quad (5)$$

Here, x^l_{ij} is the actual observed (or missing) value of sample l at time points i and assessment j, and $\tilde{x}^l_{ij}$ serves as input to the LSTM. In other words, if x^l_{ij} is missing, then it is replaced by b_{ij} in $\tilde{\mathbf{x}}$. Parameters b_{ij} are trained as an integral part of VaDER using stochastic gradient descent, and can be considered (time, assessment)-specific expected values. Note that (1) the initial arbitrary imputation does not influence the eventual clustering, and (2) the implicitly imputed values are weighted to 0 in the reconstruction loss.

VaDER achieves high accuracy on simulated data

As a first step in technically validating VaDER, we simulated data with a known ground truth clustering, and assessed how well VaDER was able to recover these clusters. A natural framework to this end is the vector autoregressive (VAR) model, because (1) it can express serial correlation between time points, (2) it can express cross-correlation between variables, and (3) given a fully parameterized VAR process, one can simulate random trajectories from that VAR process.

More specifically, to generate clusters of multivariate short time series, we simulated from VAR process mixtures, for different values of a clusterability parameter λ. The clusterability parameter λ influences how easily separable the simulated clusters are (see Section Simulation experiments). Sample data is provided in the Supplemental Material. We used the cluster purity measure [36] to record how well the true clustering could be recovered (for more details, see Section Methods).

VaDER was able to highly accurately recover the simulated clusters, achieving a cluster purity of >0.9 for $\lambda \approx 0.08$, and converging to 1.0 for larger λ (Figure 2a). Moreover, even without extensive hyperparameter optimization, VaDER performed substantially better than hierarchical clustering using various distance measures, some of which specifically designed for multivariate time series (Multidimensional Dynamic Time Warping (MD-DTW [38]) and Global Alignment Kernels (GAK [39])) or short time series (STS [37]). Only for $\lambda < 0.04$ VaDER was outperformed by multi-dimensional dynamic time-warping. This may be attributed to the fairly limited number of samples used for the simulation ($n = 2000$), and omitting extensive optimization of VaDER's hyperparameters.

We used the same VAR framework to assess how varying degrees of missing values affect the performance of VaDER. Both missing values completely at random (MCAR) and missing values not at random (MNAR) were simulated as described in Section Methods. In the MCAR simulation, missing values were uniformly distributed across time and clinical outcome measures. In the MNAR simulation, the expected degree of missing values sigmoidally depended on time (see Section Methods). For varying clusterability levels λ, it can be seen that VaDER's implicit imputation scheme is overall more robust against missing values than using VaDER with pre-imputation of missing values (Figures 2b and 2c).

VaDER achieves high accuracy on benchmark classification datasets

As an additional validation step towards applying VaDER to real-world clinical data, we collected a number of real-world benchmark datasets for multivariate time series classification (Table 1). The datasets were normalized and processed to equal and/or shorter length as described in Section Methods.

Comparing the ability of VaDER in recovering these a priori known classes to the other methods mentioned above, it can be seen that VaDER consistently achieves better results (Figure 3a). Moreover, VADER's approach of integrating imputation with model training again outperforms pre-imputation of missing values (Figures 3b and 3c).

Application 1: VaDER identifies clinically diverse AD patient subgroups

After the technical validation using simulated and benchmark data, we applied VaDER to clinical data for identifying meaningful patient subgroups. From the Alzheimer's Disease Neuroimaging Initiative (ADNI) [25], we collected data from 689 patients that were at some point diagnosed with dementia during the course of this study. Four different cognitive assessment scores were available at 8 different visits: ADAS13, CDRSB, MMSE and FAQ. We pre-processed the data as described in Section ADNI data preparation. Overall, the fraction of missing values was ~ 43%. We used VaDER to cluster patients by disease progression as measured using these cognitive assessments.

Hyperparameter optimization was performed by random grid search as described in Section Methods. For each number of clusters $k \in \{2 \dots 15\}$, the prediction strength [42] of the corresponding optimal model was compared to a null distribution (see Section Hyperparameter optimization and choice of number of clusters), which is shown in the Supplemental Materials.

For most practical applications, determining an unambiguously correct number of clusters k is not possible, and a wide range of rules-of-thumb exist, see e.g. [43, 44, 45, 46, 42]. For our subsequent analyses, we chose $k = 3$. This demonstrated relatively high prediction strength, significantly different from the null distribution, while still allowing VaDER to demonstrate its ability to uncover potentially interesting statistical interactions between trajectories of different cognitive assessments. A statistical interaction between different cognitive assessments could e.g. manifest in the ability to distinguish patient subgroups based on one cognitive assessment, while this is not possible on another assessment. Another example would be a permuted ordering of clusters with respect to different assessments scores.

For ADNI data the resulting cluster mean trajectories are shown in Figure 4, and demonstrate that (1) VaDER effectively clusters the data into patient subgroups showing divergent disease progression, and (2) VaDER is able to find interactions between the different cognitive assessments, which would be principally difficult to distill from univariate analyses of the assessments. For example, the patients in cluster 1 are the most severely progressing patients when assessed using ADAS13, CDRSB and MMSE. However, the FAQ assessment (instrumental activities of daily living) does not distinguish between these severely progressing patients and the more moderately progressing patients in cluster 1.

In addition to cognitive assessment measurements, ADNI presents a wealth of data on brain volume and various AD markers that we did not use for clustering. In this data, we identified numerous statistically significant associations with our patient subgroups. For example, the clusters strongly associated with time-to-dementia diagnosis relative to baseline, with cluster 2 showing generally the shortest time, and cluster 0 the longest. The relatively mildly progressing patients in cluster 0 also demonstrated on average a larger whole brain volume at baseline, which moreover declined less steeply over time, compared to more severely progressing patients. Especially the middle temporal gyri and fusiform gyri were larger (and shrinking more slowly over time), whereas the ventricles were smaller (and expanding more slowly over time). In-

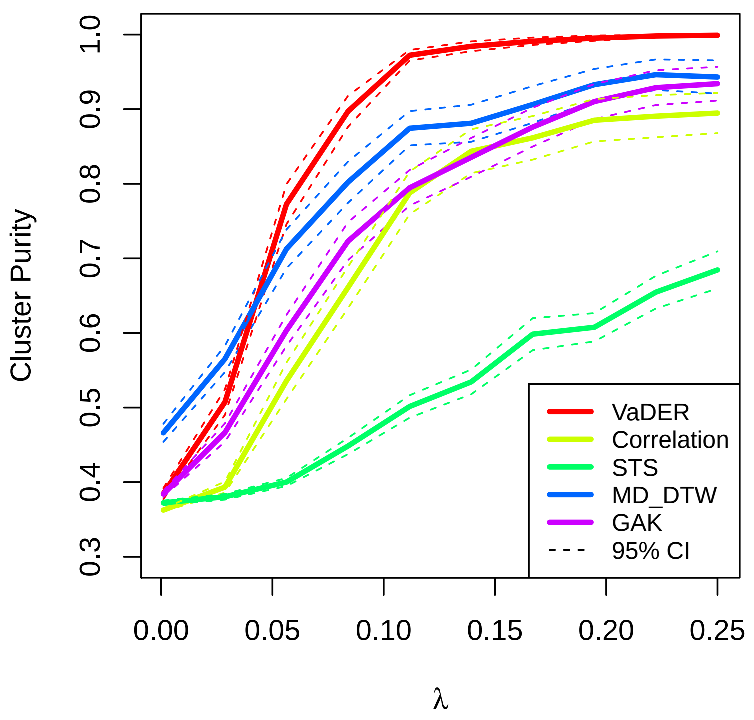

(a) Cluster purity [36] for clustering of simulated data as a function of the clusterability parameter λ, with higher λ implying a higher degree of similarity between profiles in the same cluster. Results are shown for VaDER as well as hierarchical clustering using five different distance measures, (1) Euclidean distance, (2) Pearson correlation, (3) Short time series (STS) distance [37], (4) Multi-dimensional dynamic time warping (MD_DTW) [38] and (5) Global Alignment Kernels (GAK) [39].

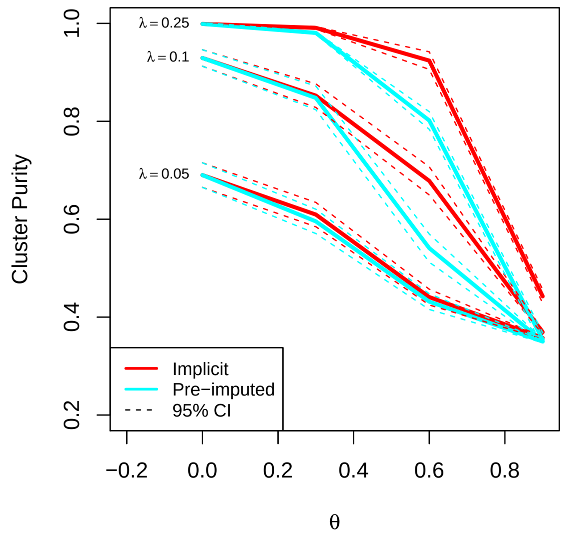

(b) Cluster purity as a function of the fraction θ of values missing completely at random (MCAR), for various levels of the clusterability parameter λ, for both VaDER with implicit imputation and VaDER with pre-imputation. Confidence intervals were determined by repeating the clustering 100 times using newly generated random data and missingness patterns.

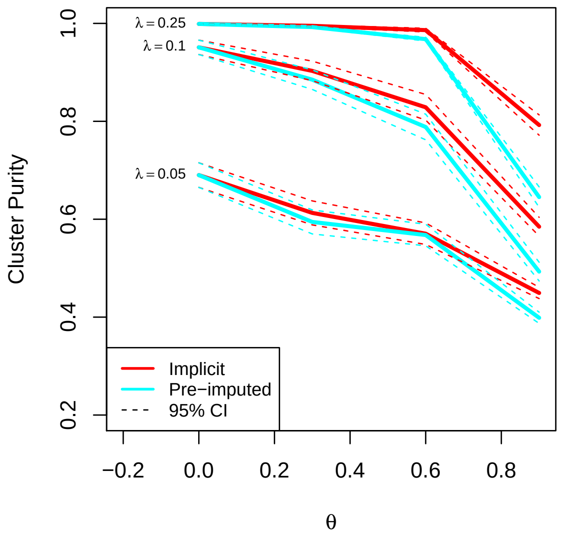

(c) Cluster purity as a function of the fraction θ of values missing not at random (MNAR) (see Section Methods for details), for various levels of the clusterability parameter λ, for both VaDER with implicit imputation and VaDER with pre-imputation. Confidence intervals were determined by repeating the clustering 100 times using newly generated random data and missingness patterns.

Figure 2. VaDER performance on simulated data, with varying degrees of clusterability and missingness.

deed, atrophy of the middle temporal gyri and fusiform gyri, as well as ventricular enlargement, have been associated with Alzheimer's disease progression [47, 48]. As another example, the more severely progressing patients (clusters 1 and es-

Table 1. Multivariate time series classification datasets used in this study.

Name	k	n	p	n_t	n'_t	Source
ArabicDigits	10	8800	13	4 - 93	24	UCI [40]
JapaneseVowels	9	640	12	7 - 29	15	UEA/UCR [41]
CharacterTrajectories	20	2858	3	109 - 205	23	UCI [40]
UWave	8	4478	3	315	25	UCI [40]

k: number of classes.
n: number of samples.
p: number of variables.
n_t: number of time points.
n'_t: number of samples after processing to equal and/or shorter length.

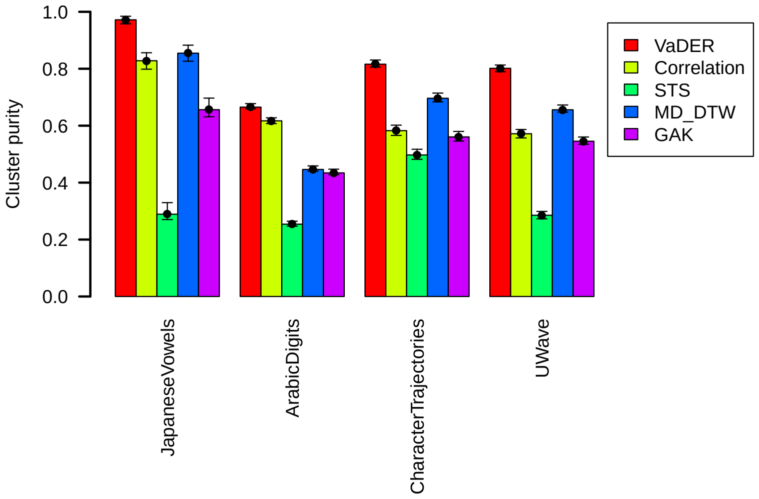

(a) Cluster purity [36] for clustering of benchmark data. Results are shown for VaDER as well as hierarchical clustering using five different distance measures, (1) Euclidean distance, (2) Pearson correlation, (3) Short time series (STS) distance [37], (4) Multi-dimensional dynamic time warping (MD_DTW) [38] and (5) Global Alignment Kernels (GAK) [39]. For each dataset, the best performance across methods is marked by a horizontal dotted line. Confidence intervals were determined by bootstrapping the clustering 10^3 times.

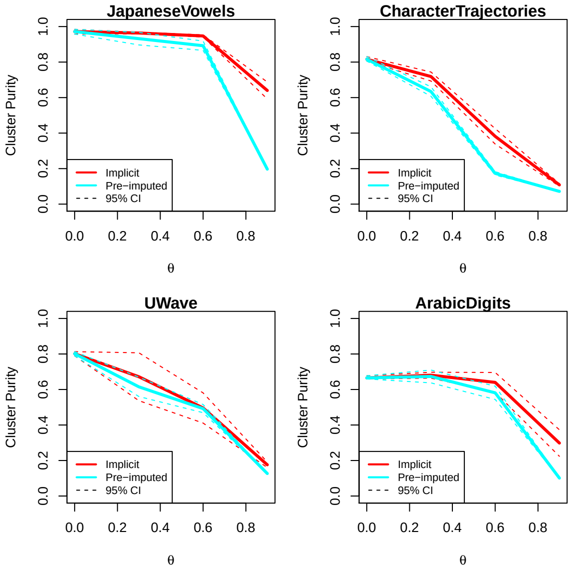

(b) Cluster purity as a function of the fraction θ of values missing completely at random (MCAR), for both VaDER with implicit imputation and VaDER with pre-imputation. Confidence intervals were by repeating the clustering 5 times using newly generated random missingness patterns.

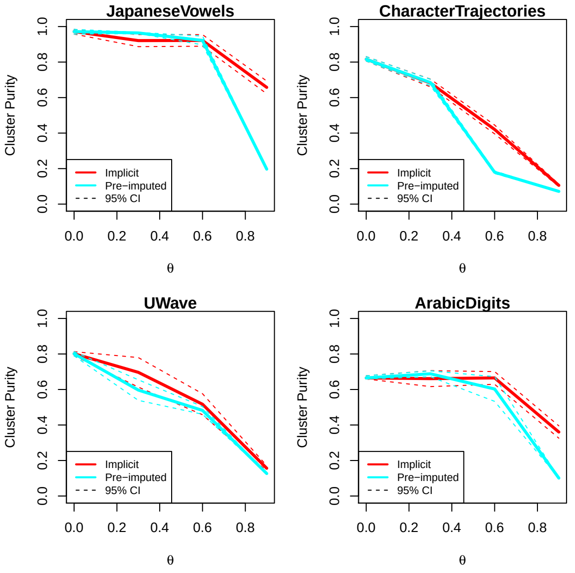

(c) Cluster purity as a function of the fraction θ of values missing not at random (MNAR), for both VaDER with implicit imputation and VaDER with pre-imputation. Confidence intervals were by repeating the clustering 5 times using newly generated random missingness patterns.

Figure 3. VaDER performance on benchmark data, for varying degrees of missingness.

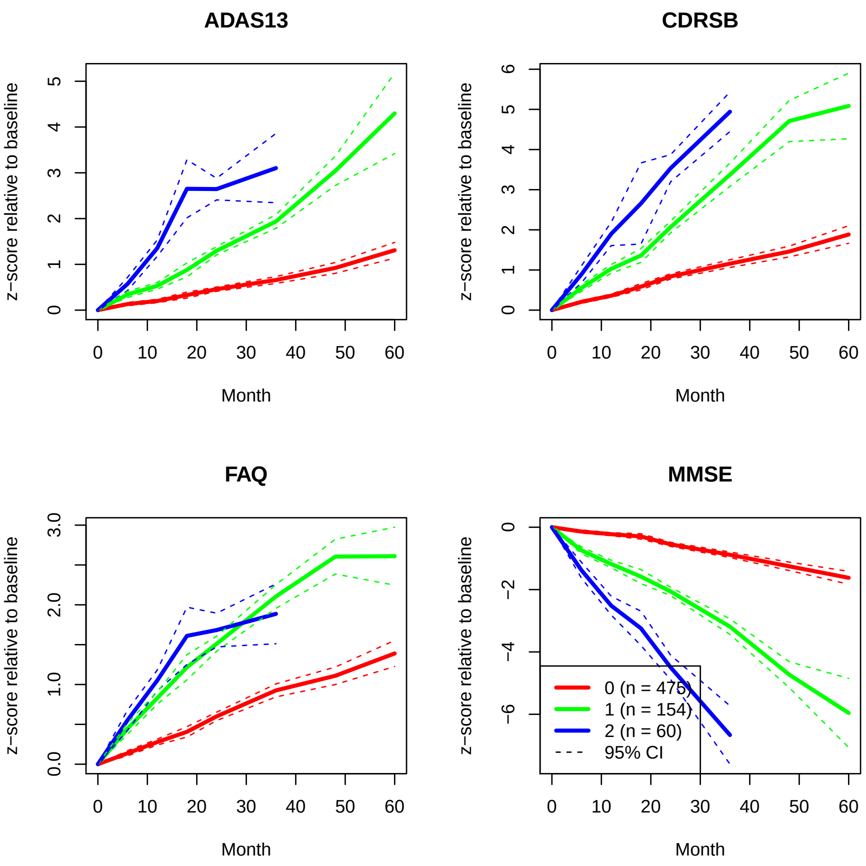

Figure 4. Normalized cluster mean trajectories relative to baseline (x-axis in months), as identified by VaDER from the ADNI cognitive assessment data.

pecially 2), demonstrated lower cerebral glucose uptake and lower cerebrospinal Abeta42 levels, again confirming the literature [49, 50] (see Section Methods and Supplemental Material). These observations demonstrate that the clinical differences between our patient subgroups reflect known Alzheimer's disease aspects.

Application 2: VaDER identifies clinically diverse PD patient subgroups

We additionally applied VaDER to clinical data from the Parkinson's Progression Markers Initiative (PPMI) [26]. From PPMI, we collected data from 362 de novo PD patients that had been diagnosed within a time period of two years before study onset and were initially not been treated. 9 variables on several motor and non-motor symptoms (UPDRS total, UPDRS1-3, TD, PIGD, RBD, ESS, SCOPA-AUT) measured at either 5 or 10 time points were available. The data was pre-processed as described in Section PPMI data preparation. Overall, the fraction of missingness values was ~ 17% (or ~ 31%, when including time points entirely missing for some assessments). We again used VaDER to cluster patients according to disease progression as measured by these assessments.

Hyperparameter optimization and selection of the number of clusters was performed in the same way as for ADNI, and we decided on $k = 3$ patient subgroups accordingly. The resulting cluster mean trajectories are shown in Figure 5. These again illustrate that (1) VaDER effectively clusters the data into clinically divergent patient subgroups, and (2) VaDER is able to find interactions between the assessments that would principally be difficult to find based on univariate analyses alone. For example, cluster 0 represents patients with a moderate progression in terms of mental impairment, behavior, and mood (UPDRS1) and autonomic dysfunction (SCOPA). However, these patients remain relatively stable, or even improve, on many other assessments, such as tremor dominance (TD), the self-reported ability to perform activities of daily life (UPDRS2) and motor symptoms evaluation (UPDRS3).

Similar to ADNI, PPMI presents a wealth of additional data on brain volume and various PD markers that were not used for clustering. Aligning these data with our PD patient subgroups, we found numerous statistically significant associations that confirmed existing literature, many related to quality of life and physiological changes to the brain. For example, men were over-represented in cluster 1, and showed the most severe disease progression, confirming the literature on gender differences in PD (e.g. [51]). Moreover, these severely progressing patients showed an expected steeply declining ability to perform activities of daily living (modified Schwab and England score [52]), as well as rapidly developing symptoms of depression (geriatric depression scale [53]), common in PD patients [54]. Additionally, these patients demonstrated physiological differences in the brain when compared to more mildly progressing patients. Examples are the caudate nucleus and putamen brain regions, which were smaller at baseline and during follow-ups in the more severely progressing patients in cluster 1, and from the literature are known to be subject to atrophy in PD [55] (see Section Methods and Supplemental Material). These observations demonstrate that the clinical differences between our patient subgroups reflect known aspects of PD disease progression.

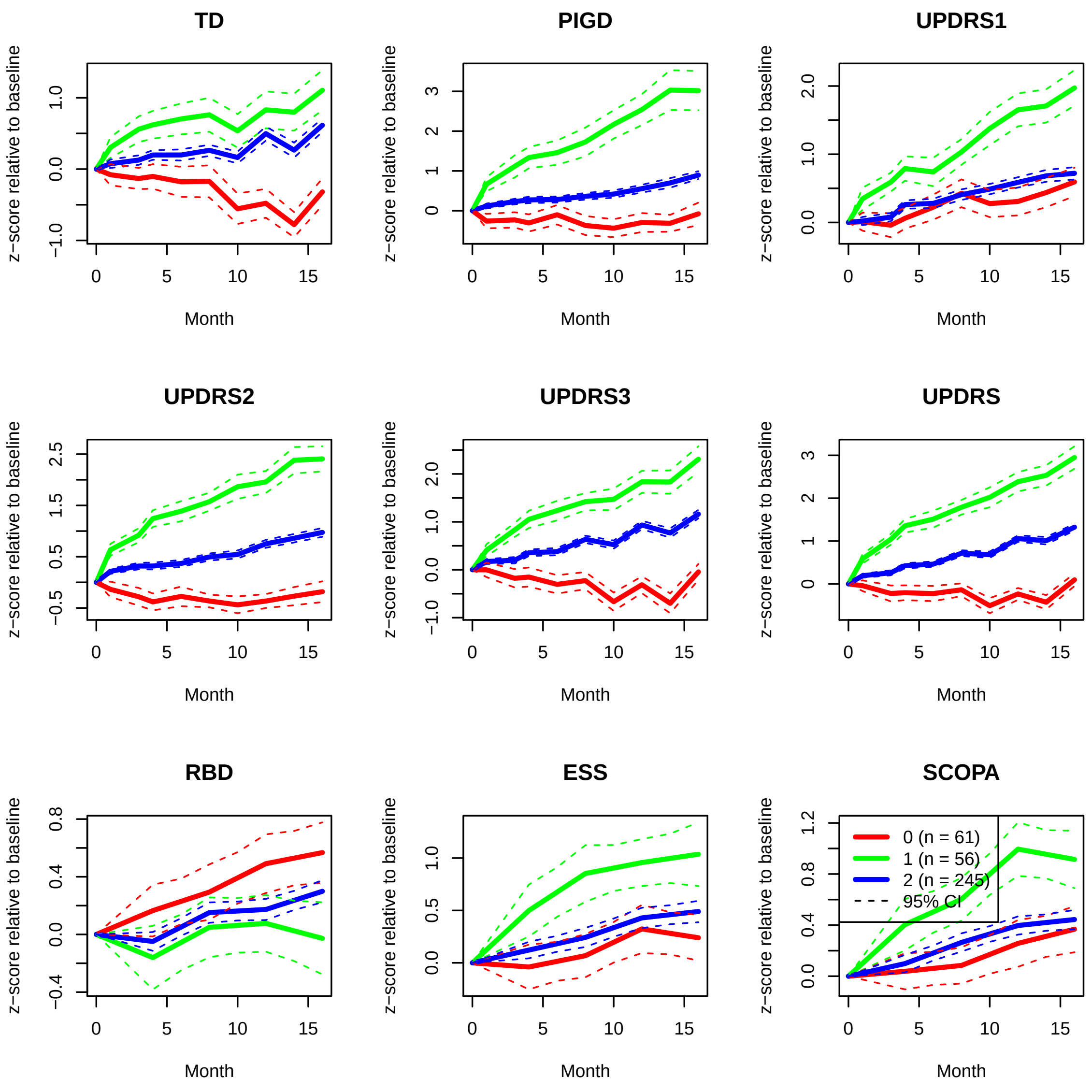

Figure 5. Normalized cluster mean trajectories relative to baseline (*x*-axis in months), as identified by VaDER from the PPMI motor/non-motor score data.

Discussion and conclusions

Identifying subgroups of patients with similar progression patterns can help to better understand the heterogeneity of complex diseases. Together with predictive machine learning methods, this might help to better decide on the right time and type of treatment for an individual patient, as well as to improve the design of clinical studies. However, one of the main challenges is the multifaceted nature of progression in many areas of disease.

In this paper, we proposed VaDER, a method for clustering multivariate short time series with potentially many missing values, a setting that seems generally not well addressed in the literature so far, but is nonetheless often encountered in clinical study data.

We validated VaDER by showing the very high accuracy on clustering simulated and real-world benchmark data with a known ground truth. We then applied VaDER to data from (1) ADNI and (2) PPMI, resulting in subgroups characterized by clinically highly divergent disease progression profiles. A comparison with other data from ADNI and PPMI, such as brain imaging, motor- and cognitive assessment data, furthermore supported the observed patient subgroups.

VaDER has two main distinctive features. One is that VaDER deals directly with missing values. For clinical research this is crucial, since clinical datasets often show a very high degree of missing values [56, 57]. The other main distinctive feature is that, as opposed to existing methods [10, 11, 12, 13, 14], VaDER is specifically designed to deal with multivariate and relatively short time series that are typical for (observational) clinical studies. However, it is worthwhile to mention that the application of VaDER is not per se limited to longitudinal clinical study data. Future applications (potentially requiring some adaptations) could e.g. include data originating from electronic health records, multiple wearable sensors, video recordings, or time series gene (co-)expression. Moreover, VaDER could be used as a generative model: given a trained model, it is possible to generate "virtual" patient trajectories.

Altogether, we believe that our results show that VaDER has the potential to enhance future patient stratification efforts, and multivariate short time series clustering in general.

Methods

Data preparation

ADNI data preparation

Data used in the preparation of this article were obtained from the Alzheimer's Disease Neuroimaging Initiative (ADNI) database (adni.loni.usc.edu). The ADNI was launched in 2003 as a public-private partnership, led by Principal Investigator Michael W. Weiner,MD. The primary goal of ADNI has been to test whether serial magnetic resonance imaging (MRI), positron emission tomography (PET), other biological markers, and clinical and neuropsychological assessment can be combined to measure the progression of mild cognitive impairment (MCI) and early Alzheimer's disease (AD). For up-to-date information, see www.adni-info.org.

The ADNIMERGE R-package [58] contains mainly two categories of data, (1) longitudinal and (2) non-longitudinal. These data represent 1737 participants that include healthy controls and patients diagnosed with Alzheimer's Disease (AD). The non-longitudinal features such as demographics and APOE e4 status were measured only once, at baseline. The longitudinal features (i.e. neuroimaging features, cerebrospinal fluid (CSF) biomarkers, cognitive tests and everyday cognition) were recorded over a span of 5 years.

Clinical data. In the current study, we have focused on those participants who were diagnosed with AD at baseline or during one of the follow-up visits. After this filtering step, we had a total of 689 patients. For these 689 patients, four cognitive assessments were selected for clustering:

- ADAS-13: The Alzheimer's disease assessment scale
- CDRSB: The clinical dementia rating sum of box score.
- FAQ: The functional activities questionnaire.
- MMSE: mini–mental state examination

The above assessments were taken at baseline and at 6, 12, 18, 24, 36 48 and 60 months after baseline. For each of the four cognitive assessments, all time points were normalized relative to baseline by (1) subtracting the baseline mean across the 689 patients, and (2) dividing by the baseline standard deviation across the 689 patients.

Imaging data. All available MR scans (T1-weighted scans) from the ADNI database were quantified by an open-source, automated segmentation pipeline at the Erasmus University Medical Center, The Netherlands. The number of slices of the T1w scans varied from 160 to 196 and the in-plane resolution was 256 x 256 on average, yielding an overall voxel-size of 1.2 x 1.0 x 1.0 mm. From the 1715 baseline ADNI scans, the volumes of 34 bilateral cortical brain regions, 68 structures in total, were calculated using a model- and surface-based automated image segmentation procedure, incorporated in the FreeSurfer Package (v.6.0, http://surfer.nmr.mgh.harvard.edu/). Segmentation in Freesurfer was performed by rigid-body registration and nonlinear normalization of images to a probabilistic brain atlas. In the segmentation process, each voxel of the MRI volumes was labeled automatically as a corresponding brain region based on two different cortex parcellation guides [59, 60], subdividing the brain into 68 and 191 regions respectively.

PPMI data preparation

Patients were selected if their PD diagnosis was less than 2 years old at baseline time, and if follow up data was available for at least 48 months (5 - 10 time points), resulting in a total of 362 patients. For these 362 patients, a set of 9 motor and non-motor symptoms were selected for clustering:

- TD: tremor-dominant
- PIGD: postural instability and gait disturbance.
- UPDRS1: Unified Parkinson's disease rating scale, part 1: mentation, behavior, and mood.
- UPDRS2: Unified Parkinson's disease rating scale, part 2: activities of daily living.
- UPDRS3: Unified Parkinson's disease rating scale, part 3: motor examination.
- UPDRS: Unified Parkinson's disease rating scale (UPDRS1 + UPDRS2 + UPDRS3).
- RBD: REM sleep behavior disorder.
- ESS: Epworth sleepiness scale.
- SCOPA-AUT: Scales for outcomes in Parkinson's disease: assessment of autonomic dysfunction.

All scores were normalized relative to baseline by (1) subtracting the baseline mean across all patients, and (2) dividing by the baseline standard deviation across all patients.

For some assessments, fewer time points were available. These were treated as missing values.

Benchmark datasets for multivariate time series classification

As no benchmark datasets exist for multivariate short time series clustering, we collected a number of benchmark datasets for multivariate time series classification [40, 41]. Since currently, VaDER still only works with equal-length time series (see also Section Discussion and conclusions), we preprocessed all samples to equal-length time series by linear interpolation between start and end point. Following [61, 62], we chose constant lengths of $\left\lceil \frac{T_{max}}{\left\lceil \frac{T_{max}}{25} \right\rceil} \right\rceil$, where T_{max} is the maximum length of the lengths of the samples in a given dataset.

Moreover, all resulting time series were standardized to zero mean and unit variance.

Variational deep embedding with recurrence (VaDER)

The VaDER model is extensively described in Section Results. This section describes how VaDER was trained.

Pre-training

Similar to [23], we pre-train VaDER by disregarding the latent loss during the first epochs, essentially fitting an non-variational LSTM autoencoder to the data. We then fit a Gaussian mixture distribution in the latent space of this autoencoder, and use its parameters to initialize the final variational training of VaDER.

Hyperparameter optimization and choice of number of clusters

We used prediction strength [42] to select suitable values for VaDER's hyperparameters. These comprise:

- number of layers (for both ADNI and PPMI: $\{1, 2\}$)
- number of nodes per hidden layer (for ADNI: $\{2^0, 2^1, 2^2, 2^3, 2^4, 2^5, 2^6\}$; for PPMI: $\{2^0, 2^1, 2^2, 2^3, 2^4, 2^5, 2^6, 2^7\}$)
- learning rate (for both ADNI and PPMI: $\{10^{-4}, 10^{-3}, 10^{-2}, 10^{-1}\}$)
- mini-batch size (for both ADNI and PPMI: $\{2^4, 2^5, 2^6, 2^7\}$)

Hyperparameter optimization was performed via a random grid search (i.e. by randomly sampling a predefined hyperparameter grid) with repeated cross-validation ($n = 20$), using the reconstruction loss as objective. This was done during the pre-training phase of VaDER.

After hyperparameter optimization we trained VaDER models for different numbers of clusters $k \in \{2 \ldots 15\}$. For each k,

prediction strength was computed by 2-fold cross-validation [42]: For a given training and test dataset:

i. Train VaDER on the training data. (the training data model)
ii. Assign clusters to the test data using the training data model.
iii. Train VaDER on the test data. (the test data model)
iv. Assign clusters to the test data using the test data model.
v. Compare the resulting two clusterings: For each cluster of the test data model, compute the fraction of pairs of samples in that cluster that are also assigned to the same cluster by the training data model. Prediction strength is defined as the minimum proportion across all clusters of the test data model. [42].

Prediction strength was then compared to an empirical null distribution of that measure. The null distribution of the prediction strength was computed by randomly permuting the predicted cluster labels 10^3 times, then recomputing the prediction strength, and eventually taking the average of the 10^3 prediction strength values. Doing this for all 20 repeats, resulted in 20 values for the eventual null distribution, which were then compared to 20 actual prediction strength values (similarly, one for each repeat) by a paired Wilcoxon rank-sum test.

Simulation experiments

Overview of data generating process

To better understand the performance of VaDER we conducted an extensive simulation study: We simulated multivariate short time series via vector autoregressive (VAR) processes [63], because (1) they can model the auto-correlation between time points, (2) they can model the cross-correlation between variables and (3) given a VAR, one can generate random trajectories from that VAR.

We used mixtures of VAR processes to simulated multivariate time series data of the same dimensions as the ADNI data: 4 variables measured over 8 time points. Given a clusterability factor λ, we generated trajectories as follows:

i. Sample coefficient matrices for 3 VAR(8) processes, by randomly sampling the individual entries of each 4×4 matrix from the uniform distribution $\mathcal{U}(-.1, .1)$. Multiply each of the matrix entries by λ.
ii. Randomly sample 3 additional 4×4 matrices from $\mathcal{U}(-.1, .1)$, and multiply each with its own transpose. Let each of results correspond to the variance-covariance matrix of one of the 3 VAR(8) processes.
iii. Repeat 10^3 times:
    i. Randomly select one of the 3 VAR(8) processes (with equal probability).
    ii. Generate a random trajectory from the selected VAR(8) process.

The above generates one set of random data. Given a value of λ, the entire sampling process was repeated 100 times, and each of the 100 datasets was clustered using both VaDER and hierarchical clustering.

For computational reasons, hyper-parameters for VaDER were fixed and not further optimized during our simulation (10^2 epochs of both pre-training and training, learning rate: 10^{-4}, two hidden layers: [36, 4], batch size: 64).

Comparison against hierarchical clustering

We compared VaDER against a conventional hierarchical clustering (complete linkage), in which we flatten the $N \times M$ data matrices of each patient into vectors. We considered three distance measures for these vectors:

- Pearson correlation
- Euclidean distance
- Short time series (STS) distance [37], a distance measure specifically developed for univariate short time series. The STS distance relies on the difference between adjacent time points. Here we first computed the STS distance for each of the different clinical outcome measures, and then summed these up to arrive at an aggregated STS distance across the M clinical measures.

Additionally, we compared VaDER against hierarhical clustering using two distance measures specifically designed for multivariate time series:

- Multidimensional dynamic time warping [38]
- Fast global alignment kernels [39]

Given that VaDER is non-deterministic, we ran 100 replicates for each (simulated / benchmark) dataset, and determined the consensus clustering by hierarchically clustering a consensus matrix listing for each pair of samples how often these two samples were clustered together across the 100 replicates.

Simulating missing data

To test the ability of VaDER to deal with missing data we performed a separate set of simulations: Let L be the number of patients in our dataset, and $\mathbf{x}^l \in \mathbb{R}^{N\times M}$ a single patient trajectory ($l \in 1 \ldots L$), where N is the number of time points and M is the number of measured features. Missing values completely at random (MCAR) were simulated by an individual entry $\mathbf{x}^l_{ij}$ to missing with probability θ.

Missing not at random (MNAR) was simulated by letting the probability of a missing value for entry $\mathbf{x}^l_{ij}$ depend on time. More specifically, each individual entry $\mathbf{x}^l_{ij}$ was set to missing with probability $\frac{1}{1+e^{i_0-i/k}}$, where $i_0 = \frac{1+N}{2}$, where $i_0 = \frac{1+N}{2}$, and k was varied to result in different overall missingness fractions *theta*.

To compare VADER's implicit imputation with pre-imputation, missing values generated using the above approach were additionally imputed using mean substitution: Each missing value was substituted with the average conditioned on the relevant time point and variable.

Given that VaDER is non-deterministic, we ran 20 replicates for each (simulated / benchmark) dataset, and determined the consensus clustering by hierarchically clustering a consensus matrix listing for each pair of samples how often these two samples were clustered together across the 20 replicates. Confidence intervals were determined by repeating the above procedure 100 times (simulation experiments) or 5 times (benchmark experiments) with newly generated missingness patterns (simulation/benchmark experiments) and/or data (simulation experiments).

Estimating clustering performance

For the simulation and benchmark datasets, the number of clusters is a priori known. Hence, an intuitive measure of comparing the performance between the different algorithms is cluster purity [36]. Cluster purity can be interpreted as the fraction of correctly clustered samples and is calculated as follows:

i. For each cluster, count the number of samples from the majority class in that cluster.
ii. Sum the above counts.
iii. Divide by the total number of samples.

For the ADNI and PPMI data, the number of clusters is not a priori known. Hence, performance was recorded using the adjusted rand index [64, 65] for different values of λ in the interval $[0.001, 0.25]$. For $\lambda \gtrapprox 0.25$, generating coefficient matrices that lead to stable VARs becomes very difficult.

Post-hoc analysis of patient clusters

We collected a wide range of additional variables from ADNI and PPMI, and assessed the association of the identified patient subgroups with a given variable by multinomial logistic regression. For any baseline variable x, we first fitted the following full model:

$$\text{subgroup} \sim x + \text{confounders} \quad (6)$$

Each of these full models were then compared to a null model:

$$\text{subgroup} \sim \text{confounders} \quad (7)$$

by means of a likelihood ratio test.

For any longitudinal variable x measured at timepoints t, we first fitted the following multinomial logistic regression model:

$$\text{subgroup} \sim x + t + x * t + \text{confounders} \quad (8)$$

We tested this model against the null model:

$$\text{subgroup} \sim \text{confounders} \quad (9)$$

by performing a likelihood ratio test, and applying an FDR correction for multiple testing. If the above test was found to be significant ($q < 0.05$), we tested the effects of the individual terms $x * t$, x and t against the same null model above.

Confounders considered were age, education and gender, but were only included when univariate significantly associated with subgroup. For ADNI, this was only age ($p = 0.0029$, ANOVA F-test). For PPMI, this was only gender ($p = 0.0017$, χ^2-test).

In the post-hoc analysis, only complete cases were included, i.e. patients with missing values were ignored.

Availability of supporting source code and requirements

A complete implementation of VaDER in Python/Tensorflow: https://github.com/johanndejong/VaDER.

An R-package for streamlining the processing of PPMI data: https://github.com/patzaw/PPMI-R-package-generator.

Other code used for generating results presented in this paper: https://github.com/johanndejong/VaDER_supporting_code.

Snapshots of all the above code and other supporting data are also available in the GigaScience database, GigaDB [66].

List of abbreviations

- AD: Alzheimer's disease
- ADAS-13: The Alzheimer's disease assessment scale
- ADNI: Alzheimer's Disease Neuroimaging Initiative
- CDRSB: The clinical dementia rating sum of box score.
- CSF: cerebrospinal fluid
- ESS: Epworth sleepiness scale.
- FAQ: The functional activities questionnaire.
- LSTM: long short term memory
- MAR: missing at random
- MCAR: missing completely at random
- MMSE: mini-mental state examination
- MNAR: missing not at random
- PD: Parkinson's disease
- PIGD: postural instability and gait disturbance.
- PPMI: Parkinson's Progression Markers Initiative
- RBD: REM sleep behavior disorder.
- SCOPA: scales for outcomes in Parkinson's disease.
- TD: tremor-dominant
- UPDRS: Unified Parkinson's disease rating scale.
- UPDRS1: Unified Parkinson's disease rating scale, part 1.
- UPDRS2: Unified Parkinson's disease rating scale, part 2.
- UPDRS3: Unified Parkinson's disease rating scale, part 3.
- UCI: The University of California Irvine machine learning repository.
- UEA/UCR: The University of East Anglia / University of California, Riverside time series classification archive.
- VaDE: variational deep embedding
- VaDER: variational deep embedding with recurrence
- VAR: vector auto regression
- VCF: variant call format

Competing interests

JdJ and HF received salaries from UCB Biosciences GmbH. UCB Biosciences GmbH had no influence on the content of this work.

Funding

The research leading to these results has received partial support from the Innovative Medicines Initiative Joint Undertaking under grant agreement #115568, resources of which are composed of financial contribution from the European Union's Seventh Framework Programme (FP7/2007-2013) and EFPIA companies' in kind contribution.

Authors' contributions

Method development: JdJ, HF; implementation and testing: JdJ; Data preparation: MAE, PW, RK, MS, AA; image analysis: HV; supervision: HF, MHA; definition of research project: HF

Acknowledgements

Data collection and sharing for this project was funded by the Alzheimer's Disease Neuroimaging Initiative (ADNI) (National Institutes of Health Grant U01 AG024904) and DOD ADNI (Department of Defense award number W81XWH-12-2-0012). ADNI is funded by the National Institute on Aging, the National Institute of Biomedical Imaging and Bioengineering, and through generous contributions from the following: AbbVie, Alzheimer's Association; Alzheimer's Drug Discovery Foundation; Araclon Biotech; BioClinica, Inc.; Biogen; Bristol-Myers

Squibb Company; CereSpir, Inc.; Cogstate; Eisai Inc.; Elan Pharmaceuticals, Inc.; Eli Lilly and Company; EuroImmun; F. Hoffmann-La Roche Ltd and its affiliated company Genentech, Inc.; Fujirebio; GE Healthcare; IXICO Ltd.; Janssen Alzheimer Immunotherapy Research & Development, LLC.; Johnson & Johnson Pharmaceutical Research & Development LLC.; Lumosity; Lundbeck; Merck & Co., Inc.; Meso Scale Diagnostics, LLC.; NeuroRx Research; Neurotrack Technologies; Novartis Pharmaceuticals Corporation; Pfizer Inc.; Piramal Imaging; Servier; Takeda Pharmaceutical Company; and Transition Therapeutics. The Canadian Institutes of Health Research is providing funds to support ADNI clinical sites in Canada. Private sector contributions are facilitated by the Foundation for the National Institutes of Health (www.fnih.org). The grantee organization is the Northern California Institute for Research and Education, and the study is coordinated by the Alzheimer's Therapeutic Research Institute at the University of Southern California. ADNI data are disseminated by the Laboratory for Neuro Imaging at the University of Southern California.

Data used in the preparation of this article were obtained from the Parkinson's Progression Markers Initiative (PPMI) database (www.ppmi-info.org/data). For up-todate information on the study, visit www.ppmi-info.org. PPMI – a public-private partnership – is funded by the Michael J. Fox Foundation for Parkinson's Research and funding partners. A list of names of all of the PPMI funding partners can be found at www.ppmi-info.org/about-ppmi/who-we-are/study-sponsors/.

References

1. Hruby A, Hu FB. The Epidemiology of Obesity: A Big Picture. Pharmacoeconomics 2015 Jul;33(7):673–689. https://www.ncbi.nlm.nih.gov/pubmed/25471927, 25471927[pmid].
2. van Tilburg J, van Haeften TW, Pearson P, Wijmenga C. Defining the genetic contribution of type 2 diabetes mellitus. Journal of Medical Genetics 2001;38(9):569–578. https://jmg.bmj.com/content/38/9/569.
3. Cordell HJ, Todd JA. Multifactorial inheritance in type 1 diabetes. Trends in Genetics 1995;11(12):499 – 504. http://www.sciencedirect.com/science/article/pii/S016895250089160X.
4. Ruppert V, Maisch B. Genetics of HumanHypertension. Herz 2003 Dec;28(8):655–662. https://doi.org/10.1007/s00059-003-2516-6.
5. Poulter N. Coronary heart disease is a multifactorial disease. American Journal of Hypertension 1999;12(10, Supplement 1):92S – 95S. http://www.sciencedirect.com/science/article/pii/S0895706199001636.
6. Hastie T, Tibshirani R, Friedman JH. The elements of statistical learning: data mining, inference, and prediction, 2nd Edition. Springer series in statistics, Springer; 2009. http://www.worldcat.org/oclc/300478243.
7. Kannan R, Vempala S. On Clusterings - Good, Bad and Spectral. In: Proc. Symp. Found. Comp. Sci.; 2000. p. 367–377.
8. Jain A, Dubes R. Algorithms for Clustering Data. Englewood Cliffs, NJ: Prentice-Hall; 1988.
9. Fukunaga K, Hostetler LD. The estimation of the gradient of a density function, with applications in pattern recognition. IEEE Transactions on Information Theory 1975;21:32–39.
10. Aghabozorgi S, Seyed Shirkhorshidi A, Ying Wah T. Time-series Clustering - A Decade Review. Inf Syst 2015 Oct;53(C):16–38. http://dx.doi.org/10.1016/j.is.2015.04.007.
11. Rani S, Sikka G. Article: Recent Techniques of Clustering of Time Series Data: A Survey. International Journal of Computer Applications 2012 August;52(15):1–9. Full text available.
12. Warren Liao T. Clustering of time series data—a survey. Pattern Recognition 2005;38(11):1857–1874.
13. Ghassempour S, Girosi F, Maeder A. Clustering Multivariate Time Series Using Hidden Markov Models. International Journal of Environmental Research and Public Health 2014;11(3):2741–2763. http://www.mdpi.com/1660-4601/11/3/2741.
14. Sun J. Clustering multivariate time series based on Riemannian manifold. Electronics Letters 2016 September;52:1607–1609(2). https://digital-library.theiet.org/content/journals/10.1049/el.2016.0701.
15. Rubin DB. Inference and Missing Data. Biometrika 1976;63(3):581–592. http://www.jstor.org/stable/2335739.
16. Kang H. The prevention and handling of the missing data. Korean Journal of Anesthesiology 2013 May;64(5):402–406. https://www.ncbi.nlm.nih.gov/pmc/articles/PMC3668100/.
17. Mikolov T, Sutskever I, Chen K, Corrado G, Dean J. Distributed Representations of Words and Phrases and Their Compositionality. In: Proceedings of the 26th International Conference on Neural Information Processing Systems - Volume 2 NIPS'13, USA: Curran Associates Inc.; 2013. p. 3111–3119. http://dl.acm.org/citation.cfm?id=2999792.2999959.
18. Hinton GE, Salakhutdinov RR. Reducing the Dimensionality of Data with Neural Networks. Science 2006;313(5786):504–507. http://science.sciencemag.org/content/313/5786/504.
19. Frome A, Corrado GS, Shlens J, Bengio S, Dean J, Ranzato MA, et al. DeViSE: A Deep Visual-Semantic Embedding Model. In: Burges CJC, Bottou L, Welling M, Ghahramani Z, Weinberger KQ, editors. Advances in Neural Information Processing Systems 26 Curran Associates, Inc.; 2013.p. 2121–2129. http://papers.nips.cc/paper/5204-devise-a-deep-visual-semantic-embedding-model.pdf.
20. Asgari E, Mofrad MRK. Continuous Distributed Representation of Biological Sequences for Deep Proteomics and Genomics. PLOS ONE 2015 11;10(11):1–15. https://doi.org/10.1371/journal.pone.0141287.
21. Trigeorgis G, Bousmalis K, Zafeiriou S, Schuller B. A Deep Semi-NMF Model for Learning Hidden Representations. In: Xing EP, Jebara T, editors. Proceedings of the 31st International Conference on Machine Learning, vol. 32 of Proceedings of Machine Learning Research Bejing, China: PMLR; 2014. p. 1692–1700. http://proceedings.mlr.press/v32/trigeorgis14.html.
22. Xie J, Girshick R, Farhadi A. Unsupervised Deep Embedding for Clustering Analysis. In: Proceedings of the 33rd International Conference on International Conference on Machine Learning - Volume 48 ICML'16, JMLR.org; 2016. p. 478–487. http://dl.acm.org/citation.cfm?id=3045390.3045442.
23. Jiang Z, Zheng Y, Tan H, Tang B, Zhou H. Variational Deep Embedding: An Unsupervised and Generative Approach to Clustering. In: IJCAI ijcai.org; 2017. p. 1965–1972.
24. Hochreiter S, Schmidhuber J. Long Short-Term Memory. Neural Comput 1997 Nov;9(8):1735–1780. http://dx.doi.org/10.1162/neco.1997.9.8.1735.
25. Petersen RC, Aisen PS, Beckett LA, Donohue MC, Gamst AC, Harvey DJ, et al. Alzheimer's Disease Neuroimaging Initiative (ADNI). Neurology 2010;74(3):201–209. http://n.neurology.org/content/74/3/201.

26. Marek K, Jennings D, Lasch S, Siderowf A, Tanner C, Simuni T, et al. The Parkinson Progression Marker Initiative (PPMI). Progress in Neurobiology 2011 12;95(4):629–635.
27. Komarova NL, Thalhauser CJ. High Degree of Heterogeneity in Alzheimer's Disease Progression Patterns. PLoS Computational Biology 2011;7(11). https://doi.org/10.1371/journal.pcbi.1002251.
28. Lam B, Masellis M, Freedman M, Stuss DT, Black SE. Clinical, imaging, and pathological heterogeneity of the Alzheimer's disease syndrome. Alzheimer's Research & Therapy 2013 Jan;5(1):1. https://doi.org/10.1186/alzrt155.
29. Lewis SJG, Foltynie T, Blackwell AD, Robbins TW, Owen AM, Barker RA. Heterogeneity of Parkinson's disease in the early clinical stages using a data driven approach. Journal of Neurology, Neurosurgery & Psychiatry 2005;76(3):343–348. https://jnnp.bmj.com/content/76/3/343.
30. von Coelln R, Shulman LM. Clinical subtypes and genetic heterogeneity: of lumping and splitting in Parkinson disease. Current Opinion in Neurology 2016;29(6). https://journals.lww.com/co-neurology/Fulltext/2016/12000/Clinical_subtypes_and_genetic_heterogeneity___of.10.aspx.
31. Kingma DP, Welling M, Auto-Encoding Variational Bayes; 2013. http://arxiv.org/abs/1312.6114, cite arxiv:1312.6114.
32. Doersch C, Tutorial on Variational Autoencoders; 2016. http://arxiv.org/abs/1606.05908, cite arxiv:1606.05908.
33. Gers FA, Schraudolph NN, Schmidhuber J. Learning Precise Timing with Lstm Recurrent Networks. J Mach Learn Res 2003 Mar;3:115–143. https://doi.org/10.1162/153244303768966139.
34. Lipton ZC, Kale DC, Wetzel RC. Directly Modeling Missing Data in Sequences with RNNs: Improved Classification of Clinical Time Series. In: MLHC, vol. 56 of JMLR Workshop and Conference Proceedings JMLR.org; 2016. p. 253–270.
35. Nazábal A, Olmos PM, Ghahramani Z, Valera I. Handling Incomplete Heterogeneous Data using VAEs. CoRR 2018;abs/1807.03653. http://arxiv.org/abs/1807.03653.
36. Manning CD, Raghavan P, Schütze H. Introduction to Information Retrieval. New York, NY, USA: Cambridge University Press; 2008.
37. Möller-Levet CS, Klawonn F, Cho K, Wolkenhauer O. Fuzzy Clustering of Short Time-Series and Unevenly Distributed Sampling Points. In: Berthold MR, Lenz H, Bradley E, Kruse R, Borgelt C, editors. Advances in Intelligent Data Analysis V, 5th International Symposium on Intelligent Data Analysis, IDA 2003, Berlin, Germany, August 28-30, 2003, Proceedings, vol. 2810 of Lecture Notes in Computer Science Springer; 2003. p. 330–340. https://doi.org/10.1007/978-3-540-45231-7_31.
38. Tormene P, Giorgino T, Quaglini S, Stefanelli M. Matching Incomplete Time Series with Dynamic Time Warping: An Algorithm and an Application to Post-Stroke Rehabilitation. Artificial Intelligence in Medicine 2008;45(1):11–34. http://dx.doi.org/10.1016/j.artmed.2008.11.007.
39. Cuturi M. Fast Global Alignment Kernels. In: Getoor L, Scheffer T, editors. ICML Omnipress; 2011. p. 929–936. http://dblp.uni-trier.de/db/conf/icml/icml2011.html#Cuturi11.
40. Dua D, Graff C, UCI Machine Learning Repository; 2017. http://archive.ics.uci.edu/ml.
41. Bagnall A, Lines J, Vickers W, The UEA and UCR Time Series Classification Repository;. Accessed: 2019-08-15. http://www.timeseriesclassification.com.
42. Tibshirani R, Walther G. Cluster validation by prediction strength. Journal of Computational and Graphical Statistics 2005;14(3):511–528.
43. Tibshirani R, Walther G, Hastie T. Estimating the number of clusters in a data set via the gap statistic. Journal of the Royal Statistical Society: Series B (Statistical Methodology) 2001;63(2):411–423. https://rss.onlinelibrary.wiley.com/doi/abs/10.1111/1467-9868.00293.
44. Sugar CA, James GM. Finding the Number of Clusters in a Dataset: An Information-Theoretic Approach. Journal of the American Statistical Association 2003;98(463):750–763. http://www.jstor.org/stable/30045303.
45. Thorndike RL. Who belongs in the family. Psychometrika 1953;p. 267–276.
46. Rousseeuw PJ. Silhouettes: A graphical aid to the interpretation and validation of cluster analysis. Journal of Computational and Applied Mathematics 1987;20:53 – 65. http://www.sciencedirect.com/science/article/pii/0377042787901257.
47. Convit A, de Asis J, de Leon MJ, Tarshish CY, Santi SD, Rusinek H. Atrophy of the medial occipitotemporal, inferior, and middle temporal gyri in non-demented elderly predict decline to Alzheimer's disease. Neurobiology of Aging 2000;21(1):19 – 26. http://www.sciencedirect.com/science/article/pii/S0197458099001074.
48. Nestor SM, Rupsingh R, Borrie M, Smith M, Accomazzi V, Wells JL, et al. Ventricular enlargement as a possible measure of Alzheimer's disease progression validated using the Alzheimer's disease neuroimaging initiative database. Brain 2008 07;131(9):2443–2454. https://doi.org/10.1093/brain/awn146.
49. Butterfield DA, Halliwell B. Oxidative stress, dysfunctional glucose metabolism and Alzheimer disease. Nature Reviews Neuroscience 2019;20(3):148–160. https://doi.org/10.1038/s41583-019-0132-6.
50. Tapiola T, Alafuzoff I, Herukka SK, Parkkinen L, Hartikainen P, Soininen H, et al. Cerebrospinal Fluid Beta-Amyloid 42 and Tau Proteins as Biomarkers of Alzheimer-Type Pathologic Changes in the Brain. JAMA Neurology 2009 03;66(3):382–389. https://doi.org/10.1001/archneurol.2008.596.
51. Moisan F, Kab S, Mohamed F, Canonico M, Le Guern M, Quintin C, et al. Parkinson disease male-to-female ratios increase with age: French nationwide study and meta-analysis. Journal of Neurology, Neurosurgery & Psychiatry 2016;87(9):952–957. https://jnnp.bmj.com/content/87/9/952.
52. Schrag A, Jahanshahi M, Quinn N. What contributes to quality of life in patients with Parkinson's disease? Journal of Neurology, Neurosurgery & Psychiatry 2000;69(3):308–312. https://jnnp.bmj.com/content/69/3/308.
53. Sheikh JI, Yesavage JA. Geriatric Depression Scale (GDS): Recent evidence and development of a shorter version. Clinical Gerontologist: The Journal of Aging and Mental Health 1986;5(1-2):165–173.
54. Marsh L. Depression and Parkinson's disease: current knowledge. Curr Neurol Neurosci Rep 2013 Dec;13(12):409–409. https://www.ncbi.nlm.nih.gov/pubmed/2419078, 24190780[pmid].
55. Pitcher TL, Melzer TR, MacAskill MR, Graham CF, Livingston L, Keenan RJ, et al. Reduced striatal volumes in Parkinson's disease: a magnetic resonance imaging study. Translational Neurodegeneration 2012 Aug;1(1):17. https://doi.org/10.1186/2047-9158-1-17.
56. Pedersen AB, Mikkelsen EM, Cronin-Fenton D, Kristensen NR, Pham TM, Pedersen L, et al. Missing data and multiple imputation in clinical epidemiological research. Clin Epidemiol 2017 Mar;9:157–166. https://www.ncbi.nlm.nih.

gov/pubmed/28352203, 28352203[pmid].
57. Marston L, Carpenter JR, Walters KR, Morris RW, Nazareth I, Petersen I. Issues in multiple imputation of missing data for large general practice clinical databases. Pharmacoepidemiology and Drug Safety 2010;19(6):618–626. https://onlinelibrary.wiley.com/doi/abs/10.1002/pds.1934.
58. the ADNI team. ADNIMERGE: Alzheimer's Disease Neuroimaging Initiative; 2018, r package version 0.0.1.
59. Desikan R, Ségonne F, Fischl B, Quinn BT, Dickerson BC, Blacker D, et al. An automated labeling system for subdividing the human cerebral cortex on MRI scans into gyral based regions of interest. NeuroImage 2006;31(3):968–980. https://doi.org/10.1016/j.neuroimage.2006.01.021.
60. Destrieux C, Fischl B, Dale AM, Halgren E. Automatic parcellation of human cortical gyri and sulci using standard anatomical nomenclature. NeuroImage 2010;53(1):1–15. http://dblp.uni-trier.de/db/journals/neuroimage/neuroimage53.html#DestrieuxFDH10.
61. Wang L, Wang Z, Liu S. An Effective Multivariate Time Series Classification Approach Using Echo State Network and Adaptive Differential Evolution Algorithm. Expert Syst Appl 2016 Jan;43(C):237–249. https://doi.org/10.1016/j.eswa.2015.08.055.
62. Øyvind Mikalsen K, Bianchi FM, Soguero-Ruiz C, Jenssen R. Time series cluster kernel for learning similarities between multivariate time series with missing data. Pattern Recognition 2018;76:569 – 581. http://www.sciencedirect.com/science/article/pii/S0031320317304843.
63. Sims C. Macroeconomics and Reality. Econometrica 1980;48(1):1–48. https://EconPapers.repec.org/RePEc:ecm:emetrp:v:48:y:1980:i:1:p:1-48.
64. Rand WM. Objective criteria for the evaluation of clustering methods. Journal of the American Statistical Association 1971;66(336):846–850.
65. Hubert L, Arabie P. Comparing partitions. Journal of classification 1985;2(1):193–218. http://scholar.google.de/scholar.bib?q=info:IkrWWF2JxwoJ:scholar.google.com/&output=citation&hl=de&ct=citation&cd=0.
66. de Jong J, Emon MA, Wu P, Karki R, Sood M, Godard P, et al. Supporting data for "Deep learning for clustering of multivariate clinical patient trajectories with missing values". GigaScience Database 2019;.

Manuscript with DOI
Click here to view linked References
Click here to access/download;Manuscript;VaDER_GigaScience__manuscri

GigaScience, 2017, 1–14

doi: xx.xxxx/xxxx
Manuscript in Preparation
Paper

PAPER

Deep learning for clustering of multivariate clinical patient trajectories with missing values

Johann de Jong[1,*], Mohammad Asif Emon[2,3], Ping Wu[4], Reagon Karki[2,3], Meemansa Sood [2,3], Patrice Godard[6], Ashar Ahmad[3], Henri Vrooman[5], Martin Hofmann-Apitius[2,3] and Holger Fröhlich[1,3,*]

[1]UCB Biosciences GmbH, 40789 Monheim, Germany and [2]Fraunhofer Institute for Algorithms and Scientific Computing, 53754 Sankt Augustin, Germany and [3]Bonn-Aachen International Center for IT, University of Bonn, 53115 Bonn, Germany and [4]UCB Pharma, Slough SL1 3WE, United Kingdom and [5]Erasmus MC, University Medical Center Rotterdam, Departments of Radiology and Medical Informatics, PO Box 2040 3000 CA Rotterdam, Netherlands and [6]UCB Pharma, 1420 Braine-l'Alleud, Belgium

[*]johann.dejong@ucb.com; holger.froehlich@ucb.com; frohlich@bit.uni-bonn.de

Abstract

Background. Precision medicine requires a stratification of patients by disease presentation that is sufficiently informative to allow for selecting treatments on a per-patient basis. For many diseases, such as neurological disorders, this stratification problem translates into a complex problem of clustering multivariate and relatively short time series, because (1) these diseases are multifactorial and not well described by single clinical outcome variables, and (2) disease progression needs to be monitored over time. Additionally, clinical often additionally suffer from the presence of many missing values, further complicating any clustering attempts.
Findings. The problem of clustering multivariate short time series with many missing values is generally not well addressed in the literature so far. In this work, we propose a deep learning-based method to address this issue, variational deep embedding with recurrence (VaDER). VaDER relies on a Gaussian mixture variational autoencoder framework, which is further extended to (1) model multivariate time series and (2) directly deal with missing values. We validated VaDER by accurately recovering clusters from simulated and benchmark data with known ground truth clustering, while varying the degree of missingness. We then used VaDER to successfully stratify Alzheimer's disease (AD) patients and Parkinson's disease (PD) patients into subgroups characterized by clinically divergent disease progression profiles. Additional analyses demonstrated that these clinical differences reflected known underlying aspects of AD and PD.
Conclusions. We believe our results show that VaDER can be of great value for future efforts in patient stratification, and multivariate short time series clustering in general.

Key words: Patient stratification; deep learning; multivariate short time series; multivariate longitudinal data; clustering

Findings

Background

In precision medicine, patients are stratified based on their disease subtype, risk, prognosis, or treatment response using specialized diagnostic tests. An important question in precision medicine, is how to appropriately model disease progression and accordingly decide for the right type and time point of therapy for an individual. However, the progression of many diseases, such as neurological disorders, cardiovascular diseases, diabetes, obesity [1, 2, 3, 4, 5], is highly multifaceted and not

Compiled on: October 19, 2019.
Draft manuscript prepared by the author.

well described by one clinical outcome measure alone. Classical univariate clustering methods are likely to miss the inherent complexity of diseases that demonstrate a highly multifaceted clinical phenotype. Accordingly, stratification of patients by disease progression translates into the challenging question of how to identify a clustering of a multivariate time series.

Clustering is a fundamental and generally well investigated problem in machine learning and statistics. Its goal is to segment samples into groups (clusters), such that there is a higher degree of similarity between samples of the same cluster than between samples of different clusters. Following [6], algorithms to solve clustering problems may be put into three main categories, (1) combinatorial algorithms, (2) mixture modeling and (3) mode seeking. Within each of these three categories, a wide range of methods is available for a great diversity of clustering problems. Combinatorial algorithms do not assume any underlying probability model, but work with the data directly. Examples are K-means clustering, spectral clustering [7] and hierarchical clustering [8]. Mixture models assume that the data can be described by some probabilistic model. An example is Gaussian mixture model clustering. Finally, in mode seeking one tries to directly estimate modes of the underlying multi-modal probability density. An important example here is the mean-shift algorithm [9].

For the clustering of multivariate time series data, a few techniques have been developed [10, 11, 12, 13, 14]. However, these approaches generally rely on time series of far greater length than available in most longitudinal clinical datasets. Moreover, these methods are not suited for the large numbers of missing values often found in clinical data.

Missing values in clinical data can occur due to different reasons: (1) patients drop out of a study, e.g. due to worsening of symptoms; (2) a certain diagnostic test is not taken at a particular visit (e.g. due to lack of patient agreement), potentially resulting into missing information for entire variable groups; (3) unclear further reasons, e.g. time constraints, data quality issues, etc. From a statistical point of view, these reasons manifest into different mechanisms of missing data [15, 16]:

i. Missing completely at random (MCAR): The probability of missing information is neither related to the specific value which is supposed to be obtained, nor to other observed data. Hence, entire patient records could be skipped without introducing any bias. However, this type of missing data mechanism is probably rare in clinical studies.
ii. Missing at random (MAR): The probability of missing information depends on other observed data, but is not related to the specific missing value that is expected to be obtained. An example would be patient drop out due the to worsening of certain symptoms, which are at the same time recorded during the study.
iii. Missing not at random (MNAR): any reason for missing data, which is neither MCAR nor MAR. MNAR is problematic, because the only way to obtain unbiased estimates is to model missing data.

Multiple imputation methods have been proposed to deal with missing values in longitudinal patient data [16]. However, any imputation method will result in certain errors, and if imputation and clustering are done separately, these errors will propagate through to the following clustering procedure.

To address the problem of clustering multivariate and relatively short time series data with many missing values, in this paper we propose an approach that uses techniques from deep learning. Autoencoder networks have been highly successful in learning latent representations of data, e.g. [17, 18, 19, 20]. Specifically for clustering, autoencoders can be first used to learn a latent representation of a multivariate distribution, to then independently find clusters [21]. More recently, some authors have suggested to simultaneously learn latent representations and cluster assignments. Interesting examples are deep embedded clustering (DEC) [22] and variational deep embedding (VaDE) [23].

Here, we present a new method for clustering multivariate short time series with potentially many missing values, VaDER (variational deep embedding with recurrence). VaDER is in part based on VaDE [23], a clustering algorithm based on variational autoencoder principles, with a latent representation forced towards a multivariate Gaussian mixture distribution. Additionally, VaDER (1) integrates two LSTM networks [24] into its architecture, to allow for the analysis of multivariate short time series, and (2) adopts an approach of implicit imputation and loss re-weighting to account for the typically high degree of missingness in clinical data.

After a validation of VaDER via simulation and benchmark studies, we applied the method to the problem of patient stratification in Alzheimer's disease (AD) and Parkinson's disease (PD), using data from the Alzheimer's Disease Neuroimaging Initiative (ADNI) [25] and the Parkinson's Progression Markers Initiative (PPMI) [26] respectively. Alzheimer's and Parkinson's disease are multifactorial and highly heterogeneous diseases, both in clinical and biological presentation, as well as in progression [27, 28, 29, 30]. For example, PD is characterized by motor symptoms, behavioral changes (e.g. sleeping disorders) as well as cognitive impairment [1]. Cognitive impairment, one of the hallmarks of AD, is not straightforward to assess, since cognition itself is highly multifaceted, and described by e.g. orientation, speech and memory. Consequently, in the field of AD, a wide range of tests have been developed to assess different aspects of cognition.

This heterogeneity presents one of the major challenges in understanding these diseases and developing new treatments. As such, better clustering (stratification) of patients by disease presentation could be of great help in improving disease management and designing better clinical trials that specifically focus on treating patients that are rapidly progressing.

Our analyses of the ADNI and PPMI data show that VaDER is highly effective at disentangling multivariate patient trajectories into clinically meaningful patient subgroups.

Results

Variational autoencoders for clustering

Our proposed variational deep embedding with recurrence (VaDER) method is in part based on variational deep embedding (VaDE) [23], a variational autoencoding clustering algorithm with a multivariate Gaussian mixture prior. In variational autoencoding algorithms, the training objective is to optimize the variational lower bound on the marginal likelihood of a data point $\mathbf{x}$ [31]:

$$\mathcal{L}(\mathbf{x}) = \mathbb{E}_{q(\mathbf{z}|\mathbf{x})}[\log(p(\mathbf{x}|\mathbf{z}))] - D_{KL}(q(\mathbf{z}|\mathbf{x})||p(\mathbf{z})) \quad (1)$$

This lower bound can be seen as composed of two parts. The first term corresponds to the likelihood of seeing $\mathbf{x}$ given a latent representation $\mathbf{z}$. Its negative is often called the *reconstruction loss*, and it forces the algorithm to learn good reconstructions of its input data. The negative of the second term is often called the *latent loss*. It is the Kullback-Leibler divergence of the prior $p(\mathbf{z})$ to the variational posterior $q(\mathbf{z}|\mathbf{x})$, and

1 https://www.ninds.nih.gov/Disorders/All-Disorders/Parkinsons-Disease-Information-Page

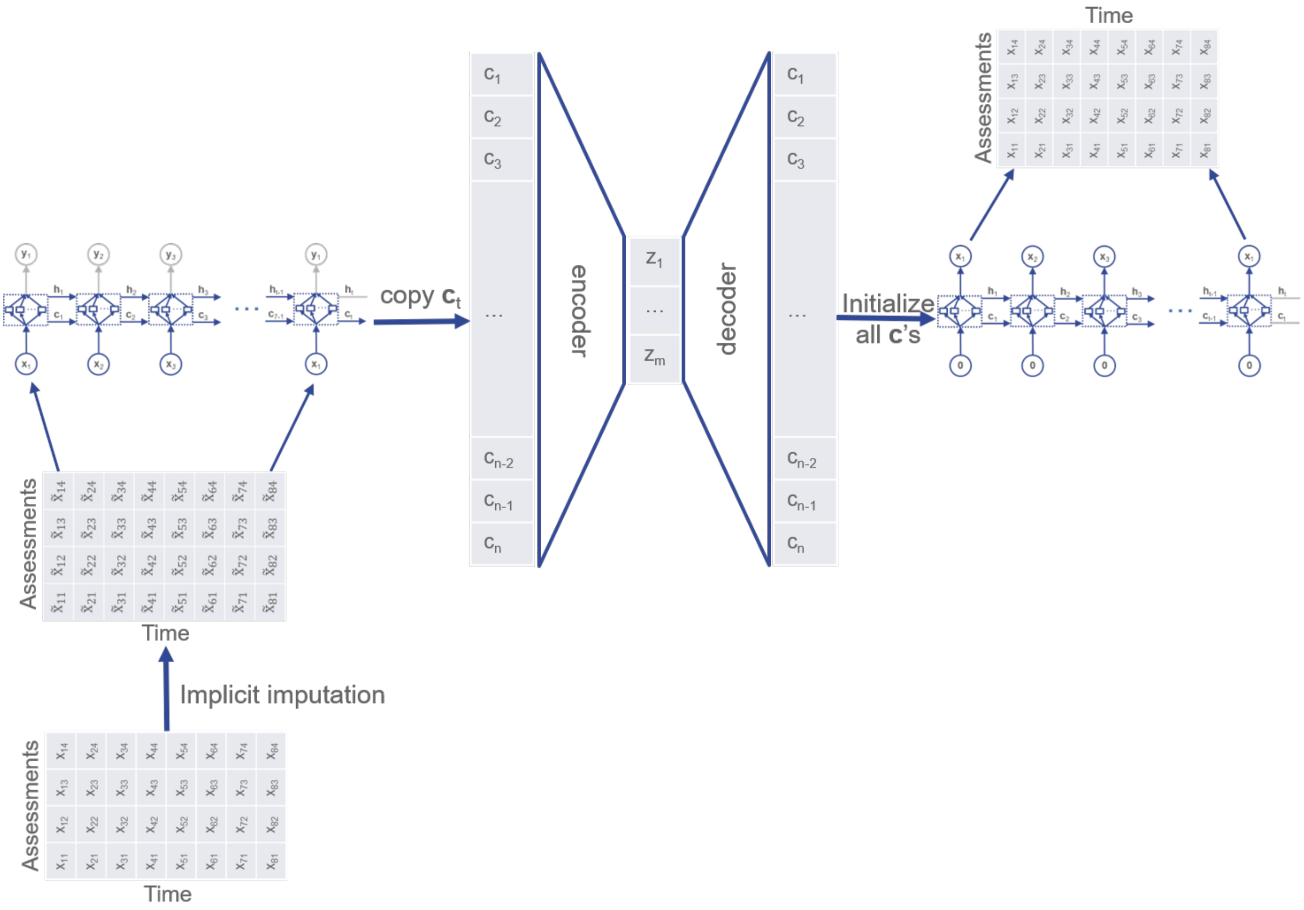

Figure 1. VaDER architecture

it regularizes the latent representation $\mathbf{z}$ to lie on a manifold specified by the prior $p(\mathbf{z})$.

In VaDE, this prior is a multivariate Gaussian mixture. Accordingly including a parameter for choosing a cluster c, the variational lower bound can then be written as follows:

$$\mathcal{L}(\mathbf{x}) = \mathbb{E}_{q(\mathbf{z},c|\mathbf{x})}[\log(p(\mathbf{x}|\mathbf{z}))] - D_{KL}(q(\mathbf{z},c|\mathbf{x})||p(\mathbf{z},c)) \quad (2)$$

By forcing the latent representation $\mathbf{z}$ towards a multivariate Gaussian mixture distribution, VaDE has the ability to simultaneously learn latent representations and cluster assignments of its input data. For more details on variational autoencoders and VaDE, we refer the reader to [32, 31, 23].

Variational deep embedding with recurrence (VaDER)

VaDER is an autoencoder-based method for clustering multivariate short time series with potentially many missing values. For simultaneously learning latent representations and cluster assignments of its input samples, VaDER uses the VaDE latent loss as described above and in [23].

To model the auto- and cross-correlations in multivariate short time series data, we integrate peephole LSTM networks [24, 33] into the autoencoder architecture (Figure 1).

To deal with missing values, we directly integrate imputation into model training. As outlined in Section Background, separating imputation from clustering can potentially introduce bias. To avoid this bias, we here propose an implicit imputation scheme, which is performed within VaDER training. Our approach to imputation bears some similarity to other approaches [34, 35]. However, in contrast to [34], VaDER uses missingness indicators for implicit imputation as an integral part of neural network training. Additionally, in contrast to [35], our method of imputation is also suited for MNAR data, which are often encountered in clinical datasets.

We first define a weighted reconstruction loss on feature and sample level: Imputed values are weighted to 0, non-imputed values are weighted to 1. To retain the balance with the latent loss, the resulting reconstruction loss is re-scaled to match the original dimensions of the data. More formally, for a mean squared reconstruction loss, let L be the number of samples in our dataset, $\mathbf{x}^l$ a single input sample, and $\hat{\mathbf{x}}^l$ its corresponding reconstructed output ($l \in 1 \ldots L$). $\mathbf{x}^l$ and $\hat{\mathbf{x}}^l$ are matrices $\in \mathbb{R}^{N \times M}$, where N is the number of time points and M is the number of clinical outcome measures (e.g. cognitive assessments) for a particular patient. Then the unweighted mean reconstruction loss is:

$$\frac{1}{L}\sum_{l=1}^{L}\sum_{i=1}^{N}\sum_{j=1}^{M}(x_{ij}^l - \hat{x}_{ij}^l)^2 \quad (3)$$

Now, let $A := \{x_{ij}^l | x_{ij}^l \text{is missing}\}$, $\mathbf{1}_A(.)$ be the indicator function on set A, and $|A|$ be the cardinality of A. Then, the weighted mean squared reconstruction loss is:

$$\frac{NM}{|A|}\sum_{l=1}^{L}\sum_{i=1}^{N}\sum_{j=1}^{M}\mathbf{1}_A(x_{ij}^l)(x_{ij}^l - \hat{x}_{ij}^l)^2 \quad (4)$$

In addition to the weighted reconstruction loss, we adopt an implicit imputation scheme, where imputed values are learned as an integral part of model training. More specifically, Let $\mathbf{x}^l$,

N, M, x^l_{ij}, A and $\mathbf{1}_A(.)$ be defined as above. Also assume that all x^l_{ij} for which $\mathbf{1}_A(x^l_{ij}) = 1$, are initially imputed with arbitrary finite values. Then we add one additional layer before the input LSTM (Figure 1) as follows:

$$\tilde{x}^l_{ij} = x^l_{ij} \times (1 - \mathbf{1}_A(x^l_{ij})) + b_{ij} \times \mathbf{1}_A(x^l_{ij}) \quad (5)$$

Here, x^l_{ij} is the actual observed (or missing) value of sample l at time points i and assessment j, and $\tilde{x}^l_{ij}$ serves as input to the LSTM. In other words, if x^l_{ij} is missing, then it is replaced by b_{ij} in $\tilde{\mathbf{x}}$. Parameters b_{ij} are trained as an integral part of VaDER using stochastic gradient descent, and can be considered (time, assessment)-specific expected values. Note that (1) the initial arbitrary imputation does not influence the eventual clustering, and (2) the implicitly imputed values are weighted to 0 in the reconstruction loss.

VaDER achieves high accuracy on simulated data

As a first step in technically validating VaDER, we simulated data with a known ground truth clustering, and assessed how well VaDER was able to recover these clusters. A natural framework to this end is the vector autoregressive (VAR) model, because (1) it can express serial correlation between time points, (2) it can express cross-correlation between variables, and (3) given a fully parameterized VAR process, one can simulate random trajectories from that VAR process.

More specifically, to generate clusters of multivariate short time series, we simulated from VAR process mixtures, for different values of a clusterability parameter λ. The clusterability parameter λ influences how easily separable the simulated clusters are (see Section Simulation experiments). Sample data is provided in the Supplemental Material. We used the cluster purity measure [36] to record how well the true clustering could be recovered (for more details, see Section Methods).

VaDER was able to highly accurately recover the simulated clusters, achieving a cluster purity of >0.9 for $\lambda \approx 0.08$, and converging to 1.0 for larger λ (Figure 2a). Moreover, even without extensive hyperparameter optimization, VaDER performed substantially better than hierarchical clustering using various distance measures, some of which specifically designed for multivariate time series (Multidimensional Dynamic Time Warping (MD-DTW [38]) and Global Alignment Kernels (GAK [39])) or short time series (STS [37]). Only for $\lambda < 0.04$ VaDER was outperformed by multi-dimensional dynamic time-warping. This may be attributed to the fairly limited number of samples used for the simulation ($n = 2000$), and omitting extensive optimization of VaDER's hyperparameters.

We used the same VAR framework to assess how varying degrees of missing values affect the performance of VaDER. Both missing values completely at random (MCAR) and missing values not at random (MNAR) were simulated as described in Section Methods. In the MCAR simulation, missing values were uniformly distributed across time and clinical outcome measures. In the MNAR simulation, the expected degree of missing values sigmoidally depended on time (see Section Methods). For varying clusterability levels λ, it can be seen that VaDER's implicit imputation scheme is overall more robust against missing values than using VaDER with pre-imputation of missing values (Figures 2b and 2c).

VaDER achieves high accuracy on benchmark classification datasets

As an additional validation step towards applying VaDER to real-world clinical data, we collected a number of real-world benchmark datasets for multivariate time series classification (Table 1). The datasets were normalized and processed to equal and/or shorter length as described in Section Methods.

Comparing the ability of VaDER in recovering these a priori known classes to the other methods mentioned above, it can be seen that VaDER consistently achieves better results (Figure 3a). Moreover, VADER's approach of integrating imputation with model training again outperforms pre-imputation of missing values (Figures 3b and 3c).

Application 1: VaDER identifies clinically diverse AD patient subgroups

After the technical validation using simulated and benchmark data, we applied VaDER to clinical data for identifying meaningful patient subgroups. From the Alzheimer's Disease Neuroimaging Initiative (ADNI) [25], we collected data from 689 patients that were at some point diagnosed with dementia during the course of this study. Four different cognitive assessment scores were available at 8 different visits: ADAS13, CDRSB, MMSE and FAQ. We pre-processed the data as described in Section ADNI data preparation. Overall, the fraction of missing values was $\sim 43\%$. We used VaDER to cluster patients by disease progression as measured using these cognitive assessments.

Hyperparameter optimization was performed by random grid search as described in Section Methods. For each number of clusters $k \in \{2 \ldots 15\}$, the prediction strength [42] of the corresponding optimal model was compared to a null distribution (see Section Hyperparameter optimization and choice of number of clusters), which is shown in the Supplemental Materials.

For most practical applications, determining an unambiguously correct number of clusters k is not possible, and a wide range of rules-of-thumb exist, see e.g. [43, 44, 45, 46, 42]. For our subsequent analyses, we chose $k = 3$. This demonstrated relatively high prediction strength, significantly different from the null distribution, while still allowing VaDER to demonstrate its ability to uncover potentially interesting statistical interactions between trajectories of different cognitive assessments. A statistical interaction between different cognitive assessments could e.g. manifest in the ability to distinguish patient subgroups based on one cognitive assessment, while this is not possible on another assessment. Another example would be a permuted ordering of clusters with respect to different assessments scores.

For ADNI data the resulting cluster mean trajectories are shown in Figure 4, and demonstrate that (1) VaDER effectively clusters the data into patient subgroups showing divergent disease progression, and (2) VaDER is able to find interactions between the different cognitive assessments, which would be principally difficult to distill from univariate analyses of the assessments. For example, the patients in cluster 1 are the most severely progressing patients when assessed using ADAS13, CDRSB and MMSE. However, the FAQ assessment (instrumental activities of daily living) does not distinguish between these severely progressing patients and the more moderately progressing patients in cluster 1.

In addition to cognitive assessment measurements, ADNI presents a wealth of data on brain volume and various AD markers that we did not use for clustering. In this data, we identified numerous statistically significant associations with our patient subgroups. For example, the clusters strongly associated with time-to-dementia diagnosis relative to baseline, with cluster 2 showing generally the shortest time, and cluster 0 the longest. The relatively mildly progressing patients in cluster 0 also demonstrated on average a larger whole brain volume at baseline, which moreover declined less steeply over time, compared to more severely progressing patients. Especially the middle temporal gyri and fusiform gyri were larger (and shrinking more slowly over time), whereas the ventricles were smaller (and expanding more slowly over time). In-

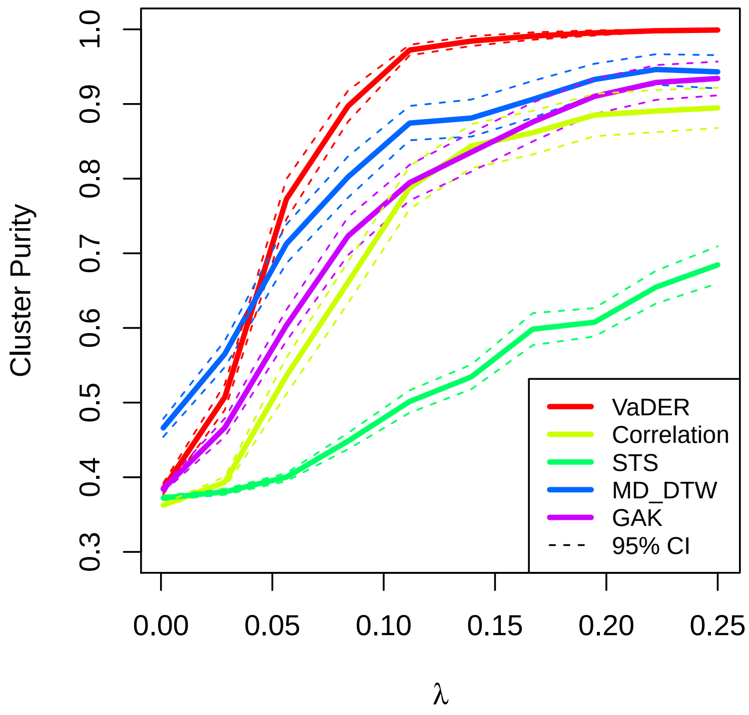

(a) Cluster purity [36] for clustering of simulated data as a function of the clusterability parameter λ, with higher λ implying a higher degree of similarity between profiles in the same cluster. Results are shown for VaDER as well as hierarchical clustering using five different distance measures, (1) Euclidean distance, (2) Pearson correlation, (3) Short time series (STS) distance [37], (4) Multi-dimensional dynamic time warping (MD_DTW) [38] and (5) Global Alignment Kernels (GAK) [39].

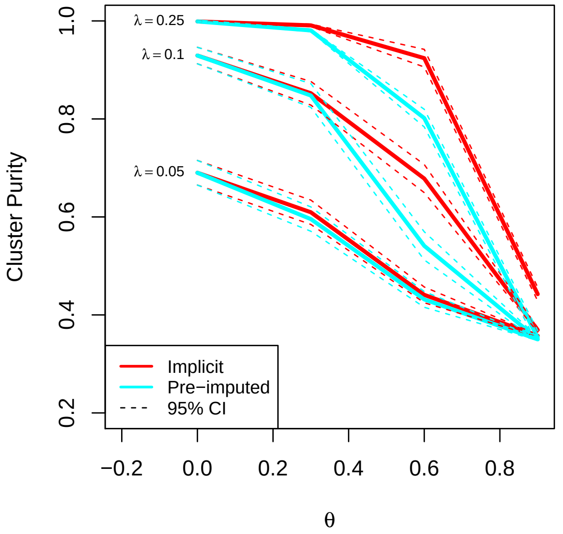

(b) Cluster purity as a function of the fraction θ of values missing completely at random (MCAR), for various levels of the clusterability parameter λ, for both VaDER with implicit imputation and VaDER with pre-imputation. Confidence intervals were determined by repeating the clustering 100 times using newly generated random data and missingness patterns.

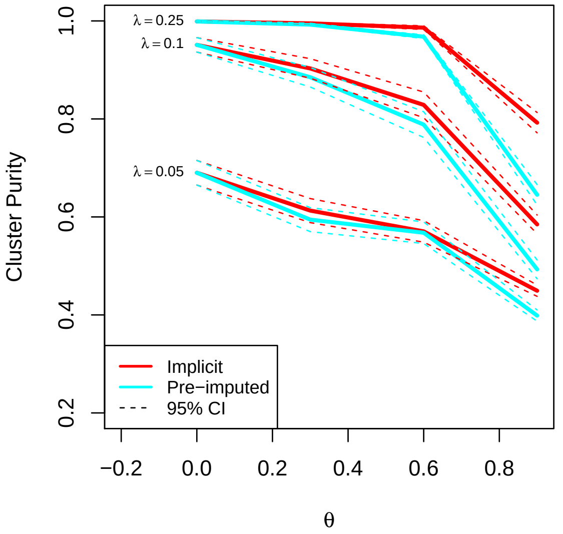

(c) Cluster purity as a function of the fraction θ of values missing not at random (MNAR) (see Section Methods for details), for various levels of the clusterability parameter λ, for both VaDER with implicit imputation and VaDER with pre-imputation. Confidence intervals were determined by repeating the clustering 100 times using newly generated random data and missingness patterns.

Figure 2. VaDER performance on simulated data, with varying degrees of clusterability and missingness.

deed, atrophy of the middle temporal gyri and fusiform gyri, as well as ventricular enlargement, have been associated with Alzheimer's disease progression [47, 48]. As another example, the more severely progressing patients (clusters 1 and es-

Table 1. Multivariate time series classification datasets used in this study.

Name	k	n	p	n_t	n'_t	Source
ArabicDigits	10	8800	13	4 - 93	24	UCI [40]
JapaneseVowels	9	640	12	7 - 29	15	UEA/UCR [41]
CharacterTrajectories	20	2858	3	109 - 205	23	UCI [40]
UWave	8	4478	3	315	25	UCI [40]

k: number of classes.
n: number of samples.
p: number of variables.
n_t: number of time points.
n'_t: number of samples after processing to equal and/or shorter length.

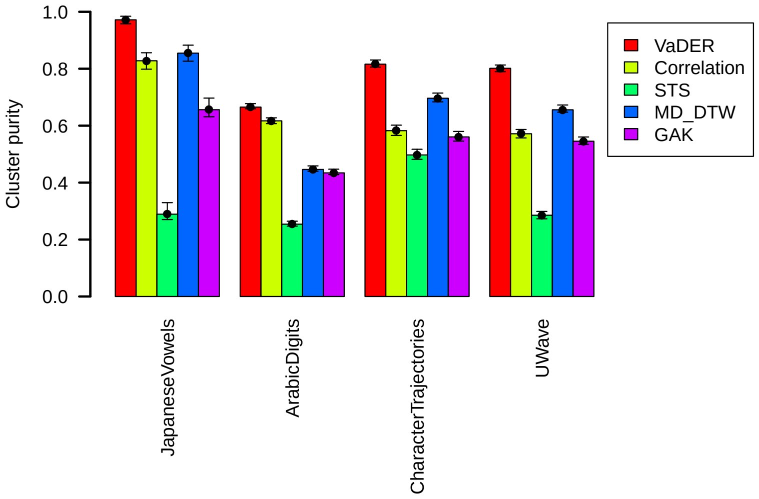

(a) Cluster purity [36] for clustering of benchmark data. Results are shown for VaDER as well as hierarchical clustering using five different distance measures, (1) Euclidean distance, (2) Pearson correlation, (3) Short time series (STS) distance [37], (4) Multi-dimensional dynamic time warping (MD_DTW) [38] and (5) Global Alignment Kernels (GAK) [39]. For each dataset, the best performance across methods is marked by a horizontal dotted line. Confidence intervals were determined by bootstrapping the clustering 10^3 times.

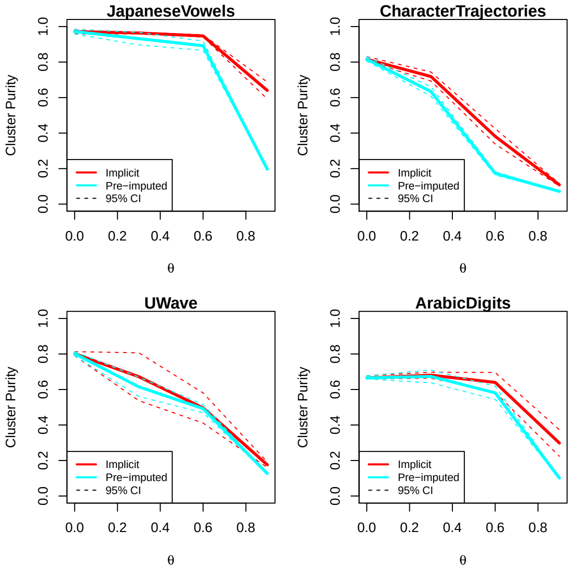

(b) Cluster purity as a function of the fraction θ of values missing completely at random (MCAR), for both VaDER with implicit imputation and VaDER with pre-imputation. Confidence intervals were by repeating the clustering 5 times using newly generated random missingness patterns.

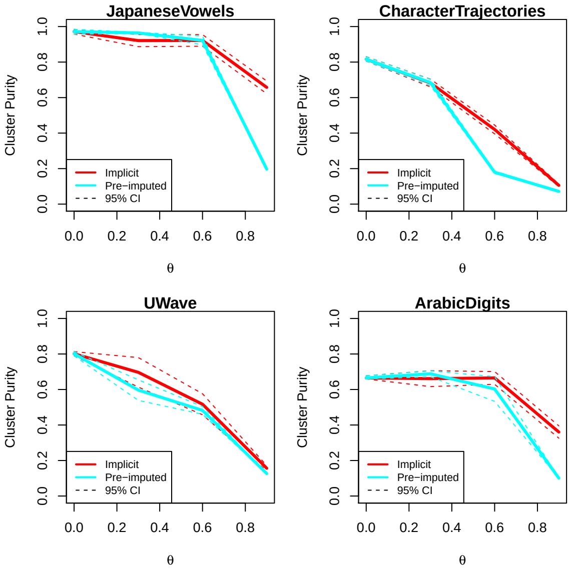

(c) Cluster purity as a function of the fraction θ of values missing not at random (MNAR), for both VaDER with implicit imputation and VaDER with pre-imputation. Confidence intervals were by repeating the clustering 5 times using newly generated random missingness patterns.

Figure 3. VaDER performance on benchmark data, for varying degrees of missingness.

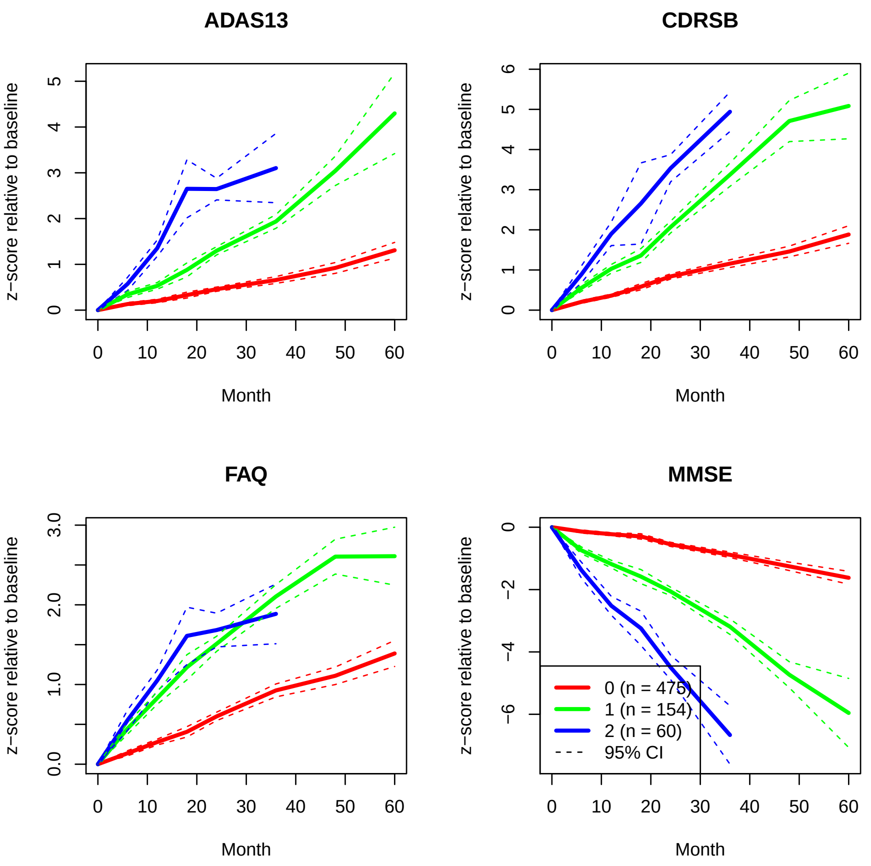

Figure 4. Normalized cluster mean trajectories relative to baseline (x-axis in months), as identified by VaDER from the ADNI cognitive assessment data.

pecially 2), demonstrated lower cerebral glucose uptake and lower cerebrospinal Abeta42 levels, again confirming the literature [49, 50] (see Section Methods and Supplemental Material). These observations demonstrate that the clinical differences between our patient subgroups reflect known Alzheimer's disease aspects.

Application 2: VaDER identifies clinically diverse PD patient subgroups

We additionally applied VaDER to clinical data from the Parkinson's Progression Markers Initiative (PPMI) [26]. From PPMI, we collected data from 362 de novo PD patients that had been diagnosed within a time period of two years before study onset and were initially not been treated. 9 variables on several motor and non-motor symptoms (UPDRS total, UPDRS1-3, TD, PIGD, RBD, ESS, SCOPA-AUT) measured at either 5 or 10 time points were available. The data was pre-processed as described in Section PPMI data preparation. Overall, the fraction of missingness values was ~ 17% (or ~ 31%, when including time points entirely missing for some assessments). We again used VaDER to cluster patients according to disease progression as measured by these assessments.

Hyperparameter optimization and selection of the number of clusters was performed in the same way as for ADNI, and we decided on $k = 3$ patient subgroups accordingly. The resulting cluster mean trajectories are shown in Figure 5. These again illustrate that (1) VaDER effectively clusters the data into clinically divergent patient subgroups, and (2) VaDER is able to find interactions between the assessments that would principally be difficult to find based on univariate analyses alone. For example, cluster 0 represents patients with a moderate progression in terms of mental impairment, behavior, and mood (UPDRS1) and autonomic dysfunction (SCOPA). However, these patients remain relatively stable, or even improve, on many other assessments, such as tremor dominance (TD), the self-reported ability to perform activities of daily life (UPDRS2) and motor symptoms evaluation (UPDRS3).

Similar to ADNI, PPMI presents a wealth of additional data on brain volume and various PD markers that were not used for clustering. Aligning these data with our PD patient subgroups, we found numerous statistically significant associations that confirmed existing literature, many related to quality of life and physiological changes to the brain. For example, men were over-represented in cluster 1, and showed the most severe disease progression, confirming the literature on gender differences in PD (e.g. [51]). Moreover, these severely progressing patients showed an expected steeply declining ability to perform activities of daily living (modified Schwab and England score [52]), as well as rapidly developing symptoms of depression (geriatric depression scale [53]), common in PD patients [54]. Additionally, these patients demonstrated physiological differences in the brain when compared to more mildly progressing patients. Examples are the caudate nucleus and putamen brain regions, which were smaller at baseline and during follow-ups in the more severely progressing patients in cluster 1, and from the literature are known to be subject to atrophy in PD [55] (see Section Methods and Supplemental Material). These observations demonstrate that the clinical differences between our patient subgroups reflect known aspects of PD disease progression.

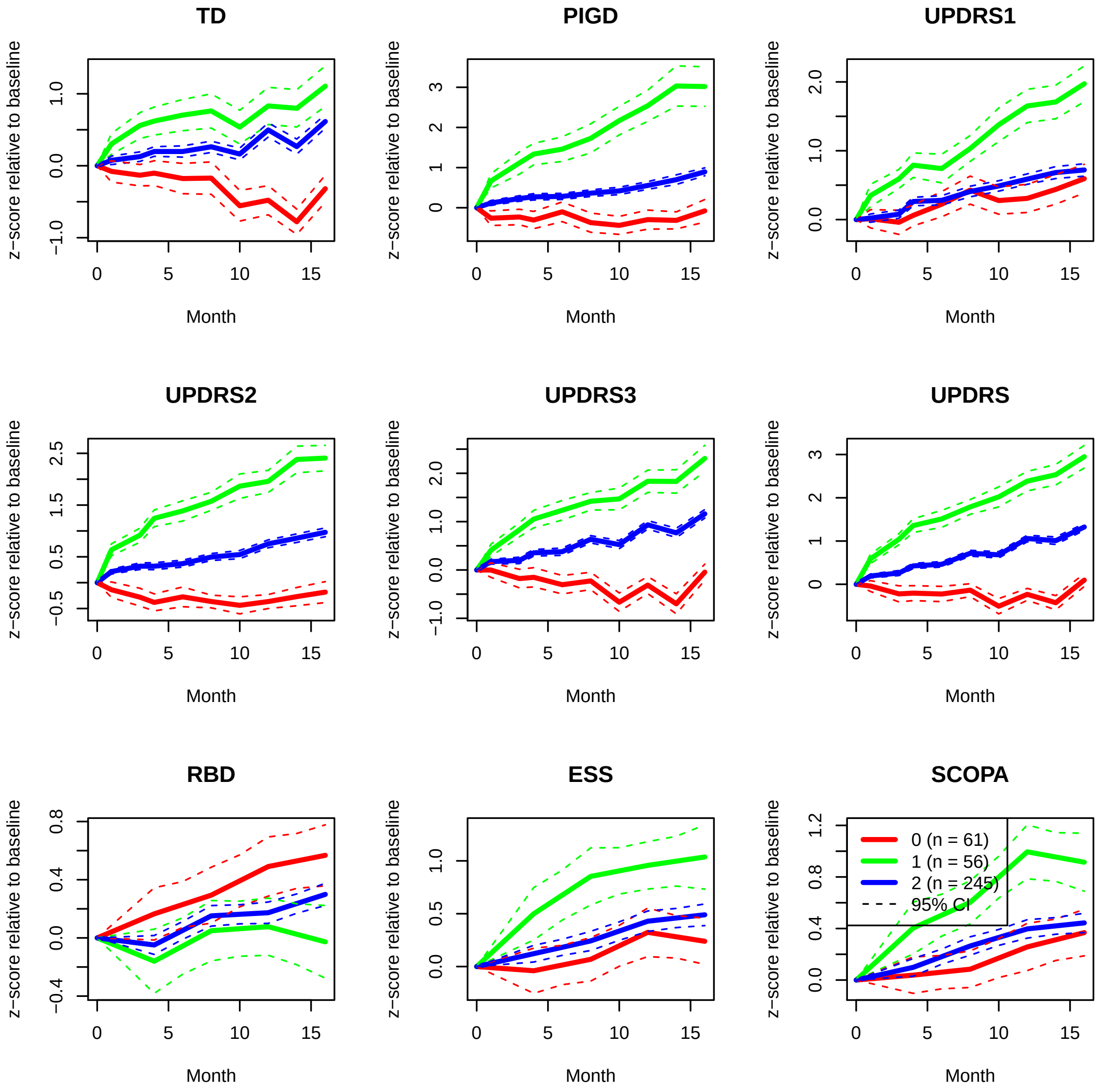

Figure 5. Normalized cluster mean trajectories relative to baseline (x-axis in months), as identified by VaDER from the PPMI motor/non-motor score data.

Discussion and conclusions

Identifying subgroups of patients with similar progression patterns can help to better understand the heterogeneity of complex diseases. Together with predictive machine learning methods, this might help to better decide on the right time and type of treatment for an individual patient, as well as to improve the design of clinical studies. However, one of the main challenges is the multifaceted nature of progression in many areas of disease.

In this paper, we proposed VaDER, a method for clustering multivariate short time series with potentially many missing values, a setting that seems generally not well addressed in the literature so far, but is nonetheless often encountered in clinical study data.

We validated VaDER by showing the very high accuracy on clustering simulated and real-world benchmark data with a known ground truth. We then applied VaDER to data from (1) ADNI and (2) PPMI, resulting in subgroups characterized by clinically highly divergent disease progression profiles. A comparison with other data from ADNI and PPMI, such as brain imaging, motor- and cognitive assessment data, furthermore supported the observed patient subgroups.

VaDER has two main distinctive features. One is that VaDER deals directly with missing values. For clinical research this is crucial, since clinical datasets often show a very high degree of missing values [56, 57]. The other main distinctive feature is that, as opposed to existing methods [10, 11, 12, 13, 14], VaDER is specifically designed to deal with multivariate and relatively short time series that are typical for (observational) clinical studies. However, it is worthwhile to mention that the application of VaDER is not per se limited to longitudinal clinical study data. Future applications (potentially requiring some adaptations) could e.g. include data originating from electronic health records, multiple wearable sensors, video recordings, or time series gene (co-)expression. Moreover, VaDER could be used as a generative model: given a trained model, it is possible to generate "virtual" patient trajectories.

Altogether, we believe that our results show that VaDER has the potential to enhance future patient stratification efforts, and multivariate short time series clustering in general.

Methods

Data preparation

ADNI data preparation

Data used in the preparation of this article were obtained from the Alzheimer's Disease Neuroimaging Initiative (ADNI) database (adni.loni.usc.edu). The ADNI was launched in 2003 as a public-private partnership, led by Principal Investigator Michael W. Weiner,MD. The primary goal of ADNI has been to test whether serial magnetic resonance imaging (MRI), positron emission tomography (PET), other biological markers, and clinical and neuropsychological assessment can be combined to measure the progression of mild cognitive impairment (MCI) and early Alzheimer's disease (AD). For up-to-date information, see www.adni-info.org.

The ADNIMERGE R-package [58] contains mainly two categories of data, (1) longitudinal and (2) non-longitudinal. These data represent 1737 participants that include healthy controls and patients diagnosed with Alzheimer's Disease (AD). The non-longitudinal features such as demographics and APOE e4 status were measured only once, at baseline. The longitudinal features (i.e. neuroimaging features, cerebrospinal fluid (CSF) biomarkers, cognitive tests and everyday cognition) were recorded over a span of 5 years.

Clinical data. In the current study, we have focused on those participants who were diagnosed with AD at baseline or during one of the follow-up visits. After this filtering step, we had a total of 689 patients. For these 689 patients, four cognitive assessments were selected for clustering:

- ADAS-13: The Alzheimer's disease assessment scale
- CDRSB: The clinical dementia rating sum of box score.
- FAQ: The functional activities questionnaire.
- MMSE: mini-mental state examination

The above assessments were taken at baseline and at 6, 12, 18, 24, 36 48 and 60 months after baseline. For each of the four cognitive assessments, all time points were normalized relative to baseline by (1) subtracting the baseline mean across the 689 patients, and (2) dividing by the baseline standard deviation across the 689 patients.

Imaging data. All available MR scans (T1-weighted scans) from the ADNI database were quantified by an open-source, automated segmentation pipeline at the Erasmus University Medical Center, The Netherlands. The number of slices of the T1w scans varied from 160 to 196 and the in-plane resolution was 256 x 256 on average, yielding an overall voxel-size of 1.2 x 1.0 x 1.0 mm. From the 1715 baseline ADNI scans, the volumes of 34 bilateral cortical brain regions, 68 structures in total, were calculated using a model- and surface-based automated image segmentation procedure, incorporated in the FreeSurfer Package (v.6.0, http://surfer.nmr.mgh.harvard.edu/). Segmentation in Freesurfer was performed by rigid-body registration and nonlinear normalization of images to a probabilistic brain atlas. In the segmentation process, each voxel of the MRI volumes was labeled automatically as a corresponding brain region based on two different cortex parcellation guides [59, 60], subdividing the brain into 68 and 191 regions respectively.

PPMI data preparation

Patients were selected if their PD diagnosis was less than 2 years old at baseline time, and if follow up data was available for at least 48 months (5 - 10 time points), resulting in a total of 362 patients. For these 362 patients, a set of 9 motor and non-motor symptoms were selected for clustering:

- TD: tremor-dominant
- PIGD: postural instability and gait disturbance.
- UPDRS1: Unified Parkinson's disease rating scale, part 1: mentation, behavior, and mood.
- UPDRS2: Unified Parkinson's disease rating scale, part 2: activities of daily living.
- UPDRS3: Unified Parkinson's disease rating scale, part 3: motor examination.
- UPDRS: Unified Parkinson's disease rating scale (UPDRS1 + UPDRS2 + UPDRS3).
- RBD: REM sleep behavior disorder.
- ESS: Epworth sleepiness scale.
- SCOPA-AUT: Scales for outcomes in Parkinson's disease: assessment of autonomic dysfunction.

All scores were normalized relative to baseline by (1) subtracting the baseline mean across all patients, and (2) dividing by the baseline standard deviation across all patients.

For some assessments, fewer time points were available. These were treated as missing values.

Benchmark datasets for multivariate time series classification

As no benchmark datasets exist for multivariate short time series clustering, we collected a number of benchmark datasets for multivariate time series classification [40, 41]. Since currently, VaDER still only works with equal-length time series (see also Section Discussion and conclusions), we preprocessed all samples to equal-length time series by linear interpolation between start and end point. Following [61, 62], we chose constant lengths of $\left\lceil \frac{T_{max}}{\left\lceil \frac{T_{max}}{25} \right\rceil} \right\rceil$, where T_{max} is the maximum length of the lengths of the samples in a given dataset.

Moreover, all resulting time series were standardized to zero mean and unit variance.

Variational deep embedding with recurrence (VaDER)

The VaDER model is extensively described in Section Results. This section describes how VaDER was trained.

Pre-training

Similar to [23], we pre-train VaDER by disregarding the latent loss during the first epochs, essentially fitting an non-variational LSTM autoencoder to the data. We then fit a Gaussian mixture distribution in the latent space of this autoencoder, and use its parameters to initialize the final variational training of VaDER.

Hyperparameter optimization and choice of number of clusters

We used prediction strength [42] to select suitable values for VaDER's hyperparameters. These comprise:

- number of layers (for both ADNI and PPMI: $\{1, 2\}$)
- number of nodes per hidden layer (for ADNI: $\{2^0, 2^1, 2^2, 2^3, 2^4, 2^5, 2^6\}$; for PPMI: $\{2^0, 2^1, 2^2, 2^3, 2^4, 2^5, 2^6, 2^7\}$)
- learning rate (for both ADNI and PPMI: $\{10^{-4}, 10^{-3}, 10^{-2}, 10^{-1}\}$)
- mini-batch size (for both ADNI and PPMI: $\{2^4, 2^5, 2^6, 2^7\}$)

Hyperparameter optimization was performed via a random grid search (i.e. by randomly sampling a predefined hyperparameter grid) with repeated cross-validation ($n = 20$), using the reconstruction loss as objective. This was done during the pre-training phase of VaDER.

After hyperparameter optimization we trained VaDER models for different numbers of clusters $k \in \{2 \ldots 15\}$. For each k,

prediction strength was computed by 2-fold cross-validation [42]: For a given training and test dataset:

i. Train VaDER on the training data. (the training data model)
ii. Assign clusters to the test data using the training data model.
iii. Train VaDER on the test data. (the test data model)
iv. Assign clusters to the test data using the test data model.
v. Compare the resulting two clusterings: For each cluster of the test data model, compute the fraction of pairs of samples in that cluster that are also assigned to the same cluster by the training data model. Prediction strength is defined as the minimum proportion across all clusters of the test data model. [42].

Prediction strength was then compared to an empirical null distribution of that measure. The null distribution of the prediction strength was computed by randomly permuting the predicted cluster labels 10^3 times, then recomputing the prediction strength, and eventually taking the average of the 10^3 prediction strength values. Doing this for all 20 repeats, resulted in 20 values for the eventual null distribution, which were then compared to 20 actual prediction strength values (similarly, one for each repeat) by a paired Wilcoxon rank-sum test.

Simulation experiments

Overview of data generating process

To better understand the performance of VaDER we conducted an extensive simulation study: We simulated multivariate short time series via vector autoregressive (VAR) processes [63], because (1) they can model the auto-correlation between time points, (2) they can model the cross-correlation between variables and (3) given a VAR, one can generate random trajectories from that VAR.

We used mixtures of VAR processes to simulated multivariate time series data of the same dimensions as the ADNI data: 4 variables measured over 8 time points. Given a clusterability factor λ, we generated trajectories as follows:

i. Sample coefficient matrices for 3 VAR(8) processes, by randomly sampling the individual entries of each 4×4 matrix from the uniform distribution $\mathcal{U}(-.1, .1)$. Multiply each of the matrix entries by λ.
ii. Randomly sample 3 additional 4×4 matrices from $\mathcal{U}(-.1, .1)$, and multiply each with its own transpose. Let each of results correspond to the variance-covariance matrix of one of the 3 VAR(8) processes.
iii. Repeat 10^3 times:
   i. Randomly select one of the 3 VAR(8) processes (with equal probability).
   ii. Generate a random trajectory from the selected VAR(8) process.

The above generates one set of random data. Given a value of λ, the entire sampling process was repeated 100 times, and each of the 100 datasets was clustered using both VaDER and hierarchical clustering.

For computational reasons, hyper-parameters for VaDER were fixed and not further optimized during our simulation (10^2 epochs of both pre-training and training, learning rate: 10^{-4}, two hidden layers: [36, 4], batch size: 64).

Comparison against hierarchical clustering

We compared VaDER against a conventional hierarchical clustering (complete linkage), in which we flatten the $N \times M$ data matrices of each patient into vectors. We considered three distance measures for these vectors:

- Pearson correlation
- Euclidean distance
- Short time series (STS) distance [37], a distance measure specifically developed for univariate short time series. The STS distance relies on the difference between adjacent time points. Here we first computed the STS distance for each of the different clinical outcome measures, and then summed these up to arrive at an aggregated STS distance across the M clinical measures.

Additionally, we compared VaDER against hierarhical clustering using two distance measures specifically designed for multivariate time series:

- Multidimensional dynamic time warping [38]
- Fast global alignment kernels [39]

Given that VaDER is non-deterministic, we ran 100 replicates for each (simulated / benchmark) dataset, and determined the consensus clustering by hierarchically clustering a consensus matrix listing for each pair of samples how often these two samples were clustered together across the 100 replicates.

Simulating missing data

To test the ability of VaDER to deal with missing data we performed a separate set of simulations: Let L be the number of patients in our dataset, and $\mathbf{x}^l \in \mathbb{R}^{N \times M}$ a single patient trajectory ($l \in 1 \ldots L$), where N is the number of time points and M is the number of measured features. Missing values completely at random (MCAR) were simulated by an individual entry $\mathbf{x}^l_{ij}$ to missing with probability θ.

Missing not at random (MNAR) was simulated by letting the probability of a missing value for entry $\mathbf{x}^l_{ij}$ depend on time. More specifically, each individual entry $\mathbf{x}^l_{ij}$ was set to missing with probability $\frac{1}{1+e^{i_0 - i/k}}$, where $i_0 = \frac{1+N}{2}$, where $i_0 = \frac{1+N}{2}$, and k was varied to result in different overall missingness fractions *theta*.

To compare VADER's implicit imputation with pre-imputation, missing values generated using the above approach were additionally imputed using mean substitution: Each missing value was substituted with the average conditioned on the relevant time point and variable.

Given that VaDER is non-deterministic, we ran 20 replicates for each (simulated / benchmark) dataset, and determined the consensus clustering by hierarchically clustering a consensus matrix listing for each pair of samples how often these two samples were clustered together across the 20 replicates. Confidence intervals were determined by repeating the above procedure 100 times (simulation experiments) or 5 times (benchmark experiments) with newly generated missingness patterns (simulation/benchmark experiments) and/or data (simulation experiments).

Estimating clustering performance

For the simulation and benchmark datasets, the number of clusters is a priori known. Hence, an intuitive measure of comparing the performance between the different algorithms is cluster purity [36]. Cluster purity can be interpreted as the fraction of correctly clustered samples and is calculated as follows:

i. For each cluster, count the number of samples from the majority class in that cluster.
ii. Sum the above counts.
iii. Divide by the total number of samples.

For the ADNI and PPMI data, the number of clusters is not a priori known. Hence, performance was recorded using the adjusted rand index [64, 65] for different values of λ in the interval $[0.001, 0.25]$. For $\lambda \gtrapprox 0.25$, generating coefficient matrices that lead to stable VARs becomes very difficult.

Post-hoc analysis of patient clusters

We collected a wide range of additional variables from ADNI and PPMI, and assessed the association of the identified patient subgroups with a given variable by multinomial logistic regression. For any baseline variable x, we first fitted the following full model:

$$\text{subgroup} \sim x + \text{confounders} \tag{6}$$

Each of these full models were then compared to a null model:

$$\text{subgroup} \sim \text{confounders} \tag{7}$$

by means of a likelihood ratio test.

For any longitudinal variable x measured at timepoints t, we first fitted the following multinomial logistic regression model:

$$\text{subgroup} \sim x + t + x * t + \text{confounders} \tag{8}$$

We tested this model against the null model:

$$\text{subgroup} \sim \text{confounders} \tag{9}$$

by performing a likelihood ratio test, and applying an FDR correction for multiple testing. If the above test was found to be significant ($q < 0.05$), we tested the effects of the individual terms $x * t$, x and t against the same null model above.

Confounders considered were age, education and gender, but were only included when univariate significantly associated with subgroup. For ADNI, this was only age ($p = 0.0029$, ANOVA F-test). For PPMI, this was only gender ($p = 0.0017$, χ^2-test).

In the post-hoc analysis, only complete cases were included, i.e. patients with missing values were ignored.

Availability of supporting source code and requirements

A complete implementation of VaDER in Python/Tensorflow: https://github.com/johanndejong/VaDER.

An R-package for streamlining the processing of PPMI data: https://github.com/patzaw/PPMI-R-package-generator.

Other code used for generating results presented in this paper: https://github.com/johanndejong/VaDER_supporting_code.

Snapshots of all the above code and other supporting data are also available in the GigaScience database, GigaDB [66].

List of abbreviations

- AD: Alzheimer's disease
- ADAS-13: The Alzheimer's disease assessment scale
- ADNI: Alzheimer's Disease Neuroimaging Initiative
- CDRSB: The clinical dementia rating sum of box score.
- CSF: cerebrospinal fluid
- ESS: Epworth sleepiness scale.
- FAQ: The functional activities questionnaire.
- LSTM: long short term memory
- MAR: missing at random
- MCAR: missing completely at random
- MMSE: mini–mental state examination
- MNAR: missing not at random
- PD: Parkinson's disease
- PIGD: postural instability and gait disturbance.
- PPMI: Parkinson's Progression Markers Initiative
- RBD: REM sleep behavior disorder.
- SCOPA: scales for outcomes in Parkinson's disease.
- TD: tremor–dominant
- UPDRS: Unified Parkinson's disease rating scale.
- UPDRS1: Unified Parkinson's disease rating scale, part 1.
- UPDRS2: Unified Parkinson's disease rating scale, part 2.
- UPDRS3: Unified Parkinson's disease rating scale, part 3.
- UCI: The University of California Irvine machine learning repository.
- UEA/UCR: The University of East Anglia / University of California, Riverside time series classification archive.
- VaDE: variational deep embedding
- VaDER: variational deep embedding with recurrence
- VAR: vector auto regression
- VCF: variant call format

Competing interests

JdJ and HF received salaries from UCB Biosciences GmbH. UCB Biosciences GmbH had no influence on the content of this work.

Funding

The research leading to these results has received partial support from the Innovative Medicines Initiative Joint Undertaking under grant agreement #115568, resources of which are composed of financial contribution from the European Union's Seventh Framework Programme (FP7/2007-2013) and EFPIA companies' in kind contribution.

Authors' contributions

Method development: JdJ, HF; implementation and testing: JdJ; Data preparation: MAE, PW, RK, MS, AA; image analysis: HV; supervision: HF, MHA; definition of research project: HF

Acknowledgements

Data collection and sharing for this project was funded by the Alzheimer's Disease Neuroimaging Initiative (ADNI) (National Institutes of Health Grant U01 AG024904) and DOD ADNI (Department of Defense award number W81XWH-12-2-0012). ADNI is funded by the National Institute on Aging, the National Institute of Biomedical Imaging and Bioengineering, and through generous contributions from the following: AbbVie, Alzheimer's Association; Alzheimer's Drug Discovery Foundation; Araclon Biotech; BioClinica, Inc.; Biogen; Bristol-Myers

Squibb Company; CereSpir, Inc.; Cogstate; Eisai Inc.; Elan Pharmaceuticals, Inc.; Eli Lilly and Company; EuroImmun; F. Hoffmann-La Roche Ltd and its affiliated company Genentech, Inc.; Fujirebio; GE Healthcare; IXICO Ltd.; Janssen Alzheimer Immunotherapy Research & Development, LLC.; Johnson & Johnson Pharmaceutical Research & Development LLC.; Lumosity; Lundbeck; Merck & Co., Inc.; Meso Scale Diagnostics, LLC.; NeuroRx Research; Neurotrack Technologies; Novartis Pharmaceuticals Corporation; Pfizer Inc.; Piramal Imaging; Servier; Takeda Pharmaceutical Company; and Transition Therapeutics. The Canadian Institutes of Health Research is providing funds to support ADNI clinical sites in Canada. Private sector contributions are facilitated by the Foundation for the National Institutes of Health (www.fnih.org). The grantee organization is the Northern California Institute for Research and Education, and the study is coordinated by the Alzheimer's Therapeutic Research Institute at the University of Southern California. ADNI data are disseminated by the Laboratory for Neuro Imaging at the University of Southern California.

Data used in the preparation of this article were obtained from the Parkinson's Progression Markers Initiative (PPMI) database (www.ppmi-info.org/data). For up-todate information on the study, visit www.ppmi-info.org. PPMI – a public-private partnership – is funded by the Michael J. Fox Foundation for Parkinson's Research and funding partners. A list of names of all of the PPMI funding partners can be found at www.ppmi-info.org/about-ppmi/who-we-are/study-sponsors/.

References

1. Hruby A, Hu FB. The Epidemiology of Obesity: A Big Picture. Pharmacoeconomics 2015 Jul;33(7):673–689. https://www.ncbi.nlm.nih.gov/pubmed/25471927, 25471927[pmid].
2. van Tilburg J, van Haeften TW, Pearson P, Wijmenga C. Defining the genetic contribution of type 2 diabetes mellitus. Journal of Medical Genetics 2001;38(9):569–578. https://jmg.bmj.com/content/38/9/569.
3. Cordell HJ, Todd JA. Multifactorial inheritance in type 1 diabetes. Trends in Genetics 1995;11(12):499 – 504. http://www.sciencedirect.com/science/article/pii/S016895250089160X.
4. Ruppert V, Maisch B. Genetics of HumanHypertension. Herz 2003 Dec;28(8):655–662. https://doi.org/10.1007/s00059-003-2516-6.
5. Poulter N. Coronary heart disease is a multifactorial disease. American Journal of Hypertension 1999;12(10, Supplement 1):92S – 95S. http://www.sciencedirect.com/science/article/pii/S0895706199001636.
6. Hastie T, Tibshirani R, Friedman JH. The elements of statistical learning: data mining, inference, and prediction, 2nd Edition. Springer series in statistics, Springer; 2009. http://www.worldcat.org/oclc/300478243.
7. Kannan R, Vempala S. On Clusterings - Good, Bad and Spectral. In: Proc. Symp. Found. Comp. Sci.; 2000. p. 367–377.
8. Jain A, Dubes R. Algorithms for Clustering Data. Englewood Cliffs, NJ: Prentice-Hall; 1988.
9. Fukunaga K, Hostetler LD. The estimation of the gradient of a density function, with applications in pattern recognition. IEEE Transactions on Information Theory 1975;21:32–39.
10. Aghabozorgi S, Seyed Shirkhorshidi A, Ying Wah T. Time-series Clustering – A Decade Review. Inf Syst 2015 Oct;53(C):16–38. http://dx.doi.org/10.1016/j.is.2015.04.007.
11. Rani S, Sikka G. Article: Recent Techniques of Clustering of Time Series Data: A Survey. International Journal of Computer Applications 2012 August;52(15):1–9. Full text available.
12. Warren Liao T. Clustering of time series data—a survey. Pattern Recognition 2005;38(11):1857–1874.
13. Ghassempour S, Girosi F, Maeder A. Clustering Multivariate Time Series Using Hidden Markov Models. International Journal of Environmental Research and Public Health 2014;11(3):2741–2763. http://www.mdpi.com/1660-4601/11/3/2741.
14. Sun J. Clustering multivariate time series based on Riemannian manifold. Electronics Letters 2016 September;52:1607–1609(2). https://digital-library.theiet.org/content/journals/10.1049/el.2016.0701.
15. Rubin DB. Inference and Missing Data. Biometrika 1976;63(3):581–592. http://www.jstor.org/stable/2335739.
16. Kang H. The prevention and handling of the missing data. Korean Journal of Anesthesiology 2013 May;64(5):402–406. https://www.ncbi.nlm.nih.gov/pmc/articles/PMC3668100/.
17. Mikolov T, Sutskever I, Chen K, Corrado G, Dean J. Distributed Representations of Words and Phrases and Their Compositionality. In: Proceedings of the 26th International Conference on Neural Information Processing Systems - Volume 2 NIPS'13, USA: Curran Associates Inc.; 2013. p. 3111–3119. http://dl.acm.org/citation.cfm?id=2999792.2999959.
18. Hinton GE, Salakhutdinov RR. Reducing the Dimensionality of Data with Neural Networks. Science 2006;313(5786):504–507. http://science.sciencemag.org/content/313/5786/504.
19. Frome A, Corrado GS, Shlens J, Bengio S, Dean J, Ranzato MA, et al. DeViSE: A Deep Visual-Semantic Embedding Model. In: Burges CJC, Bottou L, Welling M, Ghahramani Z, Weinberger KQ, editors. Advances in Neural Information Processing Systems 26 Curran Associates, Inc.; 2013.p. 2121–2129. http://papers.nips.cc/paper/5204-devise-a-deep-visual-semantic-embedding-model.pdf.
20. Asgari E, Mofrad MRK. Continuous Distributed Representation of Biological Sequences for Deep Proteomics and Genomics. PLOS ONE 2015 11;10(11):1–15. https://doi.org/10.1371/journal.pone.0141287.
21. Trigeorgis G, Bousmalis K, Zafeiriou S, Schuller B. A Deep Semi-NMF Model for Learning Hidden Representations. In: Xing EP, Jebara T, editors. Proceedings of the 31st International Conference on Machine Learning, vol. 32 of Proceedings of Machine Learning Research Bejing, China: PMLR; 2014. p. 1692–1700. http://proceedings.mlr.press/v32/trigeorgis14.html.
22. Xie J, Girshick R, Farhadi A. Unsupervised Deep Embedding for Clustering Analysis. In: Proceedings of the 33rd International Conference on International Conference on Machine Learning - Volume 48 ICML'16, JMLR.org; 2016. p. 478–487. http://dl.acm.org/citation.cfm?id=3045390.3045442.
23. Jiang Z, Zheng Y, Tan H, Tang B, Zhou H. Variational Deep Embedding: An Unsupervised and Generative Approach to Clustering. In: IJCAI ijcai.org; 2017. p. 1965–1972.
24. Hochreiter S, Schmidhuber J. Long Short-Term Memory. Neural Comput 1997 Nov;9(8):1735–1780. http://dx.doi.org/10.1162/neco.1997.9.8.1735.
25. Petersen RC, Aisen PS, Beckett LA, Donohue MC, Gamst AC, Harvey DJ, et al. Alzheimer's Disease Neuroimaging Initiative (ADNI). Neurology 2010;74(3):201–209. http://n.neurology.org/content/74/3/201.

26. Marek K, Jennings D, Lasch S, Siderowf A, Tanner C, Simuni T, et al. The Parkinson Progression Marker Initiative (PPMI). Progress in Neurobiology 2011 12;95(4):629–635.
27. Komarova NL, Thalhauser CJ. High Degree of Heterogeneity in Alzheimer's Disease Progression Patterns. PLoS Computational Biology 2011;7(11). https://doi.org/10.1371/journal.pcbi.1002251.
28. Lam B, Masellis M, Freedman M, Stuss DT, Black SE. Clinical, imaging, and pathological heterogeneity of the Alzheimer's disease syndrome. Alzheimer's Research & Therapy 2013 Jan;5(1):1. https://doi.org/10.1186/alzrt155.
29. Lewis SJG, Foltynie T, Blackwell AD, Robbins TW, Owen AM, Barker RA. Heterogeneity of Parkinson's disease in the early clinical stages using a data driven approach. Journal of Neurology, Neurosurgery & Psychiatry 2005;76(3):343–348. https://jnnp.bmj.com/content/76/3/343.
30. von Coelln R, Shulman LM. Clinical subtypes and genetic heterogeneity: of lumping and splitting in Parkinson disease. Current Opinion in Neurology 2016;29(6). https://journals.lww.com/co-neurology/Fulltext/2016/12000/Clinical_subtypes_and_genetic_heterogeneity___of.10.aspx.
31. Kingma DP, Welling M, Auto-Encoding Variational Bayes; 2013. http://arxiv.org/abs/1312.6114, cite arxiv:1312.6114.
32. Doersch C, Tutorial on Variational Autoencoders; 2016. http://arxiv.org/abs/1606.05908, cite arxiv:1606.05908.
33. Gers FA, Schraudolph NN, Schmidhuber J. Learning Precise Timing with Lstm Recurrent Networks. J Mach Learn Res 2003 Mar;3:115–143. https://doi.org/10.1162/153244303768966139.
34. Lipton ZC, Kale DC, Wetzel RC. Directly Modeling Missing Data in Sequences with RNNs: Improved Classification of Clinical Time Series. In: MLHC, vol. 56 of JMLR Workshop and Conference Proceedings JMLR.org; 2016. p. 253–270.
35. Nazábal A, Olmos PM, Ghahramani Z, Valera I. Handling Incomplete Heterogeneous Data using VAEs. CoRR 2018;abs/1807.03653. http://arxiv.org/abs/1807.03653.
36. Manning CD, Raghavan P, Schütze H. Introduction to Information Retrieval. New York, NY, USA: Cambridge University Press; 2008.
37. Möller-Levet CS, Klawonn F, Cho K, Wolkenhauer O. Fuzzy Clustering of Short Time-Series and Unevenly Distributed Sampling Points. In: Berthold MR, Lenz H, Bradley E, Kruse R, Borgelt C, editors. Advances in Intelligent Data Analysis V, 5th International Symposium on Intelligent Data Analysis, IDA 2003, Berlin, Germany, August 28-30, 2003, Proceedings, vol. 2810 of Lecture Notes in Computer Science Springer; 2003. p. 330–340. https://doi.org/10.1007/978-3-540-45231-7_31.
38. Tormene P, Giorgino T, Quaglini S, Stefanelli M. Matching Incomplete Time Series with Dynamic Time Warping: An Algorithm and an Application to Post-Stroke Rehabilitation. Artificial Intelligence in Medicine 2008;45(1):11–34. http://dx.doi.org/10.1016/j.artmed.2008.11.007.
39. Cuturi M. Fast Global Alignment Kernels. In: Getoor L, Scheffer T, editors. ICML Omnipress; 2011. p. 929–936. http://dblp.uni-trier.de/db/conf/icml/icml2011.html#Cuturi11.
40. Dua D, Graff C, UCI Machine Learning Repository; 2017. http://archive.ics.uci.edu/ml.
41. Bagnall A, Lines J, Vickers W, The UEA and UCR Time Series Classification Repository;. Accessed: 2019-08-15. http://www.timeseriesclassification.com.
42. Tibshirani R, Walther G. Cluster validation by prediction strength. Journal of Computational and Graphical Statistics 2005;14(3):511–528.
43. Tibshirani R, Walther G, Hastie T. Estimating the number of clusters in a data set via the gap statistic. Journal of the Royal Statistical Society: Series B (Statistical Methodology) 2001;63(2):411–423. https://rss.onlinelibrary.wiley.com/doi/abs/10.1111/1467-9868.00293.
44. Sugar CA, James GM. Finding the Number of Clusters in a Dataset: An Information-Theoretic Approach. Journal of the American Statistical Association 2003;98(463):750–763. http://www.jstor.org/stable/30045303.
45. Thorndike RL. Who belongs in the family. Psychometrika 1953;p. 267–276.
46. Rousseeuw PJ. Silhouettes: A graphical aid to the interpretation and validation of cluster analysis. Journal of Computational and Applied Mathematics 1987;20:53 – 65. http://www.sciencedirect.com/science/article/pii/0377042787901257.
47. Convit A, de Asis J, de Leon MJ, Tarshish CY, Santi SD, Rusinek H. Atrophy of the medial occipitotemporal, inferior, and middle temporal gyri in non-demented elderly predict decline to Alzheimer's disease. Neurobiology of Aging 2000;21(1):19 – 26. http://www.sciencedirect.com/science/article/pii/S0197458099001074.
48. Nestor SM, Rupsingh R, Borrie M, Smith M, Accomazzi V, Wells JL, et al. Ventricular enlargement as a possible measure of Alzheimer's disease progression validated using the Alzheimer's disease neuroimaging initiative database. Brain 2008 07;131(9):2443–2454. https://doi.org/10.1093/brain/awn146.
49. Butterfield DA, Halliwell B. Oxidative stress, dysfunctional glucose metabolism and Alzheimer disease. Nature Reviews Neuroscience 2019;20(3):148–160. https://doi.org/10.1038/s41583-019-0132-6.
50. Tapiola T, Alafuzoff I, Herukka SK, Parkkinen L, Hartikainen P, Soininen H, et al. Cerebrospinal Fluid Beta-Amyloid 42 and Tau Proteins as Biomarkers of Alzheimer-Type Pathologic Changes in the Brain. JAMA Neurology 2009 03;66(3):382–389. https://doi.org/10.1001/archneurol.2008.596.
51. Moisan F, Kab S, Mohamed F, Canonico M, Le Guern M, Quintin C, et al. Parkinson disease male-to-female ratios increase with age: French nationwide study and meta-analysis. Journal of Neurology, Neurosurgery & Psychiatry 2016;87(9):952–957. https://jnnp.bmj.com/content/87/9/952.
52. Schrag A, Jahanshahi M, Quinn N. What contributes to quality of life in patients with Parkinson's disease? Journal of Neurology, Neurosurgery & Psychiatry 2000;69(3):308–312. https://jnnp.bmj.com/content/69/3/308.
53. Sheikh JI, Yesavage JA. Geriatric Depression Scale (GDS): Recent evidence and development of a shorter version. Clinical Gerontologist: The Journal of Aging and Mental Health 1986;5(1-2):165–173.
54. Marsh L. Depression and Parkinson's disease: current knowledge. Curr Neurol Neurosci Rep 2013 Dec;13(12):409–409. https://www.ncbi.nlm.nih.gov/pubmed/2419078, 24190780[pmid].
55. Pitcher TL, Melzer TR, MacAskill MR, Graham CF, Livingston L, Keenan RJ, et al. Reduced striatal volumes in Parkinson's disease: a magnetic resonance imaging study. Translational Neurodegeneration 2012 Aug;1(1):17. https://doi.org/10.1186/2047-9158-1-17.
56. Pedersen AB, Mikkelsen EM, Cronin-Fenton D, Kristensen NR, Pham TM, Pedersen L, et al. Missing data and multiple imputation in clinical epidemiological research. Clin Epidemiol 2017 Mar;9:157–166. https://www.ncbi.nlm.nih.

gov/pubmed/28352203, 28352203[pmid].
57. Marston L, Carpenter JR, Walters KR, Morris RW, Nazareth I, Petersen I. Issues in multiple imputation of missing data for large general practice clinical databases. Pharmacoepidemiology and Drug Safety 2010;19(6):618–626. https://onlinelibrary.wiley.com/doi/abs/10.1002/pds.1934.
58. the ADNI team. ADNIMERGE: Alzheimer's Disease Neuroimaging Initiative; 2018, r package version 0.0.1.
59. Desikan R, Ségonne F, Fischl B, Quinn BT, Dickerson BC, Blacker D, et al. An automated labeling system for subdividing the human cerebral cortex on MRI scans into gyral based regions of interest. NeuroImage 2006;31(3):968–980. https://doi.org/10.1016/j.neuroimage.2006.01.021.
60. Destrieux C, Fischl B, Dale AM, Halgren E. Automatic parcellation of human cortical gyri and sulci using standard anatomical nomenclature. NeuroImage 2010;53(1):1–15. http://dblp.uni-trier.de/db/journals/neuroimage/neuroimage53.html#DestrieuxFDH10.
61. Wang L, Wang Z, Liu S. An Effective Multivariate Time Series Classification Approach Using Echo State Network and Adaptive Differential Evolution Algorithm. Expert Syst Appl 2016 Jan;43(C):237–249. https://doi.org/10.1016/j.eswa.2015.08.055.
62. Øyvind Mikalsen K, Bianchi FM, Soguero-Ruiz C, Jenssen R. Time series cluster kernel for learning similarities between multivariate time series with missing data. Pattern Recognition 2018;76:569 – 581. http://www.sciencedirect.com/science/article/pii/S0031320317304843.
63. Sims C. Macroeconomics and Reality. Econometrica 1980;48(1):1–48. https://EconPapers.repec.org/RePEc:ecm:emetrp:v:48:y:1980:i:1:p:1-48.
64. Rand WM. Objective criteria for the evaluation of clustering methods. Journal of the American Statistical Association 1971;66(336):846–850.
65. Hubert L, Arabie P. Comparing partitions. Journal of classification 1985;2(1):193–218. http://scholar.google.de/scholar.bib?q=info:IkrWWF2JxwoJ:scholar.google.com/&output=citation&hl=de&ct=citation&cd=0.
66. de Jong J, Emon MA, Wu P, Karki R, Sood M, Godard P, et al. Supporting data for "Deep learning for clustering of multivariate clinical patient trajectories with missing values". GigaScience Database 2019;.

Click here to access/download;Figure;ADNI__variables_brain_level2.pdf

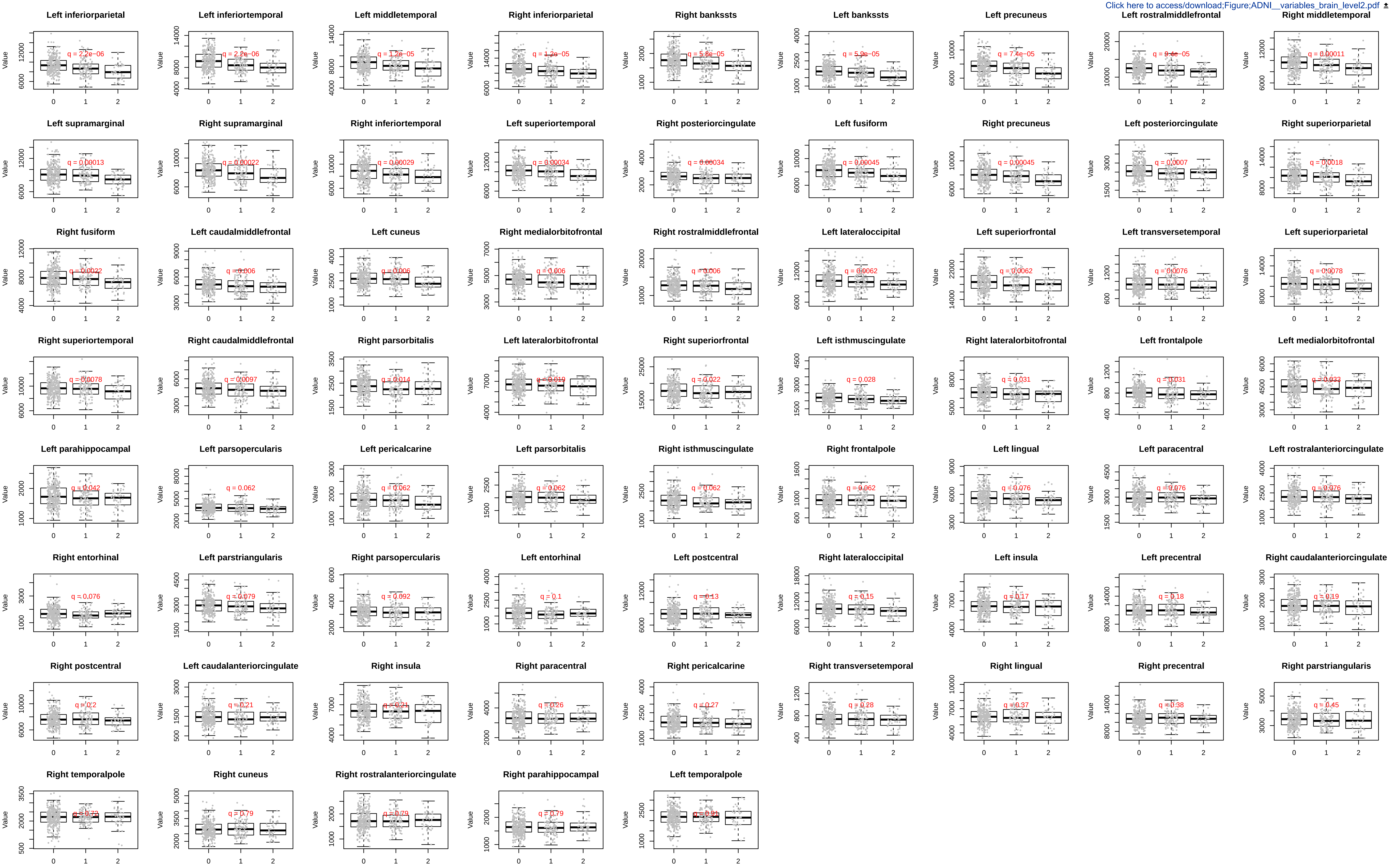

Click here to access/download;Figure;ADNI__variables_brain_level3.pdf

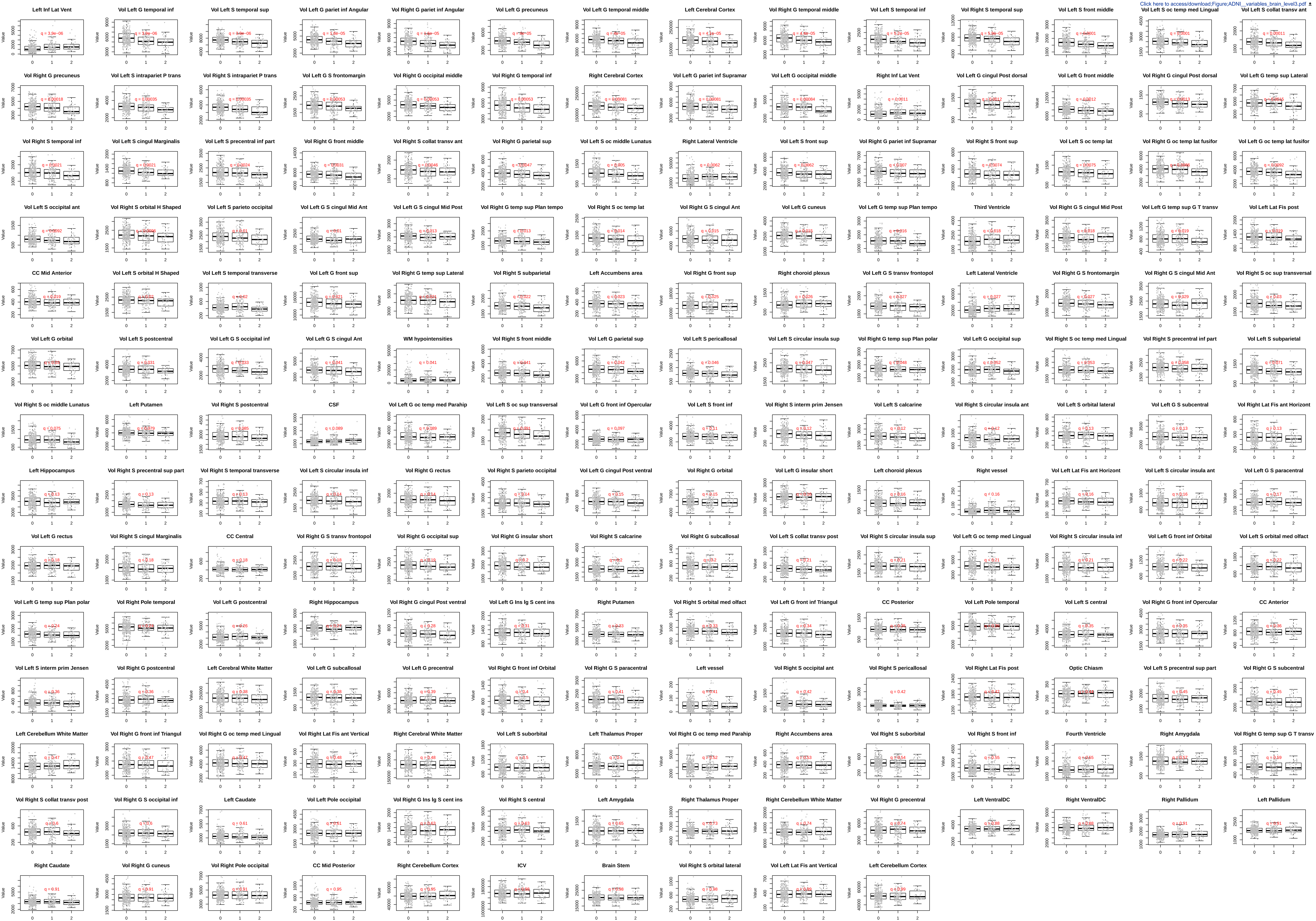

Click here to access/download;Figure;ADNI__variables_marker.pdf

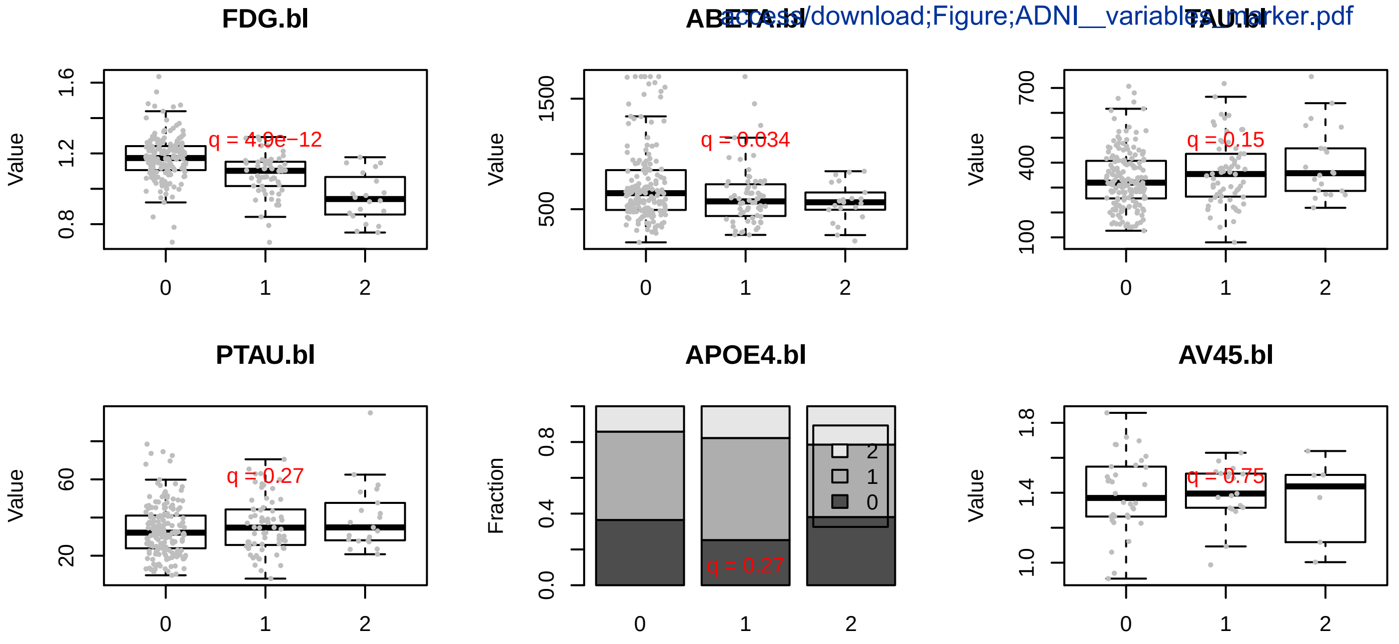

Click here to access/download;Figure;ADNI__variables_patient_chara

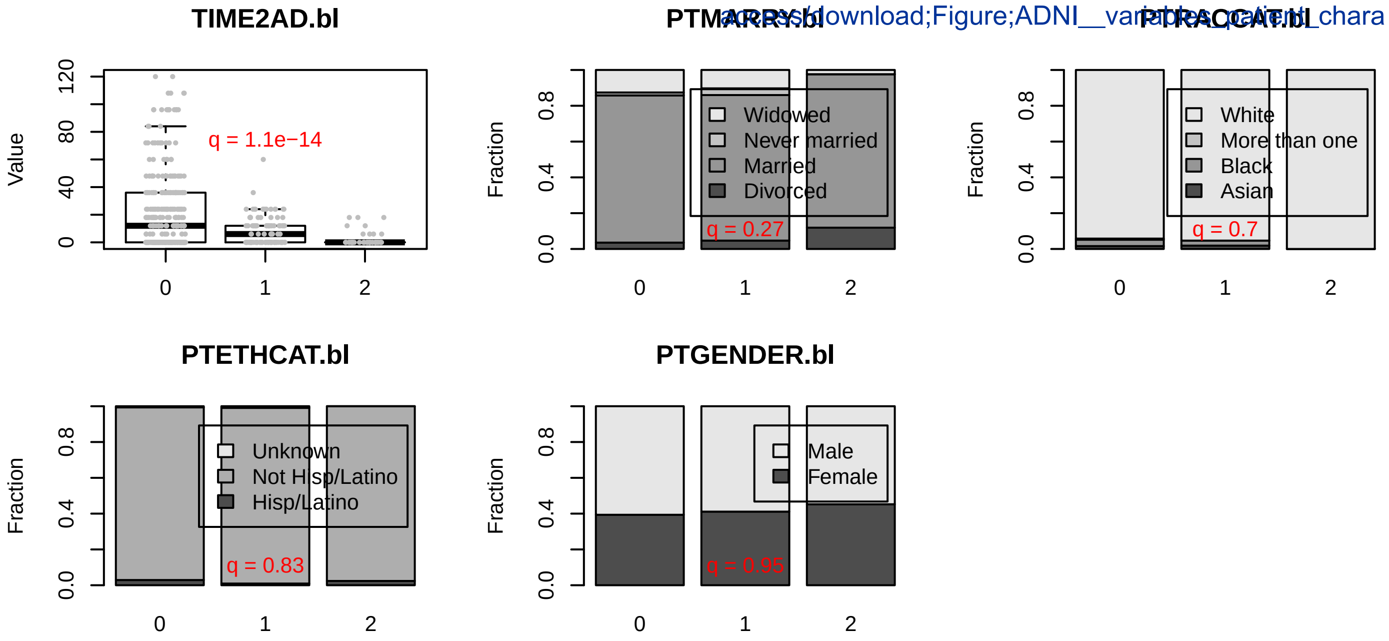

Click here to access/download;Figure;ADNI__variables_series.pdf

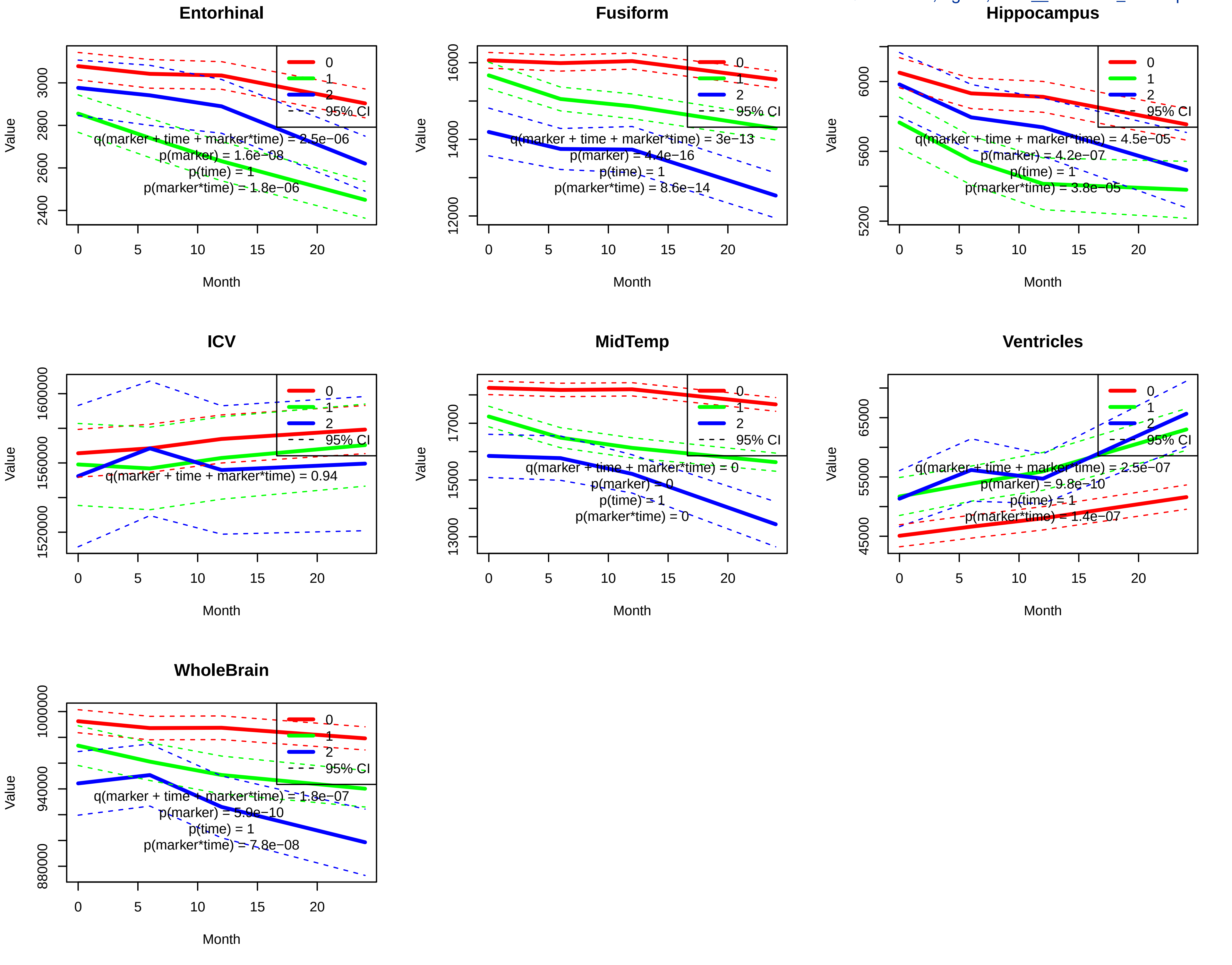

Click here to access/download;Figure;ADNI__variables_series_subset.pdf

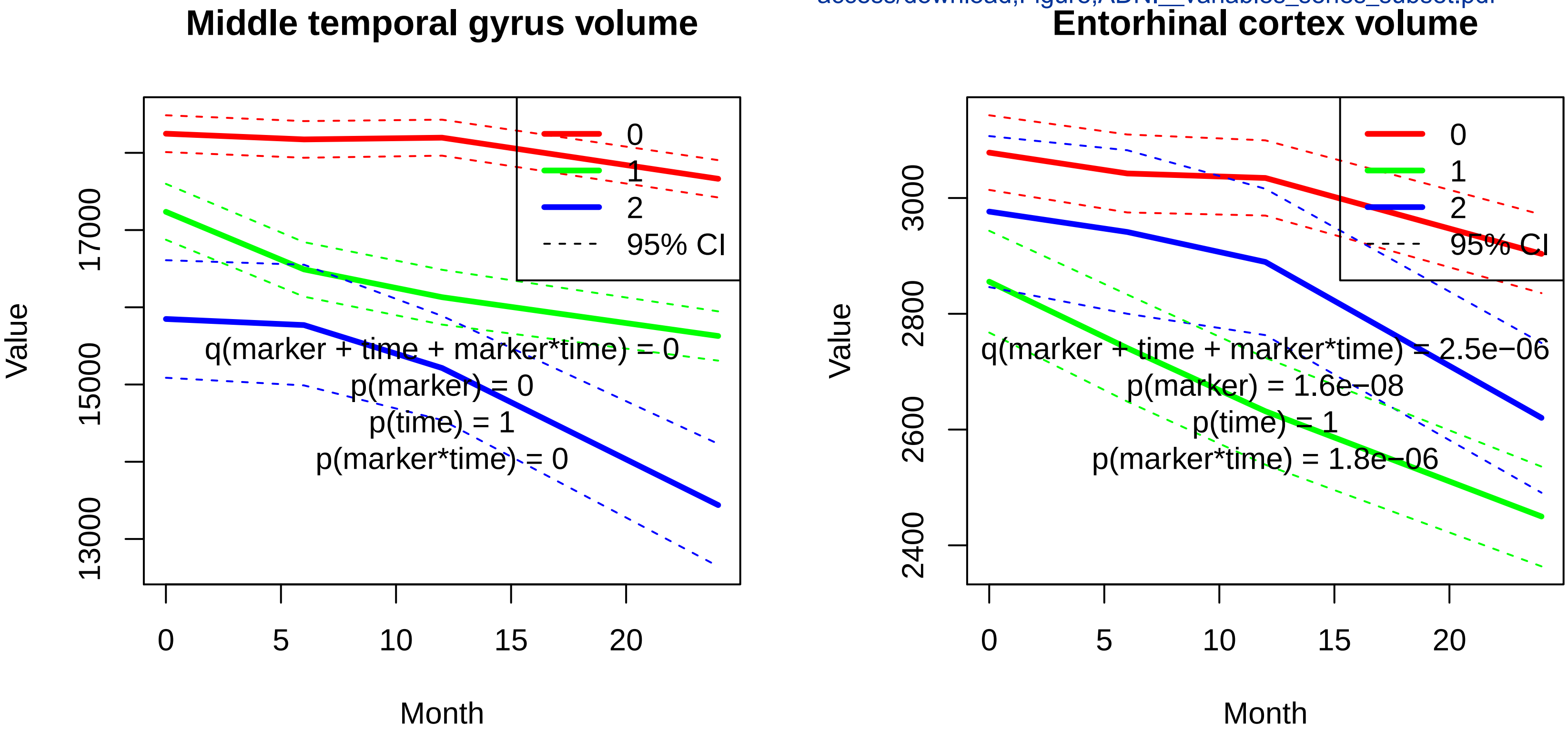

Click here to access/download;Figure;ADNI__variables_s

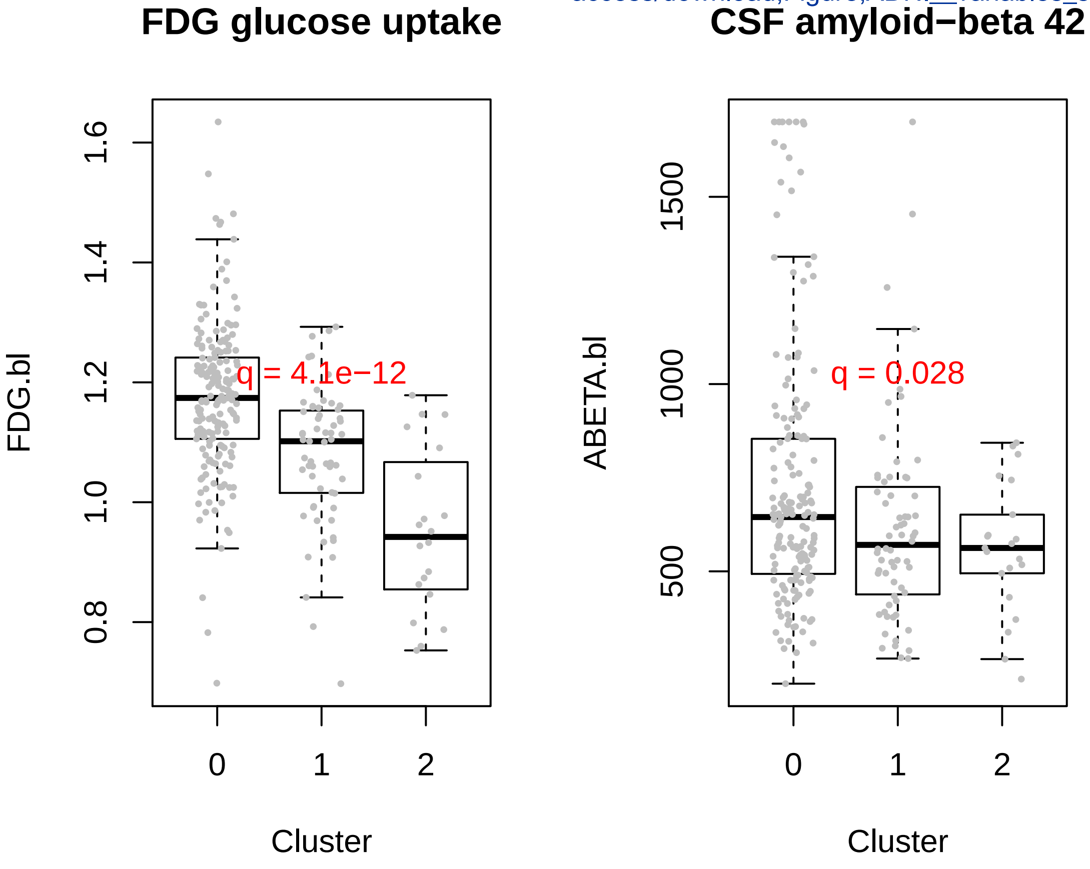

Click here to access/download;Figure;example_data_n5E2.pdf

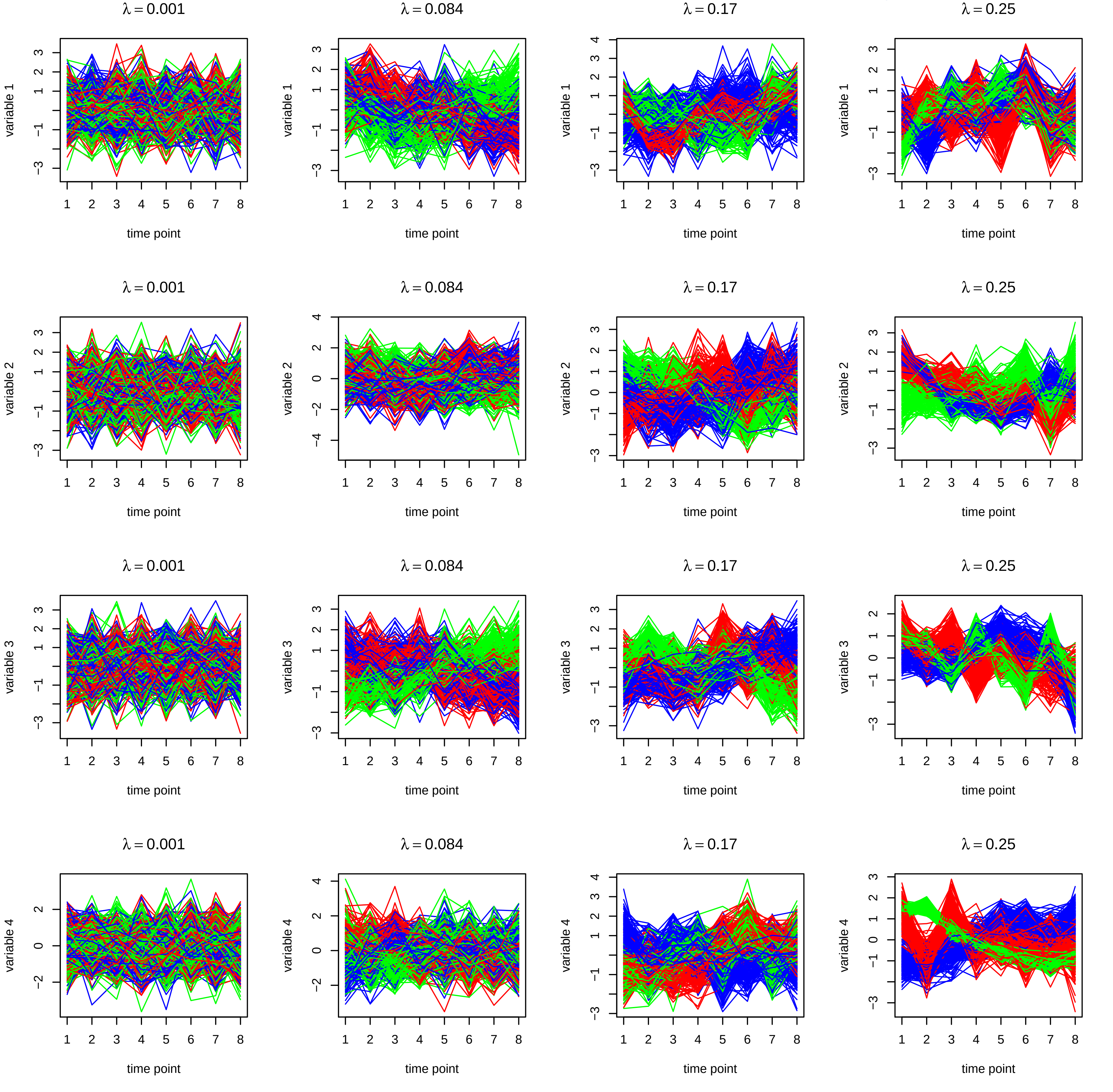

Click here to
access/download;Figure;PPMI__confounders.pdf

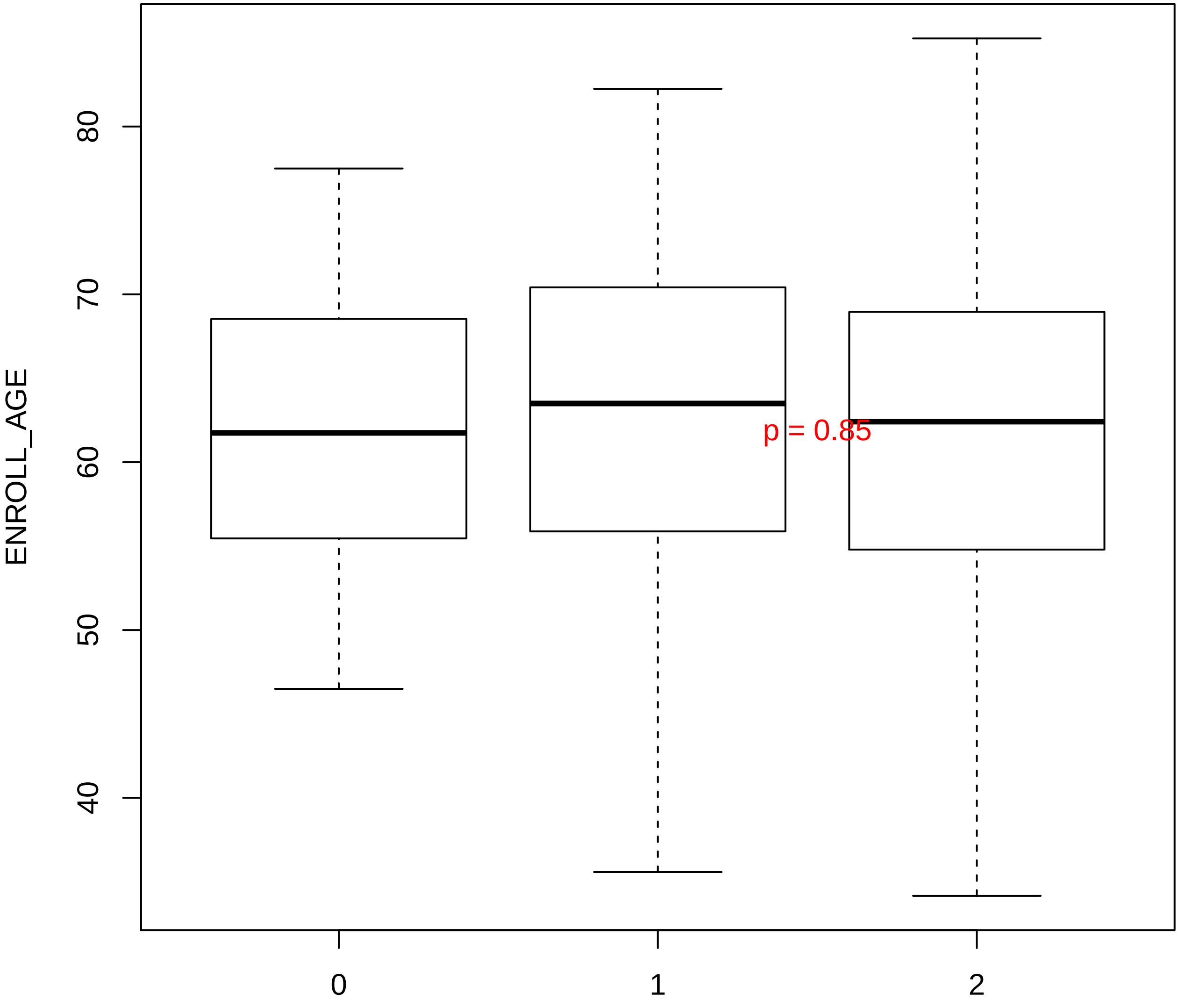

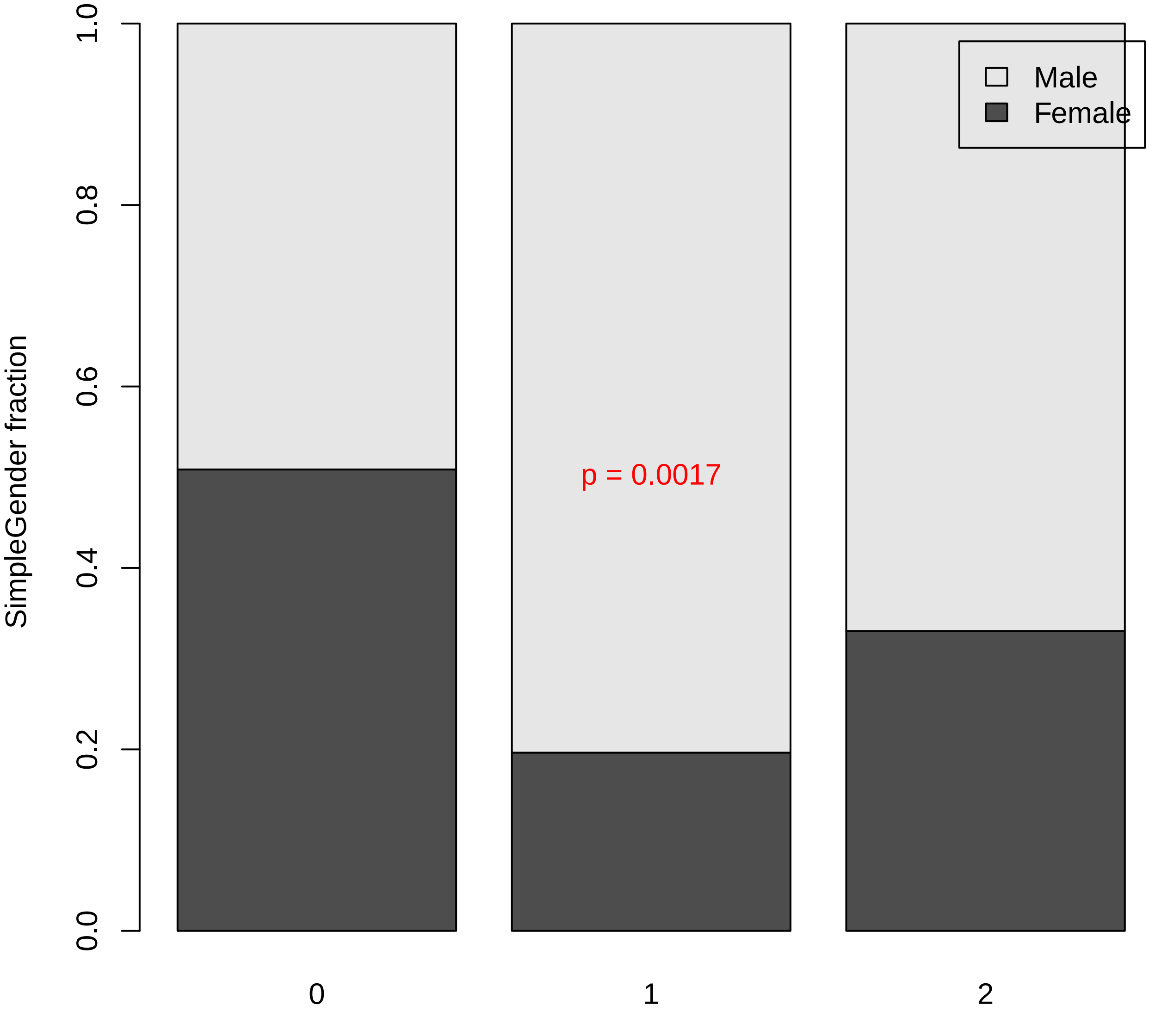

1.0
0.8
0.6
0.4
0.2
0.0
SimpleGender fraction
Male
Female
p = 0.0017
0
1
2

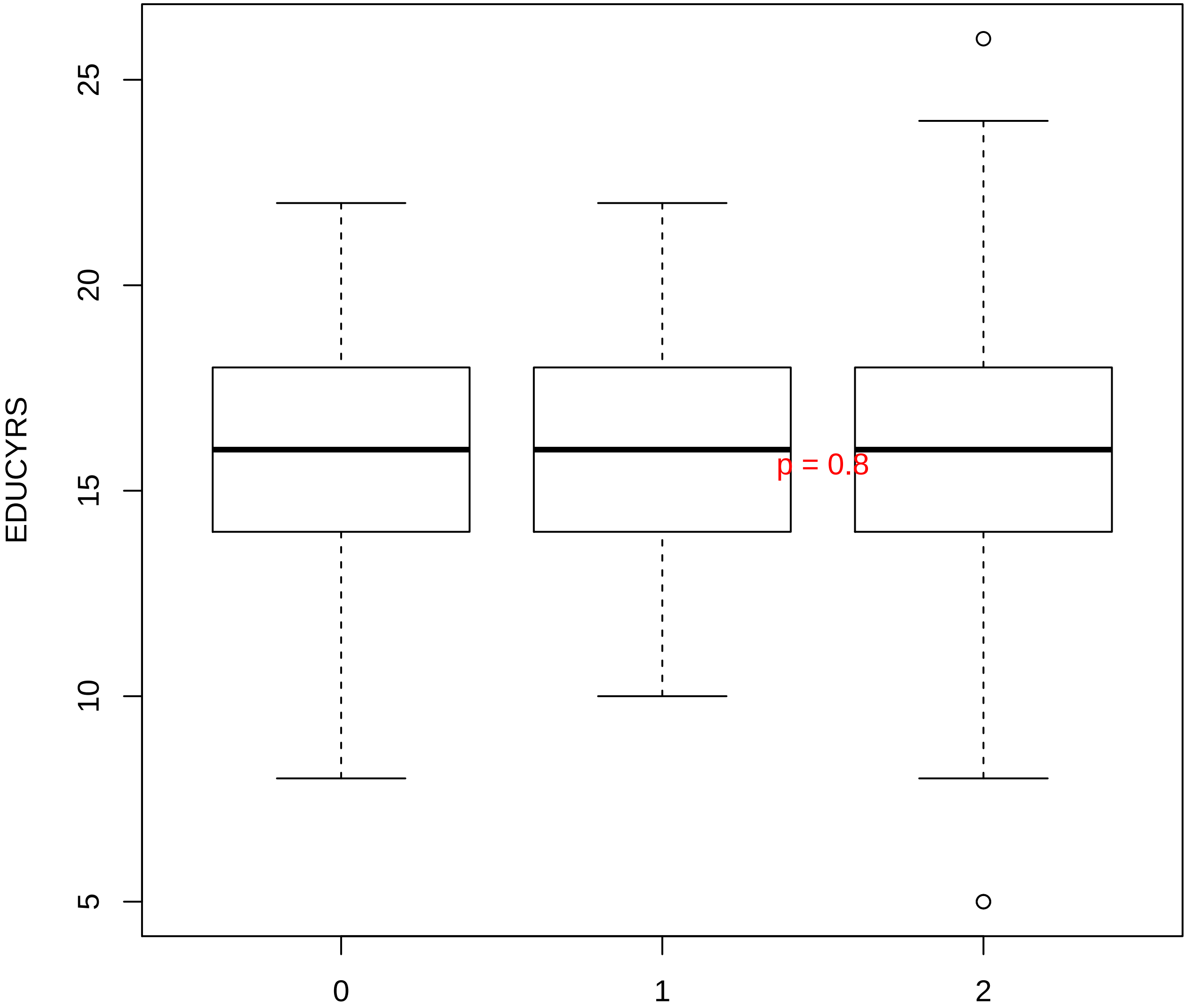
EDUCYRS
25
20
15
10
5
0
1
2
p = 0.8

Click here to access/download;Figure;PPMI__control_variables.p

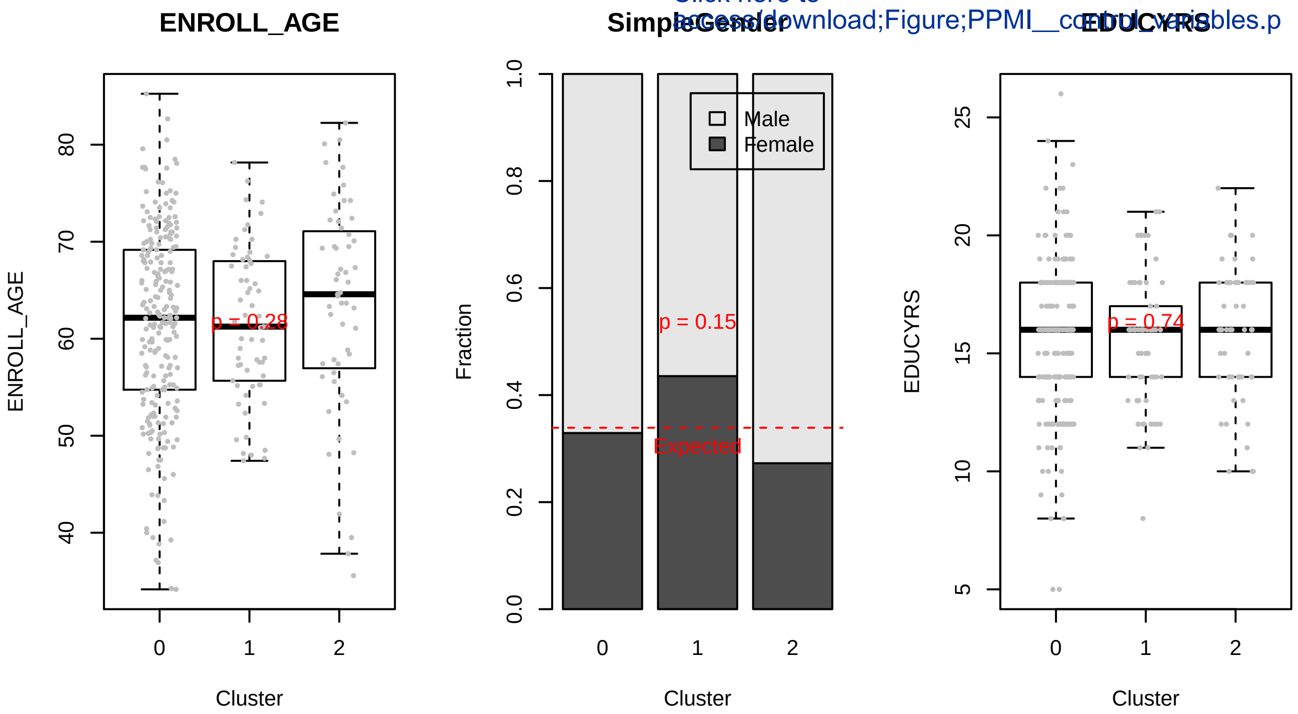

Click here to access/download;Figure;PPMI__number_of_clusters

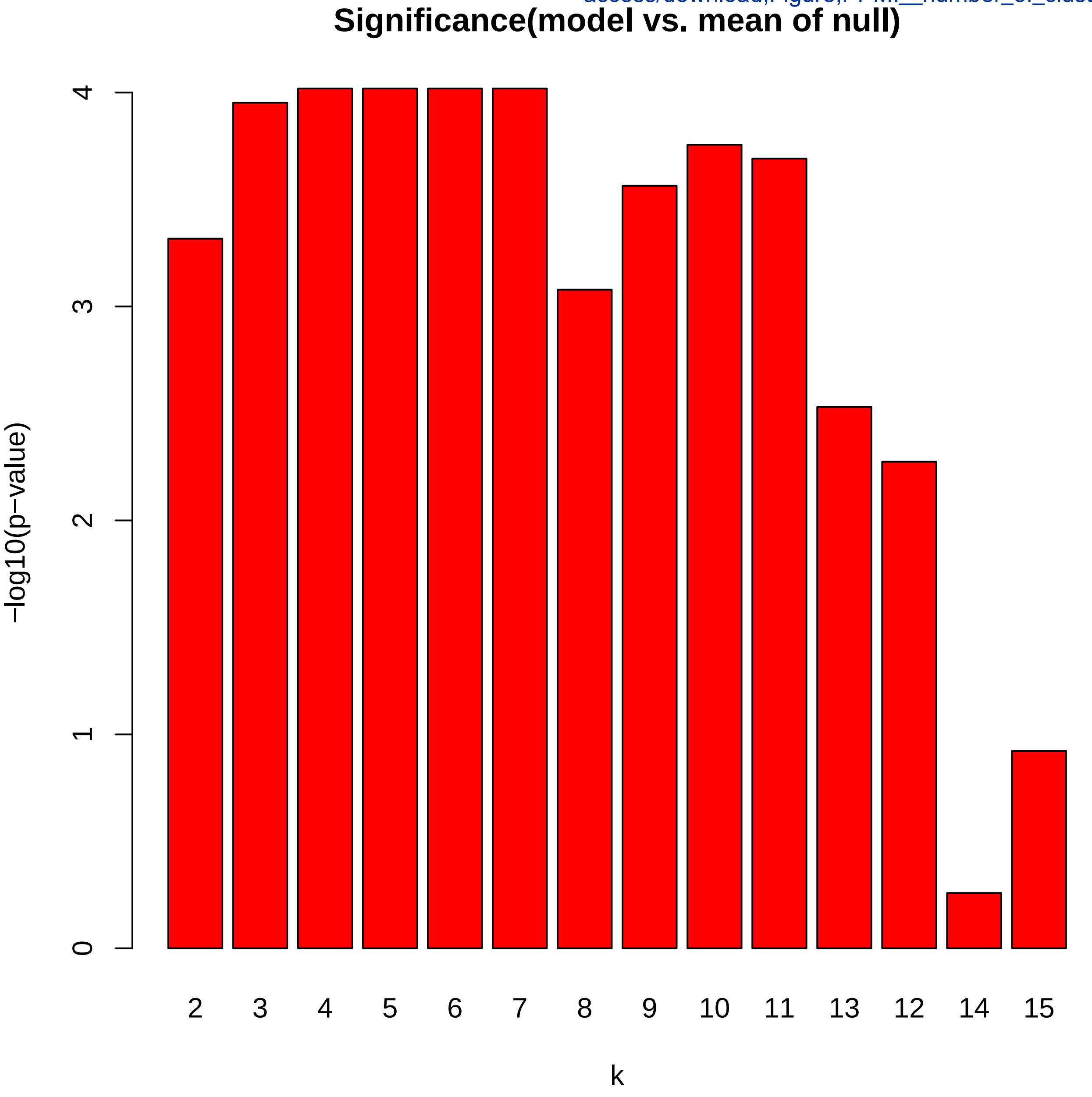

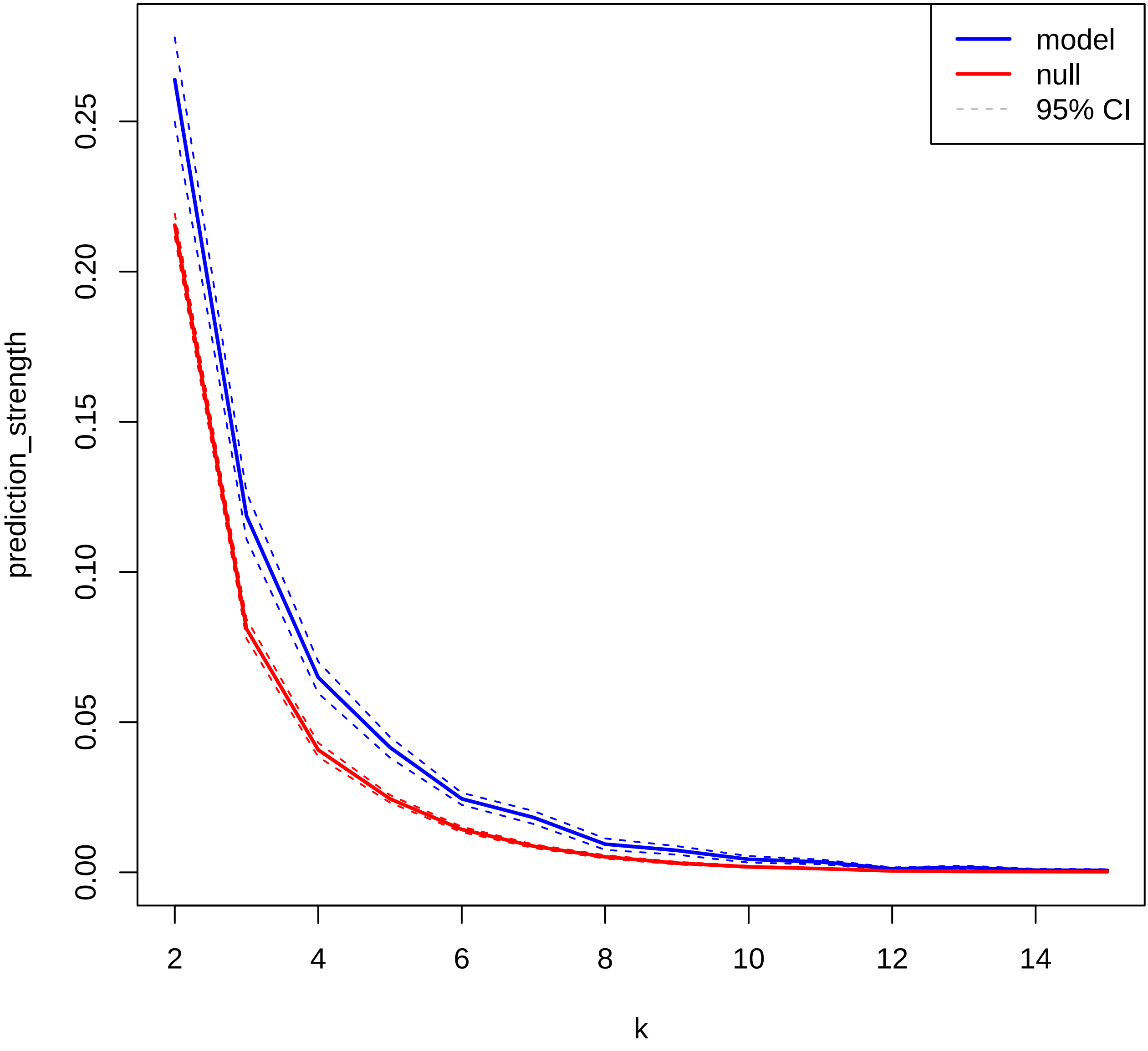
model
null
95% CI
prediction_strength
0.00
0.05
0.10
0.15
0.20
0.25
k
2
4
6
8
10
12
14

Click here to access/download;Figure;PPMI__PPMI.pdf

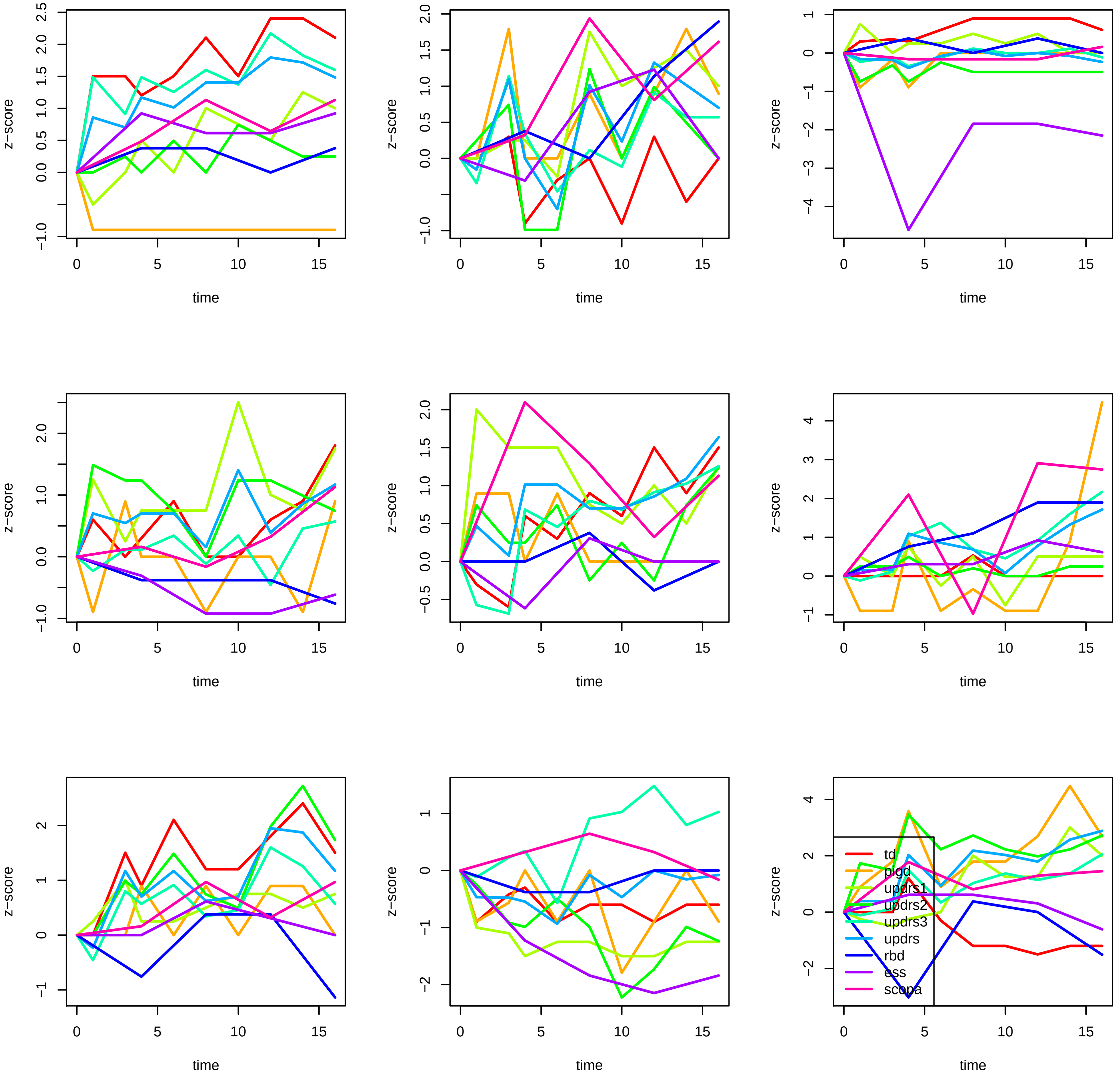

Click here to access/download;Figure;PPMI__variables_cognitive_assessment.pdf

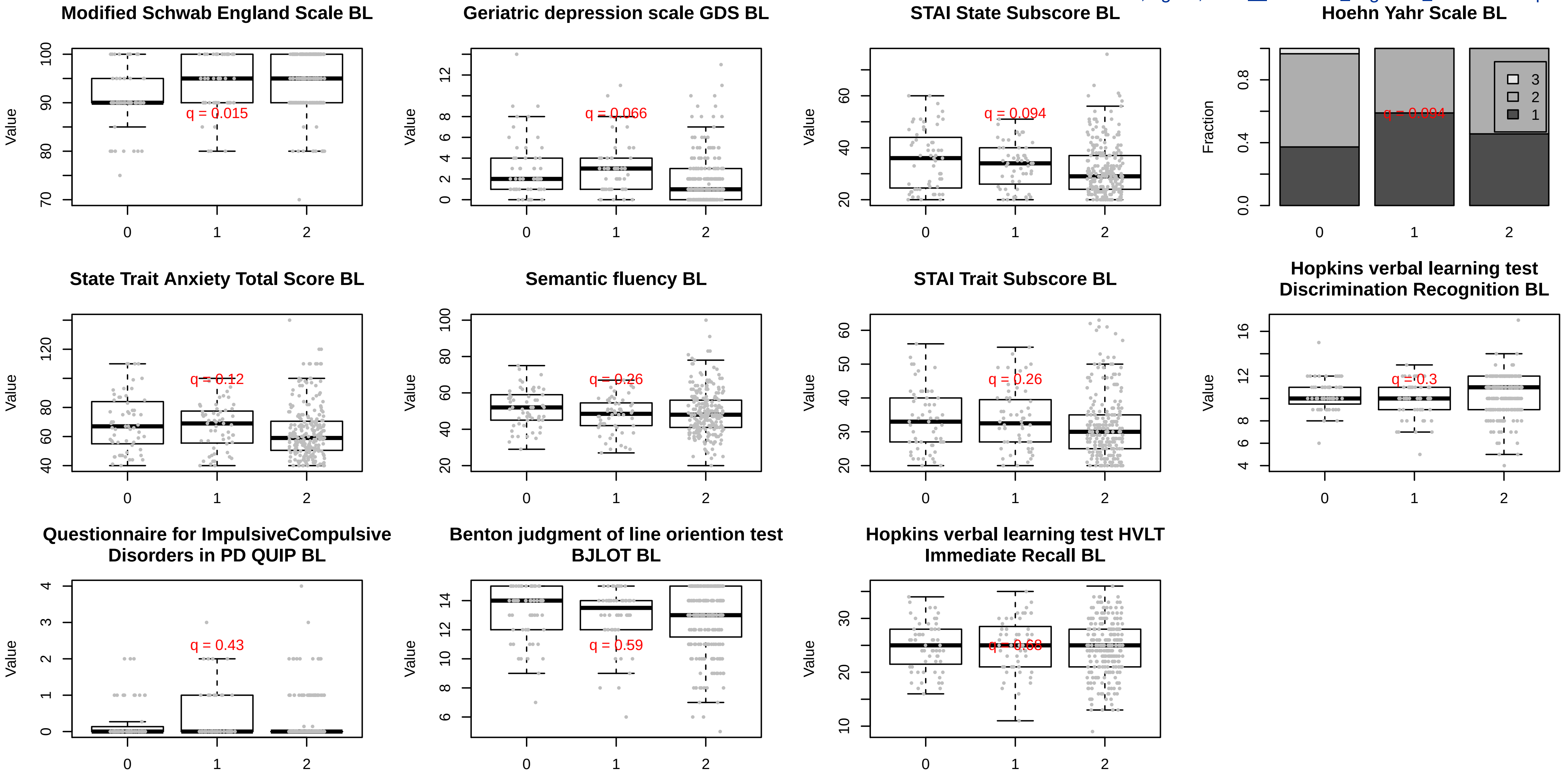

Click here to access/download;Figure;PPMI__variables_marker.pdf

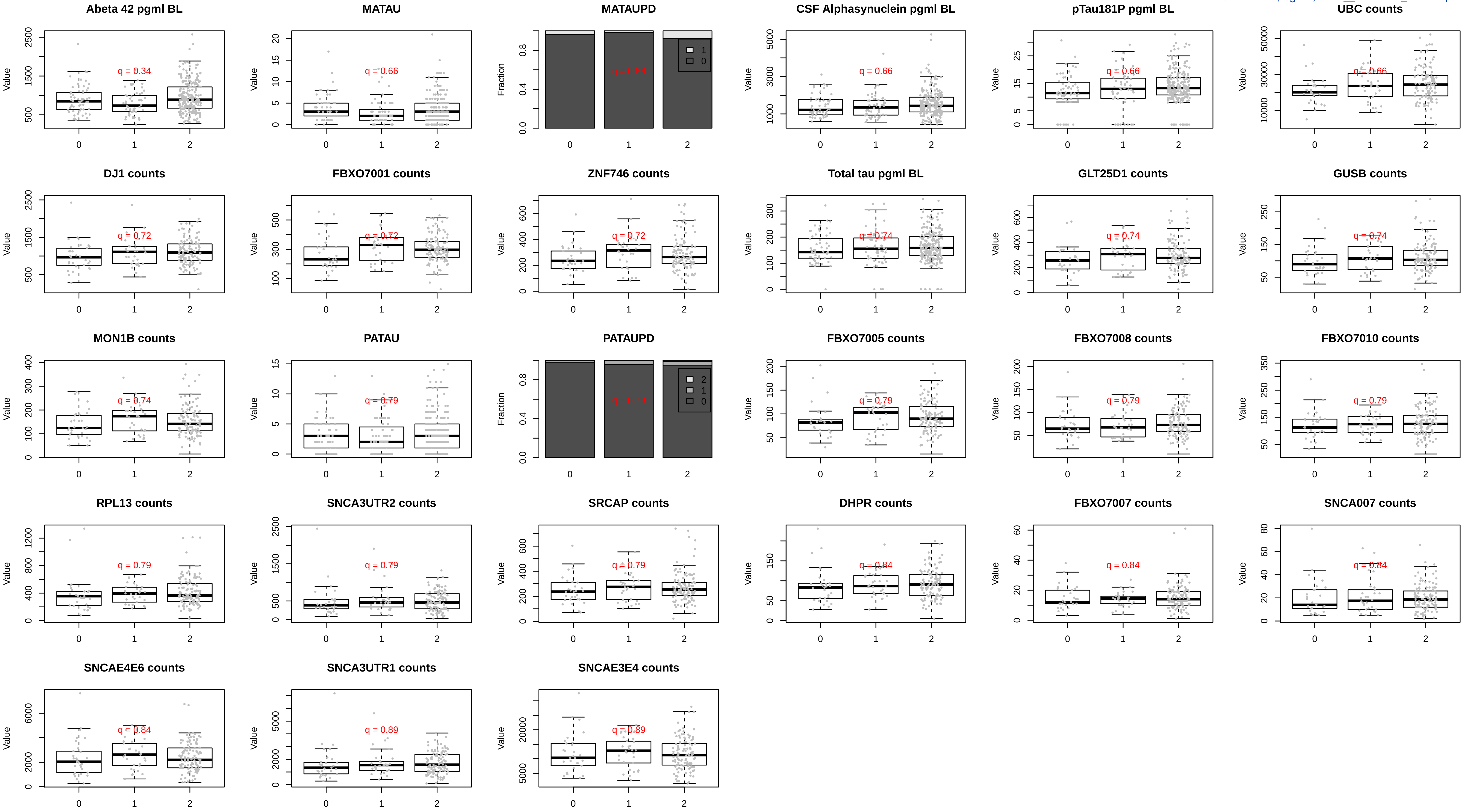

Click here to access/download;Figure;PPMI__variables_patient_characteristic.pdf

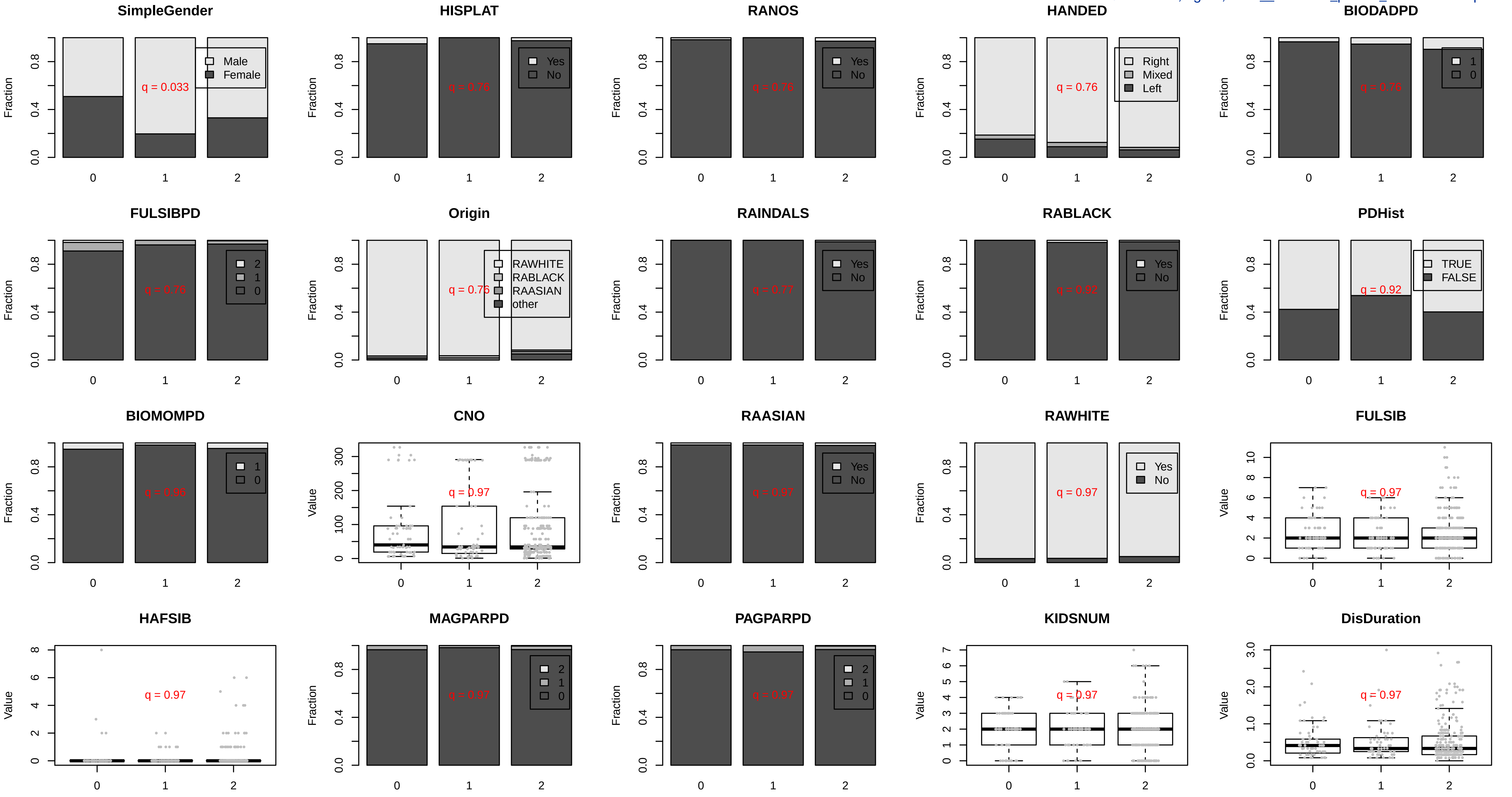

Click here to access/download;Figure;PPMI_variables_series_brain.pdf

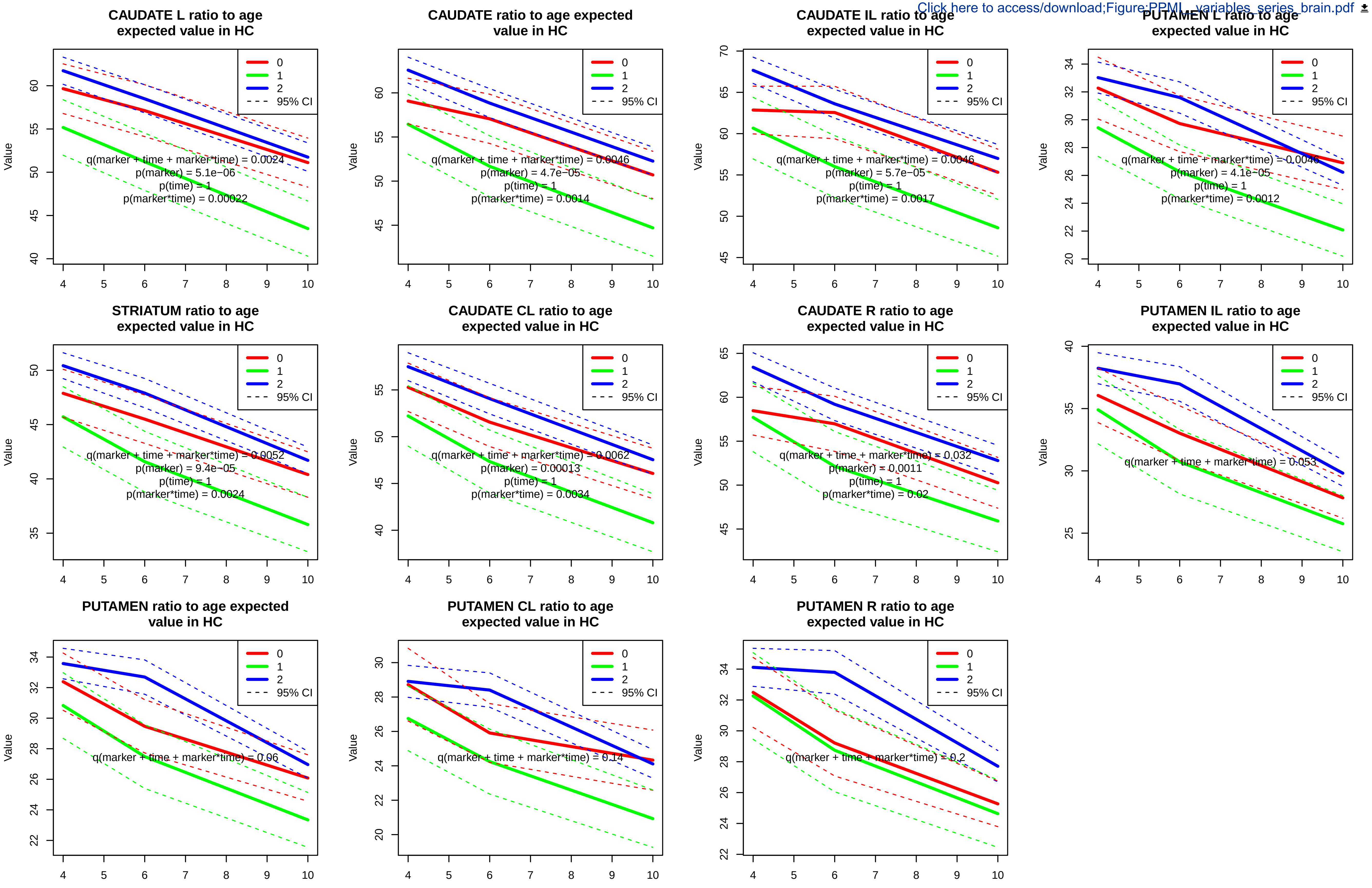

Click here to access/download;Figure;PPMI__variables_series_cognitive_assessment.pdf

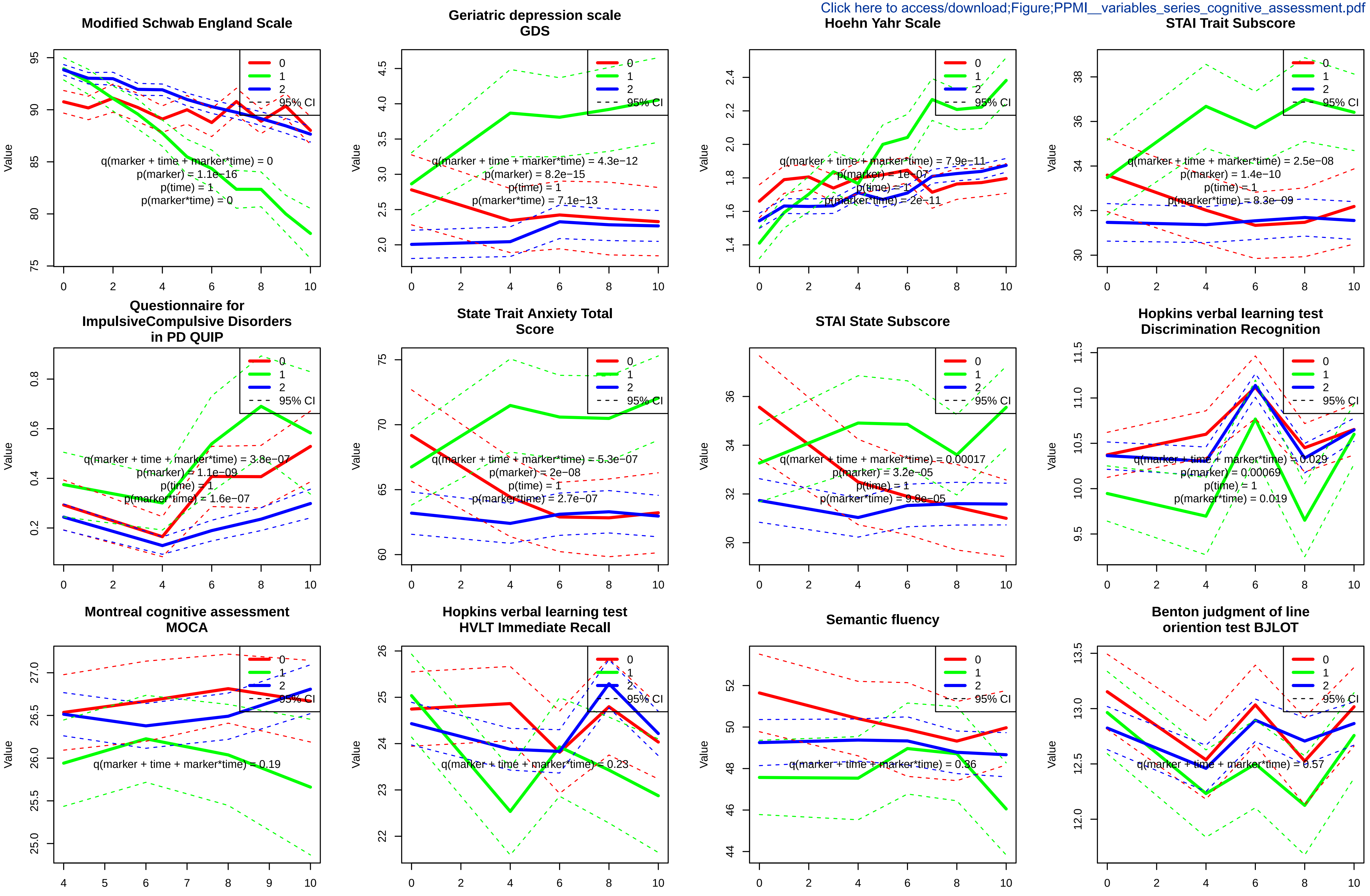

Click here to access/download;Figure;PPMI_variables_series_m

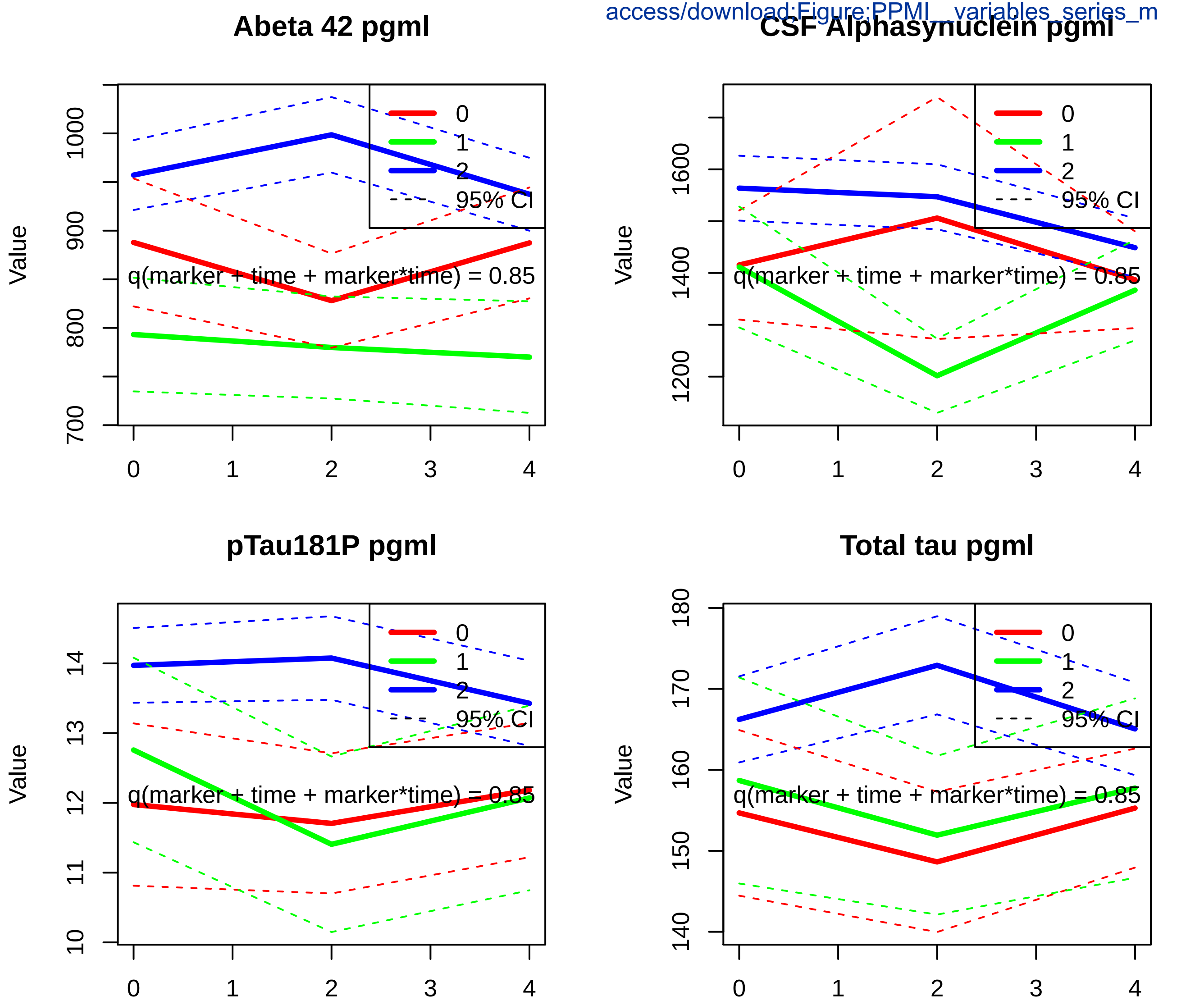

Click here to access/download;Figure;ADNI__ADNI.pdf

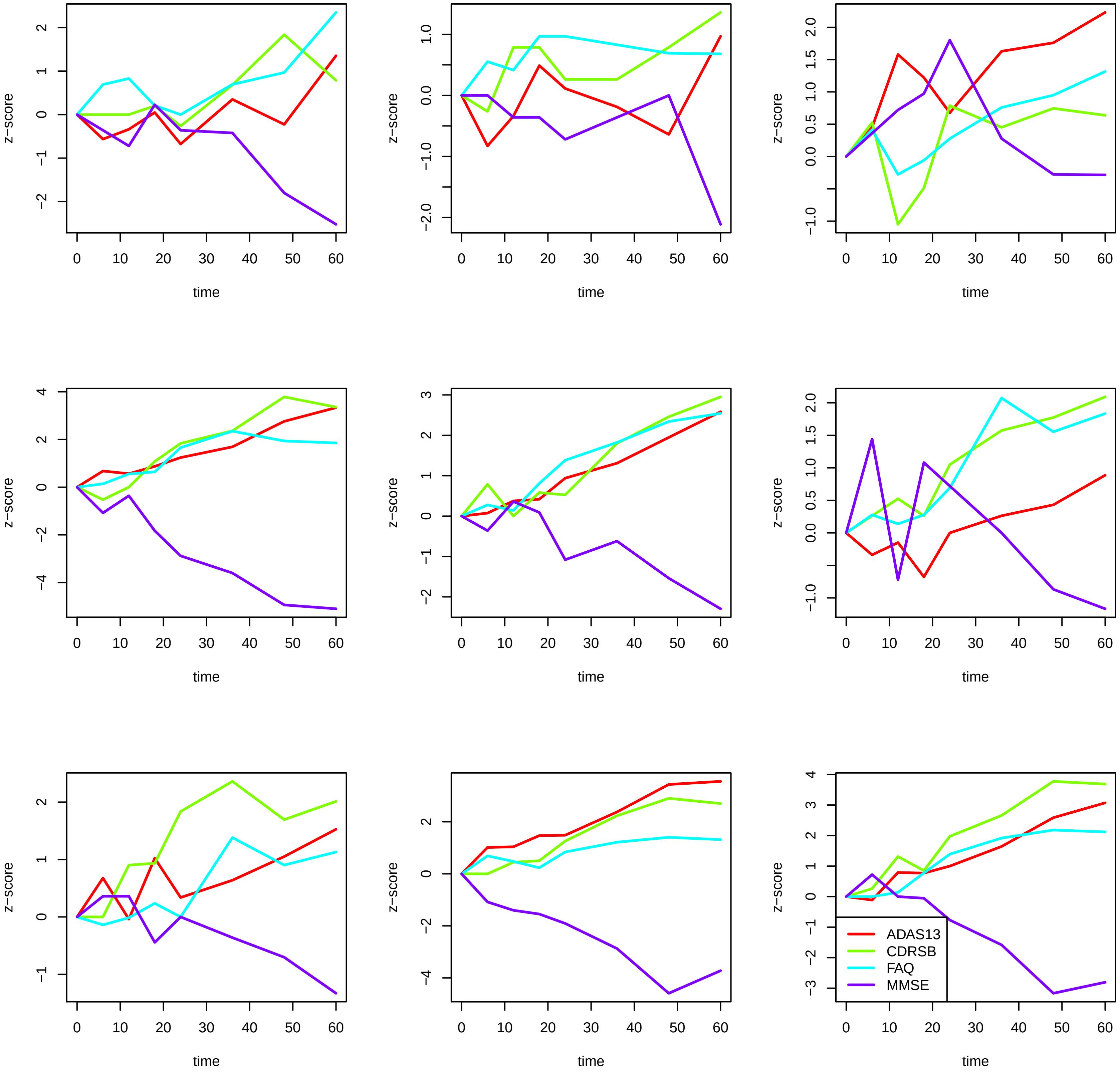

Click here to
access/download;Figure;ADNI__confounders.pdf

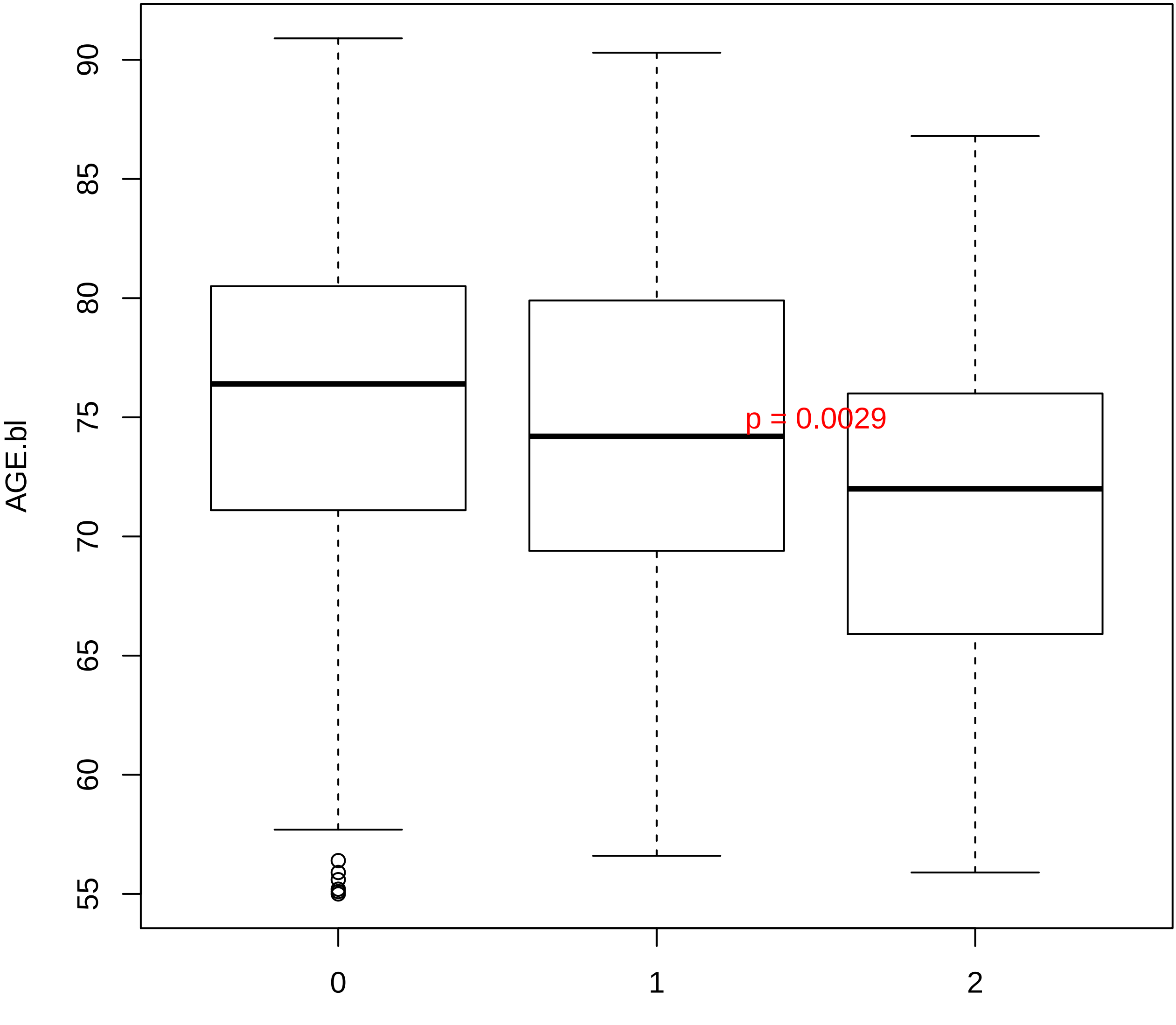

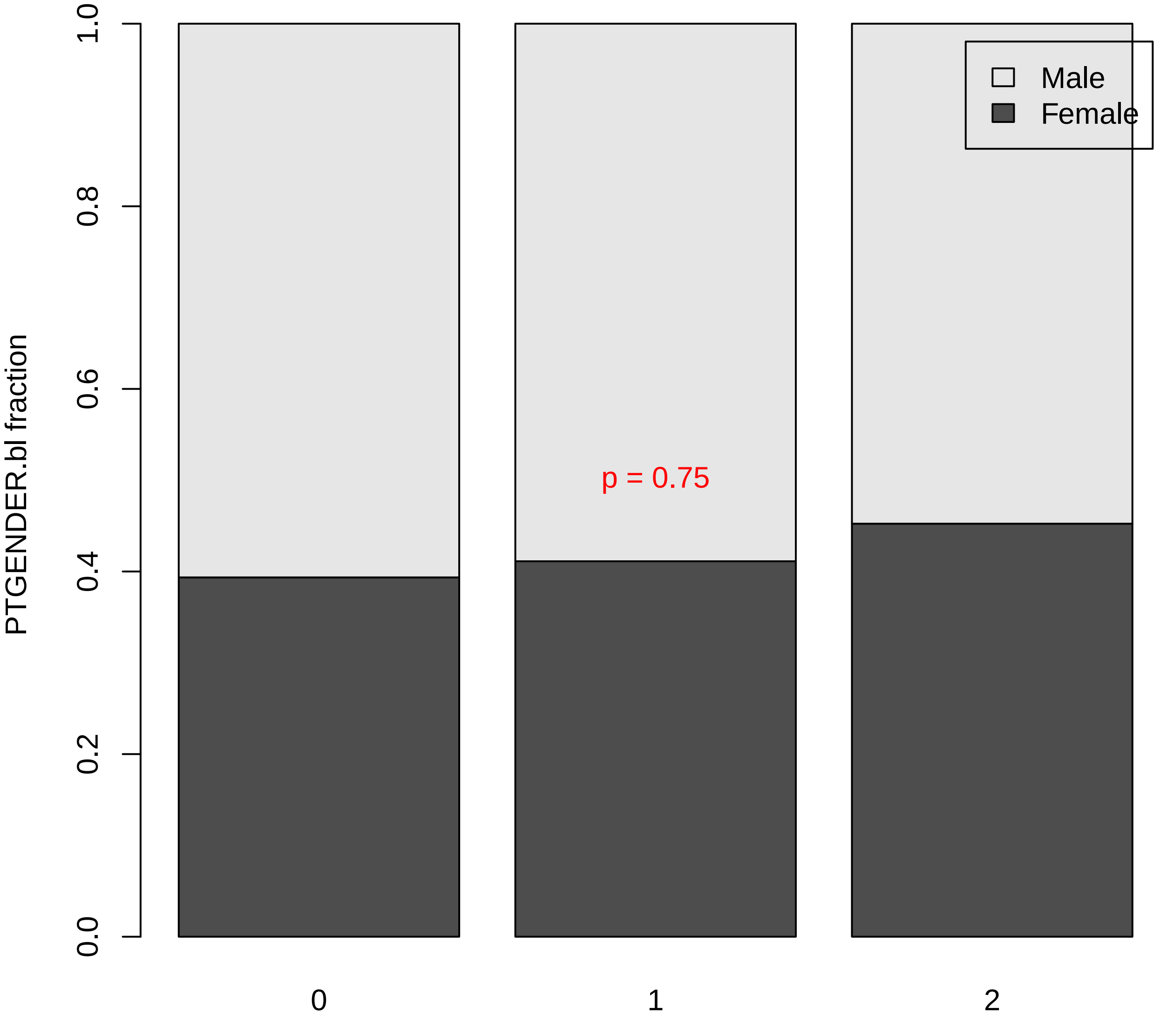

PTGENDER.bl fraction
0.0
0.2
0.4
0.6
0.8
1.0
0
1
2
Male
Female
p = 0.75

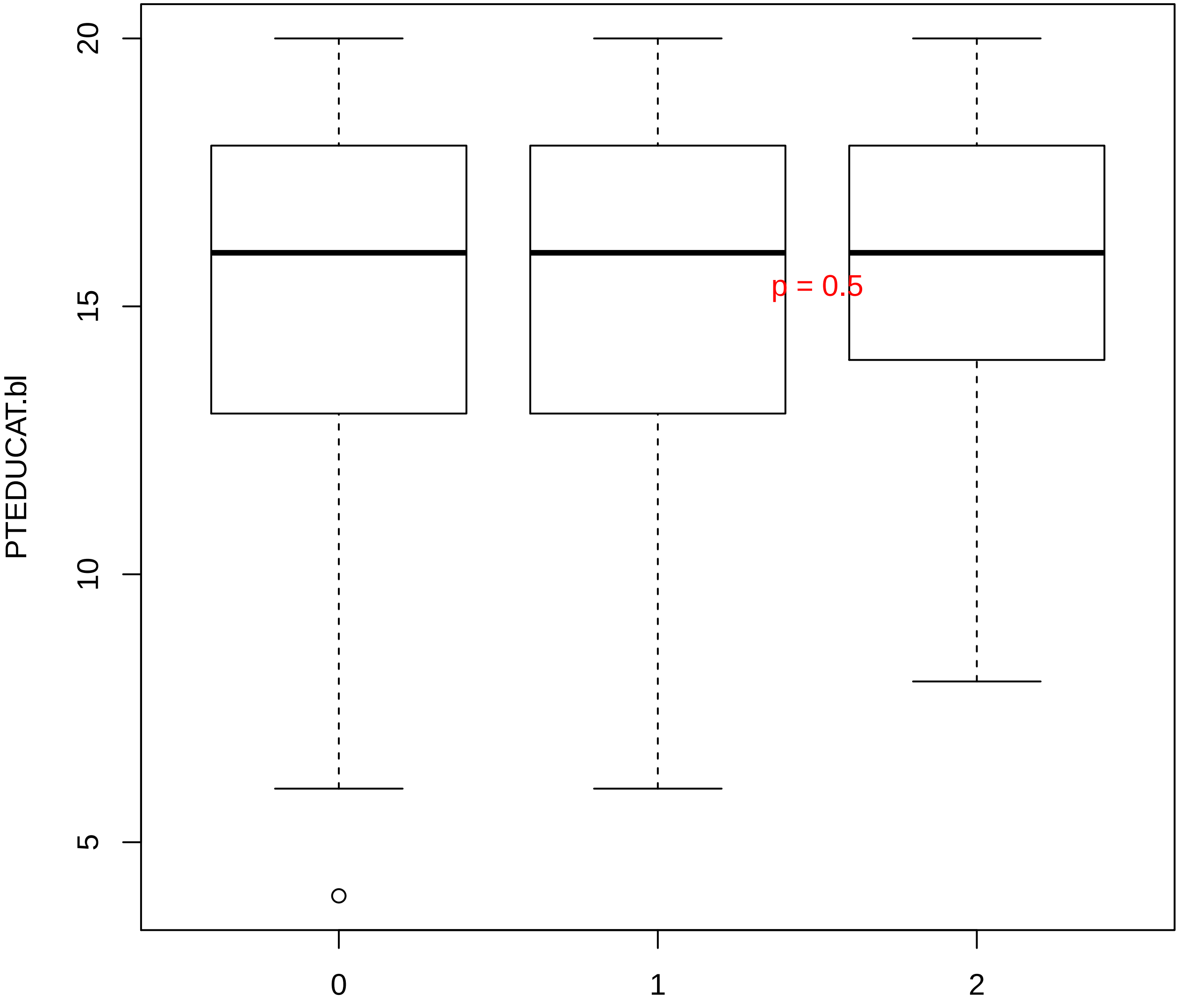

PTEDUCAT.bl
20
15
10
5
0
1
2
p = 0.5

Click here to access/download;Figure;ADNI__control_variables.p

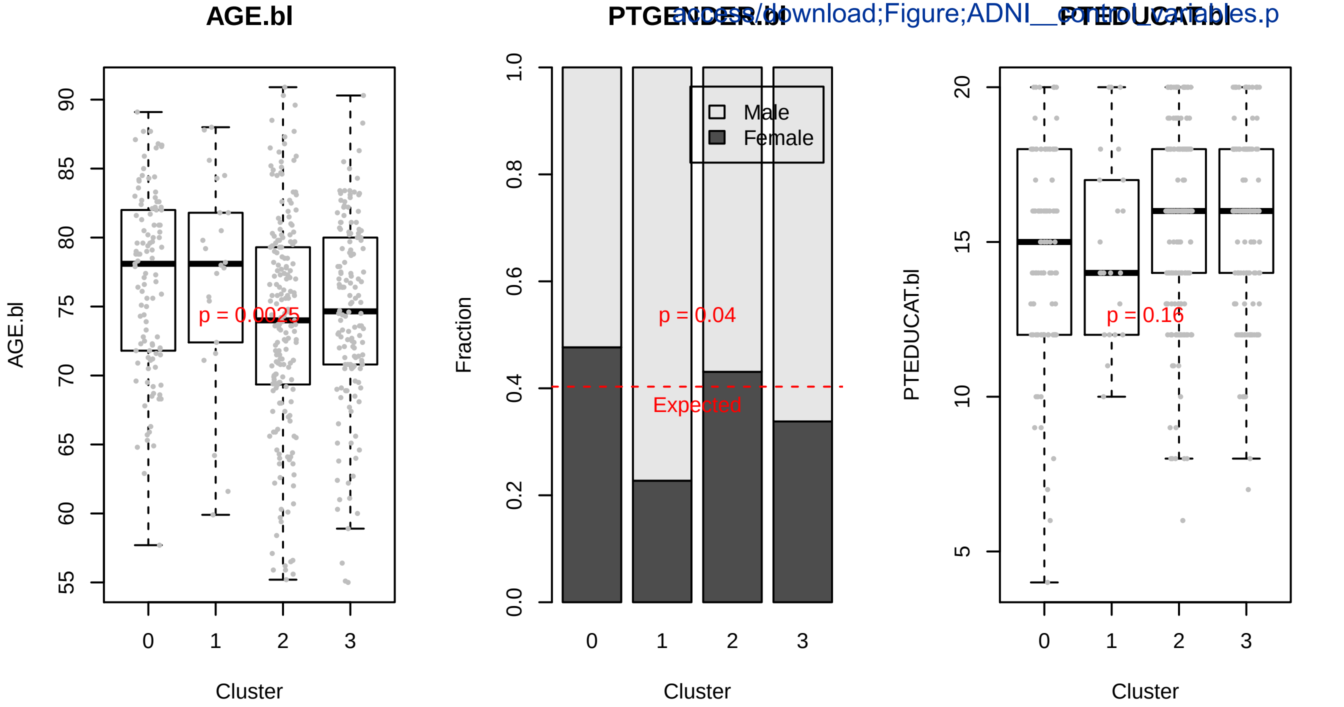

Click here to
access/download;Figure;ADNI__number_of_clusters

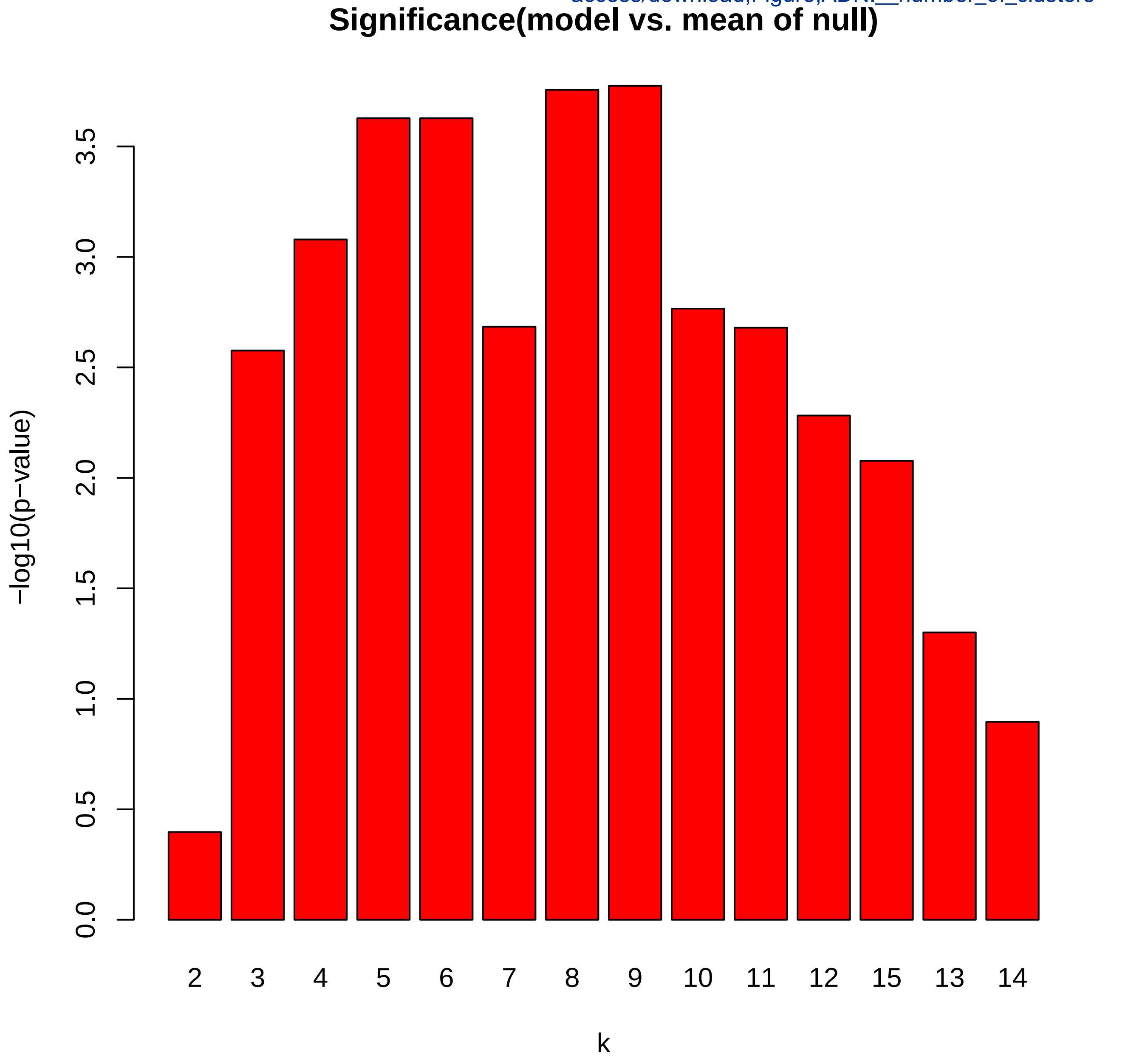

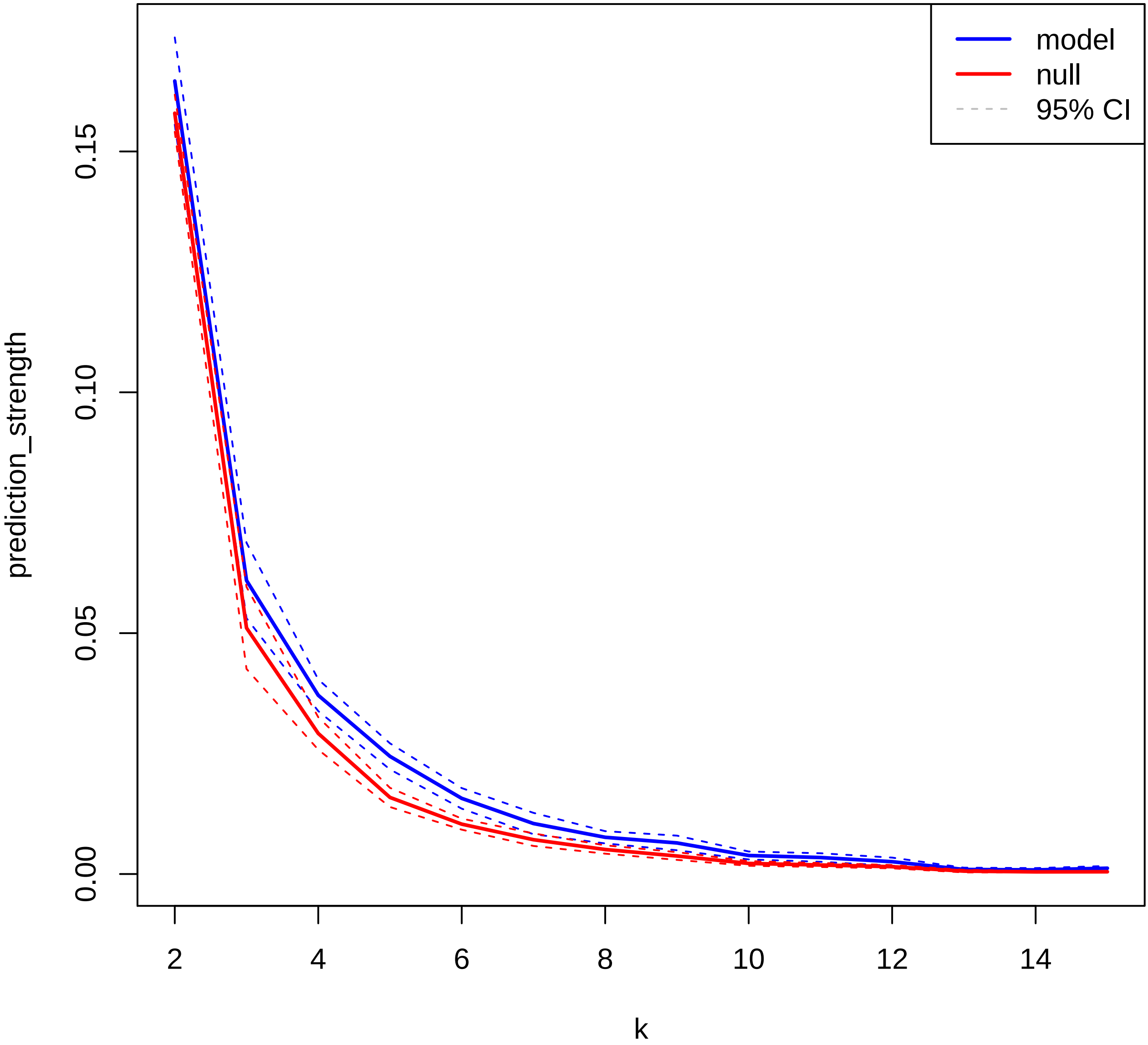
model
null
95% CI
prediction_strength
0.00
0.05
0.10
0.15
2
4
6
8
10
12
14
k

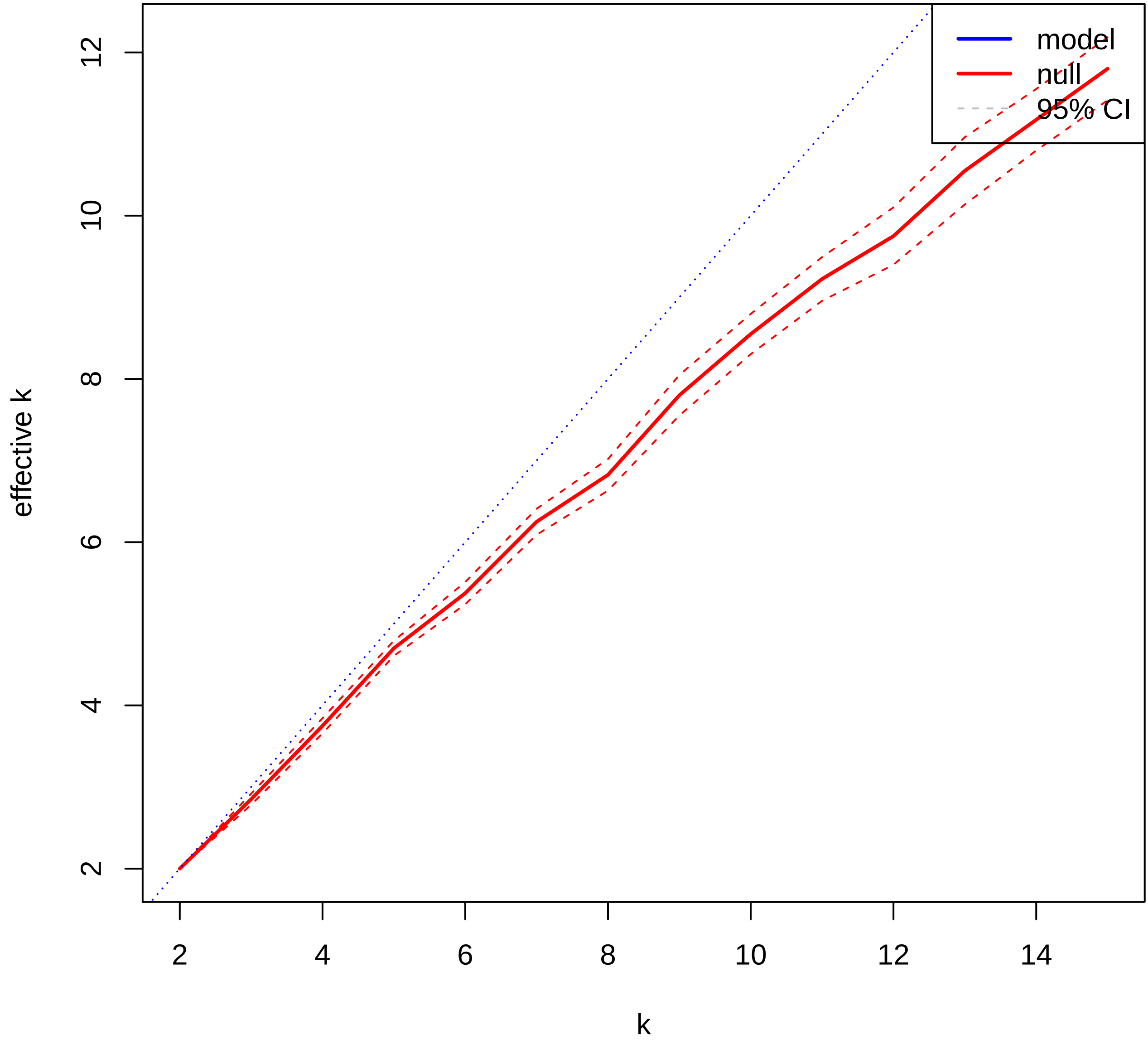
model
null
95% CI
effective k
k
2
4
6
8
10
12
14

Click here to access/download;Figure;ADNI__variables_brain_level1.

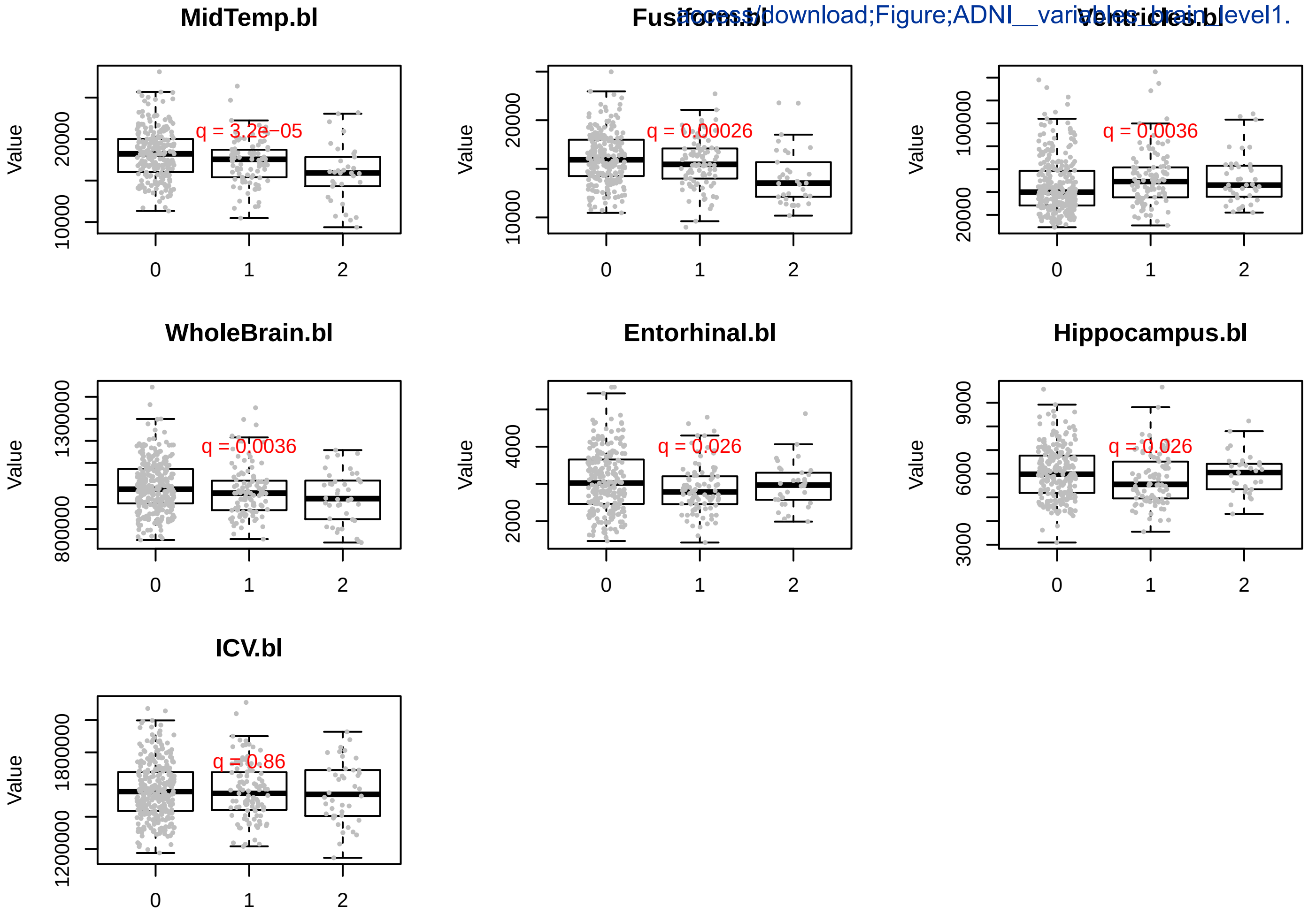

Click here to access/download
Supplementary Material
supplemental_material.tex

Click here to access/download
Supplementary Material
VaDER_GigaScience__supplementals.pdf

Personal Cover

Click here to access/download;Personal Cover;letter_to_the_editor__revision.pdf

Prof. Dr. Holger Fröhlich
University of Bonn
Bonn-Aachen International Center for IT
Endenicher Allee 19a
53115 Bonn, Germany

To the Editor in Chief
GigaScience

Dear Editor,

Revision of our manuscript "Deep learning for clustering of multivariate clinical patient trajectories with missing values"

Thank you for giving us the opportunity to revise our manuscript. We appreciated the reviewers' constructive comments, and we believe that their suggestions have led to a more convincing presentation of our method in the manuscript.

In this revised manuscript, we were able to address all of the reviewers' requests, which were mainly related to the technical validation of the clustering method VaDER that we propose in the manuscript. More specifically, we (1) included additional methods to compare to VaDER, (2) included additional analyses comparing VaDER's implicit imputation with pre-imputation and (3) included analyses of a number of real-world benchmark datasets.

We have compiled a detailed rebuttal letter providing point-by-point answers to each of the reviewers' concerns.

We look forward to hearing from you.

Sincerely yours,

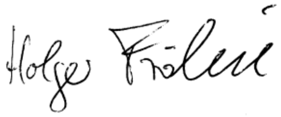

Holger Fröhlich
