# Supplementary material for: Deep learning for clustering of multivariate clinical patient trajectories with missing values
Source: Gigascience. 2019 Nov 15;8(11):giz134. doi: 10.1093/gigascience/giz134 (PMC6857688; doi:10.1093/gigascience/giz134)
Supplement: giz134_Supplemental_Files [file giz134_supplemental_files.zip › VaDER_GigaScience__supplementals.pdf]

GigaScience, 2017, 1–16

doi: xx.xxxx/xxxx
Manuscript in Preparation
Supplemental Material

SUPPLEMENTAL MATERIAL

Deep learning for clustering of multivariate clinical patient trajectories with missing values

Johann de Jong[1,*], Mohammad Asif Emon[2,3], Ping Wu[4], Reagon Karki[2,3], Meemansa Sood [2,3], Patrice Godard[6], Ashar Ahmad[3], Henri Vrooman[5], Martin Hofmann-Apitius[2,3] and Holger Fröhlich[1,3,*]

[1]UCB Biosciences GmbH, 40789 Monheim, Germany and [2]Fraunhofer Institute for Algorithms and Scientific Computing, 53754 Sankt Augustin, Germany and [3]Bonn-Aachen International Center for IT, University of Bonn, 53115 Bonn, Germany and [4]UCB Pharma, Slough SL1 3WE, United Kingdom and [5]Erasmus MC, University Medical Center Rotterdam, Departments of Radiology and Medical Informatics, PO Box 2040 3000 CA Rotterdam, Netherlands and [6]UCB Pharma, 1420 Braine-l'Alleud, Belgium

[*]johann.dejong@ucb.com; holger.froehlich@ucb.com
[*]johann.dejong@ucb.com; holger.froehlich@ucb.com

Compiled on: October 19, 2019.
Draft manuscript prepared by the author.

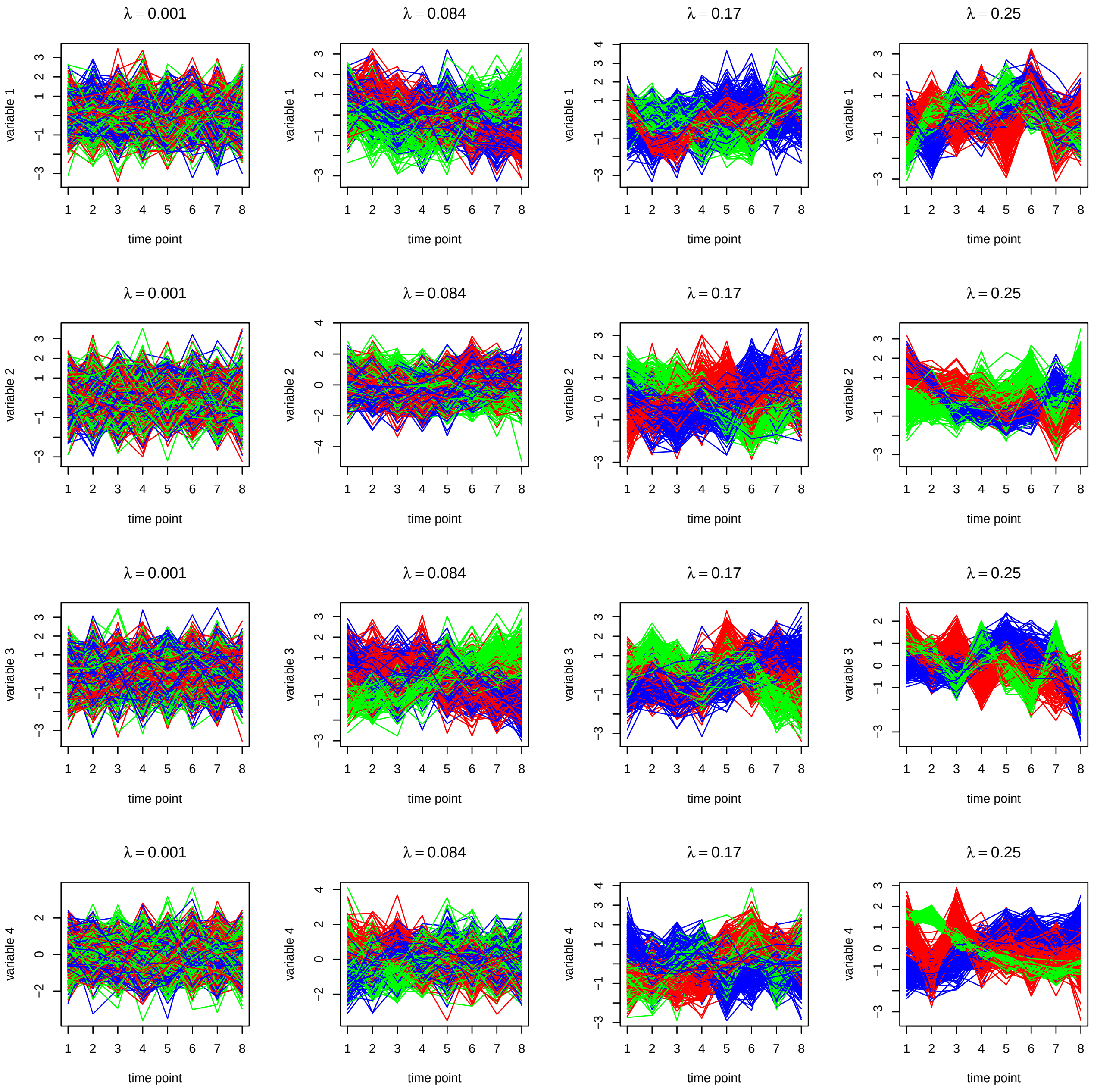

Figure 1. Multivariate short time series data simulated using vector autoregressive processes, for 4 variables, 8 time points and 3 clusters, and different levels of the similarity parameter λ.

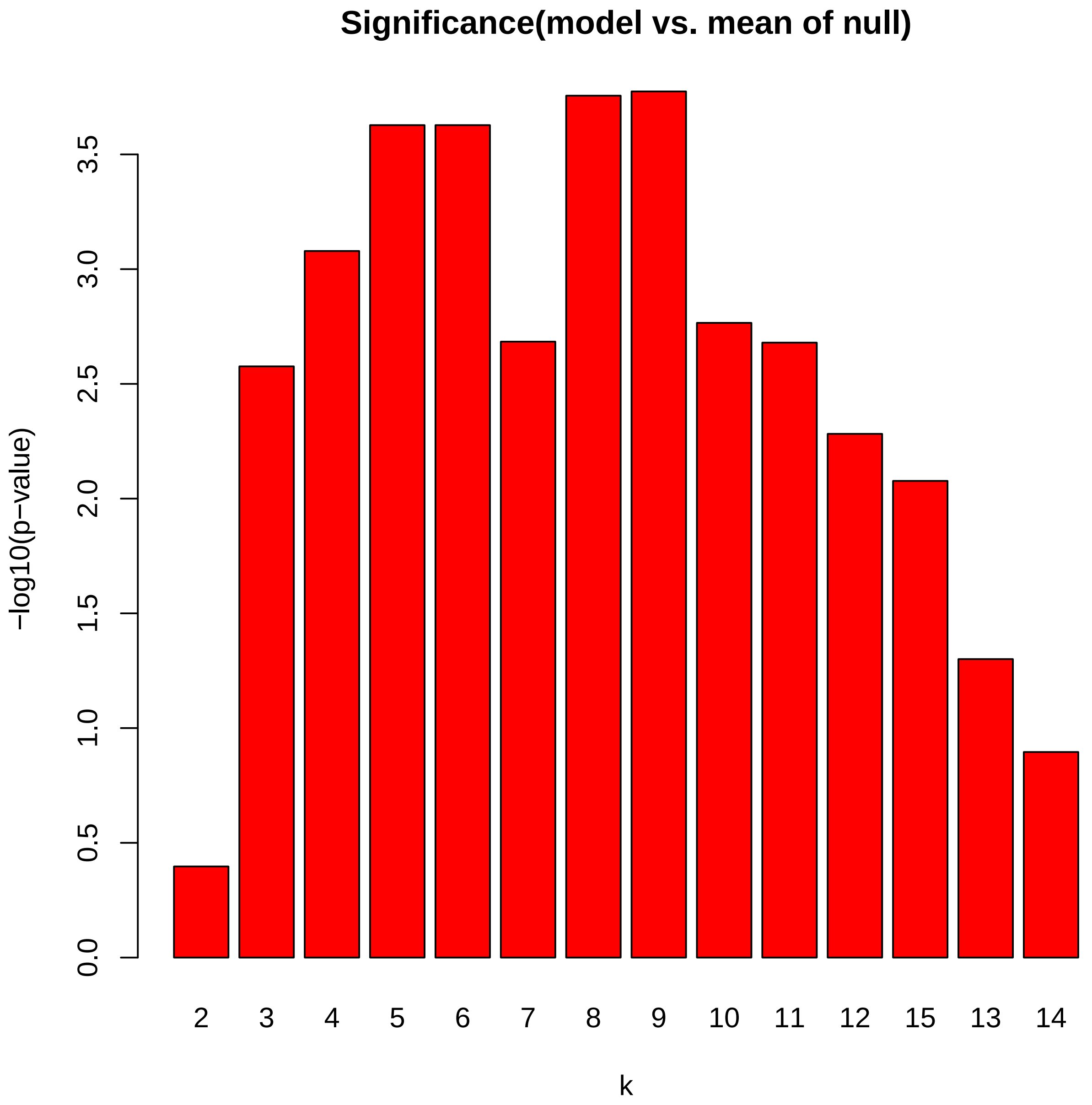

Figure 2. ADNI: prediction strength of VaDER for each *k* (blue) and the corresponding permutation-based null distribution.

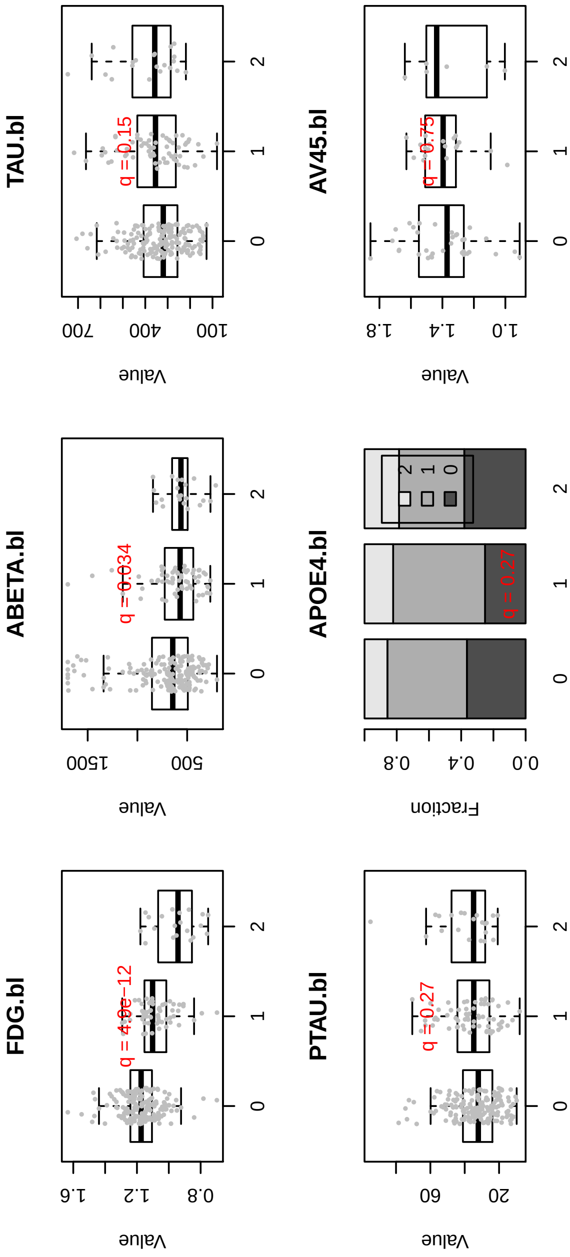

Figure 3. ADNI: associations of the VaDER clustering with a wide range of other baseline data available from ADNI.

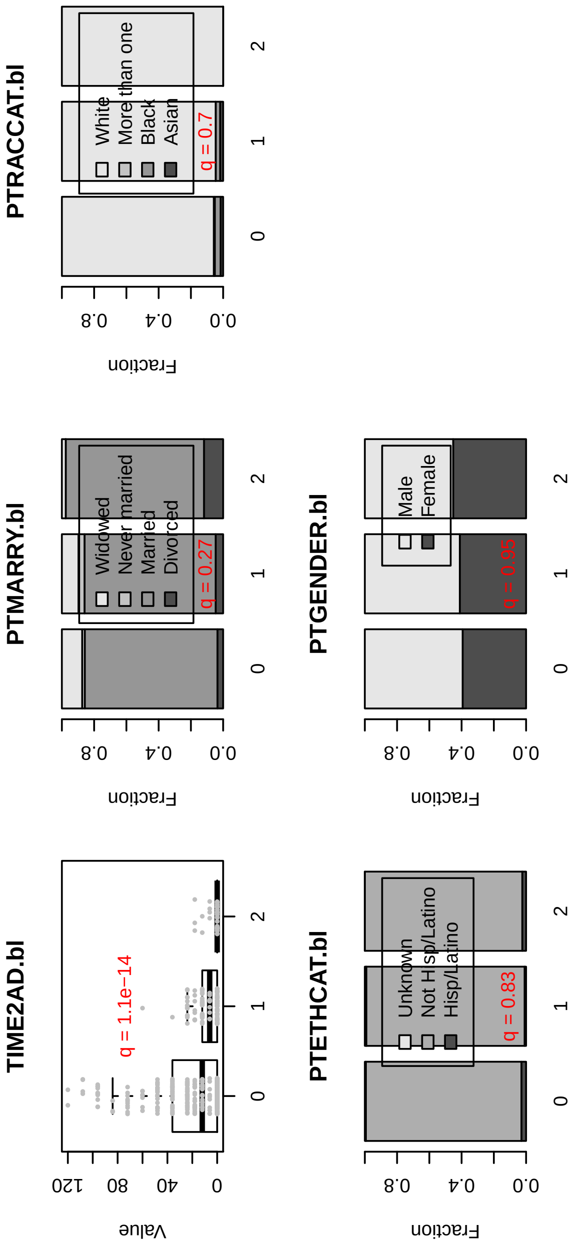

Figure 4. ADNI: associations of the VaDER clustering with a wide range of other baseline data available from ADNI.

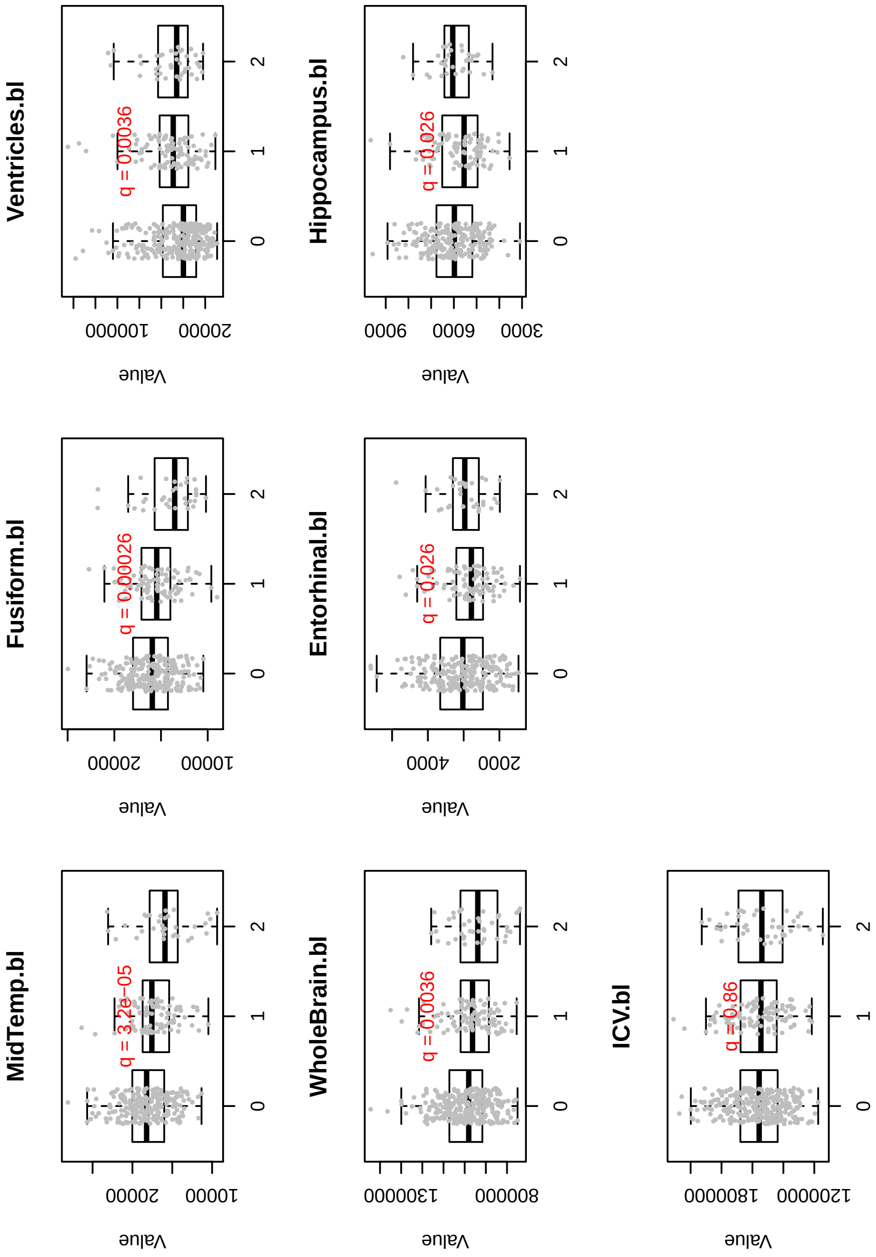

Figure 5. ADNI: associations of the VaDER clustering with a wide range of other baseline data available from ADNI.

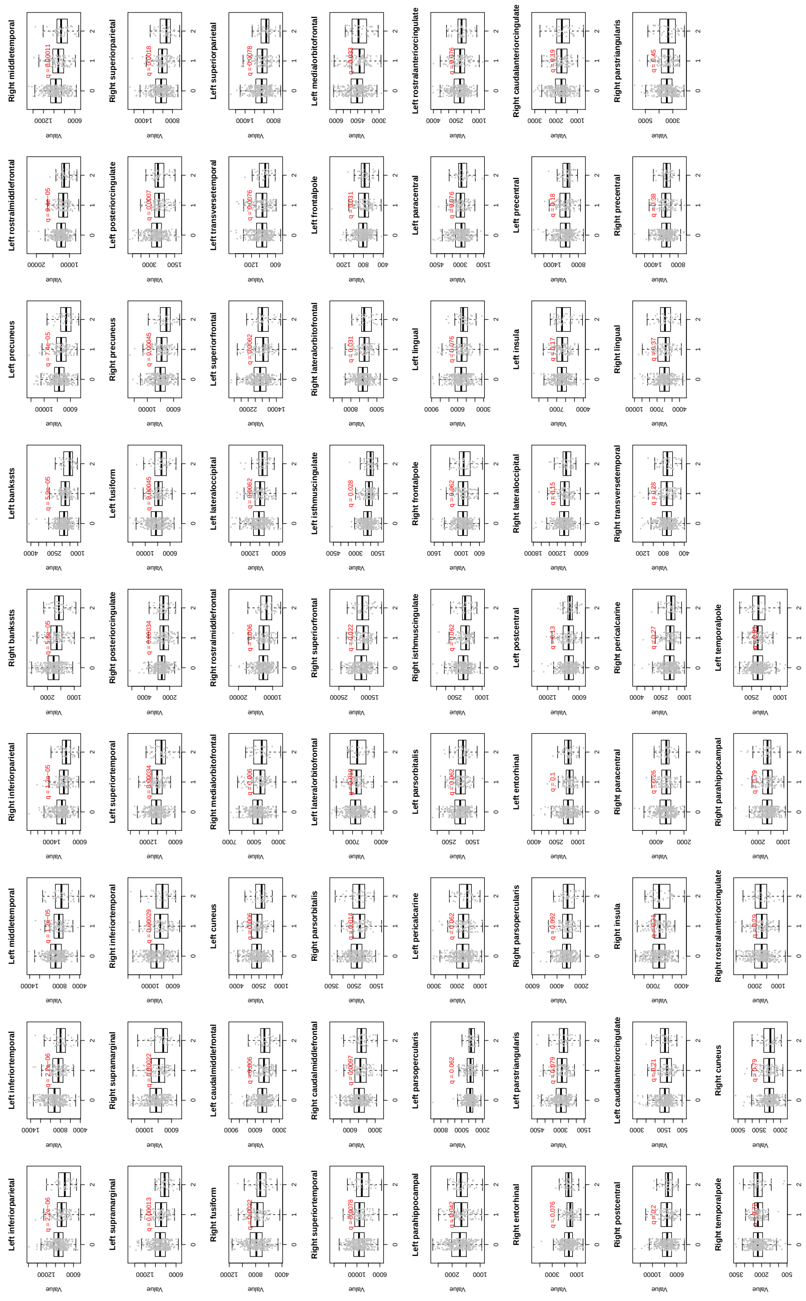

Figure 6. ADNI: associations of the VaDER clustering with a wide range of other baseline data available from ADNI.

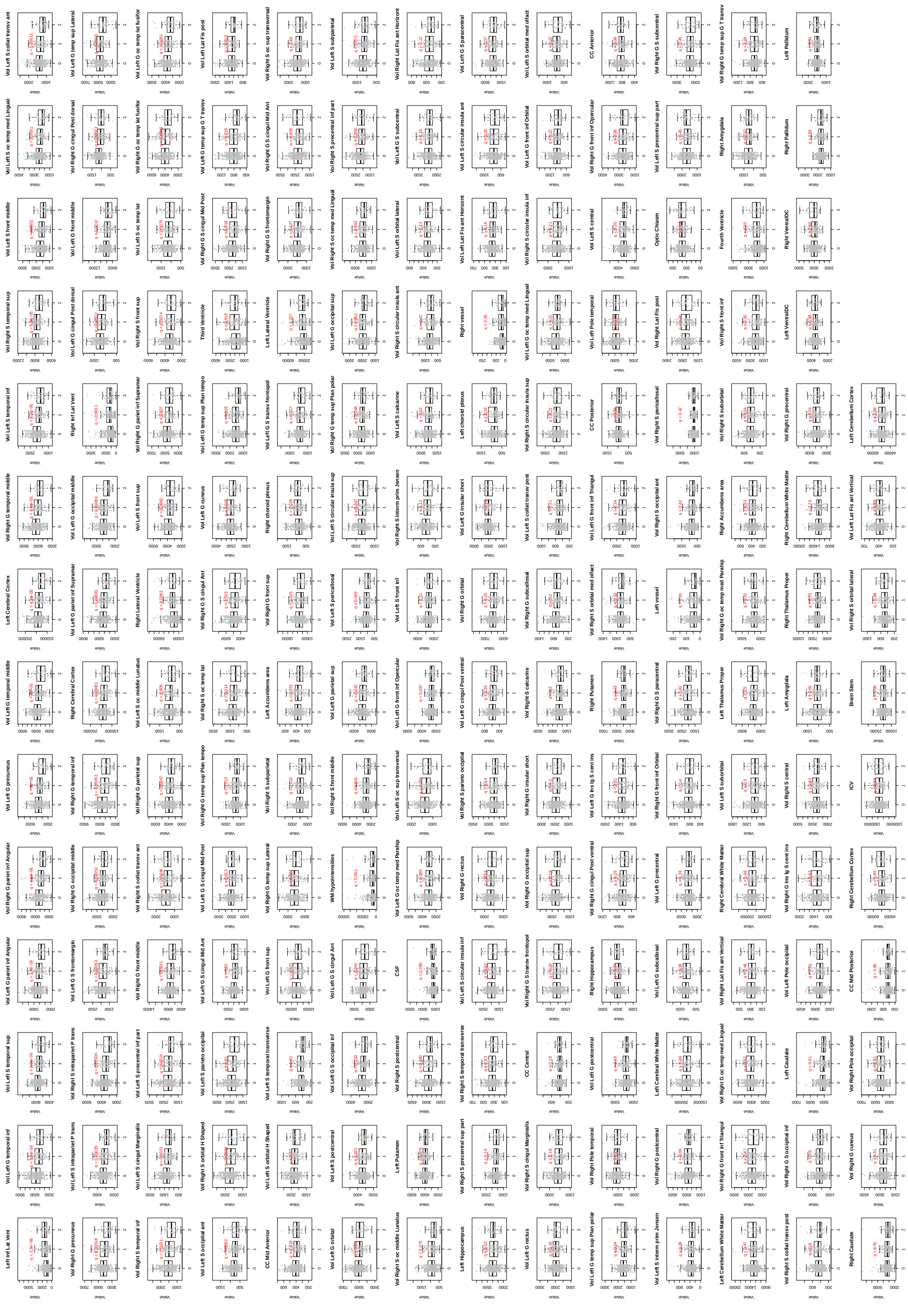

Figure 7. ADNI: associations of the VaDER clustering with a wide range of other baseline data available from ADNI.

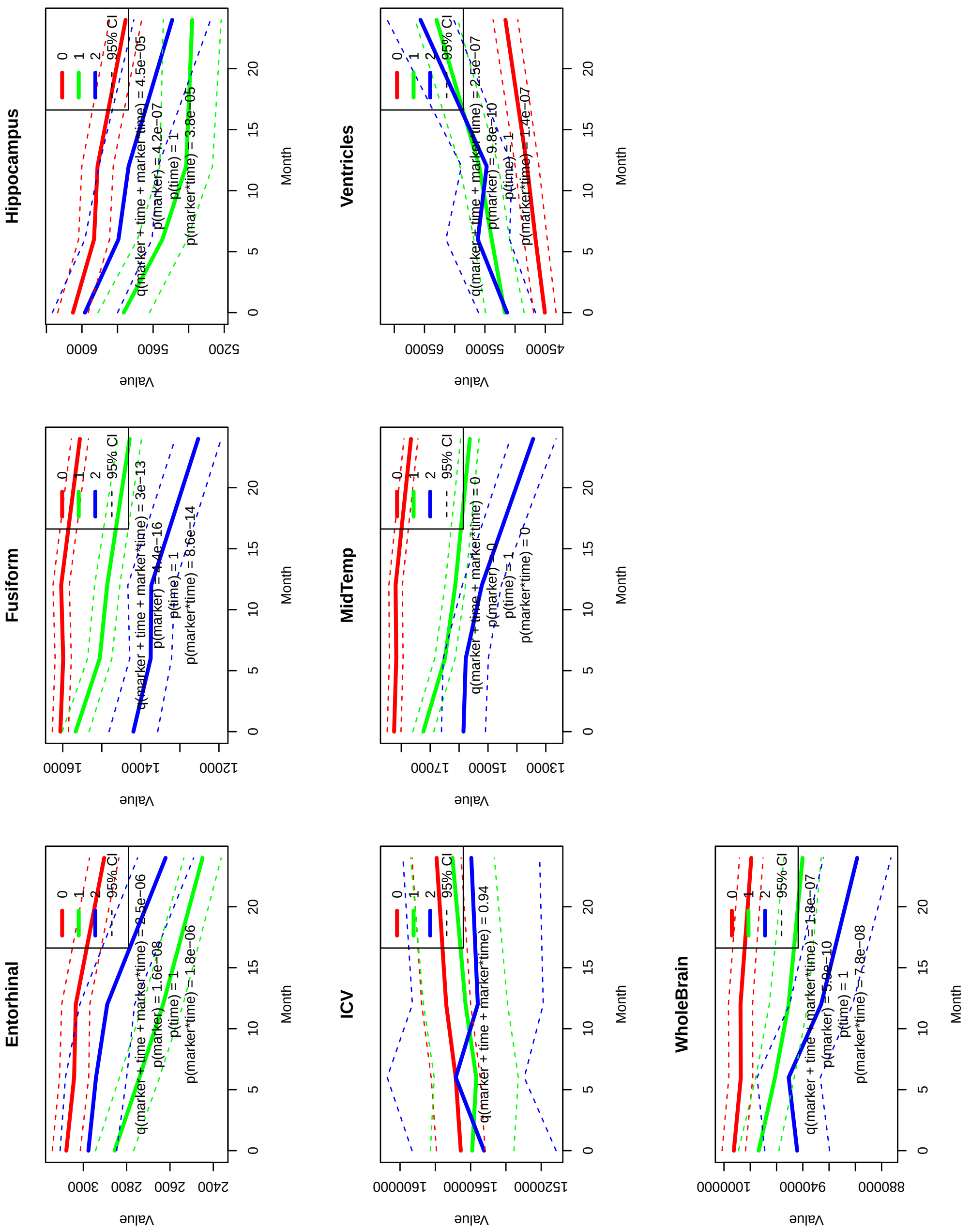

Figure 8. ADNI: associations of the VaDER clustering with a wide range of other longitudinal data available from ADNI.

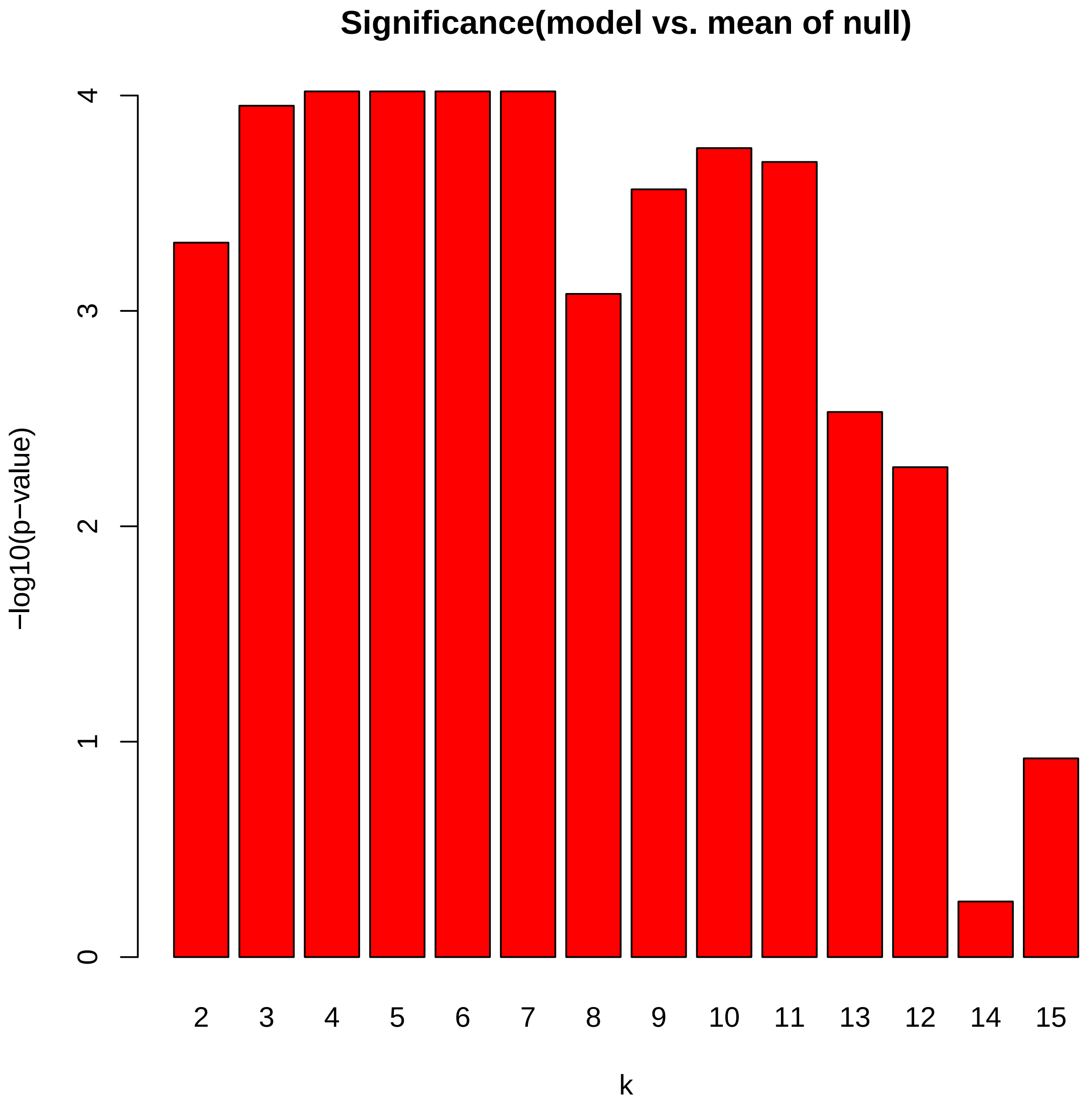

Figure 9. PPMI: prediction strength of VaDER for each *k* (blue) and the corresponding permutation-based null distribution.

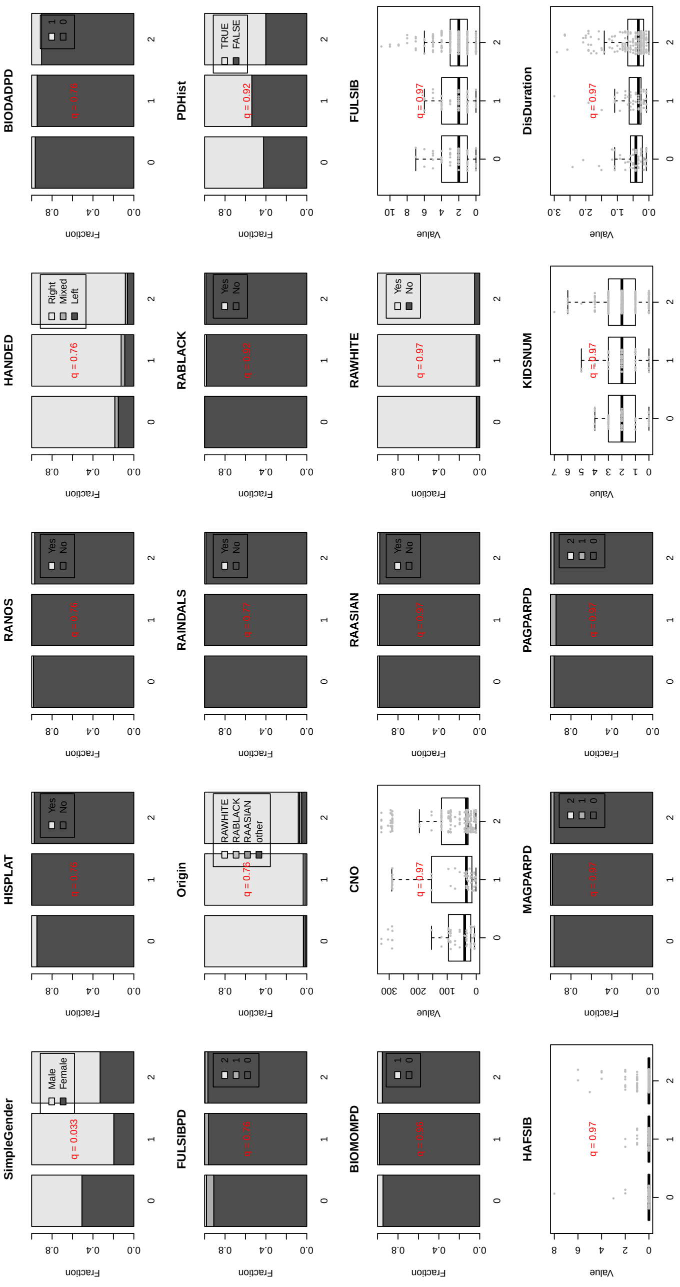

Figure 10. PPMI: associations of the VaDER clustering with a wide range of other baseline data available from PPMI.

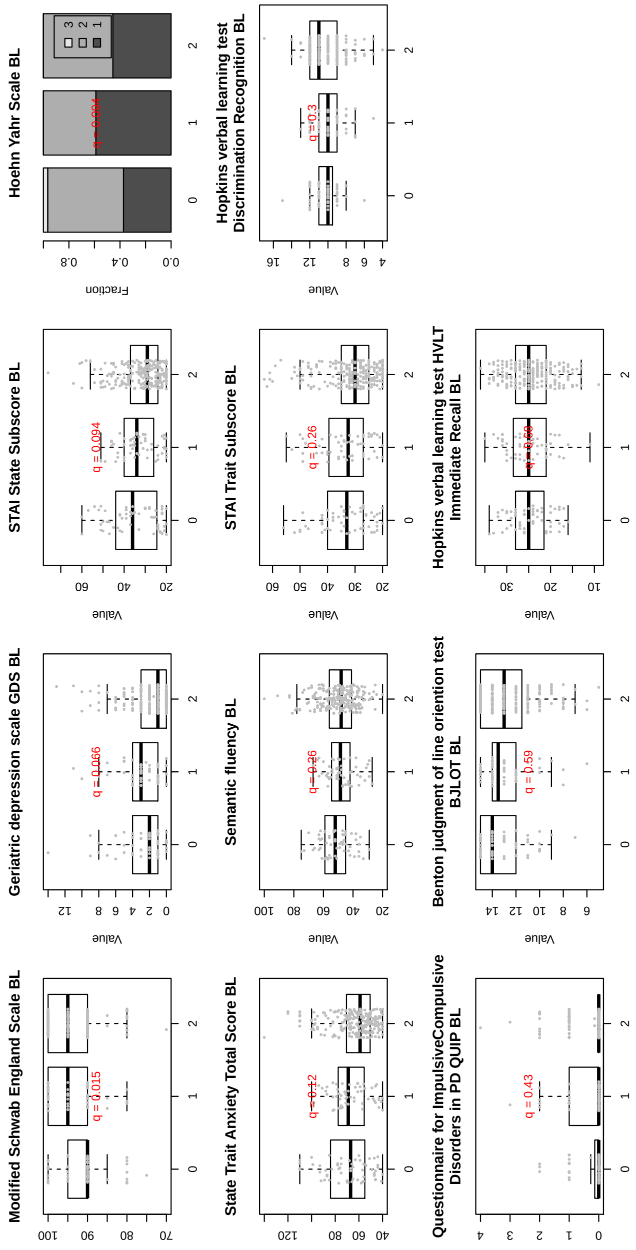

Figure 11. PPMI: associations of the VaDER clustering with a wide range of other baseline data available from PPMI.

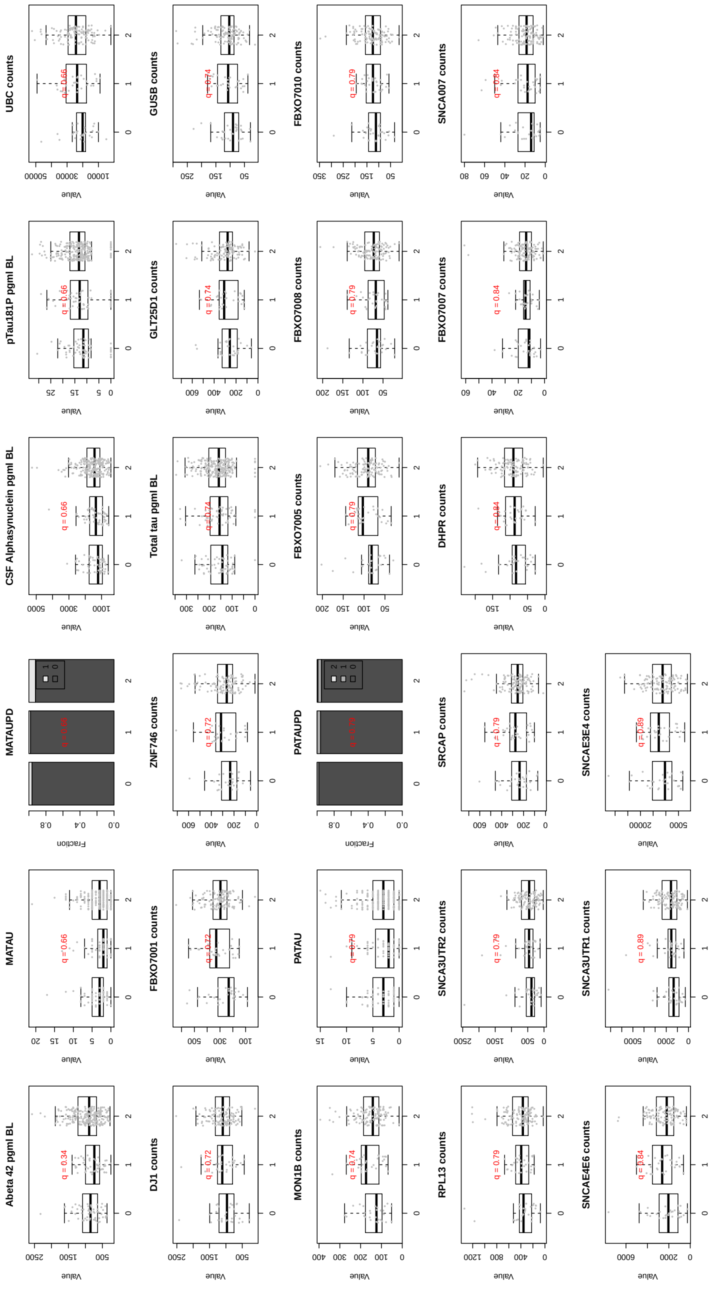

Figure 12. PPMI: associations of the VaDER clustering with a wide range of other baseline data available from PPMI.

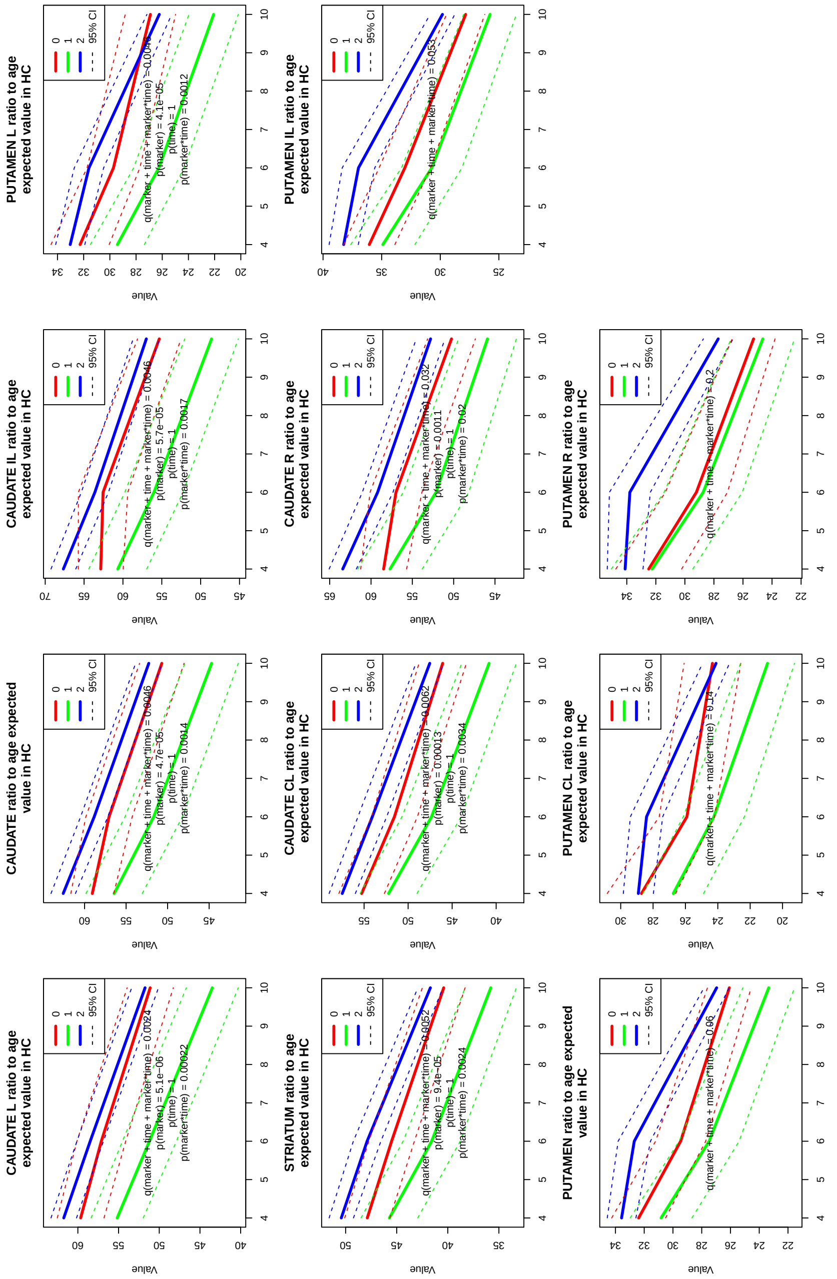

Figure 13. PPMI: associations of the VaDER clustering with a wide range of other longitudinal data available from PPMI.

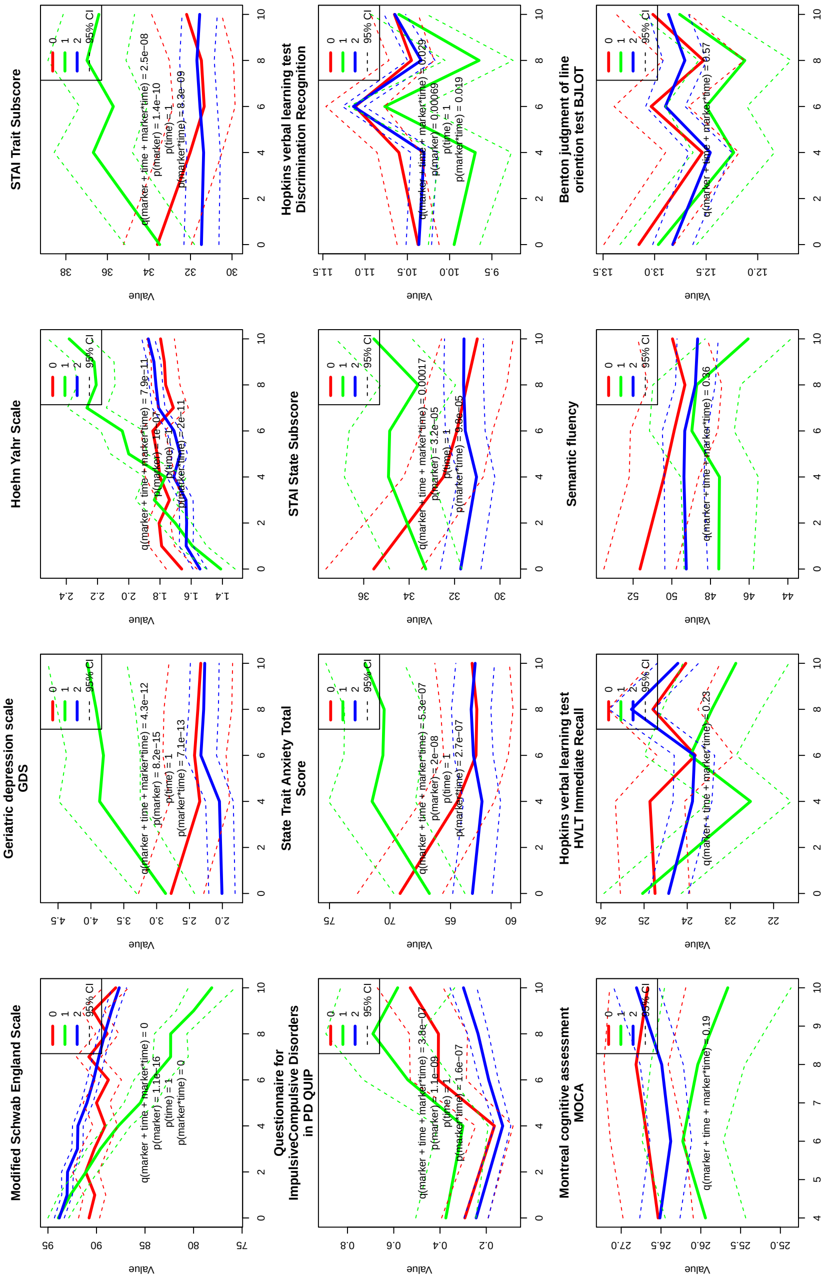

Figure 14. PPMI: associations of the VaDER clustering with a wide range of other longitudinal data available from PPMI.

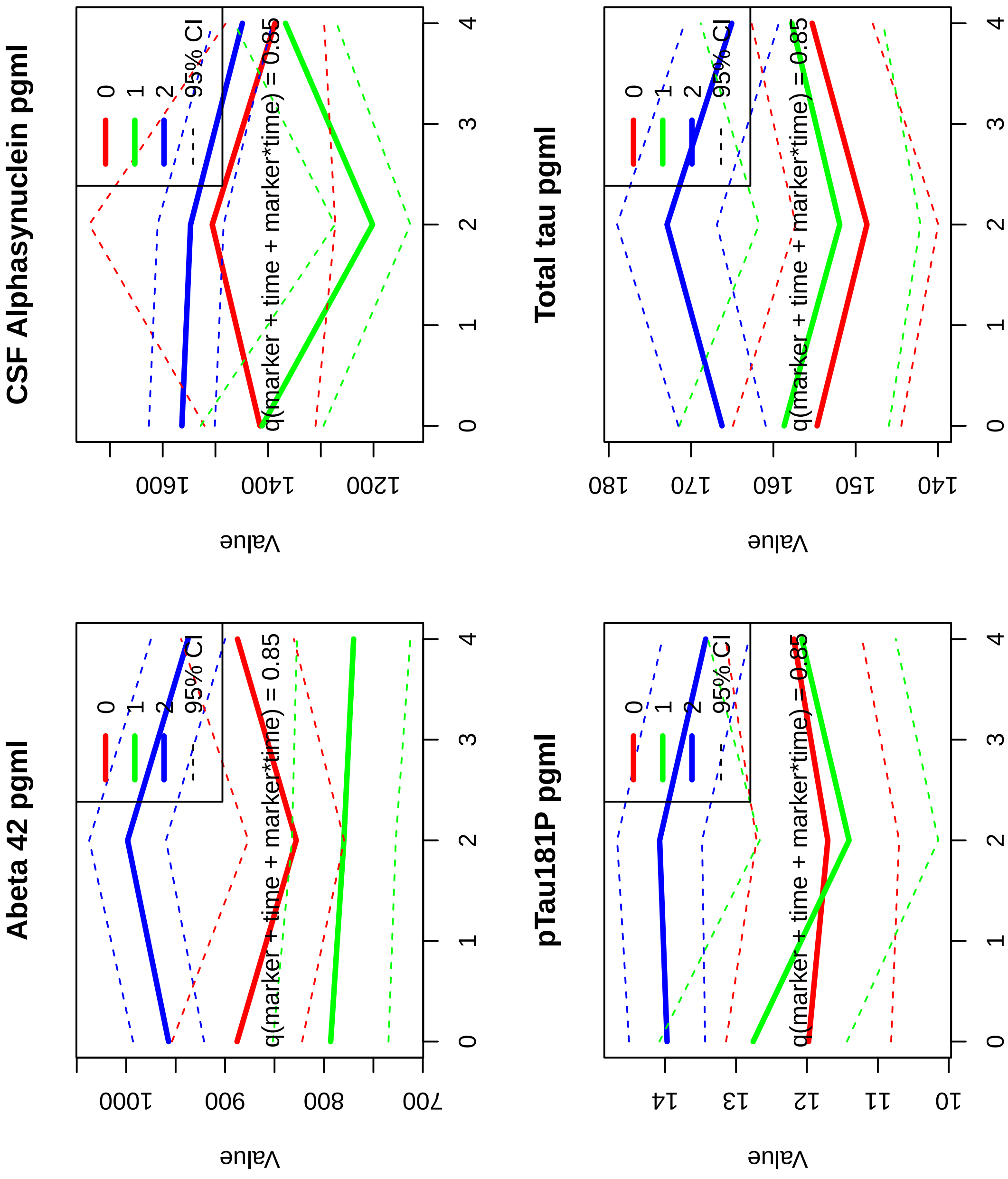

Figure 15. PPMI: associations of the VaDER clustering with a wide range of other longitudinal data available from PPMI.
