# Supplementary material for: Deep learning for clustering of multivariate clinical patient trajectories with missing values
Source: Gigascience. 2019 Nov 15;8(11):giz134. doi: 10.1093/gigascience/giz134 (PMC6857688; doi:10.1093/gigascience/giz134)
Supplement: giz134_Response_to_Reviewer_Comments_Original_Submission [file giz134_response_to_reviewer_comments_original_submission.pdf]

Author's Response To Reviewer Comments

Close

RESPONSE TO REVIEWER 1
Following the reviewer's suggestion, we extended the technical validation by including four real-world benchmark datasets for multivariate time series classification, to assess how well VaDER and other methods can recover the a priori known classes.
Morover, we extended the missingness analyses by adding experiments comparing VaDER's implicit imputation directly with pre-imputed inputs, for various degrees and modes of missingness.
Regarding comparing VaDER with other methods for multivariate (short) time series clustering, we appreciate the suggestions and references given by the reviewer, but after careful examination must conclude that VaDER can unfortunately not be directly compared with the methods in these references:
• Paparrizos et al.: k-shape does not support multivariate time series.
• Mikalsen et al.: While it would be very interesting to compare TCK to VaDER, unfortunately no free software implementation of TCK is available. Although the paper states that an R implementation is available, after a correspondence with the authors it appears this is not the case.
• Nazabal et al.: Although there is some resemblance in imputation techniques, HI-VAE was not designed for time series, and can therefore not be compared to VaDER.
To nonetheless address the reviewer's request for a more extensive comparison with other methods, we extended our comparison to include hierarchical clustering using two other distance/similarity measures specifically designed for multivariate time series: (1) multi-dimensional dynamic time warping (Tormene et al, 2008) and (2) global alignment kernels (Cuturi et al., 2011).
We agree with the reviewer that it is not clear whether VaDER performs better than the other methods on clustering the ADNI/PPMI data. We would however argue that it is not possible to unambiguously determine which method is better, because no ground-truth clustering is available for these two datasets.
Likewise, determining an unambiguously correct number of clusters is difficult. This strongly depends on the method, the data and the question of interest, the general treatment of which we consider outside the scope of our current work. We agree with the reviewer that if we were to use the gap between the null and the model, we would choose k = 2 for the ADNI data. However, we instead chose a number of clusters that we could demonstrate performs significantly better than expected by random chance, while still allowing VaDER enough flexibility to demonstrate its ability to uncover interactions between the time series. The ability to uncover interactions between variables is one of the main advantages of taking a multivariate approach to time series clustering, and hence one that we wish to highlight in this manuscript. By interactions, we mean e.g. distinguishing patients on one cognitive assessment that are indistinguishable on another cognitive assessment. We agree with the reviewer that this should have been better clarified in the manuscript, and we have therefore adjusted the text accordingly.

RESPONSE TO REVIEWER 2
While we cannot directly compare VaDER to VaDE, because VaDE does neither model time series nor handle missing values, we appreciate the reviewer's request for quantitatively assessing how implicit imputation affects the results. We therefore extended the missingness analyses by adding experiments comparing VaDER with implicit imputation directly to VaDER with pre-imputed inputs, for various degrees and modes of missingness.
We furthermore extended our method comparison to include hierarchical clustering using two other distance measures specifically designed for multivariate time series: (1) multi-dimensional dynamic time warping (Tormene et al, 2008) and (2) global alignment kernels (Cuturi et al., 2011).

Close
