# Supplementary material for: Deep learning for clustering of multivariate clinical patient trajectories with missing values
Source: Gigascience. 2019 Nov 15;8(11):giz134. doi: 10.1093/gigascience/giz134 (PMC6857688; doi:10.1093/gigascience/giz134)
Supplement: giz134_Reviewer_1_Report_Original_Submission — Alexandr Kalinin -- 7/26/2019 Reviewed [file giz134_reviewer_1_report_original_submission.pdf]

Reviewer Report

Title: Deep learning for clustering of multivariate clinical patient trajectories with missing values

Version: Original Submission **Date:** 7/26/2019

Reviewer name: Alexandr Kalinin

Reviewer Comments to Author:

Deep learning for clustering of multivariate clinical patient trajectories with missing values
The paper describes an approach for clustering multivariate short time series data with missing values using a modification of variational deep embedding (VaDE) autoencoder. It discusses applications of this model to recover known clusters in the simulated data and to stratify real-world Alzheimer's disease (AD) and Parkinson's disease (PD) patient data. The results show the ability of the model to these datasets into subgroups characterized by disease progression profiles. Overall, the paper is well written, has a clear structure, and may be of interest to a broad range of readers.
While reviewing existing solutions for time-series clustering, the authors identify few disadvantages: these methods rely on greater length of series, are not suited for datasets with large numbers of missing values, and may have higher error rate if imputation and clustering are performed as separate steps. The proposed approach is claimed to address these limitations.
This approach is an extension of VaDE model, which is capable of simultaneous learning of latent representations and cluster assignments from input data. The authors extend VaDE by: 1) applying LSTMs to both inputs and outputs of the autoencoder, and 2) using weighted reconstruction loss and an additional layer for learnable imputation. The authors claim that in comparison to HI-VAE [35] this architecture can handle values missing not at random. While the methodological component of the approach is technically sound, the experimental results could be improved in order to strengthen the support for the claims made in the Background section.
Methods and data preparation methodologies are described in sufficient detail. Since the main contribution of the paper is a modified model architecture it is good practice to run ablation tests and compare to other models in order to demonstrate the benefits of the proposed approach. For example, the authors note that in contrast to [34] and other existing approaches, VaDER used missingness indicators for implicit imputation as an integral part of neural network training. This claim could be better supported with an experiment on, for example, using VAR simulated data with various amounts and modes of missingness to compare the performance of VaDER and LSTM with pre-imputed inputs. It would be also helpful to directly compare VaDER with other models on various simulated and real-world data. Examples of these models could include openly available implementations of k-Shape (Paparrizos and Gravano, 2015), Time series cluster kernel (Mikalsen et al., 2018), and HI-VAE (Nazabal et al., 2018) - please see corresponding GitHub URLs in the Reference section of this review. As an intermediate evaluation step between simulated data and datasets with multiple clinical outcomes, it would be useful to test the model on few labeled datasets. Even if clusters obtained in unsupervised manner are not fully representative of data partitioning by the label variable, it still can serve as a useful proxy for comparing different models with a fixed known number of clusters, e.g., see (Mikalsen et al., 2018) and (Sai

Madiraju et al., 2018). It also possible to evaluate model robustness by varying levels and types of value missingness. This type of performance analysis is needed to support the claim that VaDER architecture allow for better handling short time-series data with many missing values.
Overall, the results on the ADNI and PPMI data are convincing and demonstrate the ability of VaDER identify clusters of patients that can have a meaningful connection with their disease progression. It is, again, unclear whether this particular approach performs than other recently proposed ones. It would be interesting to expand on the choice of k in each case. It seems like on ADNI data, the biggest gap between null and model prediction strength is at k=2, but then it decreases drastically with the increase of k from 2 to 6. While for PPMI data there is a noticeable difference between the model and the null until k=8. As these parameters change from dataset to dataset, it would be helpful to provide a guideline for choosing k in various situations.

References:

Mikalsen, Karl Ã˜yvind, et al. "Time series cluster kernel for learning similarities between multivariate time series with missing data." Pattern Recognition 76 (2018): 569-581. https://github.com/kmi010/Time-series-cluster-kernel-TCK-

Nazabal, Alfredo, et al. "Handling incomplete heterogeneous data using vaes." arXiv preprint arXiv:1807.03653 (2018). https://github.com/probabilistic-learning/HI-VAE

Paparrizos, John, and Luis Gravano. "k-shape: Efficient and accurate clustering of time series." Proceedings of the 2015 ACM SIGMOD International Conference on Management of Data. ACM, 2015. https://github.com/Mic92/kshape

Sai Madiraju, Naveen, et al. "Deep Temporal Clustering: Fully Unsupervised Learning of Time-Domain Features." arXiv preprint arXiv:1802.01059 (2018).

Level of Interest

Please indicate how interesting you found the manuscript: Choose an item.

Quality of Written English

Please indicate the quality of language in the manuscript: Choose an item.

Declaration of Competing Interests

Please complete a declaration of competing interests, considering the following questions:

- Have you in the past five years received reimbursements, fees, funding, or salary from an organisation that may in any way gain or lose financially from the publication of this manuscript, either now or in the future?
- Do you hold any stocks or shares in an organisation that may in any way gain or lose financially from the publication of this manuscript, either now or in the future?

- Do you hold or are you currently applying for any patents relating to the content of the manuscript?
- Have you received reimbursements, fees, funding, or salary from an organization that holds or has applied for patents relating to the content of the manuscript?
- Do you have any other financial competing interests?
- Do you have any non-financial competing interests in relation to this paper?

If you can answer no to all of the above, write 'I declare that I have no competing interests' below. If your reply is yes to any, please give details below.

I declare that I have no competing interests

I agree to the open peer review policy of the journal. I understand that my name will be included on my report to the authors and, if the manuscript is accepted for publication, my named report including any attachments I upload will be posted on the website along with the authors' responses. I agree for my report to be made available under an Open Access Creative Commons CC-BY license (http://creativecommons.org/licenses/by/4.0/). I understand that any comments which I do not wish to be included in my named report can be included as confidential comments to the editors, which will not be published.

Choose an item.

To further support our reviewers, we have joined with Publons, where you can gain additional credit to further highlight your hard work (see: https://publons.com/journal/530/gigascience). On publication of this paper, your review will be automatically added to Publons, you can then choose whether or not to claim your Publons credit. I understand this statement.

Yes Choose an item.
